# Neoadjuvant stereotactic body radiation therapy with durvalumab and oleclumab in ER^+^HER2^−^ breast cancer: a randomized phase 2 trial

**DOI:** 10.1038/s41591-026-04453-z

**Published:** 2026-06-25

**Authors:** Alex De Caluwé, Isabelle Desmoulins, Kim Cao, Vincent Remouchamps, Adinda Baten, Eleonore Longton, Karine Peignaux, Andrea Joaquin Garcia, David Venet, Luca Arecco, Elisa Agostinetto, Guilherme Nader-Marta, Zoë Denis, Jennifer Dhont, Paulus Kristanto, Xavier Catteau, Denis Larsimont, Roberto Salgado, Philip Poortmans, John Stagg, Christos Sotiriou, Martine Piccart, Michail Ignatiadis, Emanuela Romano, Laurence Buisseret

**Affiliations:** 1 Institut Jules Bordet, Hôpitaux Universitaires de Bruxelles (H.U.B), Université Libre de Bruxelles (ULB), Brussels, Belgium; 2 Centre Georges-François Leclerc, Université Bourgogne Europe, Dijon, France; 3 Institut Curie, Paris, France; 4 CHU St Elisabeth, Namur, Belgium; 5 Universitaire Ziekenhuizen Leuven, Leuven, Belgium; 6 Hôpital Universitaire St Luc, Brussels, Belgium; 7 ZAS Hospitals, Antwerp, Belgium; 8 Peter Mac Callum Cancer Centre, Melbourne, Victoria Australia; 9 Iridium Netwerk, Antwerp, Belgium; 10 University of Antwerp, Antwerp, Belgium; 11 Goodman Cancer Institute, McGill University, Montreal, Quebec Canada

**Keywords:** Randomized controlled trials, Translational research, Breast cancer

## Abstract

Patients with estrogen receptor-positive (ER^+^), HER2-negative, early breast cancer (BC) have low pathologic complete response (pCR) rates following neoadjuvant chemotherapy. Immune checkpoint inhibitors (ICIs) provide limited benefit in programmed death-ligand 1 (PD-L1)-negative tumors, characterized by an immune-cold tumor microenvironment. Here we hypothesized that immune-modulating stereotactic body radiation therapy (iSBRT; 3 × 8 Gy) could enhance response through tumor microenvironment reprogramming, and that CD73 blockade could further improve efficacy. We conducted a phase 2, randomized, multicenter trial (Neo-CheckRay) in 147 female patients with high-risk, ER^+^HER2^−^ early BC. Patients received neoadjuvant chemotherapy plus iSBRT alone (No_ICI), with anti-PD-L1 durvalumab (Single_ICI) or with durvalumab plus anti-CD73 oleclumab (Double_ICI). In the intention-to-treat population, the primary endpoint, residual cancer burden 0/1 rate, was 35.4% with No_ICI, 45.1% with Single_ICI and 47.9% with Double_ICI, without statistically significant differences. pCR rates were 16.7%, 29.4% and 33.3%, respectively (*P* = 0.059). In the per-protocol population (MammaPrint High Risk, *n* = 131), pCR rates were 16.3%, 32.6% and 35.6%, respectively (*P* = 0.040). Among PD-L1-negative tumors (*n* = 91), pCR rates were 3.4%, 28.1% and 30.0%, respectively. No new safety signals were observed. Baseline transcriptomic analysis showed low immune signature expression in PD-L1-negative tumors. Paired baseline and on-treatment biopsies obtained 1 week after iSBRT demonstrated tumor microenvironment reprogramming toward an inflamed phenotype in the iSBRT + anti-PD-L1 arms. These findings suggest that iSBRT + anti-PD-L1 may convert immune-cold ER^+^HER2^−^ BC into more inflamed tumors and improve response, particularly in PD-L1-negative disease. ClinicalTrials.gov registration: NCT03875573.

## Abstract (web summary)

In the randomized phase 2 Neo-CheckRay trial, patients with early-stage ER^+^HER2^−^ breast cancer received neoadjuvant immune-modulating stereotactic body radiation therapy with or without durvalumab, and with or without the anti-CD73 antibody oleclumab, leading to encouraging clinical responses when durvalumab was added including in patients with PD-L1-negative tumors.

## Main

In 2022, over 2.3 million cases of breast cancer (BC) were diagnosed worldwide, with 70% being the ER^+^HER2^−^ subtype^1^. The ER^+^HER2^−^ subtype varies in treatment responses and outcomes due to differences in proliferation rate, immune infiltration and ER/progesterone receptor expression^2^. Within ER^+^HER2^−^ cases, the high-risk luminal B-like subtype can be identified using tumor grade, Ki67 and gene-expression signatures^3,4^. Current treatments for this high-risk subgroup of early-stage BC include (neo)adjuvant chemotherapy; surgery; adjuvant radiation therapy (RT) to the chest wall, breast and elective nodal regions; followed by endocrine therapy with or without cyclin-dependent kinase 4/6 inhibitors and poly(ADP-ribose) polymerase inhibitors (PARPi)^5^. Despite these treatments, more than 40% of the patients will develop a local or distant recurrence within 5 years, underscoring the need for developing new therapeutic strategies in high-risk ER^+^HER2^−^ BC^6–8^.

Immunotherapy with PD-1/PD-L1 ICIs has been investigated in combination with neoadjuvant chemotherapy (NACT) in early-stage BC. In triple-negative BC, the addition of anti-PD-1 to NACT increased pCR by 13.6% and improved overall survival by 5.0% at 5 years^9,10^. In high-risk ER^+^HER2^−^ BC, the addition of anti-PD-(L)1 to NACT increased pCR by 8.7–10.7% and long-term outcome data are awaited^11–13^. Unlike triple-negative BC, the tumor microenvironment (TME) in ER^+^HER2^−^ BC is generally less inflamed with a lower presence of tumor-infiltrating lymphocytes (TILs) and lower PD-L1 expression, which may contribute to its lowered sensitivity to ICI^14,15^. RT has emerged as a promising strategy to enhance the efficacy of ICI by transforming an immune-cold TME into a more immune-responsive environment^16–18^. Preclinical and clinical studies suggest that RT can modulate the immune response, enhance tumor immunogenicity and improve recognition of tumor cells by the immune system^19^. In the neoadjuvant context, iSBRT can be precisely targeted at the primary tumor, while sparing the tumor-draining lymph nodes to avoid disrupting the early immune response^20,21^. However, RT may also induce immunosuppressive pathways, such as upregulation of cluster of differentiation 73 (CD73), an ecto-enzyme that converts ATP released by dying tumor cells into adenosine^22^. Extracellular adenosine functions as a metabolic immune checkpoint by decreasing the anti-tumor activity of immune cells and by improving the survival and metastatic characteristics of tumor cells^23^. CD73 is expressed on tumor cells, stromal cells and immune cells in the TME and is associated with poor outcomes in multiple solid cancers^24,25^.

The Neo-CheckRay trial aimed to evaluate whether combining NACT, iSBRT and ICIs (anti-PD-L1 ± anti-CD73) improves pathological response rates in luminal B-like (MammaPrint High Risk) BC. Additional objectives included assessing the safety of the new treatment combination and evaluating early dynamic changes in the TME by comparing on-treatment biopsies taken 1 week after iSBRT (week 6) with baseline biopsies. In the trial, iSBRT (24 Gy in three fractions to the primary BC) was given concomitantly with NACT (No_ICI arm) ± the anti-PD-L1 durvalumab (Single_ICI arm) ± the anti-CD73 oleclumab (Double_ICI arm). The experimental neoadjuvant treatment combination was followed by surgery and adjuvant treatment according to standard of care, excluding a boost to the tumor bed during adjuvant RT. Here, we report the results of the primary endpoint (residual cancer burden (RCB) 0/1 rate) and the secondary endpoint (pCR rate), both evaluated at surgery. We also present analyses of baseline biomarkers, including PD-L1 immunohistochemistry (IHC) expression and TIL levels, and characterize early biomarker dynamics using paired tumor biopsies obtained at baseline and 1 week after iSBRT, assessed by both IHC and RNA sequencing.

## Results

### Patients and treatment

**Fig. 1:**
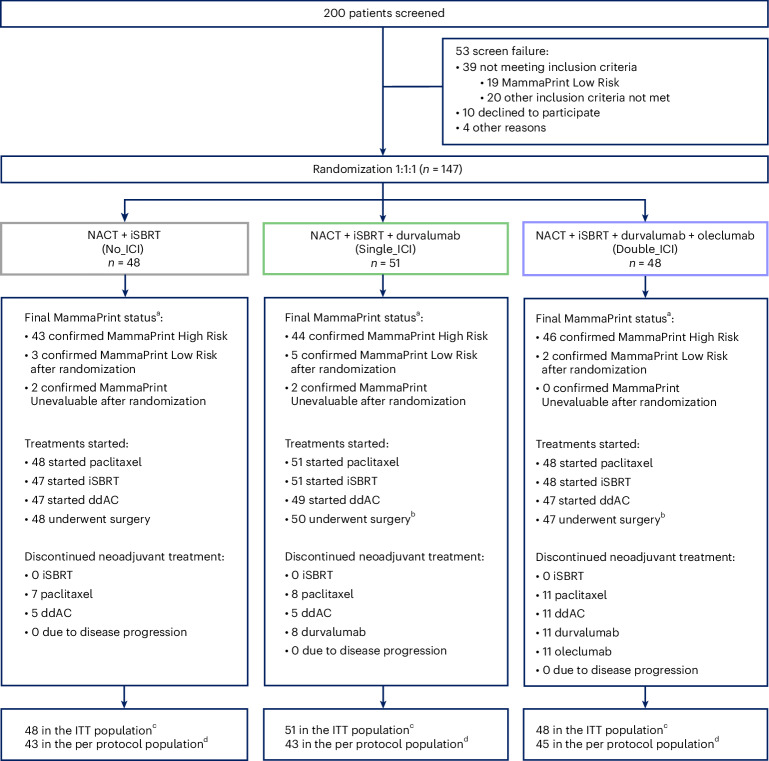
Flow chart showing patient disposition. ᵃIf the MammaPrint result was not available by the end of the screening period and in presence of clinical high-risk characteristics, patients were allowed to be randomized as a ‘MammaPrint Unknown’ participant. Clinical high-risk patients were defined by the following criteria: for patients <50 years old, a tumor with a Ki67 > 20% or grade III was mandatory; for patients ≥50 years, a tumor with a Ki67 > 20% or grade III and node-positive disease was required. These criteria were required in addition to the standard inclusion and exclusion criteria. Once the MammaPrint result became available, patients who had been previously randomized as ‘MammaPrint Unknown’ were assigned a final MammaPrint status as MammaPrint High Risk, MammaPrint Low Risk or MammaPrint Unevaluable. Patients with a MammaPrint status Low Risk that was available before randomization were not allowed to enter the trial. MammaPrint Unevaluable were cases for which the MammaPrint testing was inconclusive due to technical failure or insufficient tumoral material on the biopsy. MammaPrint testing was allowed to be repeated on a new biopsy. ᵇFor one patient in the Single_ICI arm the result at surgery (RCB score) was not available; and one patient in the Double_ICI arm withdrew her consent for trial participation and data collection before reaching the surgery timepoint. Patients without result at surgery were counted as nonresponders in the ITT analysis. ᶜThe ITT population consists of all randomized patients, regardless of their MammaPrint status and regardless of whether the treatment was started or discontinued. In case the result at surgery was not available, the patient was considered as a nonresponder for the analysis. ᵈThe per-protocol population consists of the MammaPrint High Risk patients for whom an RCB score is available. All patients who initiated neoadjuvant treatment were included in the per-protocol population, irrespective of whether study treatments were discontinued. Source data

From 15 June 2021 to 11 March 2024, 200 patients were screened across seven sites in Belgium and France. Of the 200 screened patients, 147 were randomized to the No_ICI arm (48 patients), the Single_ICI arm (51 patients) or the Double_ICI arm (48 patients). The intention-to-treat (ITT) population comprises all randomized patients, regardless of whether the treatment was started or not. The predefined per-protocol population consists of patients identified as High Risk according to the MammaPrint gene-expression signature. Figure 1 presents the CONSORT flow diagram, and the study design is shown in Supplementary Fig. 1 (ref. ^26^).

In the ITT population, patient demographics and clinical characteristics were evenly distributed among the three treatment arms (Table 1). The median duration of neoadjuvant treatment was 5 months in the three arms. Breast-conserving surgery was performed in 73.3% (68.9% in No_ICI, 80.0% in Single_ICI and 71.1% in Double_ICI). At baseline, 59.2% of patients were node-positive, and at surgery an axillary lymph node dissection was performed in 60.9% (62.5% in No_ICI, 62.7% in Single_ICI and 58.3% in Double_ICI). Patients without axillary lymph node dissection had a sentinel lymph node biopsy. The database lock for all analyses was 4 December 2025.

**Table 1:** Baseline patient demographics and clinical characteristics (ITT population, *n* = 147)

		NACT + iSBRT ‘No_ICI’ (*n* = 48)	NACT + iSBRT + durvalumab ‘Single_ICI’ (*n* = 51)	NACT + iSBRT + durvalumab + oleclumab ‘Double_ICI’ (*n* = 48)
Patient characteristics				
Age, years	Median (IQR)	50 (41.75; 57)	48 (42; 54)	48.5 (41; 57.5)
Menopausal status, *n* (%)	Premenopausal Postmenopausal	30 (62.5%) 18 (37.5%)	37 (72.55%) 14 (27.45%)	28 (58.33%) 20 (41.67%)
BMI	Median (IQR)	24.3 (22.5; 27.8)	25 (23.15; 29.575)	27 (23; 31.6)
ECOG PS, *n* (%)	0 1	44 (91.67%) 4 (8.33%)	47 (92.16%) 4 (7.84%)	46 (95.83%) 2 (4.17%)
Tumor characteristics				
T-stage, *n* (%)^a,b^	T1–T2 T3	39 (81.25%) 9 (18.75%)	36 (70.59%) 15 (29.41%)	38 (79.17%) 10 (20.83%)
N-stage, *n* (%)^a,b^	N0 N1 N2 N3	19 (39.58%) 26 (54.17%) 3 (6.25%) 0	21 (41.18%) 24 (47.06%) 4 (7.84%) 2 (3.92%)	20 (41.67%) 23 (47.92%) 3 (6.25%) 2 (4.17%)
PD-L1 IC score, *n* (%)^a,c^	<1% ≥1%	29 (60.42%) 19 (39.58%)	32 (62.75%) 19 (37.25%)	30 (62.5%) 18 (37.5%)
PD-L1 CPS, *n* (%)	<1 ≥1	31 (64.58%) 17 (35.42%)	33 (64.71%) 18 (35.29%)	30 (62.5%) 18 (37.5%)
MammaPrint index, *n* (%)^d^	Ultra-High (MP2) High (MP1) Low Unevaluable	15 (31.25%) 28 (58.33%) 3 (6.25%) 2 (4.17%)	8 (15.69%) 36 (70.59%) 5 (9.8%) 2 (3.92%)	13 (27.08%) 33 (68.75%) 2 (4.17%) 0
MammaPrint index^d^	Median (IQR)	−0.3195 (−0.69275; −0.1975)	−0.389 (−0.512; −0.182)	−0.391 (−0.641; −0.24375)
BluePrint, *n* (%)^d^	Luminal Basal Unevaluable	40 (83.33%) 6 (12.5%) 2 (4.17%)	47 (92.16%) 2 (3.92%) 2 (3.92%)	43 (89.58%) 5 (10.42%) 0
Histologic subtype	NST Lobular	47 (97.02%) 1 (2.08%)	49 (96.99%) 2 (3.01%)	47 (97.87%) 1 (2.13%)
Histologic grade, *n* (%)^e^	Grade 2 Grade 3	19 (39.58%) 29 (60.42%)	32 (62.75%) 19 (37.25%)	24 (50%) 24 (50%)
ER, *n* (%)	ER negative (0%) ER low (1–10%) ER intermediate (10–50%) ER high (≥50%)	0 0 6 (12.5%) 42 (87.5%)	0 0 3 (5.88%) 48 (94.12%)	0 1 (2.08%) 4 (8.33%) 43 (89.58%)
PR, *n* (%)	PR negative (0%) PR low (1–10%) PR intermediate (10–50%) PR high (≥50%)	8 (16.67%) 16 (33.33%) 4 (8.33%) 20 (41.67%)	4 (7.84%) 13 (25.49%) 3 (5.88%) 31 (60.78%)	8 (16.67%) 6 (12.5%) 10 (20.83%) 24 (50%)
Ki67%^e^	Median (IQR)	30.0% (25.0%; 60.0%)	33.5% (25.0%; 60.0%)	30.0% (20.0%; 61.3%)
sTILs, *n* (%)^f^	<1% ≥1% Unevaluable	23 (47.92%) 25 (52.08%) 0	27 (52.94%) 22 (43.14%) 2 (3.92%)	24 (50%) 24 (50%) 0
Multifocal, *n* (%)	No Yes	33 (68.75%) 15 (31.25%)	40 (78.43%) 11 (21.57%)	32 (66.67%) 16 (33.33%)
Laterality, *n* (%)	Bilateral Left Right	1 (2.08%) 27 (56.25%) 20 (41.67%)	2 (3.92%) 21 (41.18%) 28 (54.9%)	3 (6.25%) 20 (41.67%) 25 (52.08%)

Data are from the ITT population. All patients had previously untreated, centrally confirmed ER^+^, HER2^−^ disease and all patients were female based on biological sex. BMI, body mass index; CPS, combined positive score; ECOG PS, Eastern Cooperative Oncology Group performance status; IQR, interquartile range; NST, no special subtype; PR, progesterone receptor.

^a^Stratification factors.

^b^American Joint Committee on Cancer Staging Manual, 7th edition.

^c^PD-L1-expressing tumor-infiltrating immune cells as percentage of tumor area using the VENTANA SP263 assay, per central assessment.

^d^Centrally assessed by Agendia.

^e^Locally assessed.

^f^sTILs were centrally assessed following the 2014 guidelines from the International Tumor Infiltrating Lymphocyte Working Group.

### Efficacy

**Fig. 2:**
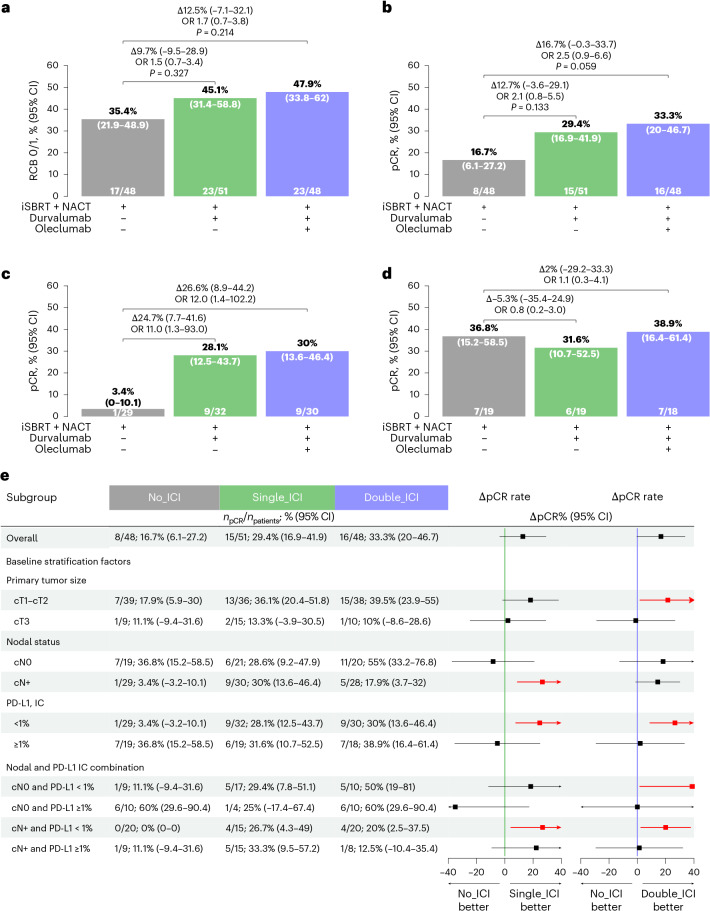
Efficacy endpoints in the ITT population (*n* = 147). The proportion difference is presented with unpooled Wald 95% CIs assuming independent proportions. ORs and their Wald 95% CIs were estimated from logistic regression. The *P* values are based on chi-squared tests. Bonferroni correction was applied for multiple comparisons of the RCB 0/1 as the primary endpoint; therefore, *α* = 0.025 to compare Single_IC versus No_ICI and Double_IC versus No_ICI. No *α* correction was specified for other analyses. The PD-L1 IC score is defined as the percentage of the tumor area occupied by PD-L1-positive immune cells and was assessed using the VENTANA SP263 IHC assay. **a**–**d**, Bar plots depicting RCB 0/1 (**a**) and pCR rates in the overall (**b**) and by subgroups defined by PD-L1-negative (**c**) and PD-L1-positive (**d**). Rates of RCB 0/1 and pCR (total, PD-L1-negative and PD-L1-positive) in the per-protocol population are shown in Extended Data Fig. 2. **e**, Forest plot showing between-arms differences in percentages of patients with a pCR according to the stratification factors cT (cT1c-T2 versus cT3), cN (cN0 versus cN+) and PD-L1 (<1% versus ≥1%), and of the combinations of the stratification factors cN and PD-L1. Points represent proportion differences; horizontal bars denote unpooled Wald 95% CIs for the difference of independent proportions. Variables with statistically significant differences in pCR rate between study arms are highlighted in red. OR, odds ratio. Source data

**Extended Data Fig. 1:**
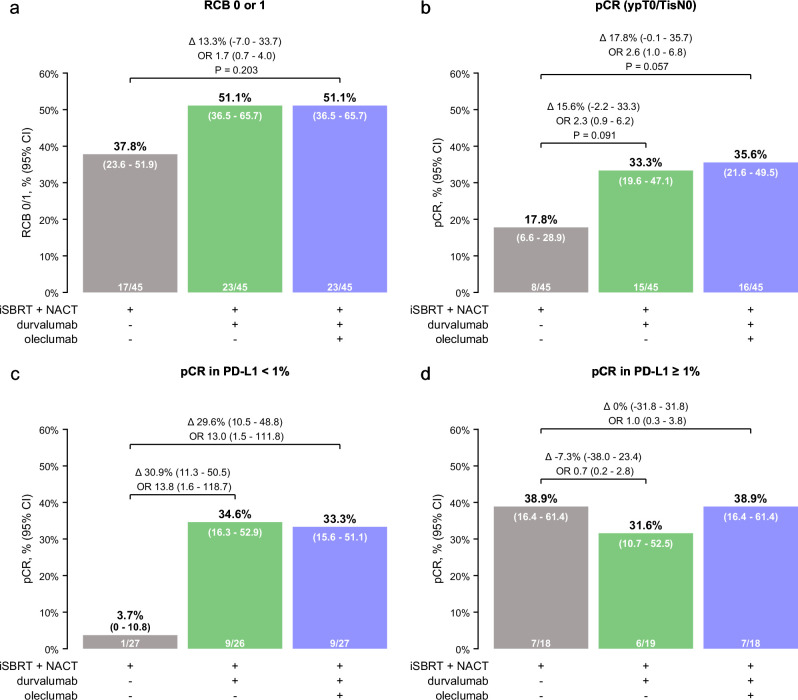
RCB and pCR outcomes in the per-protocol cohort, comprising only patients classified as MammaPrint High Risk (n = 131). The proportion difference is presented with unpooled Wald 95% confidence intervals assuming independent proportions. Odds ratios and their Wald 95% confidence intervals were estimated from logistic regression. The p-values are based on Chi-Square tests. Bonferroni correction was applied for multiple comparisons of the RCB 0/1 as the primary endpoint; therefore α = 0.025 to compare Single_ICI vs No_ICI and Double_ICI vs No_ICI. No α correction was specified for other analyses. **a**, bar plots depicting RCB 0/1 and **b**, pCR rates in the overall and by subgroups defined by **c**, PD-L1-negative and **d**, PD-L1-positive. The PD-L1 IC (immune cell) score is defined as the percentage of the tumor area occupied by PD-L1–positive immune cells and was assessed using the VENTANA SP263 immunohistochemistry assay. *Abbreviations*: iSBRT, immune-modulating stereotactic body radiation therapy; pCR, pathological complete response; PD-L1, programmed death-ligand 1; RCB, residual cancer burden. Source data

Among the 147 patients in the ITT population, the primary endpoint RCB 0/1 rate was 35.4% in No_ICI (95% confidence interval (95% CI), 21.9–48.9), 45.1% in Single_ICI (95% CI, 31.4–58.8) and 47.9% in Double_ICI (95% CI, 33.8–62.0) (No_ICI versus Double_ICI: *P* = 0.21) (Fig. 2a). The proportion of patients who achieved a pCR (ypT0/Tis, ypN0) was 16.7% in No_ICI (95% CI, 6.1–27.2), 29.4% in Single_ICI (95% CI, 20.0–46.7) and 33.3% in Double_ICI (95% CI, 21.6–49.5) (No_ICI versus Double_ICI: *P* = 0.059) (Fig. 2b). pCR (ypT0/Tis, ypN0) is defined as the absence of residual invasive disease in the breast and lymph nodes at surgery, while allowing the presence of residual in situ carcinoma in the breast. Among the 131 patients in the predefined per-protocol population, a significant increase in pCR rate was seen in the Double_ICI arm: 16.3% in No_ICI (95% CI, 5.2–27.3), 32.6% in Single_ICI (95% CI, 18.6–46.6) and 35.6% in Double_ICI (95% CI, 21.6–49.5) (No_ICI versus Double_ICI: *P* = 0.04) (Extended Data Fig. 1).

**Extended Data Fig. 2:**
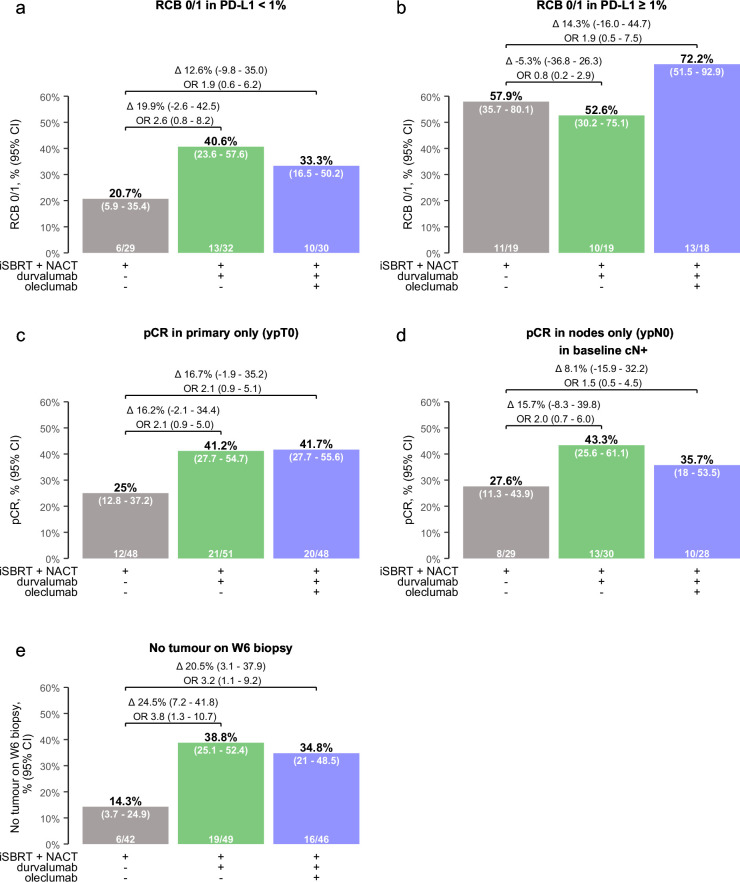
RCB and pCR in subgroups, and result of week 6 biopsy (ITT population, n = 147). The PD-L1 IC (immune cell) score is defined as the percentage of the tumor area occupied by PD-L1–positive immune cells and was assessed using the VENTANA SP263 immunohistochemistry assay. The proportion difference is presented with unpooled Wald 95% confidence intervals assuming independent proportions. Odds ratios and their Wald 95% confidence intervals were estimated from logistic regression. The p-values are based on Chi-Square tests. No α correction was applied for multiple comparisons. Bar plots depicting RCB 0/1 in the **a**, PD-L1-negative subgroup and **b**, PD-L1-positive subgroup. **c**, pCR rate in the primary breast cancer, irrespective of the response in the nodes. **d**, pCR in the nodes, irrespective of the response in the primary breast cancer. **e**, proportions of patients with absence of tumor on the week 6 biopsy. *Abbreviations*: iSBRT, immune-modulating stereotactic body radiation therapy; pCR, pathological complete response; PD-L1, programmed death-ligand 1; RCB, residual cancer burden. Source data

Subgroup analyses were predefined on the stratification factors, and consisted of PD-L1 status, tumor stage and lymph node status. PD-L1 was prospectively and centrally assessed before randomization using the VENTANA IHC SP263 assay and scored as <1% versus ≥1% according to the Immune Cell (IC) score, defined as the percentage of the tumor area occupied by PD-L1-positive immune cells. Tumors with IC score <1% were defined as PD-L1-negative and those with IC score ≥1% as PD-L1-positive. Among patients with PD-L1-negative tumors (*n* = 91, 61.9% of the ITT population), those treated with ICI experienced significantly higher pCR rates, compared with patients treated with iSBRT + NACT only (No_ICI). The pCR rates were: 3.4% in No_ICI (95% CI, 0–10.1), 28.1% in Single_ICI (95% CI, 12.5–43.7) and 30.0% in Double_ICI (95% CI, 13.6–46.4) (Fig. 2c). In contrast, in the PD-L1-positive subgroup no differences in pCR rates were observed between the three arms (Fig. 2d). T1 and T2 tumor stages achieved higher pCR rates in the three arms compared with T3 (Fig. 2e). Patients with baseline node-positive disease (cN+) benefited more from the addition of ICI (Single_ICI or Double_ICI) in comparison with patients with baseline node-negative disease (cN0). When PD-L1 status and nodal status were combined, significant benefit was observed with Single_ICI and Double_ICI in node-positive patients with PD-L1-negative tumors (Fig. 2e). Rates of RCB 0/1 by PD-L1 status, pCR in primary tumor only and nodal response only are detailed in Extended Data Fig. 2. As an exploratory analysis, the difference in pCR proportions between the Single_ICI and Double_ICI arms in the overall population and across stratification factors is presented in Supplementary Fig. 2, together with the corresponding confidence intervals.

At the data cut-off, after a median follow-up of 34 months, three events occurred in the No_ICI group (two distant metastases and one second primary cancer), none in the Single_ICI group and one event (distant metastasis) in the Double_ICI group. Kaplan–Meier analysis estimated 3-year event-free survival (EFS) probabilities of 90.6% (95% CI, 80.6–100) for No_ICI, 100% (95% CI, 100–100) for Single_ICI and 97.9% (95% CI, 94.0–100) for Double_ICI (Supplementary Fig. 3). No deaths were observed in any treatment arm, and 3-year overall survival remained 100% across all groups. Additional prespecified secondary endpoints, including EFS and other efficacy outcomes, are not reported in this paper and will be analyzed at a later timepoint when follow-up is sufficiently mature.

### Safety

During the neoadjuvant phase, treatment-related adverse events (AEs) of grade ≥3 occurred in 29.2% of patients in the No_ICI, 64.7% in the Single_ICI and 70.8% in the Double_ICI arms (Table 2). Serious treatment-related AEs of any grade were reported in 8.3% (No_ICI), 21.6% (Single_ICI) and 20.88% (Double_ICI). No deaths occurred during the study. Discontinuation of any study treatment due to treatment-related AEs occurred in 20.8% (No_ICI), 29.4% (Single_ICI) and 37.3% (Double_ICI). Across all treatment arms, the most frequent treatment-related AEs were asthenia, alopecia, nausea, anemia and diarrhea. Immune-mediated AEs of any grade were observed in 6.3% of No_ICI patients compared with 49.0% in Single_ICI and 43.7% in Double_ICI. Grade 3/4 immune-mediated AEs were reported in 0%, 11.8% and 8.3%, respectively. The most frequently occurring immune-mediated AEs of any grade were hyperthyroidism (0%, 13.7% and 4.2%, respectively) and hypothyroidism/thyroiditis (0%, 11.8% and 8.3%, respectively). Adrenal insufficiency (0%, 3.9% and 0%, respectively) and hypophysitis/hypopituitarism (0%, 0% and 1%, respectively) were uncommon. There were no grade ≥3 AEs related to iSBRT. The most common surgery-related grade ≥3 AE was infection in 4.2% (No_ICI), 5.9% (Single_ICI) and 4.2% (Double_ICI).

**Table 2:** Neoadjuvant safety summary (ITT population, *n* = 147)

	NACT + iSBRT ‘No_ICI’ (*n* = 48)		NACT + iSBRT + durvalumab ‘Single_ICI’ (*n* = 51)		NACT + iSBRT + durvalumab + oleclumab ‘Double_ICI’ (*n* = 48)	
	Any grade, *n* (%)	Grade 3 or 4, *n* (%)	Any grade, *n* (%)	Grade 3 or 4, *n* (%)	Any grade, *n* (%)	Grade 3 or 4, *n* (%)
AE	48 (100)	17 (35.4)	51 (100)	35 (68.6)	48 (100)	36 (75.0)
TRAE	48 (100)	14 (29.2)	51 (100)	33 (64.7)	48 (100)	34 (70.8)
SAE	8 (16.7)	5 (10.4)	15 (29.4)	12 (23.5)	10(20.8)	8 (16.7)
TRSAE	4 (8.3)	4 (8.3)	11 (21.6)	9 (19.6)	10 (20.88)	8 (16.7)
AE leading to discontinuation	10 (20.8)	N/A^a^	15 (29.4)	N/A^a^	19 (37.3)	N/A^a^
TRAE						
Asthenia	27 (56.3)	0 (0)	23 (45.1)	1 (2.0)	26 (54.2)	2 (4.2)
Alopecia	29 (60.4)	0 (0)	30 (58.8)	0 (0)	25 (52.1)	0 (0)
Peripheral neuropathy	32 (66.7)	1 (2.1)	31 (60.8)	3 (5.9)	24 (50)	3 (6.3)
Nausea	28 (58.3)	1 (2.1)	32 (62.7)	0 (0)	24 (50)	0 (0)
Anemia	16 (33.3)	0 (0)	22 (43.1)	4 (7.8)	23 (47.9)	7 (14.6)
Diarrhea	16 (33.3)	0 (0)	19 (37.3)	0 (0)	20 (41.7)	1 (2.1)
Fatigue	15 (31.3)	0 (0)	19 (37.3)	4 (7.8)	18 (37.5)	5 (10.4)
Neutropenia	13 (27.1)	6 (12.5)	14 (27.5)	10 (19.6)	16 (33.3)	14 (29.2)
ALT increased	8 (16.7)	1 (2.1)	14 (27.5)	4 (7.8)	10 (20.8)	0 (0)
AST increased	4 (8.3)	1 (2.1)	10 (19.6)	1 (2)	5 (10.4)	0 (0)
IMAEs, endocrine						
Hyperthyroidism	0 (0)	0 (0)	7 (13.7)	0 (0)	2 (4.2)	0 (0)
Hypothyroidism/thyroiditis	0 (0)	0 (0)	6 (11.8)	1 (2)	4 (8.3)	0 (0)
Adrenal insufficiency	0 (0)	0 (0)	2 (3.9)	1 (2)	0 (0)	0 (0)
Diabetes mellitus	0 (0)	0 (0)	0 (0)	0 (0)	1 (2.1)	0 (0)
Hypophysitis/hypopituitarism	0 (0)	0 (0)	0 (0)	0 (0)	1 (2.1)	0 (0)
IMAEs, nonendocrine						
Infusion-related reaction	2 (4.2)	0 (0)	1 (2.0)	0 (0)	1 (2.1)	0 (0)
Ocular disorders	1 (2.1)	0 (0)	0 (0)	0 (0)	3 (6.3)	1 (2.1)
Renal dysfunction	0 (0)	0 (0)	1 (2.0)	1 (2.0)	2 (4.2)	2 (4.2)
Colitis	0 (0)	0 (0)	1 (2.0)	1 (2.0)	0 (0)	0 (0)
Interstitial lung disease	0 (0)	0 (0)	1 (2.0)	0 (0)	1 (2.1)	0 (0)
AE related to iSBRT						
Radiodermatitis	0 (0)	0 (0)	0 (0)	0 (0)	3 (6.3)	0 (0)
Asthenia	2 (4.2)	0 (0)	0 (0)	0 (0)	2 (4.2)	0 (0)
Breast pain	2 (4.2)	0 (0)	1 (2)	0 (0)	0 (0)	0 (0)
Radio pneumonitis	0 (0)	0 (0)	0 (0)	0 (0)	0 (0)	0 (0)
Breast edema	0 (0)	0 (0)	0 (0)	0 (0)	0 (0)	0 (0)
AE related to surgery						
Infection	2 (4.2)	1 (2.1)	3 (5.9)	0 (0)	2 (4.2)	1 (2.1)
Procedural pain	1 (2.1)	0 (0)	0 (0)	0 (0)	2 (4.2)	0 (0)
Wound complication	0 (0)	0 (0)	0 (0)	0 (0)	2 (4.2)	0 (0)
Lymphocele	1 (2.1)	0 (0)	2 (3.9)	0 (0)	0 (0)	0 (0)
Hematoma or erythema	1 (2.1)	0 (0)	2 (3.9)	0 (0)	0 (0)	0 (0)
Breast edema	1 (2.1)	0 (0)	0 (0)	0 (0)	0 (0)	0 (0)

Data are from the ITT population (*n* = 147). Database lock: 4 December 2025. Events reported between the first dose and surgery, except the ones related to iSBRT and surgery (30 d after surgery). The TRAEs shown are the ten most frequent in the oleclumab arm (Arm 3). Alopecia is likely to have been under-reported. IMAEs were based on a list of preferred terms intended to capture known risks of durvalumab and oleclumab, and were considered regardless of attribution to study treatment by the investigator. ALT, alanine transaminase; AST, aspartate transaminase; N/A, not available; SAE, serious adverse event; TRAE, treatment-related adverse event; TRSAE, treatment-related serious adverse event.

^a^The grade of the AE leading to treatment discontinuation was not reported by investigators; only the occurrence of a treatment discontinuation due to an AE was reported.

### Exploratory analyses and early dynamic changes

**Fig. 3:**
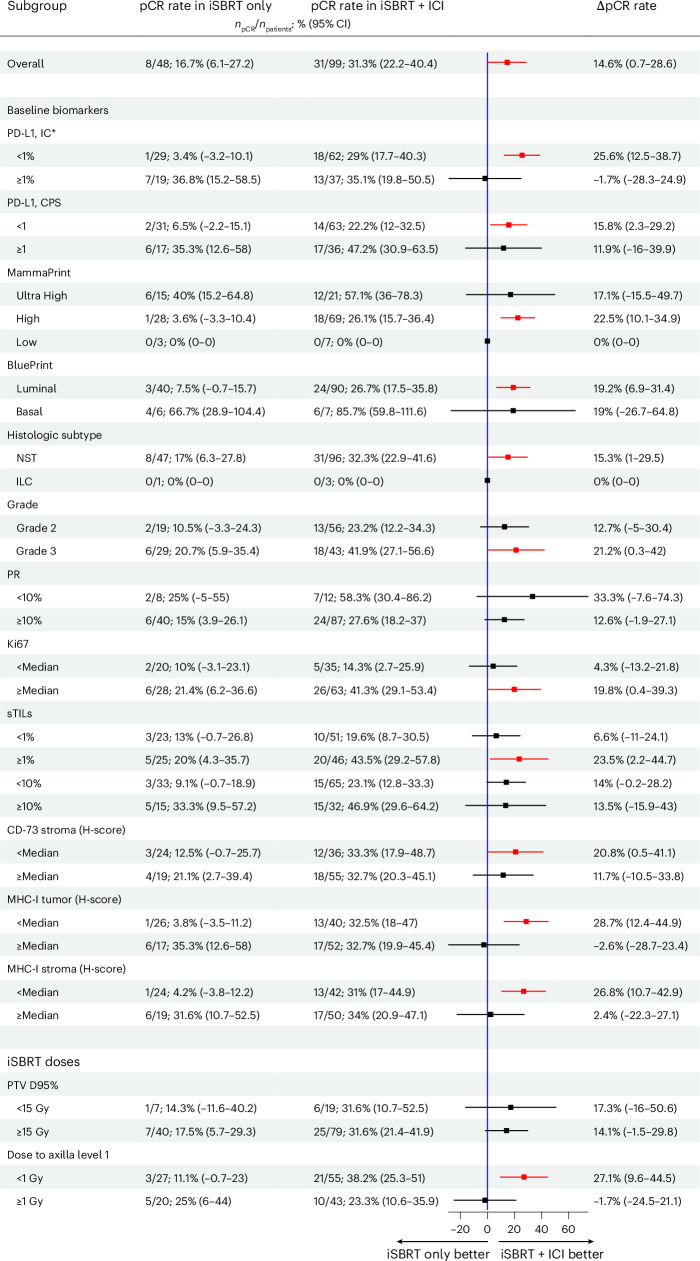
pCR rate percentage differences between iSBRT_only and iSBRT + ICI in the ITT population (*n* = 147) based on exploratory analysis of baseline biomarkers and iSBRT doses. iSBRT_only is the No_ICI arm and iSBRT + ICI is the pooled combination of the Single_ICI and Double_ICI arms. The PD-L1 IC score is defined as the percentage of the tumor area occupied by PD-L1-positive immune cells and was assessed using the VENTANA SP263 IHC assay. The CPS for PD-L1 was calculated by dividing the number of PD-L1-positive cells (tumor cells, lymphocytes, macrophages) by the total number of viable tumor cells and then multiplying by 100. The PTV is the volume that encompasses the primary BC added with 3-mm margin to account for treatment uncertainty during RT. The PTV D95% is the RT dose in Gy to 95% of the volume of the PTV. The RT dose to the axilla level 1 is the mean dose to the level 1 volume (*D*_mean_) in Gy. Points represent proportion differences; horizontal bars denote unpooled Wald 95% CIs for the difference of independent proportions. Variables with statistically significant differences in pCR rate between study arms are highlighted in red. CPS, combined positive score; PR, progesterone receptor; PTV, planning target volume. The asterisk refers to stratification factor. Source data

To isolate the benefit of adding ICI to iSBRT + NACT, we evaluated the pCR rate in the combined ICI arms (Single_ICI and Double_ICI combined as ‘iSBRT + ICI’) compared with the No_ICI arm (iSBRT_only). Overall, the addition of ICI in the ITT population led to a significant 14.6% pCR increase (95% CI, 0.7–28.6) (Fig. 3). In the PD-L1-negative subgroup, ICI addition resulted in a 25.6% pCR increase (95% CI, 12.5–38.7), suggesting a particularly pronounced benefit in this immune-cold population.

In an exploratory subgroup analysis, we investigated whether the exposure of the axilla to iSBRT influenced the response at surgery. The iSBRT targeted only the primary breast tumor while avoiding the uninvolved ipsilateral breast and the nodal regions, even in cN+ cases. Among patients who received less than 1 Gy to the axilla level 1 (*n* = 82), adding ICI to iSBRT increased pCR rate by 27.1% (95% CI, 9.6–44.5), whereas patients receiving more than 1 Gy to the axilla level 1 (*n* = 63) did not benefit from the addition of ICI (Fig. 3).

**Extended Data Fig. 3:**
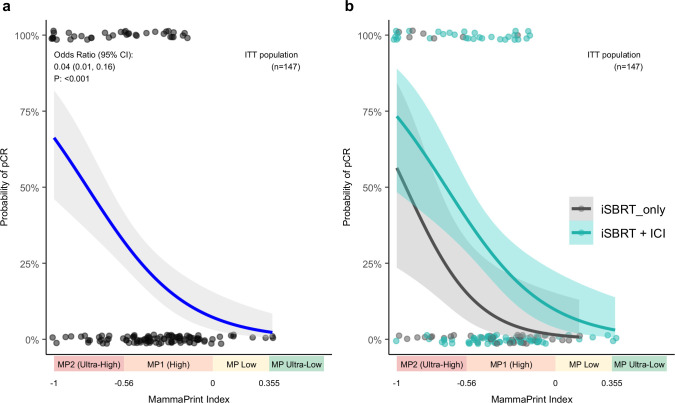
Logistic regression analysis of the correlation between the continuous MammaPrint Index and pathologic complete response (pCR) in the ITT population (n = 147). The estimated probability of pCR is derived from the logistic transformation of the fitted log-odds. The confidence band is based on the fitted log-odds±1.96 SE, transformed to probabilities of pCR. **a**, correlation assessed in the overall ITT population. Statistical significance was assessed using the Wald test. The exact p-value is 2.08×10^−5^, annotated as p < 0.001 on the plot. **b**, correlation assessed in the overall ITT population and stratified by iSBRT_alone (No_ICI arm: iSBRT + NACT) and iSBRT + ICI (Single_ICI and Double_ICI combined). *Abbreviations*: SE, standard error; CI, confidence interval; ICI, immune checkpoint inhibitor; iSBRT, immune-modulating stereotactic body radiation therapy; NACT, neo-adjuvant chemotherapy; pCR, pathological complete response. Source data

We next examined the association between baseline tumor biology and treatment response. First, we evaluated the MammaPrint index, calculated from whole-genome microarray-based gene expression profiling previous randomization. Although patients with Ultra-High MammaPrint (MP2) or higher Ki67 index experienced higher pCR rates (Supplementary Fig. 4), those with Ultra-High MammaPrint index did not derive greater benefit from adding ICI to iSBRT compared with patients with MammaPrint High Risk (MP1) index (Fig. 3 and Extended Data Fig. 3). No patient classified as MammaPrint Low Risk achieved a pCR (*n* = 10).

PD-L1 negativity and the presence of stromal TILs (sTILs) (cut-off ≥ 1%) were associated with greater benefit from adding ICI to iSBRT + NACT (Fig. 3). Notably, the baseline median sTIL percentage was 10% in patients achieving pCR, compared with 1% in patients without pCR.

**Fig. 4:**
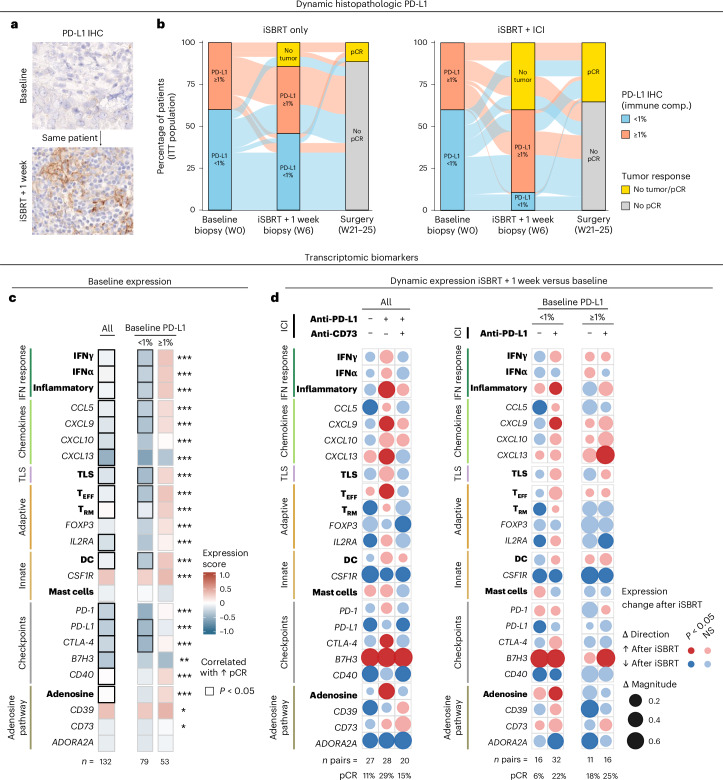
Dynamic histopathologic and transcriptomic biomarkers following iSBRT in the ITT population (*n* = 147). **a**, Example of PD-L1 IHC at baseline and at iSBRT + 1 week; samples originate from the same patient. **b**, Alluvial plot showing the dynamic changes in PD-L1 IHC (IC score) between baseline, iSBRT + 1 week (week 6) and the response at surgery. iSBRT_only (left alluvial) is the No_ICI arm, and iSBRT + ICI (right alluvial) is the pooled combination of the Single_ICI and Double_ICI arms. **c**,**d**, Transcriptomic biomarkers: genes are presented in uppercase italics, whereas signatures are indicated in bold plain text. Official gene symbols (*PDCD1*, *CD274*, *CTLA4*, *CD276*, *ENTPD1* and *NT5E*) are provided here according to approved nomenclature; aliases are used in the figure (*PD-1*, *PD-L1*, *CTLA-4*, *B7-H3*, *CD39* and *CD73*, respectively). The IFNγ response, IFNα response and inflammatory response signatures were derived from MSigDB Hallmarks (v7.0) from Liberzon et al.^41^; the TLS signature from Wang et al.^42^; the T_EFF_, DC and Mast cell signatures from CIBERSORT from Newman et al.^43^; the T_RM_ signature from Lee et al.^44^; and the adenosine pathway signature from Sidders et al.^45^. The gene composition of each transcriptomic signature is provided in Supplementary Fig. 15. **c**, Baseline transcriptomic gene and immune signatures derived from bulk RNA sequencing and compared by baseline PD-L1 IC status (<1% versus ≥1%) using the Wilcoxon rank-sum test with *P* values adjusted for multiple testing using the Benjamini–Hochberg method and reported as FDR. FDR values: *FDR < 0.05, **FDR < 0.01, ***FDR < 0.001. Associations between transcriptomic features and increased pCR were assessed using logistic regression; *P* < 0.05 denoted by a black square outline without *α* correction for multiplicity. **d**, Changes in transcriptomic features from baseline to 1 week after iSBRT were assessed in paired tumor biopsies from the same patient and calculated as the median of within-patient differences in *Z*-score-normalized signature scores (week 6 minus baseline). Transcriptomic analyses were not performed for cases without tumor present in the week 6 biopsy; these cases were therefore excluded from the paired analysis. Data are shown for all available patients across the three study arms (left), and stratified by baseline PD-L1 IC status (<1% versus ≥1%) and ICI exposure (right). Dot size reflects the magnitude of change, and color indicates the direction of change (red, increase; blue, decrease) at week 6. Saturated colors denote statistically significant changes (*P* < 0.05), whereas desaturated colors indicate nonsignificant changes. *P* values were calculated using the Wilcoxon signed-rank test. Comp, compartment; DC, dendritic cells; FDR, false discovery rate; NS, nonsignificant; T_EFF_, effector T cells; TLS, tertiary lymphoid structure; T_RM_, tissue-resident T cells; W, week. Source data

**Extended Data Fig. 4:**
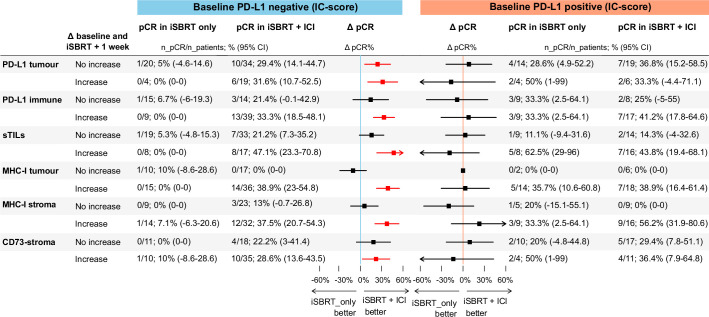
Dynamic histopathologic biomarkers following iSBRT in the ITT population. Forest plot depicting the difference in percentage of patients with a pCR according to biomarker changes between baseline and week 6. The rate of pCR is compared between the study arms that did not receive an ICI (iSBRT_only) and the study arms that did receive an ICI (iSBRT+ICI). The results are represented according to the baseline PD-L1 IC status (<1% vs ≥1%). The number of patients for each subgroup varied on the biomarker availability at baseline and week 6 because histopathologic analyses could not be performed for cases without tumour present in the week 6 biopsy; these cases were therefore excluded from the analysis. Points represent proportion differences; horizontal bars denote unpooled Wald 95% confidence intervals for the difference of independent proportions. Variables with statistically significant differences in pCR rate between study arms are highlighted in red. *Abbreviations*: CD73, cluster of differentiation 73; ICI, immune checkpoint inhibitor; IHC, immunohistochemistry; iSBRT, immune-modulating stereotactic body radiation therapy; pCR, pathological complete response; PD-L1, programmed death-ligand 1; sTILs, stromal tumour infiltrating lymphocytes. Source data

To explore the treatment-induced changes in sTILs and PD-L1 expression, these biomarkers were assessed in on-treatment biopsies obtained at week 6 (iSBRT + 1 week). PD-L1 expression was evaluated separately in immune (‘PD-L1-immune’) and tumor (‘PD-L1-tumor’) cells (Fig. 4a). The alluvial plot shows that a subset of PD-L1-negative tumors at baseline converted to PD-L1-positive at week 6, with a higher conversion rate in the iSBRT + ICI arm (55.2%) than in the iSBRT_only arm (30.0%). Notably, a substantial fraction of cases had no detectable tumor cells at week 6, and this proportion was higher in the iSBRT + ICI group than in the iSBRT_only group (Fig. 4b and Extended Data Fig. 2e). The absence of tumor cells at week 6 was significantly associated with higher pCR rates (odds ratio 4.25; 95% CI, 1.88–9.82) (Supplementary Fig. 4). No significant increase in sTIL levels was observed following treatment and the proportion of patients with sTILs ≥ 1% decreased across all treatment arms (Supplementary Fig. 5). However, an increase in sTILs at week 6 was associated with a pCR benefit among PD-L1-negative tumors treated with ICI (Extended Data Fig. 4).

With respect to PD-L1, increased expression was primarily observed on immune cells (Supplementary Fig. 6). In the iSBRT + ICI group, the increase in PD-L1-immune and PD-L1-tumor expression was associated with a higher pCR rate compared with iSBRT_only, but only among patients whose tumors were PD-L1-negative at baseline (Extended Data Fig. 4). Although PD-L1 expression in immune cells increased at week 6, this was not accompanied by a rise in TIL levels, as PD-L1 expression was rather localized on macrophages (Supplementary Fig. 7).

We then examined two immune-escape mechanisms potentially modulated by iSBRT, namely major histocompatibility complex class I (MHC-I) expression and CD73-mediated adenosine signaling. MHC-I expression on tumor cells is essential for immune recognition, and its downregulation is a well-recognized tumor immune-escape mechanism^27^. Here, we hypothesized that iSBRT + ICI would increase the levels of MHC-I and, consequently, pCR rates. Patients whose tumors exhibited low expression of MHC-I (both in tumor and stroma) benefited most from the addition of ICI to iSBRT (Fig. 3). Of interest, an increase in MHC-I expression both in tumor and stroma is significantly correlated to reaching a pCR in patients treated by iSBRT + ICI (Supplementary Fig. 8).

Considering that iSBRT may upregulate CD73 expression, we examined treatment effects on CD73 in both tumor and stromal compartments. CD73 expression, as detected by IHC, was primarily localized to the stroma. Lower baseline stromal CD73 expression was associated with better response to iSBRT + ICI (Fig. 3). At week 6, stromal CD73 decreased across all treatment arms; however, this reduction was not associated with increased pCR, whereas an increase in stromal CD73 was associated with benefit from the addition of ICI to iSBRT in the PD-L1-negative population (Extended Data Fig. 4 and Supplementary Fig. 9).

To further characterize the transcriptomic changes accompanying these treatment-induced immune dynamics, we performed bulk RNA sequencing on baseline and paired week 6 biopsies, enabling evaluation of immune-related pathways, including interferon (IFN) response, chemokines, immune checkpoints and adenosine signaling, as well as adaptive and innate cell components. At baseline, PD-L1-negative tumors (IC score < 1%) exhibited lower expression of almost all immune-related features, including IFN response signatures, chemokine expression, tertiary lymphoid structure (TLS) signature, adaptive immune cell subsets, dendritic cells and immune checkpoints, compared with PD-L1-positive tumors (Fig. 4c). These findings further support baseline PD-L1 IHC using the IC score as a biomarker for identifying immune-inflamed tumors (Fig. 4c). In addition, the adenosine signaling signature was significantly upregulated in PD-L1-positive tumors compared with PD-L1-negative tumors (Fig. 4c).

We next evaluated treatment-induced changes in immune gene expression in 75 paired biopsies in which tumor was still present at week 6 (Fig. 4d). In the Single_ICI arm, we observed increased expression of inflammatory response signatures, CXCL9, CXCL13, effector T cell markers and CTLA-4, and the adenosine signature. This arm included 28 paired samples, with eight patients achieving pCR, enabling exploratory analyses of features associated with response. In contrast, the No_ICI and Double_ICI arms included 27 and 20 paired samples, respectively, but only three pCR cases in each arm, limiting robust analyses of transcriptomic correlates of response. Nevertheless, these arms showed lower immune activation, consistent with the lower pCR rates observed in these RNA sequencing subsets. Upon treatment, modulation of the adenosine pathway was observed across the three treatment arms, with an increase in the Single_ICI arm and variable changes in the Double_ICI arm (Fig. 4d). Finally, dynamic analyses stratified by baseline PD-L1 status revealed that treatment-induced immune activation at week 6 was primarily seen in PD-L1-negative tumors treated with iSBRT + anti-PD-L1. This finding is consistent with the IHC-based observations and could suggest that iSBRT combined with ICI preferentially reprograms immunologically ‘cold’ tumors toward an inflamed phenotype.

## Discussion

Neo-CheckRay evaluates the potential immunomodulatory role of concomitant neoadjuvant iSBRT with NACT, anti-PD-L1 and an anti-CD73 in high-risk, ER^+^HER2^−^ early BC. Our findings indicate that the combination of iSBRT with systemic therapies, including ICI and chemotherapy, is associated with enhanced immune activation and favorable pathological responses.

The landmark phase III trials CheckMate 7FL and KEYNOTE-756 investigated the addition of anti-PD-(L)1 to standard NACT in this high-risk luminal B-like population, resulting in an increase in pCR rates from 13.8% to 24.5% and 15.6% to 24.3%, respectively^11,12^. In our study, the addition of anti-PD-L1 to NACT + iSBRT increased the pCR rate from 16.7% to 31.3%. iSBRT was administered in all treatment arms to maintain consistency in the local cytostatic RT effect, thereby identifying between-arm differences induced by the ICIs. Additionally, iSBRT was exclusively targeted to the primary tumor, with deliberate avoidance of the involved lymph nodes and the locoregional lymph nodes. Therefore, in patients with baseline positive lymph nodes (56.3% of the trial), nodal response after treatment reflects a systemic antitumoral effect. As the median RT dose to the involved lymph nodes was low (<1 Gy), a cytostatic RT effect on the positive lymph nodes is unlikely^20^. Furthermore, our findings indicate that when the axilla is exposed to ≥1 Gy, the benefit of adding anti-PD-L1 to iSBRT is lost. In contrast, limiting the dose to the axilla to <1 Gy is associated with a 27.9% increase in pCR when iSBRT is combined with anti-PD-L1. This finding is consistent with preclinical studies showing that the exposure of tumor-draining lymph nodes to RT diminishes the efficacy of ICI, likely due to interference of RT with immune priming^28,29^. In addition, preclinical studies suggest that the precise sequencing of RT and ICI can influence the immune response^30,31^. In our trial, iSBRT was delivered 4 weeks after the first cycle of anti-PD-L1 and immediately before the second, aiming to reduce anti-PD-L1 radio sensitizing lymphocytes right before treatment with RT^30^. However, the optimal timing of RT and ICI remains unclear and may depend on the tumor characteristics and its TME, as inflamed tumors may benefit from a distinct priming strategy compared with noninflamed tumors^32–34^. Alternative sequencing strategies are under investigation in ongoing studies, such as in the P-RAD clinical trial in early BC, where SBRT is delivered just before the initiation of the systemic neoadjuvant chemo-immunotherapy^35^.

In contrast to the phase III trials CheckMate 7FL and KEYNOTE-756, which evaluated the addition of anti-PD-(L)1 to standard NACT and reported the greatest benefit in PD-L1-positive tumors, our study revealed a different pattern of response. In Neo-CheckRay, the PD-L1-negative subgroup experienced the most pronounced benefit from the addition of anti-PD-L1 to iSBRT + NACT, with an absolute increase in pCR rate of 30.3% (95% CI, 15.7–44.9). By comparison, in the PD-L1-negative subgroups of CheckMate 7FL and KEYNOTE-756, the addition of anti-PD-(L)1 to NACT resulted in pCR increases of only 3.5% and 4.5%, respectively^11,12^. In KEYNOTE-756, PD-L1 positivity was defined as combined positive score (CPS) ≥1, whereas in CheckMate 7FL it was defined as an IC score ≥ 1%. In our trial, PD-L1-negative tumors derived the greatest benefit from adding anti-PD-L1 ICI, regardless of whether PD-L1 status was assessed using the IC score (as stratification factor) or the CPS score (in exploratory analyses). A similar observation was recently reported in metastatic non-small-cell lung cancer: Huang et al. showed that delivering 24 Gy in three fractions to a single metastasis, combined with anti-PD-L1 ICI, improved progression-free survival and enhanced systemic immune responses in PD-L1-negative (‘cold’) tumors, whereas this effect was not observed in PD-L1-positive disease^36^.

In our study, RNA sequencing analysis confirmed that PD-L1-negative tumors exhibit lower levels of immune-related gene-expression signatures, consistent with an immune-cold TME. We were able to assess the modulation of the TME through the inclusion of an early on-treatment biopsy, performed 1 week after iSBRT (week 6 of the trial, approximately 20 weeks before surgery). Paired RNA sequencing revealed early, dynamic reprogramming of the TME in PD-L1-negative tumors after iSBRT + ICI, with coordinated activation of IFN signaling, effector T cell programs and immune checkpoint pathways. In paired IHC analyses, this reprogramming was observed as an upregulation of MHC-I expression in both tumor and stromal cells, an upregulation of PD-L1 (particularly on immune cells) and a downregulation of CD73 in the stroma, suggesting a shift toward a more ‘inflamed’ TME and more ‘immunogenic’ tumors. Supporting this hypothesis, increases in PD-L1 and MHC-I expression were associated with higher pCR rates in patients treated with ICIs, particularly among those who were PD-L1-negative at baseline. Together, these observations suggest that iSBRT may modulate the TME in a way that sensitizes PD-L1-negative tumors to ICI, which may explain the higher pCR rates in this subgroup compared with the other trials investigating NACT + anti-PD-(L)1 without iSBRT.

Oleclumab, an anti-CD73 antibody, was given concomitantly with durvalumab and iSBRT to prevent the conversion of ATP (released during cellular death) into immunosuppressive adenosine. Preclinical models demonstrated that CD73 blockade acts synergistically with RT and ICI, and a phase II trial in non-small-cell lung cancer indicated progression-free-survival benefit of CD73 blockade combined with ICI after chemoradiation^22,37,38^. In our trial, however, the combination of CD73 blockade with NACT and anti-PD-L1 failed to improve outcome at surgery in comparison with anti-PD-L1 with NACT. Safety was consistent with the known safety profiles of NACT, durvalumab and oleclumab^37^. The addition of iSBRT resulted in minimal AEs and had no impact on the feasibility of breast-conserving surgery or mastectomy. Breast cosmesis is important given the novel use of iSBRT and will be evaluated during the follow-up period^26^.

We observed a high rate of tumor-free biopsies at the on-treatment timepoint (week 6), particularly in the ICI arms. The absence of tumor at this timepoint was associated with a significantly increased likelihood of achieving a pCR at surgery, supporting its potential as an early indicator of treatment efficacy. However, the lack of tumor in these early biopsies limits the ability to analyze TME modulation in patients with rapid and profound responses and introduces a potential selection bias, as only patients with residual tumor at week 6 can be included in paired analyses comparing baseline with week 6. Faster tumor clearance in the anti-CD73 arm could have led to the exclusion of the best responders, thereby enriching the paired analyses for less-responsive tumors. This may explain the lower inflammatory and effector T cell signatures observed in the Double_ICI arm compared with the Single_ICI arm. These limitations also warrant caution in the interpretation of dynamic transcriptomic differences between treatment arms. Notably, transcriptomic analyses revealed modulation of the adenosine signaling pathway in association with treatment-induced immune activation. This supports the concept that immune activation may be accompanied by the engagement of compensatory immunoregulatory pathways, highlighting a dynamic balance between immune stimulation and adaptive resistance within the TME. During the planned 5-year post-surgical follow-up, additional translational research—including blood samples at multiple timepoints in all patients and biopsies at relapse—will further elucidate the immune response triggered by iSBRT, with or without ICI.

Limitations of our trial include the small sample size, the short follow-up time at this data cut-off and the use of iSBRT in all three arms, which precludes disentangling the individual contributions of RT and concurrent systemic therapies—including paclitaxel and ICI—to TME modulation and pCR^39,40^. Consequently, the observed effects should be interpreted as reflecting the combined impact of multimodal treatments, including their mutual synergistic effects, rather than a direct causal effect of iSBRT alone. In addition, while RCB and pCR provide an early signal of activity, EFS remains the clinically decisive endpoint; therefore, longer follow-up and the conduct of future phase III trials will be essential to determine whether the increase in pCR observed with iSBRT + ICI translates to an improved EFS.

The key strengths of this randomized study include the innovative use of RT in a well-defined ER^+^HER2^−^ BC population prospectively selected with the MammaPrint genomic test and stratified by centrally assessed PD-L1 status. In addition, the availability of baseline and on-treatment tumor tissue enabled a unique evaluation of dynamic treatment-induced modulation of the TME and its association with treatment outcomes.

In conclusion, the Neo-CheckRay phase II study shows that a multimodal neoadjuvant strategy is associated with high pCR rates in ER^+^HER2^−^ BC and with dynamic modulation of the TME. The observed immune activation—particularly in PD-L1-negative tumors—is consistent with the hypothesis that multimodal approaches incorporating iSBRT and ICI are associated with the induction of an inflamed phenotype in immunologically ‘cold’ tumors.

## Methods

### Ethical approval and consent

The trial protocol was approved by the ethics committees in Belgium and in France. For Belgium: Commissie Medische Ethiek UZ Brussels/VUB, Laarbeeklaan 101, 1090 Brussels, Reference number: 2019/P/02. For France: CHU de Grenoble, Comité de Protection des personnes, CS 10217 38043, Grenoble Cedex 9, Reference number: 20-JUBO-01. All the patients provided written, informed consent before enrollment. Participants were not financially compensated for study participation. The sponsor covered all study-specific costs, including protocol-mandated consultations, examinations and procedures not part of standard care, as well as the investigational immunotherapy (durvalumab and oleclumab), which was provided free of charge. Participants therefore incurred no additional costs compared with standard treatment. Reasonable travel expenses were reimbursed upon presentation of supporting documents, up to a maximum of €75 for the entire study. All the authors attest that the trial was conducted in accordance with the protocol, its amendments and the standards of Good Clinical Practice. The protection of all clinical trial subjects was consistent with the principles of the Declaration of Helsinki. Before enrollment in the trial, all patients provided written, informed consent for the publication of anonymized and unidentifiable study results generated by the trial. No patients or members of the public were involved in the design, conduct, reporting or dissemination plans of this clinical trial. The present publication does not contain identifiable patient data or images.

### Patients

Eligibility was restricted to individuals of female sex with newly diagnosed early-stage BC, defined as: ER^+^ (≥1% of cells showing positive ER staining); HER2-negative (IHC scores of 0 or 1+, or 2+ with a negative in situ hybridization^46^); tumor stage cT2-3N0 or cT1b-3N1-3 classified according to the American Joint Committee on Cancer staging criteria (AJCC, 7th edition)^47^; primary tumor size ≥ 1.5 cm on magnetic resonance imaging; a proliferation index Ki67 ≥ 15% or histological grade 3; MammaPrint High Risk status^3^; an Eastern Cooperative Oncology Group performance-status score of 0 or 1 (ref. ^48^), and adequate organ function. Patients with a pending MammaPrint result at the end of screening could enroll if they had Ki67 > 20% and/or histological grade III; for patients aged 50 or older, node-positive disease was also required. Patients with a known MammaPrint Low Risk tumor before randomization were not allowed to enter the trial. The full eligibility criteria are provided in the clinical trial protocol, which is available as Supplementary Information.

### Trial design and treatments

The Neo-CheckRay clinical trial is a prospective, randomized, multicenter, open-label phase 2 trial (ClinicalTrials.gov identifier: NCT03875573, registered on 8 February 2019). Patients were randomized in three arms to receive iSBRT + NACT (No_ICI); iSBRT + NACT + durvalumab (Single_ICI); or iSBRT + NACT + durvalumab + oleclumab (Double_ICI). NACT consisted of once per week paclitaxel 80 mg m^−2^ intravenously for 12 administrations followed by once every 2 weeks dose-dense doxorubicin-cyclophosphamide (ddAC) intravenously (60 mg m^−2^ and 600 mg m^−2^, respectively) for four administrations (Supplementary Fig. 1). Durvalumab was 1,500 mg intravenously once every 4 weeks for four administrations and oleclumab 3,000 mg intravenously once every 2 weeks for four administrations followed by once every 4 weeks for three administrations. The use of glucocorticoids as pre/post medication was allowed. iSBRT was delivered only to the primary tumor during the end of week 4 at a dose of three fractions of 8 Gy on 3 consecutive days. It was mandatory to administer iSBRT before initiating the second cycle of durvalumab. In the pre-operative phase, RT did not target the uninvolved ipsilateral breast and the nodal regions, even in case of positive lymph nodes. Comprehensive information on iSBRT treatment planning in this trial has been reported in earlier publications^20^.

Patients were randomly assigned (in a 1:1:1 ratio) to the three arms, with stratification based on centrally assessed baseline PD-L1 using the IC score (<1% versus ≥1%). The IC score is defined as the percentage of the tumor area occupied by PD-L1-positive immune cells and was assessed using the VENTANA SP263 IHC assay. In addition, patients were stratified by lymph node involvement (positive versus negative) and primary tumor stage (cT1c-T2 versus cT3). The investigator registered the participant for enrollment using an integrated web-response system.

Patients underwent definitive surgery per local standards (breast conservation or mastectomy with sentinel lymph node biopsy and/or axillary lymph node dissection) within 2–6 weeks of completing the neoadjuvant treatment phase. Whenever indicated, patients received adjuvant RT to the breast, chest wall and lymph node regions according to local standards. A postoperative RT boost to the tumor bed was not allowed, as the pre-operative iSBRT was considered as an anticipated boost. As per protocol, durvalumab and oleclumab were not administered after surgery. Adjuvant endocrine treatment, ovarian suppression, CDK 4/6 inhibitors and PARPi were administered according to local guidelines. The clinical trial protocol of the Neo-CheckRay trial is provided in Supplementary Information.

Three safety and one futility interim analyses were performed in the study. The futility analysis was performed at 50% information rate (66 evaluable patients) to evaluate the futility of the Single_ICI and Double_ICI arms in comparison with No_ICI for achieving the primary endpoint. Based on the prespecified criteria for futility, an external independent data monitoring committee (IDMC) had recommended that all arms continue in the trial (date of the IDMC meeting: 15 September 2023). For the phase II trial, from 15 June 2021 to 11 March 2024, 200 patients were screened across seven sites in Belgium and France, of whom 147 were randomized. The database lock for all analyses was 4 December 2025.

### Endpoints

The study’s primary objective was to demonstrate an improved tumor response at surgery. The primary endpoint was RCB 0 or 1 assessed at the time of surgery, with RCB 0 corresponding to a pCR. RCB is calculated as a continuous index combining pathologic measurements of the primary tumor (size and cellularity) and lymph node metastases (number and size)^49^. Secondary endpoints included pCR (defined as ypT0/Tis, ypN0), pCR limited to the primary tumor and pCR limited to lymph nodes in the case of baseline nodal positivity. Additional prespecified secondary endpoints, including EFS, other efficacy outcomes and cosmetic results, are not reported in this paper and will be analyzed at a later timepoint once follow-up has sufficiently matured, with planned analyses at 3 and 5 years after surgery. Safety during the neoadjuvant and adjuvant phases was evaluated in all patients who received at least one dose of the study drug. Prespecified exploratory translational endpoints included associations of surgical outcome with biomarkers assessed at baseline and the on-treatment biopsy (week 6).

### Study assessments

Pathological response and RCB scores were assessed by local pathologists. AEs were monitored throughout the trial in all patients, and AEs that occurred before surgery (within 30 d after surgery for AEs related to SBRT and surgery) were analyzed. AEs were graded according to the Common Terminology Criteria for Adverse Events, version 5.0, of the National Cancer Institute^50^. Immune-mediated AEs were based on a list of preferred terms intended to capture known risks of durvalumab and oleclumab, and were considered regardless of attribution to study treatment by the investigator. Other recorded patient and disease characteristics included tumor grade, axillary nodal status, menopausal status, body mass index and age. Clinical assessments, including physical examination of the breast and the lymph nodes, were performed every 2 weeks during the treatment with paclitaxel and every 4 weeks during treatment with ddAC. Breast magnetic resonance imaging was performed at baseline and following the 12 weeks of paclitaxel before starting the ddAC regimen.

### Biomarker analyses

Core biopsies of the primary tumor were collected at baseline and on-treatment (week 6, 1 week after iSBRT) for translational research. The biomarker analysis was conducted and reported following the Reporting Recommendations for Tumor Marker Prognostic Studies (REMARK) guidelines^51^. Biomarkers (PD-L1, sTILs, CD73, MHC-I, MammaPrint and RNA sequencing) were centrally assessed on all patients. The flow diagram of translational sample inclusion by timepoint is provided in Supplementary Fig. 10. PD-L1 was centrally assessed before randomization.

IHC expression was evaluated on formalin-fixed paraffin-embedded (FFPE) full-face tissue sections (4 μm). Staining was performed using the Ventana Benchmark ULTRA instrument (Ventana Medical Systems). The following primary antibodies were used: PD-L1 (clone SP263, Ventana/Roche, cat. no. 790-4905, ready-to-use), Ki67 (clone 30-9, Ventana/Roche, cat. no. 790-4286, ready-to-use), CD73 (clone D7F9A, Cell Signaling Technology, cat. no. 13160S, dilution 1:400) and MHC-I (clone EMR8-5, Abcam, cat. no. ab70328, dilution 1:1,000). Detailed staining conditions and antibody information are provided in Supplementary Fig. 11. All antibodies were validated for IHC on FFPE tissues by the manufacturers, and staining was performed using standardized protocols on an automated platform with appropriate positive and negative controls included. Detailed information on antibody validation and standardized staining protocols is available on the respective manufacturers’ websites. Slides were scanned on the NanoZoomer S360 Digital slide scanner and visualized at high resolution using the NDPI viewer (Hamamatsu NDP.view, version 2.5). PD-L1 was prospectively and centrally assessed before randomization using the VENTANA IHC SP263 assay and scored as <1% versus ≥1% according to the IC score, defined as the percentage of the tumor area occupied by PD-L1-positive immune cells. Tumors with IC score <1% were defined as PD-L1-negative and those with IC score ≥1% as PD-L1-positive. The CPS was calculated by dividing the number of PD-L1-positive cells (tumor cells, lymphocytes, macrophages) by the total number of viable tumor cells, and then multiplying by 100.

The percentage of sTILs was quantified on hematoxylin and eosin-stained slides according to the recommendations of the International TILs Working Group^36^. CD73 and MHC-I IHC expression were quantified using a histological score (H-score), percentage of positive tumor or stromal cells multiplied by staining intensity graded from 1 to 3 (ref. ^52^). The positive control for CD73 and MHC-I IHC was human tonsil tissue, which was used for staining optimization and included with each IHC run. The negative control was the same tonsil processed without the primary antibody. Examples of PD-L1, MHC-I and CD73 staining, along with positive and negative controls, are shown in Supplementary Figs. 12–14.

Whole-transcriptome microarray-based gene-expression analysis at baseline was performed by Agendia for the assignment of the MammaPrint and the BluePrint indices. MammaPrint indices were further subdivided into High Risk 1 (MammaPrintHigh1, or MP1; from 0 to −0.56) or High Risk 2 (MammaPrintHigh2, or MP2; from −0.57 to −1.0)^53^.

### Bulk RNA sequencing

Bulk RNA sequencing was performed on all baseline samples and on week 6 samples (iSBRT + 1 week) in which tumor tissue was still present. Total RNA was extracted from frozen tissue samples using the AllPrep DNA/RNA Mini Kit (Qiagen, cat. no. 80204) for baseline samples and the RNeasy Mini Kit (Qiagen, cat. no. 74104) for week 6 samples, according to the manufacturer’s instructions. RNA concentration and purity were assessed using an Agilent 2100 Bioanalyzer (Agilent Technologies). RNA integrity for frozen samples was evaluated using the RNA integrity number. For each sample, 100 ng of total RNA was used to generate indexed complementary DNA libraries using the NEBNext Ultra II Directional RNA Library Prep Kit v2 for Illumina (New England Biolabs), following the manufacturer’s protocol. Multiplexed libraries were subsequently converted using the MGIEasy Universal Library Conversion Kit (MGI Tech) and sequenced on an MGI DNBSEQ-T7 platform using paired-end 150-base-pair reads. Reads were trimmed using Trimmomatic 0.39^54^. Trimmed reads were mapped on the reference human genome hg38 using STAR 2.7.11^55^. Genes were quantified using Salmon 1.10.0 with GENCODE release 46^56^. Gene-level counts were imported into R using tximport 1.38.2^57^ with counts derived from length-scaled TPM values. Genes with low expression (less than three samples with at least ten reads) were filtered out, and only protein-coding genes were retained based on Ensembl annotation (biomaRt 2.66)^58,59^. Expression data were normalized using DESeq2 1.50.2 size factors and variance-stabilized using the variance-stabilizing transformation^60^. For genes mapping to the same HGNC symbol, the transcript with the highest variance across samples was retained to generate a unique gene-by-sample expression matrix.

A curated repository of immune-related genes and gene-expression signatures was used for downstream analyses, and all preselected genes and gene-expression signatures are reported. The IFNγ response, IFNα response and inflammatory response signatures were derived from MSigDB Hallmarks (v7.0) from Liberzon et al.^41^; the TLS signature from Wang et al.^42^; the effector T cell, dendritic cell and mast cell signatures from CIBERSORT from Newman et al.^43^; the tissue-resident T cell signature from Lee et al.^44^; and the adenosine pathway signature from Sidders et al.^45^. The gene sets for each signature are provided in Supplementary Fig. 15.

For each sample, signature scores were computed from the corresponding gene sets using the signature weights for the tissue-resident T cell signature and GSVA 2.4.4 for the other signatures, and assembled into a signature-by-sample matrix^61^. Results were visualized using heatmaps (ComplexHeatmap 2.26)^62^. Differences in signature expression between PD-L1 strata were evaluated using the Wilcoxon rank-sum test, with *P* values adjusted for multiple testing using the Benjamini–Hochberg method^63^. Associations between baseline individual signature scores and pCR were assessed using univariate logistic regression models; signatures were considered significant at a two-sided *α* = 0.05.

Paired analyses were performed to compare baseline and on-treatment (week 6) gene and gene-signature expression within the same patients. Only patients with matched baseline and week 6 tumor samples were retained, ensuring strictly paired comparisons. For each gene or signature, within-patient changes between baseline and week 6 were assessed using the paired Wilcoxon signed-rank test. The magnitude and direction of change were summarized as the median of paired differences (week 6 minus baseline).

### Statistical analysis

Primary and secondary endpoints were assessed in the ITT population and in the predefined per-protocol population. The ITT population comprised all randomized patients, regardless of their MammaPrint status, regardless of whether the treatment was started or not, and regardless of treatment discontinuation or not. In the case that the result at surgery was unavailable (regardless of the reason for unavailability), the patient was considered as a nonresponder. The per-protocol population comprised all MammaPrint High Risk patients with an available RCB score. All patients who initiated neoadjuvant treatment were included in the per-protocol population, irrespective of whether study treatments were discontinued or not. Safety was assessed in the ITT population.

The randomization was performed by an integrated web-response system. The trial was stratified by centrally assessed PD-L1 status (IC score <1% versus ≥1%), nodal status (cN0 versus cN+) and tumor size (cT1c-T2 versus cT3). The cT1c-T2 versus cT3 stratification factor was incorporated into the randomization algorithm following a protocol amendment and was therefore not applied from the start of enrollment. Randomization was performed using the minimization method. The allocation ratio was 1:1:1, with parallel group comparisons and no crossover allowed. The randomization arm was unblinded (open-label) to allow closer monitoring of immune-related AEs in this exploratory phase II open-label trial examining a novel combination treatment. Bonferroni correction was applied for multiple comparisons of the primary endpoint; therefore, *α* = 0.025 for each comparison. The chi-squared test was performed to compare the percentages of patients with RCB 0/1 (primary endpoint) and pCR (secondary endpoint) between Single_ICI and No_ICI groups, as well as between Double_ICI and No_ICI groups. No formal comparison was foreseen between Single_ICI and Double_ICI. The sample size was estimated using EAST software by using binomial superiority two-sample design with no continuity correction, a pooled estimate of variance, a power of 80% and a two-sided *α* level of 2.5%. With a total of 132 evaluable patients, the trial had 80% power to detect a true difference (superiority) of 30 percentage points for the comparison of the rate of RCB 0/1 between No_ICI and Single_ICI, or No_ICI and Double_ICI, at a two-sided *α* level of 0.025 for each comparison, assuming a 15% RCB 0/1 rate in No_ICI. Binomial 95% CI values for RCB 0/1 and pCR were estimated using normal approximation. Futility interim analysis was scheduled at information fraction of 0.5 and the futility nonbinding boundaries were calculated using Lan–Demets beta spending functions. The study protocol also defined a (modified) ITT population including all randomized patients but excluding patients for whom an RCB result was not available and excluding MammaPrint Low Risk patients. As a sensitivity analysis, results of this population are shown in Supplementary Fig. 16.

Exploratory analyses were conducted using logistic regression to assess the association of biomarkers and other baseline characteristics with pCR. In selected exploratory analyses, the Single_ICI and Double_ICI treatment arms were grouped together as iSBRT + ICI for comparison with No_ICI (iSBRT-only).

Statistical analyses were conducted using R v4.2.2 (R Foundation for Statistical Computing). The statistical analysis plan of the Neo-CheckRay trial is provided in Supplementary Information.

### Trial oversight

This trial was an investigator-initiated academic trial sponsored by the Institut Jules Bordet and overseen by an academic steering committee of the institute. A safety run-in was conducted in six patients as a pilot study before starting the phase II randomized trial^64^. An external IDMC oversaw the safety run-in and was consulted for advice before starting the phase II trial. During the phase II trial, the IDMC assessed safety at two predefined timepoints and assessed efficacy at one prespecified interim analysis. AstraZeneca supported the trial and provided the drug supply for oleclumab and durvalumab. AstraZeneca had no role in the design of the study, in the collection, analysis and interpretation of the data, and in the writing of the paper. The MammaPrint/BluePrint testing and whole-transcriptome microarray analysis were conducted with support from Agendia. Agendia had no role in the design of the study, the collection of the samples, the interpretation of the data or the writing of the paper. The trial protocol and amendments were approved by the ethics committees in Belgium and in France. All the patients provided written, informed consent before enrollment. All the authors attest that the trial followed the protocol, its amendments and the standards of Good Clinical Practice. The protection of all clinical trial subjects was consistent with the principles of the Declaration of Helsinki.

### Inclusion and ethics statement

Eligibility criteria restricted enrollment to biological females because the trial investigated BC in its predominant patient population. Female sex was determined based on self-report. No additional demographic restrictions were applied. Participants were recruited irrespective of race, ethnicity or socioeconomic status.

### Reporting summary

Further information on research design is available in the Nature Portfolio Reporting Summary linked to this article.

## Online content

Any methods, additional references, Nature Portfolio reporting summaries, source data, extended data, supplementary information, acknowledgements, peer review information; details of author contributions and competing interests; and statements of data and code availability are available at 10.1038/s41591-026-04453-z.

## Supplementary information

Supplementary Information Supplementary Information, including all Supplementary Figures with their legends; the clinical trial protocol; and the statistical analysis plan (SAP).

Reporting Summary

Peer Review File

Supplementary Code 1 Code for all figures, including Supplementary Figures, with the exception of Fig. 4c,d.

Supplementary Code 2 Code for Fig. 4c.

Supplementary Code 3 Code for Fig. 4d.

Supplementary Data 1 Source data for all Supplementary Figures.

## Source data

Source Data Fig. 1 Statistical source data.

Source Data Fig. 2 Statistical source data.

Source Data Fig. 3 Statistical source data.

Source Data Fig. 4 Statistical source data.

Source Data Extended Data Fig. 1 Statistical source data.

Source Data Extended Data Fig. 2 Statistical source data.

Source Data Extended Data Fig. 3 Statistical source data.

Source Data Extended Data Fig. 4 Statistical source data.

## Data Availability

Data used for the analyses are available via figshare at 10.6084/m9.figshare.29489597 (ref. ^65^). The clinical trial protocol and statistical analysis plan are provided as Supplementary Information. Raw sequencing data have been deposited in the European Genome-phenome Archive (EGA) under accession number EGAD50000002552. Additional de-identified individual participant-level data generated in the Neo-CheckRay study, beyond the source data files provided with this paper, have been deposited at the Data Centre of Institut Jules Bordet. Institut Jules Bordet will honor requests for clinical trial data from qualified researchers with a clearly defined scientific objective. Sharing is also subject to the protection of patient privacy, compliance with applicable regulatory, ethical and General Data Protection Regulation (GDPR) requirements, and respect for patients’ informed consent. Data considered for sharing may include nonidentifiable patient-level and study-level clinical trial data, full clinical study reports and protocols. The expected timeframe for response to access requests is up to 6 months. Once access has been granted, the data will be available for 12 months which may be extended upon approval. No third-party data were used in this study. Source data are provided with this paper.

## Code availability

The R code used for the analyses reported in this study is available via figshare at 10.6084/m9.figshare.29489597 (ref. ^65^). The code includes all scripts used for data processing, statistical analyses and figure generation.
