# Supplementary material for: Neoadjuvant stereotactic body radiation therapy with durvalumab and oleclumab in ER+HER2− breast cancer: a randomized phase 2 trial
Source: Nat Med. 2026 Jun 25;32(7):2461–72. doi: 10.1038/s41591-026-04453-z (PMC13375581; doi:10.1038/s41591-026-04453-z)
Supplement: Supplementary file 1 — Supplementary Information, including all Supplementary Figures with their legends; the clinical trial protocol; and the statistical analysis plan (SAP). [file 41591_2026_4453_MOESM1_ESM.pdf]

nature medicine

Supplementary information https://doi.org/10.1038/s41591-026-04453-z

Neoadjuvant stereotactic body radiation therapy with durvalumab and oleclumab in $ER^{+}HER2^{-}$ breast cancer: a randomized phase 2 trial

In the format provided by the authors and unedited

Contents

RESULTS . . . 2
Patients and treatment . . . 2
Supplemental Figure 1 Trial design. . . . 2
Efficacy . . . 3
Supplemental Figure 2 Single_ICI vs. Double_ICI . . . 3
Supplemental Figure 3 Event-free survival . . . 4
Exploratory analyses and early dynamic changes . . . 5
Supplemental Figure 4 Biomarkers association with pCR, presented as odds ratios . . . 5
Supplemental Figure 5 TILs biomarker dynamics . . . 6
Supplemental Figure 6 PD-L1 biomarker dynamics . . . 7
Supplemental Figure 7 Multiplex IHC example baseline and week 6 . . . 9
Supplemental Figure 8 MHC-I biomarker dynamics . . . 10
Supplemental Figure 9 CD73 biomarker dynamics . . . 12
MATERIALS AND METHODS . . . 14
Flow diagram of translational sample inclusion (REMARK guidelines) . . . 14
Supplemental Figure 10 Flow diagram of translational sample inclusion (REMARK guidelines) 14
Immunohistochemistry . . . 15
Supplemental Figure 11 List of antibodies used for IHC . . . 15
Supplemental Figure 12 Examples of H&E, PD-L1, MHC-I and CD73 IHC at baseline and 1 week after iSBRT. . . . 16
Supplemental Figure 13 Representative baseline MHC-I IHC examples . . . 17
Supplemental Figure 14 Representative baseline CD73 IHC examples. . . . 18
RNAseq . . . 19
Supplemental Figure 15 Gene composition of transcriptomic signatures. . . . 19
Sensitivity analysis . . . 20
Supplemental Figure 16 RCB and pCR sensitivity analysis . . . 20
CLNICAL TRIAL PROTOCOL . . . 21
STATISTICAL ANALYSIS PLAN . . . 184

Supplemental Figure 1

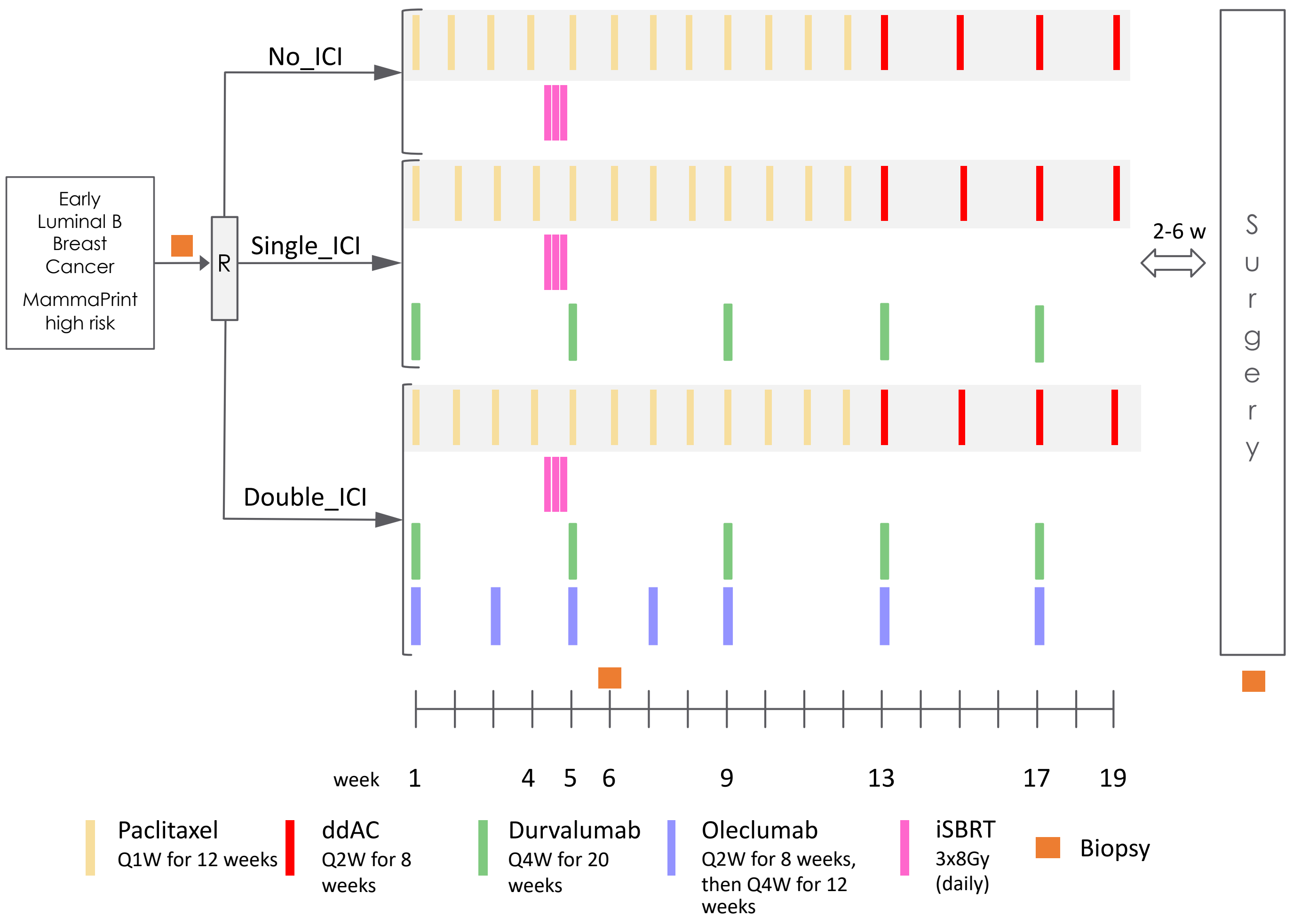

Supplemental Figure 1 | Trial design. Pre-operative systemic treatment consisted of q1w paclitaxel 80 mg/m^2 IV for 12 administrations (12 weeks) followed by q2w dose-dense doxorubicin-cyclophosphamide (ddAC) IV (60 mg/m^2 and 600 mg/m^2 respectively) for 4 administrations; the anti-PD-L1 antibody durvalumab 1500 mg IV q4w for 5 administrations and the anti-CD73 antibody oleclumab 3000 mg IV q2w for 4 administrations followed by q4w for 3 administrations. iSBRT was delivered daily immediately before the week 5 systemic treatment at a dose of 3 fractions of 8 Gy. Reproduced from *De Caluwé et al., BMC Cancer 2021*, under CC BY 4.0 licence. *Abbreviations*: ICI, immune checkpoint inhibitor; iSBRT, immune-modulating stereotactic body radiation therapy; w, weeks.

Supplemental Figure 2

Subgroup	pCR rate in Single_ICI	pCR rate in Double_ICI	Δ pCR rate
	n_pCR/n_patients; % (95% CI)		
**Overall**	**15/51; 29.4% (16.9-41.9)**	**16/48; 33.3% (20-46.7)**	**3.9% (-14.4-22.2)**
Primary tumour size			
cT1-cT2	13/36; 36.1% (20.4-51.8)	15/38; 39.5% (23.9-55)	3.4% (-18.7-25.4)
cT3	2/15; 13.3% (-3.9-30.5)	1/10; 10% (-8.6-28.6)	-3.3% (-28.7-22)
Nodal status			
cN0	6/21; 28.6% (9.2-47.9)	11/20; 55% (33.2-76.8)	26.4% (-2.7-55.6)
cN+	9/30; 30% (13.6-46.4)	5/28; 17.9% (3.7-32)	-12.1% (-33.8-9.5)
PD-L1, IC			
< 1%	9/32; 28.1% (12.5-43.7)	9/30; 30% (13.6-46.4)	1.9% (-20.7-24.5)
≥ 1%	6/19; 31.6% (10.7-52.5)	7/18; 38.9% (16.4-61.4)	7.3% (-23.4-38)
**Nodal and PD-L1 IC combination**			
cN0 and PD-L1 < 1%	5/17; 29.4% (7.8-51.1)	5/10; 50% (19-81)	20.6% (-17.2-58.4)
cN0 and PD-L1 ≥ 1%	1/4; 25% (-17.4-67.4)	6/10; 60% (29.6-90.4)	35% (-17.2-87.2)
cN+ and PD-L1 < 1%	4/15; 26.7% (4.3-49)	4/20; 20% (2.5-37.5)	-6.7% (-35.1-21.8)
cN+ and PD-L1 ≥ 1%	5/15; 33.3% (9.5-57.2)	1/8; 12.5% (-10.4-35.4)	-20.8% (-53.9-12.2)

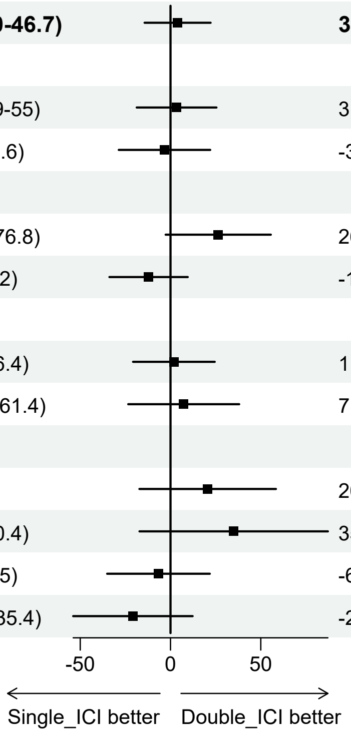

Supplemental Figure 2 | Exploratory analysis comparing the pCR rate between Single_ICI and Double_ICI in the ITT population (n=147). Forest plot showing differences in percentages of patients with a pCR between Single_ICI and Double_ICI in the ITT population, in the overall population and according to the stratification factors cT (cT1-2 vs. cT3), cN (cN0 vs. cN+) and PD-L1 (< 1% vs. ≥ 1%). Points represent proportion differences; horizontal bars denote unpooled Wald 95% confidence intervals for the difference of independent proportions. *Abbreviations*: ITT, intention-to-treat; PD-L1, programmed death-ligand 1; pCR, pathologic complete response.

Supplemental Figure 3

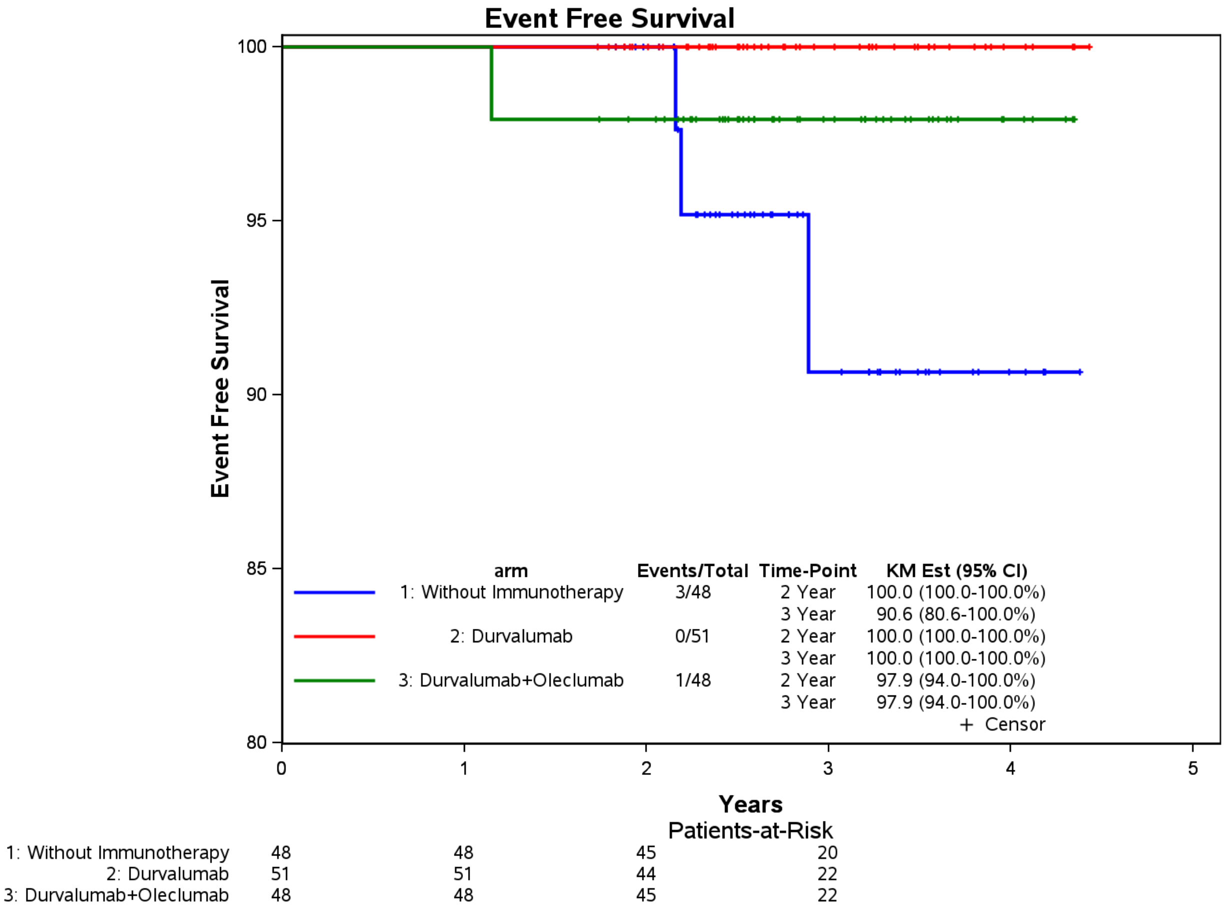

Supplemental Figure 3 | Event-free survival in the ITT population (n=147). Kaplan–Meier estimates of event-free survival (EFS) stratified by randomisation arm in the ITT population. Survival probabilities were estimated using the Kaplan–Meier method, with 95% confidence intervals calculated using Greenwood's formula and displayed as shaded areas around the curves. Tick marks denote censored observations. Numbers at risk are shown below the plot. *Abbreviations*: ITT, intention-to-treat; KM Est, Kaplan-Meier estimation.

Supplemental Figure 4

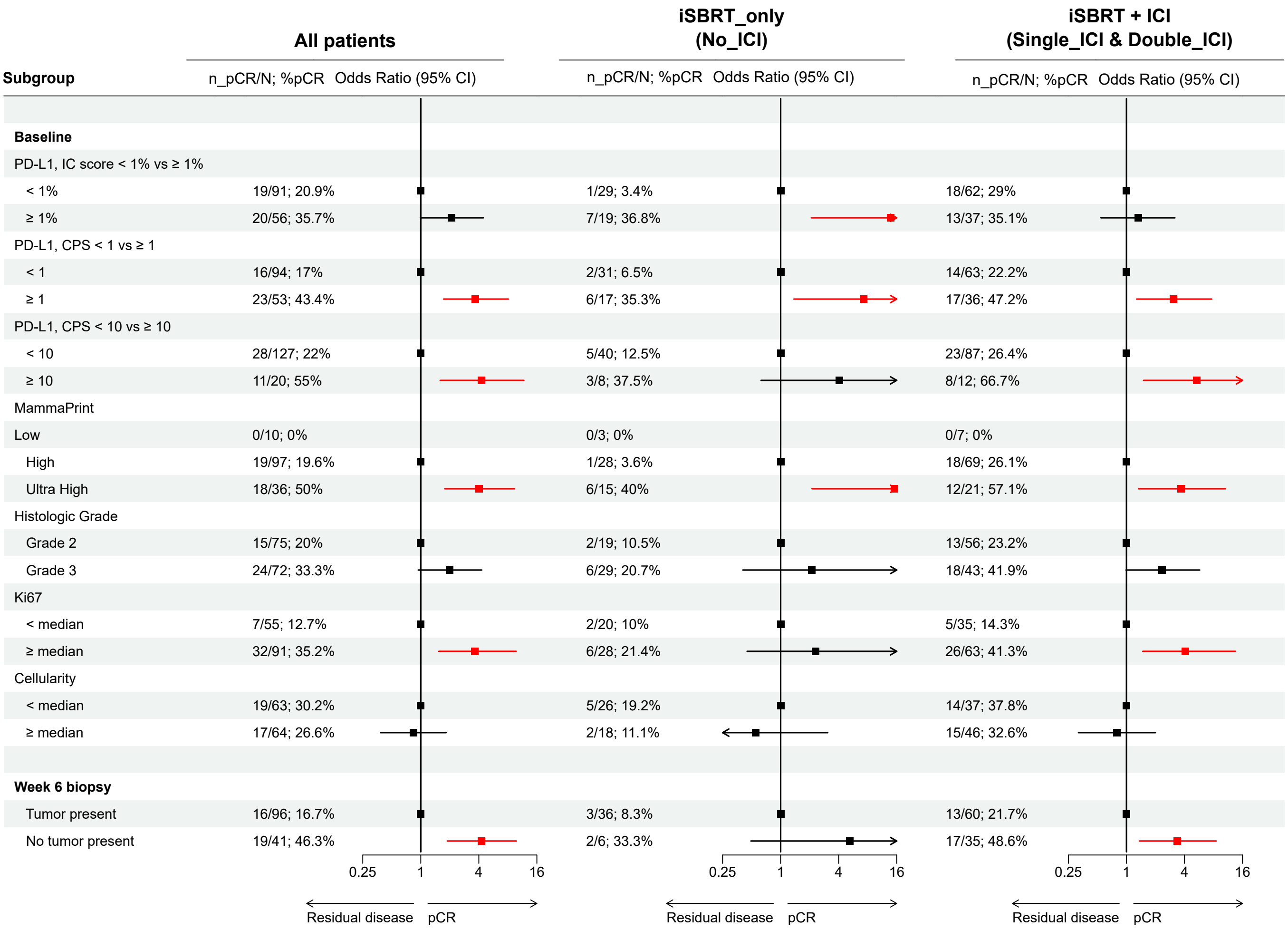

Supplemental Figure 4 | Exploratory analysis of histopathologic biomarker associations with pathological complete response (pCR), presented as odds ratios (ORs) in the ITT population (n = 147). The forest plot shows ORs for achieving pCR across predefined subgroups. Points represent the estimated OR for pCR in each subgroup relative to the reference subgroup, for which the OR was defined as 1. Horizontal bars indicate the corresponding 95% confidence intervals (CIs). ORs and 95% CIs were calculated using logistic regression models with pCR as the binary outcome and subgroup as the explanatory variable. An OR greater than 1 indicates higher odds of achieving pCR compared with the reference subgroup, whereas an OR less than 1 indicates lower odds of pCR (that is, higher odds of residual disease). Statistically significant associations ($P < 0.05$) are shown in red. The vertical dashed line at OR = 1 represents the reference subgroup and indicates no difference in the odds of pCR. *Abbreviations*: ITT, intention-to-treat; iSBRT, immune-modulating stereotactic body radiation therapy; OR, odds ratio; pCR, pathological complete response.

Supplemental Figure 5

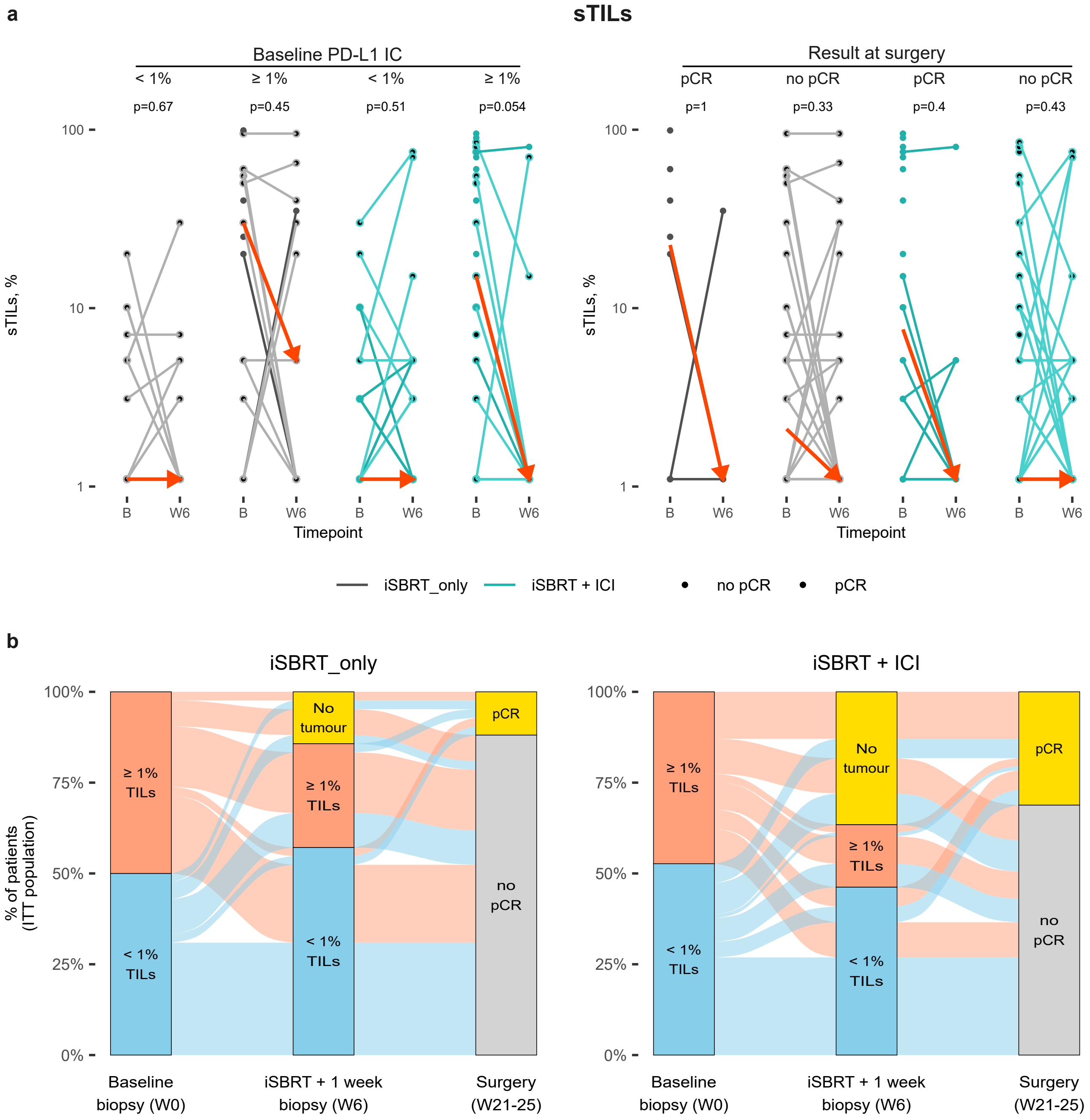

Supplemental Figure 5 | Exploratory histopathologic biomarker dynamics: TILs (ITT population, n=147). The percentage of TILs was quantified on haematoxylin and eosin-stained slides according to the recommendations of the *International TILs Working Group*. **a**, TIL dynamic between baseline and week 6 biopsy (1 week after iSBRT). The red arrow depicts the change in the median between baseline (B) and week 6 (W6). P-values were calculated using the Wilcoxon Signed-Rank Test, with paired samples originating from the same patient. Left, population is stratified by baseline PD-L1 IC score (< 1% vs. ≥ 1%. The PD-L1 IC score is defined as the percentage of the tumour area occupied by PD-L1–positive immune cells and was assessed using the VENTANA SP263 immunohistochemistry assay. Right, the population is stratified by presence of pCR at surgery or residual disease (no pCR). iSBRT without ICI is the No_ICI arm (iSBRT+NACT), and iSBRT with ICI is the Single_ICI (No_ICI+durvalumab) and Double_ICI combined (No_ICI + durvalumab + oleclumab). **b**, Alluvial plot showing TILs dynamics between baseline and week 6, and the response at surgery. *Abbreviations*: TILs, tumour infiltrating lymphocytes; ITT, intention-to-treat; pCR, pathological complete response.

Supplemental Figure 6

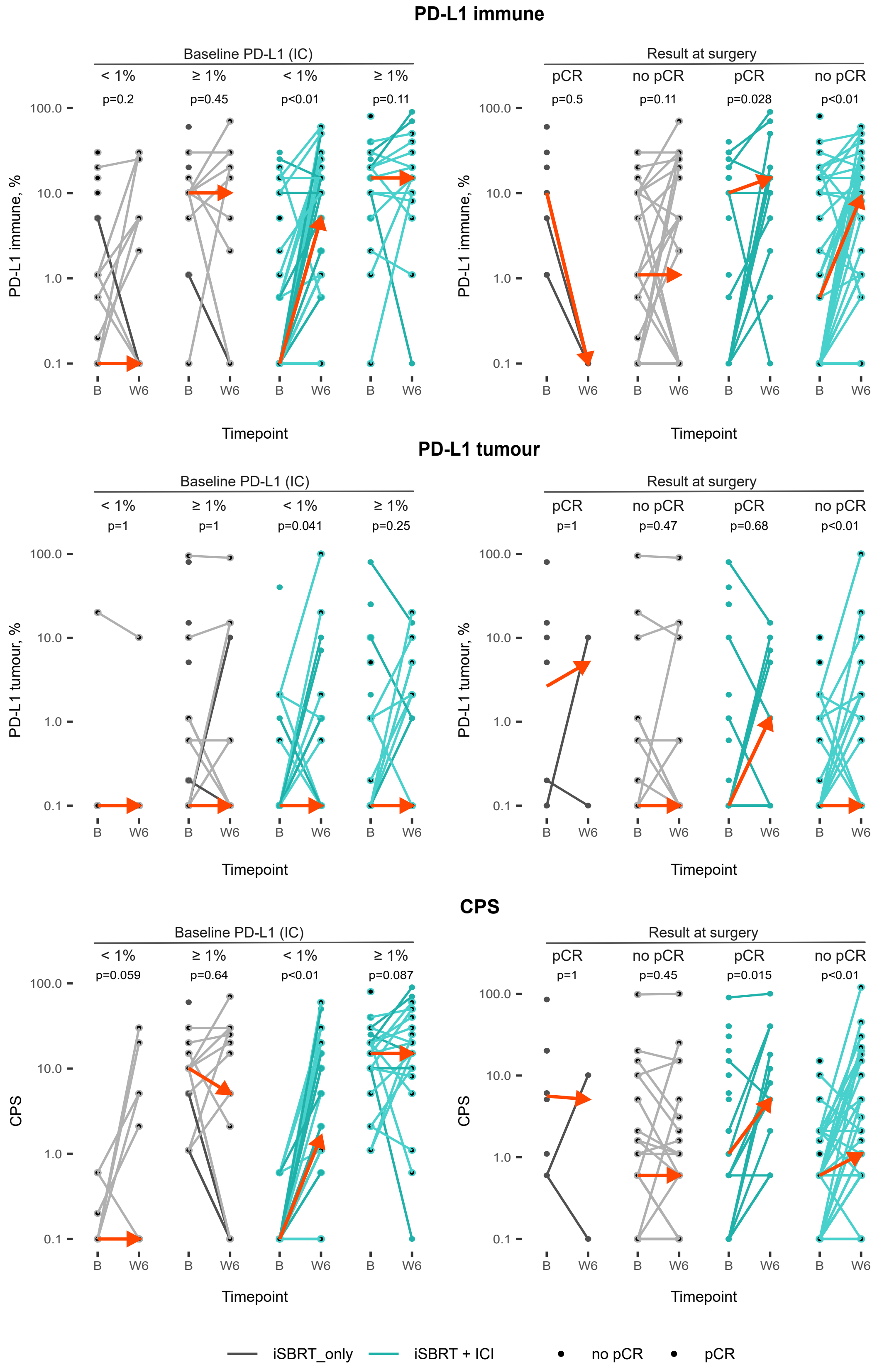

Supplemental Figure 6 | Histopathologic biomarker dynamics between baseline and week 6 (ITT population, n=147). Early histopathologic PD-L1 biomarker dynamics between baseline and week 6 biopsy (1 week after iSBRT). The red arrow depicts the change in the median between baseline (B) and week 6 (W6). P-values were calculated using the Wilcoxon Signed-Rank Test, with paired samples originating from the same patient. In the left column, the population is stratified by baseline PD-L1 IC score (< 1% vs. ≥ 1%; in the right column the population is stratified by presence of pCR at surgery or residual disease (no pCR). The PD-L1 IC score is defined as the percentage of the tumour area occupied by PD-L1–positive immune cells and was assessed using the VENTANA SP263 immunohistochemistry assay. *Abbreviations*: CI, confidence interval; CPS, combined positive score; ICI, immune checkpoint inhibitor; iSBRT, immune-modulating stereotactic body radiation therapy; NACT, neo-adjuvant chemotherapy; pCR, pathological complete response.

Supplemental Figure 7

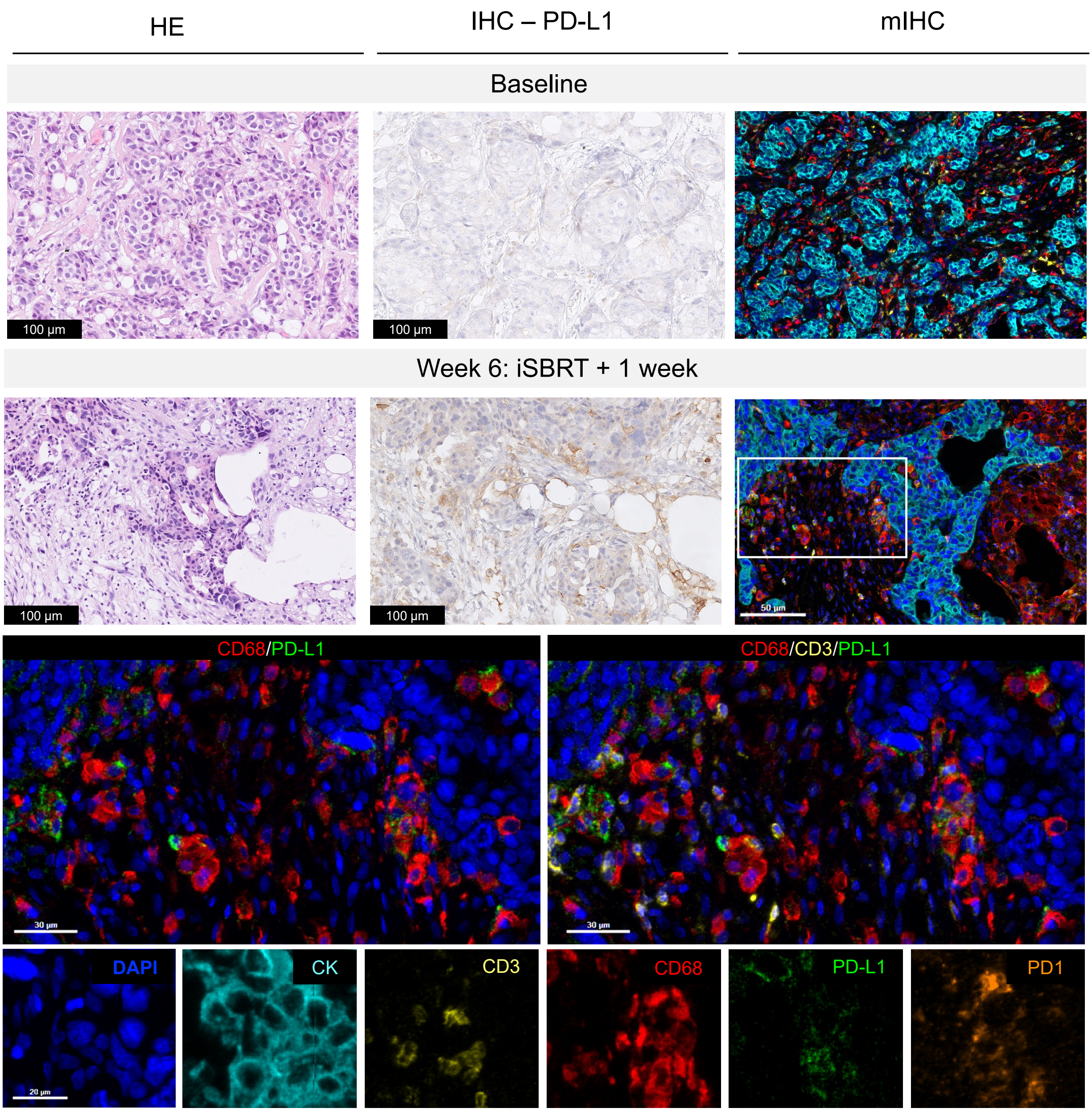

Supplemental Figure 7 | Example of multiplex IHC comparing baseline and week 6 (iSBRT + 1 week). In this example, the baseline and iSBRT + 1 week samples were obtained from the same patient. Multiplex images in the third row are magnified views of the white inset shown in the second row. The multiplex imaging demonstrates that the PD-L1 expression at week 6 is primarily localized to the CD-68 positive macrophages. *Abbreviations*: CD, cluster of differentiation; DAPI, 4′,6-diamidino-2-phenylindole; HE, haematoxylin and eosin; IHC, immunohistochemistry; iSBRT, immune-modulating stereotactic body radiation therapy; mIHC, multiplex immunohistochemistry; PD-(L)1, programmed death (ligand) 1.

Supplemental Figure 8

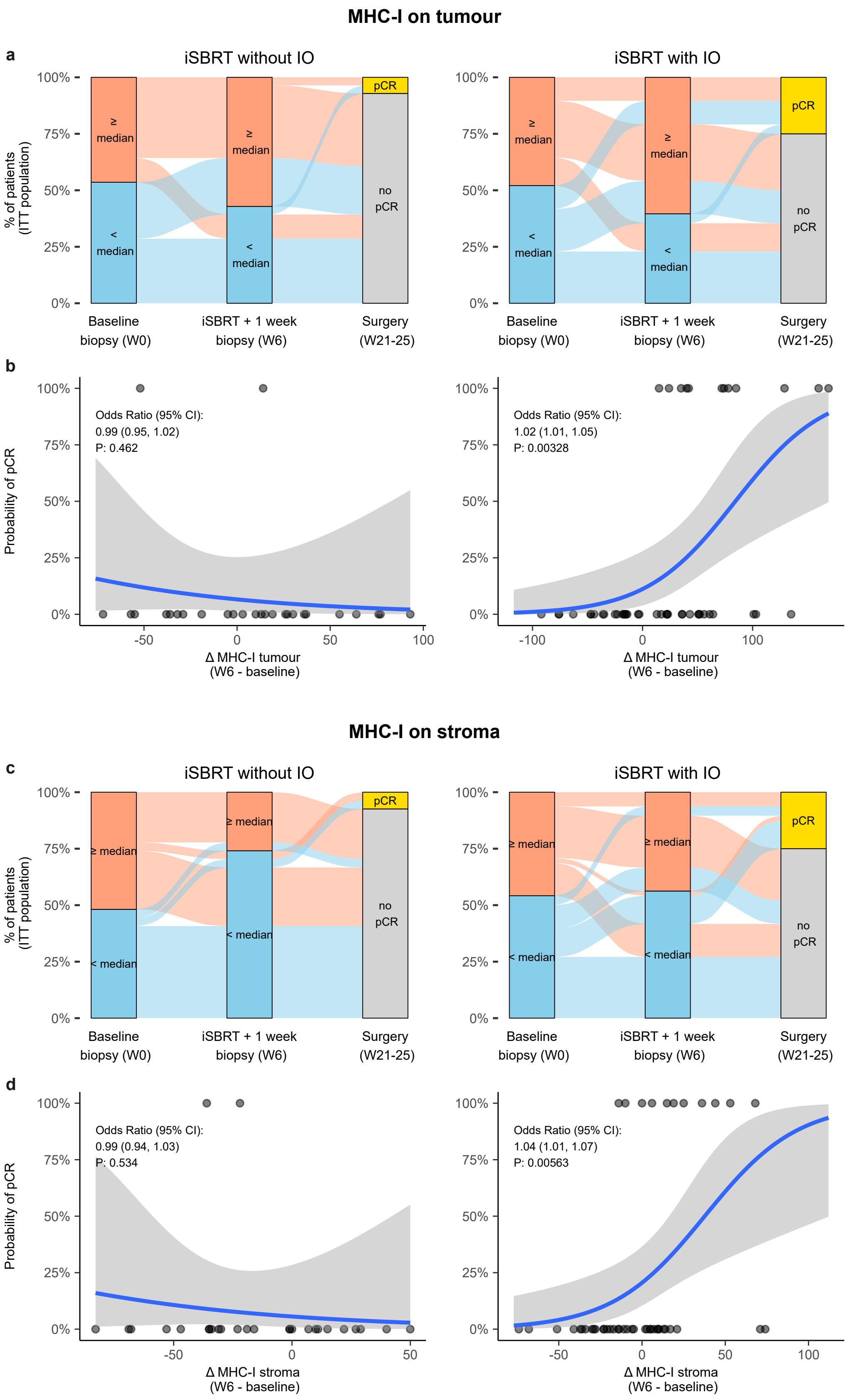

Supplemental Figure 8 | Exploratory histopathologic biomarker dynamics: MHC-I on tumour and on stroma (ITT population, n=147). a and c, Alluvial plots showing the dynamic changes in MHC-I on tumour and on stroma between baseline, iSBRT + 1 week (week 6) and at surgery. MHC-I was assessed with an H-score on the tumour and on the stroma separately. H-scores at baseline were separated in two groups: < median (< Mdn) and ≥ the median (≥ Mdn). The median value at baseline was kept to separate the H-score at week 6. **b and d**, Logistic regression analysis assessed the correlation between the H-score and pCR. The estimated probability of pCR is derived from the logistic transformation of the fitted log-odds. The confidence band is based on the fitted log-odds±1.96 SE, transformed to probabilities of pCR. Statistical significance was assessed using the Wald test. *Abbreviations*: iSBRT, immuno-modulating stereotactic radiation therapy; Mdn, median; MHC-I, major histocomptability complex I; pCR, pathologic complete response; W6, week 6.

Supplemental Figure 9

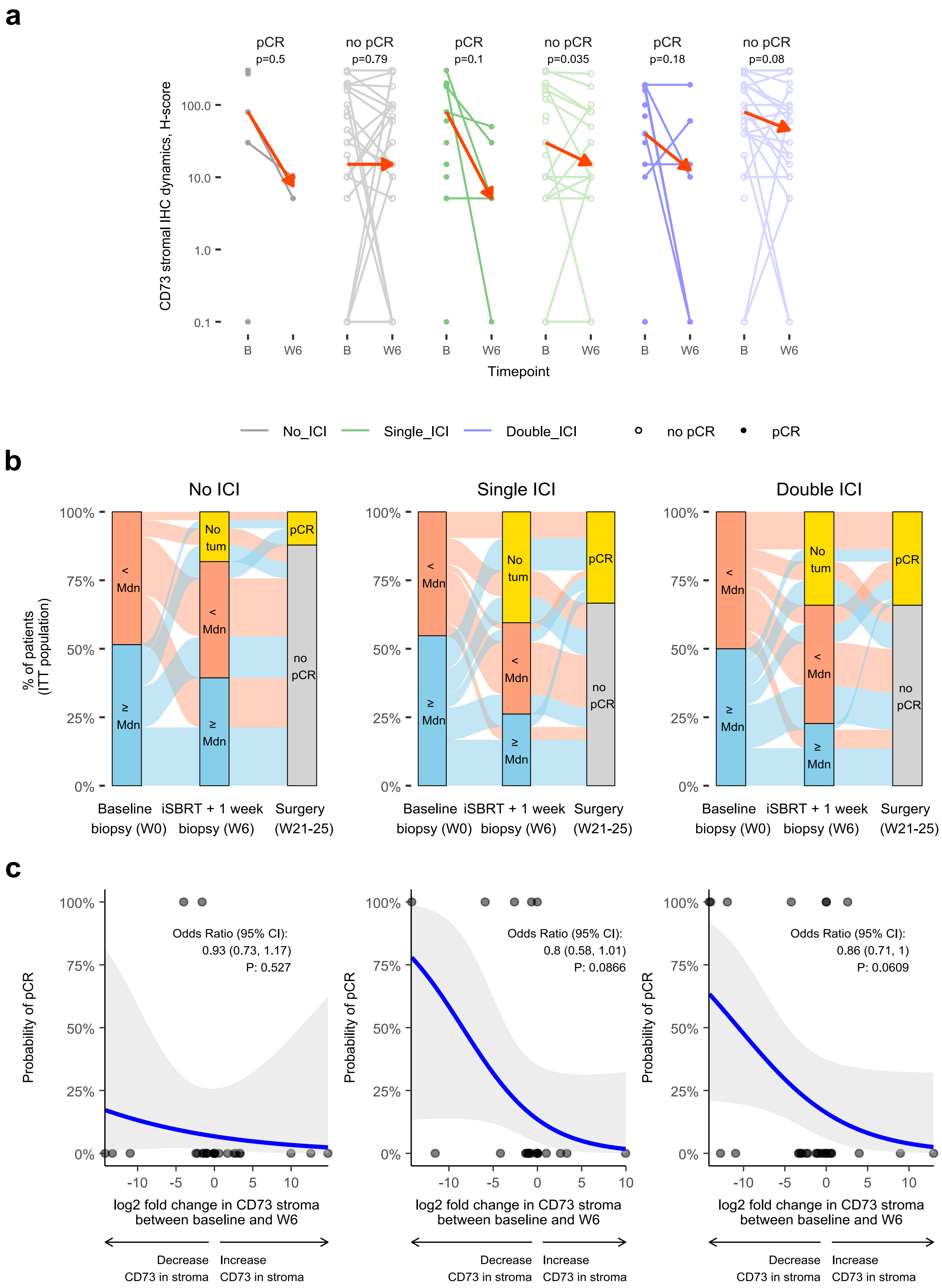

Supplemental Figure 9 | Exploratory histopathologic biomarker dynamics: CD73 (ITT population, n=147). a, Change in expression of stroma CD73 (H-score) between baseline (B) and iSBRT + 1 week (week 6, W6). The red arrow depicts the change in the median between baseline and week 6. P-values were calculated using the Wilcoxon Signed-Rank Test, with paired samples originating from the same patient. **b**, Alluvial plot showing the changes in stromal CD73 expression (H-score) between baseline, iSBRT + 1 week (week 6) and at surgery. H-scores at baseline were separated in two groups: < median (< Mdn) and ≥ the median (≥ Mdn). The median value at baseline was used to separate the H-score at week 6. **c**, Logistic regression analysis assessed the correlation between the H-score and pCR. The confidence band is based on the fitted log-odds±1.96 SE, transformed to probabilities of pCR. Statistical significance was assessed using the Wald test. *Abbreviations*: iSBRT, immuno-modulating stereotactic radiation therapy; Mdn, median; pCR, pathologic complete response; W6, week 6.

Supplemental Figure 10

Biomarkers analyses in the ITT population (n=147)

	NACT-iSBRT (“No_ICI”) **48** allocated to ITT							NACT-iSBRT-durvalumab (“Single_ICI”) **51** allocated to ITT							NACT-iSBRT-durvalumab-oleclumab (“Double_ICI”) **48** allocated to ITT						
	**TILs**	**PD-L1**	**Ki67**	**MHC-I**	**CD73**	**MP**	**RNA-seq**	**TILs**	**PD-L1**	**Ki67**	**MHC-I**	**CD73**	**MP**	**RNA-seq**	**TILs**	**PD-L1**	**Ki67**	**MHC-I**	**CD73**	**MP**	**RNA-seq**
**Pre-treatment (Baseline)**																					
No biopsy available/Not done	0	0	0	0	0	0	1	0	0	0	0	0	0	0	0	0	0	0	0	0	1
Insufficent material or no tumour on slide	0	0	0	5	5	2	0	2	0	1	5	6	2	2	0	0	0	2	2	0	0
Failed staining	0	0	0	0	0	0	-	0	0	0	0	0	0	-	0	0	0	0	0	0	-
RNA extraction failure[a]	-	-	-	-	-	-	2	-	-	-	-	-	-	1	-	-	-	-	-	-	5
Library preparation failure[b]	-	-	-	-	-	-	0	-	-	-	-	-	-	1	-	-	-	-	-	-	2
**Evaluable**	**48/48**	**48/48**	**48/48**	**43/48**	**43/48**	**46/48**	**45/48**	**49/51**	**51/51**	**50/51**	**46/51**	**45/51**	**49/51**	**47/51**	**48/48**	**48/48**	**48/48**	**46/48**	**46/48**	**48/48**	**40/48**
**On-treatment (Week 6)**																					
Biopsy unavailable or biopsy not performed	4	4	4	4	4	-	4	2	2	2	2	2	-	1	2	2	2	2	2	-	1
Representative tissue biopsy without tumour cells	8	8	8	8	8	-	14	19	19	19	19	19	-	17	16	16	16	16	16	-	19
Insufficient tissue available	0	2	3	4	6	-	0	0	2	3	3	4	-	0	0	0	0	0	0	-	0
Failed staining	0	2	1	0	0	-	-	0	0	0	0	0	-	-	0	1	1	0	0	-	-
RNA extraction failure[a]	-	-	-	-	-	-	3	-	-	-	-	-	-	3	-	-	-	-	-	-	8
Library preparation failure[b]	-	-	-	-	-	-	0	-	-	-	-	-	-	1	-	-	-	-	-	-	0
**Evaluable**	**36/48**	**32/48**	**32/48**	**32/48**	**30/48**	**-**	**27/48**	**30/51**	**28/51**	**27/51**	**27/51**	**26/51**	**-**	**29/51**[c]	**30/48**	**29/48**	**29/48**	**30/48**	**30/48**	**-**	**20/48**

Supplemental Figure 10 | Collected and analyzed tumour samples on pre-treatment (baseline) and post-treatment (week 6) for the exploratory analyses. A dash (–) indicates not applicable for the given biomarker. [a] Insufficient quantity and/or poor RNA integrity. [b] Insufficient yield or quality control failure. [c] For one patient in the Single_IO arm, baseline RNA sequencing was not evaluable, whereas the week 6 sample was evaluable. Consequently, 28 paired baseline–week 6 samples were available for the Single_IO arm, despite 29 evaluable samples at week 6. *Abbreviations:* MP, MammaPrint.

Supplemental Figure 11

Target	Clone	Isotype	Company	Catalog	Dilution	Antigen Retrieval	Incubation Conditions	Amplification	Detection Kit
**PD-L1**	SP263	Rabbit monoclonal (IgG)	Ventana Roche	790-4905	Ready to use	Tris/Borate/ EDTA buffer pH 8,4	16 min at 36°C	No	OptiView DAB
**Ki67**	30-9	Rabbit monoclonal (IgG)	Ventana Roche	790-4286	Ready to use	Tris/Borate/ EDTA buffer pH 8,5	16 min at 36°C	No	UltraView DAB
**CD73**	D7F9A	Rabbit monoclonal (IgG)	Cell Signaling	13160T	1:400	Citrate buffer pH 6	32 min at 36°C	Yes	UltraView DAB
**MHC-I**	EMR8-5	Rabbit monoclonal (IgG)	Abcam	ab70328	1:1000	Citrate buffer pH 7	32 min at 36°C	Yes	UltraView DAB

Supplemental Figure 11 | List of antibodies used for IHC in the exploratory biomarker analyses (Ventana Benchmark platform).

Supplemental Figure 12

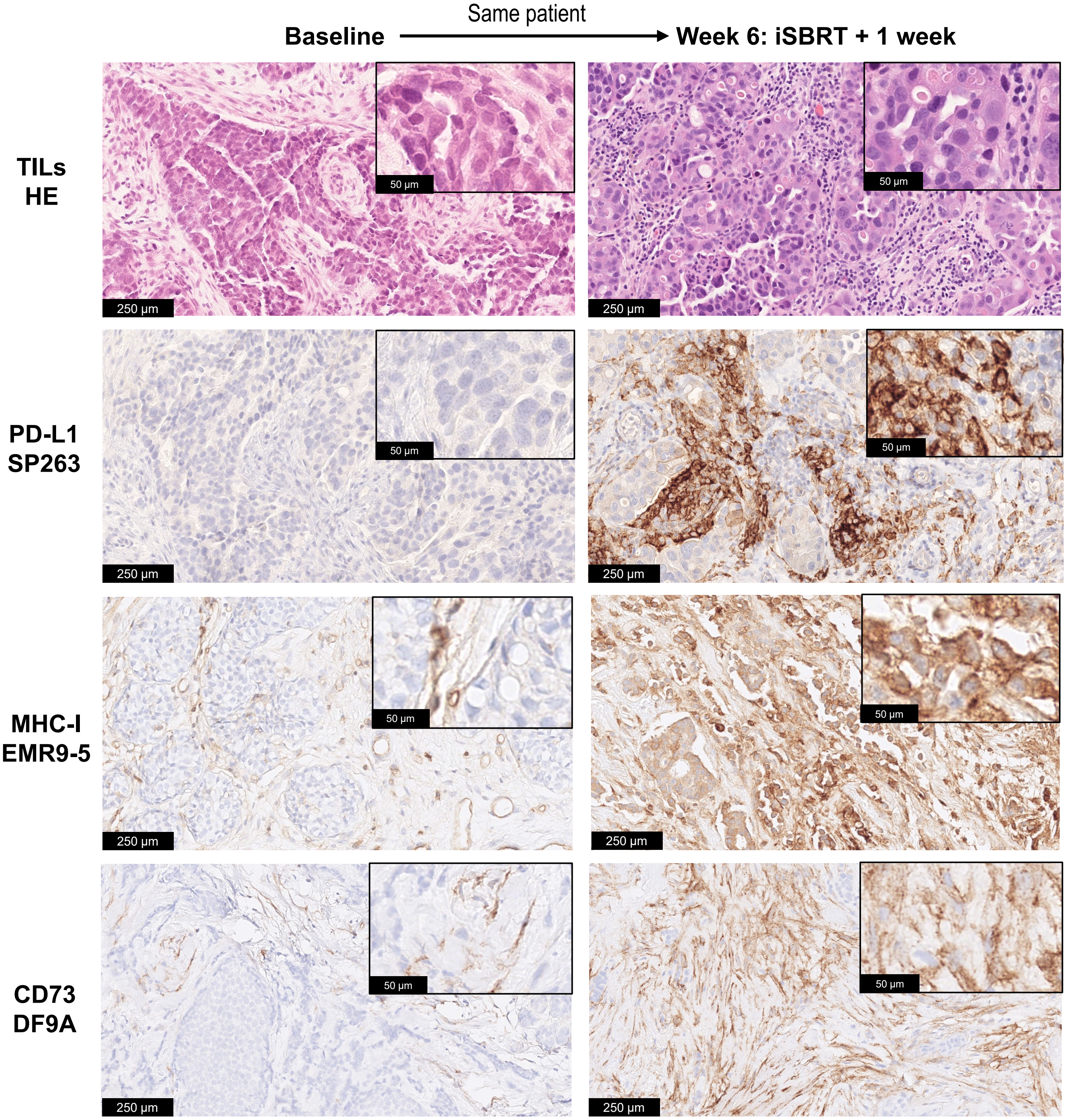

Supplemental Figure 12 | Examples of H&E, PD-L1, MHC-I and CD73 IHC at baseline and 1 week after iSBRT. Representative examples of histopathological and immunohistochemical staining in tumour biopsy specimens. All samples originate from the same patient and are shown at two timepoints: at baseline and 1 week after iSBRT. Haematoxylin and eosin (H&E) staining illustrates tumour architecture and tumour-infiltrating lymphocytes (TILs) within the stromal compartment. Programmed death-ligand 1 (PD-L1, clone SP263) immunohistochemistry highlights membranous PD-L1 expression predominantly on immune cells. Major histocompatibility complex class I (MHC-I, clone EMR8-5) staining demonstrates tumour cell surface antigen presentation. CD73 (clone D9F9A) staining shows stromal and epithelial expression of the ectonucleotidase within the tumour microenvironment. Scale bars as indicated. *Abbreviations*: H&E, haematoxylin and eosin; TILs, tumour-infiltrating lymphocytes; PD-L1, programmed death-ligand 1; MHC-I, major histocompatibility complex class I.

Supplemental Figure 13

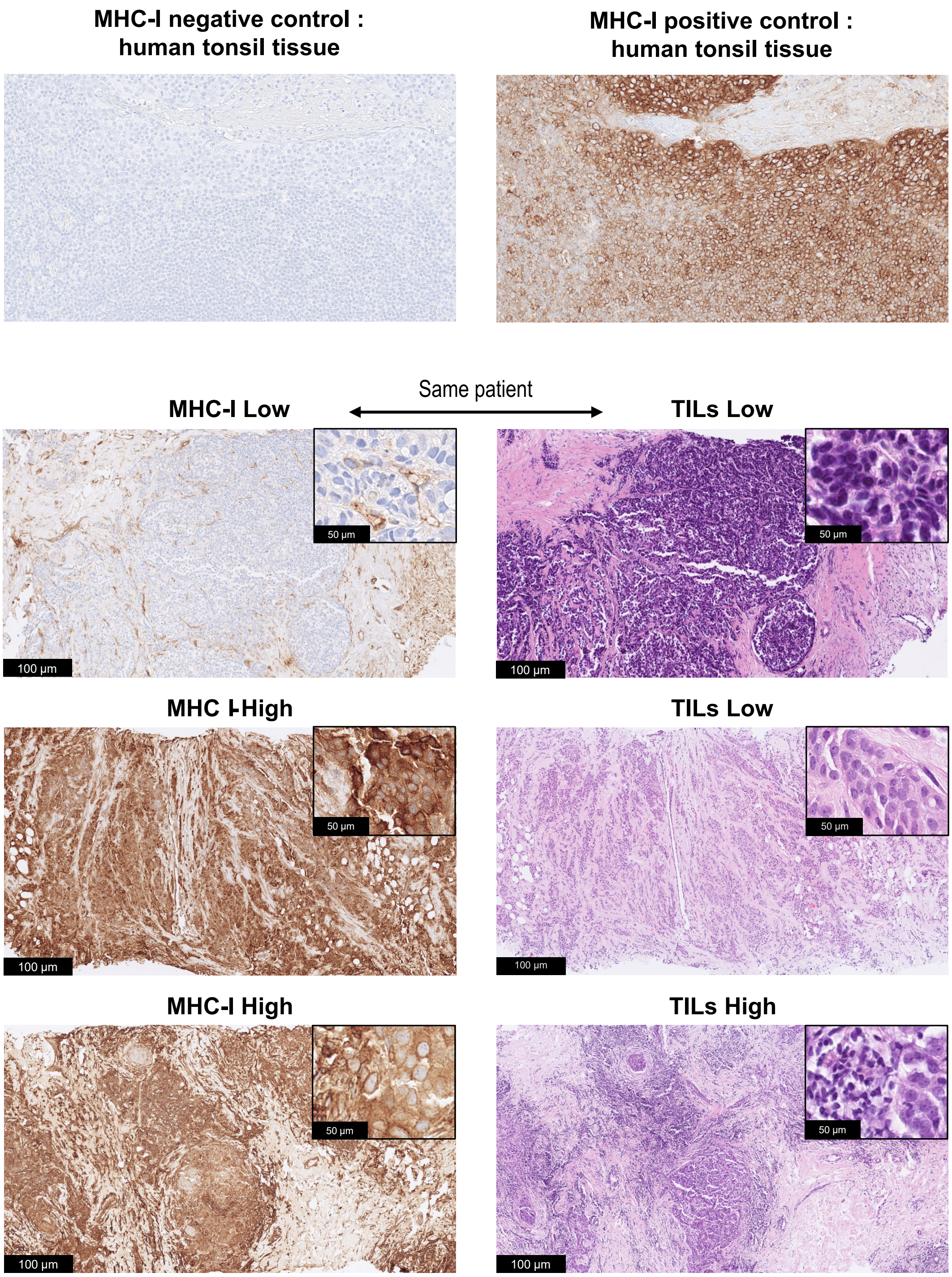

Supplemental Figure 13 | Representative baseline MHC-I IHC examples with different H-scores and the corresponding H&E. In addition, a negative and positive control on human tonsil tissue is provided. Every row originates from the same patient. MHC-I IHC expression was quantified using a histological score (H-score), defined as the % of positive tumour or stromal cells multiplied by staining intensity graded from 1 to 3. The positive control was human tonsil tissue, which was used for staining optimization and included with each IHC run. The negative control was the same tonsil processed without the primary antibody.

Supplemental Figure 14

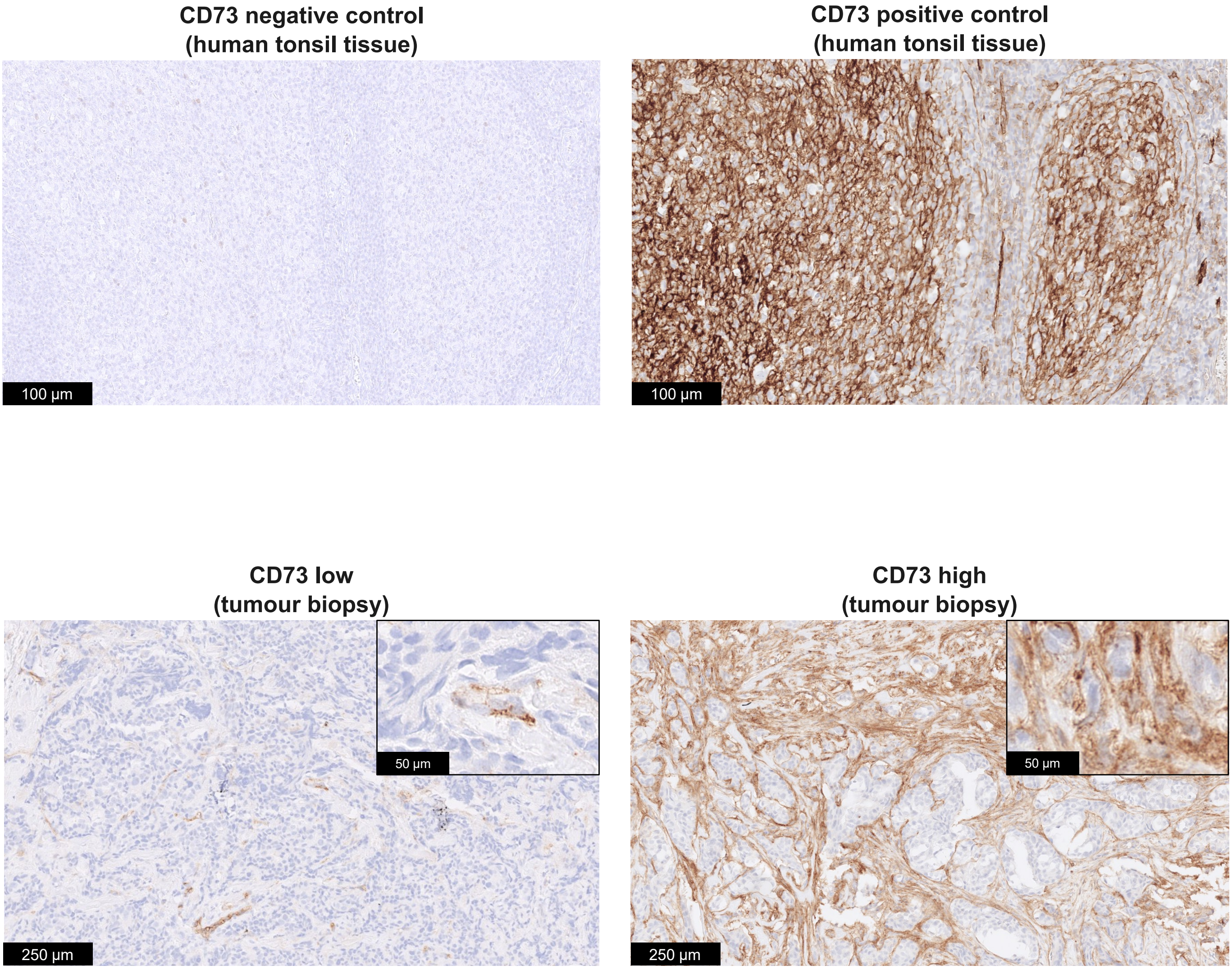

Supplemental Figure 14 | Representative baseline CD73 IHC examples. CD-73 IHC expression was evaluated in the stromal and epithelial compartments separately. MHC-I IHC expression was quantified using a histological score (H-score), defined as the % of positive tumour or stromal cells multiplied by staining intensity graded from 1 to 3. The positive control was human tonsil tissue, which was used for staining optimization and included with each IHC run. The negative control was the same tonsil processed without the primary antibody. *Abbreviations:* CD73, cluster of differentiation 73; IHC, immunohistochemistry.

Supplemental Figure 15

Signature	Reference and Genes
Interferon gamma response	**MSigDB Hallmarks v7.0 - Liberzon et al, Cell Systems 2015.** STAT1; ISG15; IFIT1; MX1; IFIT3; IFI35; IRF7; IFIT2; OAS2; TAP1; EIF2AK2; RSAD2; MX2; IRF1; OAS3; TNFSF10; IRF9; CXCL10; IFI44; BST2; XAF1; SP110; OASL; PSMB8; IFI44L; IFITM3; DDX60; LGALS3BP; GBP4; IRF8; PSMB9; PML; IFIH1; UBE2L6; IFI27; ADAR; LY6E; STAT2; CXCL9; IL10RA; PLA2G4A; TRIM21; USP18; PTGS2; EPSTI1; C1S; DDX58; IL15; NLRC5; NMI; IDO1; PSMB10; CXCL11; ITGB7; SAMHD1; HERC6; CMPK2; SAMD9L; RTP4; PTPN2; PARP14; TNFAIP2; IFITM2; PLSCR1; SOCS1; CASP1; ICAM1; WARS; PSME1; ISG20; IRF2; TRIM14; FCGR1A; MARCH1; SOCS3; JAK2; HLA-DMA; PARP12; TNFAIP6; TRIM26; VCAM1; CD274; CIITA; NAMPT; SELP; GPR18; FPR1; HELZ2; PSME2; SERPING1; CCL5; RNF31; SOD2; TRIM25; LAP3; PSMA3; RNF213; PELI1; CFB; CD86; TXNIP; HLA-DQA1; GCH1; PNP; CCL7; PTPN6; SPPL2A; IL4R; PNPT1; DHX58; BTG1; CASP8; IFI30; CCL2; FGL2; CASP7; SECTM1; IL15RA; CD40; TRAFD1; HLA-DRB1; GBP6; LCP2; HLA-G; MT2A; RIPK1; KLRK1; UPP1; PSMB2; TDRD7; HIF1A; EIF4E3; VAMP8; PFKP; CD38; ZBP1; BANK1; TOR1B; RBCK1; PDE4B; MVP; IL7; BPGM; CMTR1; AUTS2; B2M; RIPK2; CD69; MYD88; PSMA2; PIM1; NOD1; CFH; TAPBP; SLC25A28; PTPN1; TNFAIP3; SSPN; NUP93; MTHFD2; CDKN1A; IRF4; NFKB1; BATF2; HLA-B; LATS2; IRF5; SLAMF7; ISOC1; P2RY14; STAT3; NCOA3; HLA-A; IL6; GZMA; IFNAR2; CD74; RAPGEF6; CASP4; FAS; OGFR; ARL4A; SRI; LYSMD2; CSF2RB; ST3GAL5; C1R; CASP3; CMKLR1; NFKBIA; METTL7B; ST8SIA4; XCL1; IL2RB; VAMP5; IL18BP; ZNFX1; ARID5B; APOL6; STAT4.
Interferon alpha response	**MSigDB Hallmarks v7.0 - Liberzon et al, Cell Systems 2015.** MX1; ISG15; OAS1; IFIT3; IFI44; IFI35; IRF7; RSAD2; IFI44L; IFITM1; IFI27; IRF9; OASL; EIF2AK2; IFIT2; CXCL10; TAP1; SP110; DDX60; UBE2L6; USP18; PSMB8; IFIH1; BST2; LGALS3BP; ADAR; ISG20; GBP2; IRF1; PLSCR1; PSMB9; HERC6; SAMD9; CMPK2; IFITM3; RTP4; STAT2; SAMD9L; LY6E; IFITM2; HELZ2; CXCL11; TRIM21; PARP14; TRIM26; PARP12; NMI; RNF31; HLA-C; CASP1; TRIM14; TDRD7; DHX58; PARP9; PNPT1; TRIM25; PSME1; WARS; EPSTI1; UBA7; PSME2; B2M; TRIM5; C1S; LAP3; LAMP3; GBP4; NCOA7; TMEM140; CD74; GMPR; PSMA3; PROCR; IL7; IFI30; IRF2; CSF1; IL15; CNP; FAM46A; IL4R; CMTR1; CD47; LPAR6; MOV10; CASP8; TXNIP; SLC25A28; SELL; TRAFD1; BATF2; RIPK2; CCRL2; NUB1; OGFR; MVB12A; ELF1.
Inflammatory response	**MSigDB Hallmarks v7.0 - Liberzon et al, Cell Systems 2015.** CXCL10; CCL2; CCL5; FPR1; CCL20; IL1A; CXCL8; CCL7; CCL22; CXCL11; CCR7; EDN1; CD40; CXCL9; IL6; IL1B; TLR2; IL1R1; CD69; ICAM1; CCRL2; AQP9; EREG; C3AR1; GNA15; CMKLR1; PTGER4; LIF; IL15; NAMPT; OPRK1; ITGB8; PTAFR; ADM; PLAUR; NFKB1; INHBA; OSM; TNFSF10; TNFSF15; IFNGR2; ADGRE1; IL12B; CSF1; CXCL6; TNFRSF9; LYN; ACVR2A; LDLR; BDKRB1; HRH1; F3; BST2; PTGIR; CD55; CALCRL; CSF3; GPR132; IL4R; NLRP3; IL15RA; ADORA2B; GCH1; OLR1; PTGER2; CSF3R; MYC; RELA; TNFAIP6; IL7R; IL18; GABBR1; CD82; TNFSF9; NMUR1; IL2RB; TLR1; LPAR1; IRAK2; RIPK2; MMP14; P2RX7; SLC11A2; SELL; P2RY2; ABCA1; FFAR2; PROK2; GNAI3; TACR1; SLC7A1; CDKN1A; CYBB; TIMP1; HBEGF; SCARF1; EBI3; NFKBIA; SRI; SLC7A2; CCL17; TLR3; APLNR; OSMR; IL10RA; PSEN1; GPR183; ATP2B1; TNFRSF1B; BEST1; GPC3; SCN1B; ACVR1B; HPN; SEMA4D; KLF6; CD48; CXCR6; SLC1A2; GP1BA; TAPBP; RGS16; SLAMF1; LCK; HIF1A; AHR; NMI; RHOG; TPBG; NPFFR2; IFNAR1; ICOSLG; RASGRP1; IFITM1; KCNJ2; LY6E; IL18R1; IL10; KCNA3; HAS2; DCBLD2; LAMP3; VIP; CD70; RGS1; SLC31A1; ADRM1; KCNMB2; SERPINE1; MXD1; AXL; MEFV; PVR; CCL24; PDE4B; LCP2; PDPN; IRF7; MET; ATP2A2; SLC31A2; FZD5; ITGA5; SGMS2; MARCO; CD14; EIF2AK2; ROS1; ATP2C1; NDP; BTG2; MSR1; PTPRE; RNF144B; PCDH7; SPHK1; IL18RAP; RTP4; RAF1; CHST2; ITGB3; KIF1B; SELE; NOD2; C5AR1; EMP3; CLEC5A; TACR3; SLC4A4; MEP1A; SELENOS; LTA; PIK3R5; STAB1; IRF1; ICAM4; P2RX4; ABI1; CX3CL1; SLC28A2.
TLS	**Wang et al, Nature Communications 2024**. AL928768.3; ATP2A3; BLK; CCL19; CCR7; CD19; CD22; CD37; CD52; CD79A; CD79B; CXCL13; CXCR5; FCMR; FCRLA; IKZF3; IL16; LINC00926; LTB; MS4A1; NIBAN3; POU2AF1; RAC2; RASGRP2; RIPOR2; SELL; TCF7; TCL1A; TNFRSF13C; VPREB3.
Teff	**CIBERSORT - Newman et al, Nat Methods, 2015.** CD8A; EOMES; PRF1; IFNG; CD274.
Trm	**Lee et al, Science Immunology 2022.** NBL1; RP4-728D4.2; LMO4; VCAM1; MLLT11; SEMA4A; CD244; FASLG; TNFSF4; C1orf21; RGS13; NR5A2; SNAP47; GALNT2; LYST; AC092580.4; FAM49A; FAM179A; CRIM1; GNLY; CD8A; CD8B; GPAT2; NR4A2; GPD2; CERS6; TTN; CCDC141; ITM2C; UBE2F; PDCD1; SRGAP3; RP11-222K16.2; EOMES; CMC1; CCR1; CCR5; ABHD6; DZIP3; CD200R1; GTPBP8; HEG1; GOLIM4; GNB4; CD38; DTHD1; STAP1; TNIP3; TMEM155; ITGA1; ITGA2; GZMA; PLPP1; RASA1; PDLIM4; TIMD4; HAVCR2; DBN1; GFOD1; AIF1; HLA-DRA; HLA-DRB5; HLA-DRB1; HLA-DQA1; HLA-DQB1; SOBP; VNN2; CHST12; ETV1; CHN2; WIPF3; TRGV9; TRG-AS1; LIMK1; PON3; PON2; LRRN3; FAM3C; ADAM28; TOX; ASPH; MSC; FABP5; MTSS1; PIP5K1B; RP11-305L7.1; RP11-305L7.3; SLC2A8; RP11-492E3.2; SLC2A6; DBH-AS1; CLIC3; PNPLA7; PRR5L; PLA2G16; CTSW; TPCN2; MYO7A; PRSS23; DIXDC1; FXYD2; CRTAM; BARX2; NEBL; SPAG6; PRF1; ENTPD1; PIK3AP1; AFAP1L2; CD9; LPAR5; PTMS; LAG3; CLECL1; CLEC2B; KLRD1; RP11-277P12.20; KLRK1; KLRC4; KLRC2; KLRC1; RP11-291B21.2; NELL2; KRT86; KRT81; ITGB7; CD63; IFNG; TMTC2; HVCN1; KATNAL1; SERP2; TSC22D1; SPRY2; KDELC1; GZMH; GZMB; AKAP5; FUT8; DPF3; ATP8B4; DAPK2; PIF1; SEMA7A; MIR9-3HG; MCTP2; LRRC28; GTF3C1; NOD2; CPNE2; ADGRG5; ADGRG1; GSG2; RASD1; AC069363.1; CCL3; CCL4; CCL3L3; CCL4L2; LINC00672; RP11-357H14.17; GNGT2; ABI3; SCPEP1; PECAM1; FBF1; GALNT1; BCAS4; ZFP82; LINC01480; ATP1A3; NKG7; KIR2DL4; DGCR6; KIAA1671; HMOX1; APOBEC3H; LINC00158; MIR155HG.
DC	**CIBERSORT - Newman et al, Nat Methods, 2015**. CCL13; CD209; HSD11B1.
Mast cells	**CIBERSORT - Newman et al, Nat Methods, 2015.** CPA3; HDC; MS4A2; TPSAB1; TPSB2.
Adenosine	**Sidders et al, Clinical Cancer Research 2020.** PPARG; CYBB; COL3A1; FOXP3; LAG3; APP; CD81; GPI; PTGS2; CASP1; FOS; MAPK1; MAPK3; CREB1.

Supplemental Figure 15 \| Gene composition of transcriptomic signatures. Gene composition of transcriptomic signatures presented in Fig 4d-e in the main manuscript. Each signature is listed with its corresponding gene set and reference. *Abbreviations:* TLS, tertiary lymphoid structures; Trm, tissue-resident memory T cells; Teff, effector T cells; DC, dendritic cells.

Supplemental Figure 16

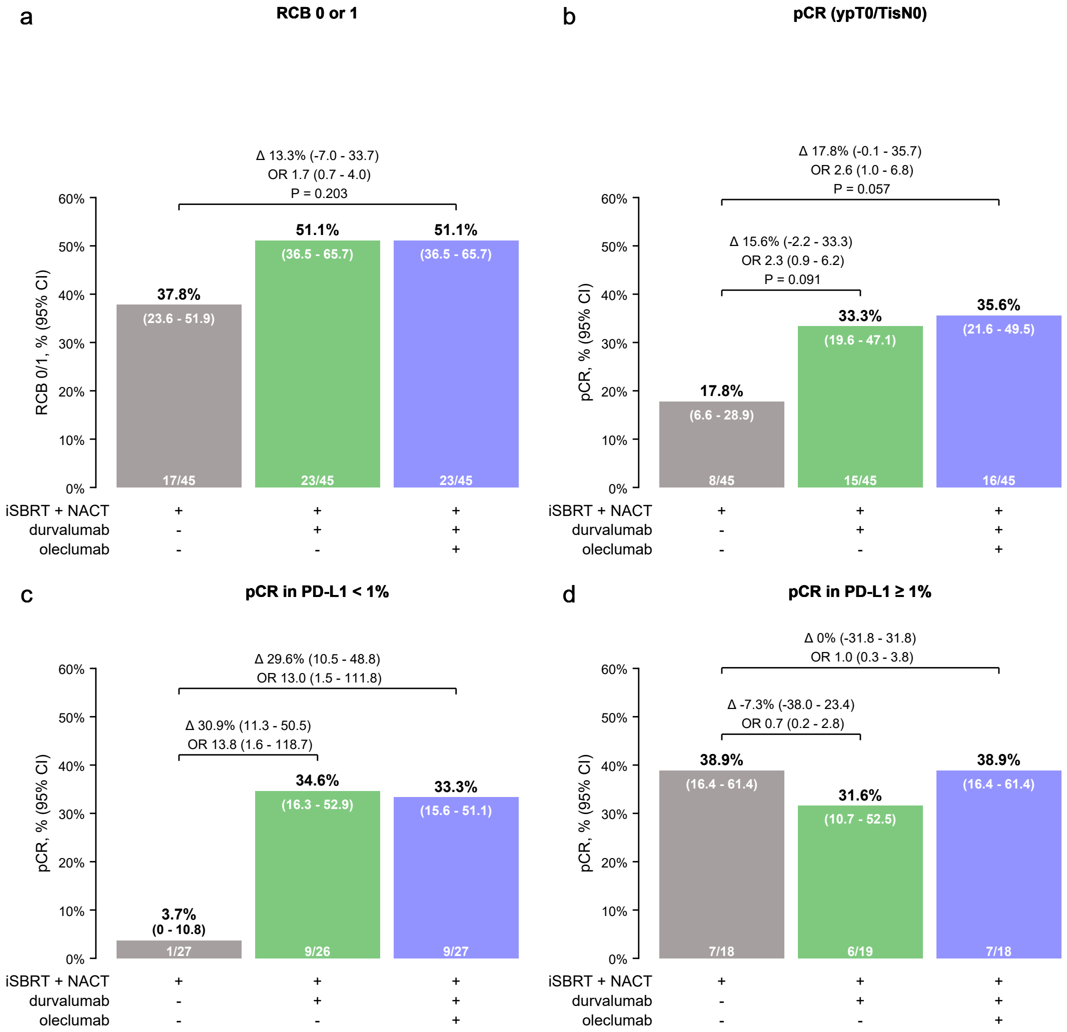

Supplemental Figure 16 | RCB and pCR in the population excluding MammaPrint low risk patients and excluding patients without result at surgery (sensitivity analysis, n=135). This population includes MammaPrint status Unknown and MammaPrint High Risk, however excludes patients without known result at surgery. This result is provided as a sensitivity analysis. The proportion difference is presented with unpooled Wald 95% confidence intervals assuming independent proportions. Odds ratios and their Wald 95% confidence intervals were estimated from logistic regression. The p-values are based on Chi-Square tests. **a**, bar plots depicting RCB 0/1, and **b**, pCR rates in the overall and by subgroups defined by **c**, PD-L1 negative and **d**, PD-L1- positive. *Abbreviations*: iSBRT, immune-modulating stereotactic body radiation therapy; pCR, pathological complete response; PD-L1, programmed death-ligand 1; RCB, residual cancer burden.

EU Number: 2024-511849-19-00
Sponsor Protocol Number: IJB-LBC-NEOCHECKRAY-2018
ClinicalTrials.gov Number: NCT03875573

Neo-adjuvant chemotherapy combined with Stereotactic Body Radiotherapy to the primary tumour +/- durvalumab (MEDI4736), +/- oleclumab (MEDI9447) in luminal B breast cancer: a phase II randomised trial
Neo-CheckRay

Study Chair

Alex De Caluwé, MD – Institut Jules Bordet

Co-Study Chair

Emanuela Romano, MD PhD– Institut Curie

Sponsor:

Institut Jules Bordet

Rue Meylemeersch 90 1, 1070 Bruxelles/Brussel
Belgique/België

ctsu.neocheckray@hubruxelles.be

Protocol version 8.0

31/01/2025

PROTOCOL WRITING COMMITTEE

Institut Jules Bordet	Institut Curie	University Hospital of Antwerp
Alex De Caluwé, MD<br>(Radiation Oncologist)	Emanuela Romano, MD PhD<br>(Medical Oncologist)	Philip Poortmans MD PhD<br>(Radiation Oncologist)
Laurence Buisseret, MD PhD<br>(Medical Oncologist)		
Roberto Salgado, MD PhD<br>(Pathologist)		
Michail Ignatiadis, MD PhD<br>(Medical Oncologist)		
Martine Piccart, MD PhD<br>(Medical Oncologist)		
Marianne Paesmanns, Msc<br>(Study Statistician)		
Clinical Trials Support Unit<br>(Operational Support)		

PROTOCOL APPROVAL – Signature Page

EU Number: *2024-511849-19-00* **Sponsor Protocol Number:** IJB-LBC-NEOCHECKRAY-2018

ClinicalTrials.gov Number: NCT03875573

The signature below constitutes the approval of this protocol and the attachments, and provides the necessary assurances that this study will be conducted according to all stipulations of the protocol, including all statements regarding confidentiality, and according to national, local legal and regulatory requirements and applicable European regulations and ICH GCP guideline.

Sponsor

Title and Name	Approval date	Signature
Michail Ignatiadis MD, PhD	24-Feb-25 | 3:31 PM CET	DocuSigned by: 7D15F40D993A43A...

Study Chair

Title and Name	Approval date	Signature
Alex De Caluwé, MD	24-Feb-25 | 3:16 PM CET	DocuSigned by: Alex de Caluwé BAB84CD960EA4F6...

Co-Study Chair and National Coordinator in France

Title and Name	Approval date	Signature
Emanuela Romano, MD PhD		

National Coordinator in Belgium

Title and Name	Function	Approval date	Signature
Laurence Buisseret, MD PhD	National Coordinator	25-Feb-25 | 4:56 PM C T	DocuSigned by: Laurence Buisseret 4EED53575AE94A6...

Site Principal Investigator

Title and Name	Function	Approval date	Signature
	Site Principal Investigator		

The name and function of the representatives of the sponsor authorised to sign the protocol or any substantial modification to the protocol are :

- Doctor Martine Piccart, Scientific Director, Institut Jules Bordet
- Doctor Michail Ignatiadis, Leader of the Academic Promoting Team, Institut Jules Bordet

The contact details of the Study Chair are:
Alex De Caluwé
Phone: +32 2541 3485
e-mail: *alex.decaluwe@hubruxelles.be*

DOCUMENT HISTORY

Version	Protocol version release date	Short description of modifications	Country(ies) if applicable	Submission as substantial amendment
1.0		This document is based on the CTSU protocol template v2.	Belgium<br>France	☐ Yes<br>☒ No
1.1	24/05/2019	Modifications in response to the request for additional information in Belgium (INI-0022):<br>• IDMC added for the Phase II<br>• Section added about the combination of radiation therapy and docetaxel, including radiation dermatitis and radiation recall phenomenom<br>• Wording clarification.	Belgium<br>France	☐ Yes<br>☐ No<br>☒ Not applicable
2.0	14/10/2019	• Clarification of the breast appearance assessment (synopsis, sections 2.2.3 and 8.4)<br>• Rectification or modification of inclusion criteria n°5,6,10,11,15,17 and 19 (synopsis, sections 4.1 and 8.1)<br>• Rectification or modification of exclusion criteria n°7,13,16,17,21 and 23 (synopsis, sections 4.2)<br>• Procedure timeline rectifications (section 2.2.3, figure 2)<br>• Modification of immune related toxicity of special interest (section 3.2.1.4)<br>• Precision on SRT treatment preparation (section 6.8.1.1)<br>• Modification of general management of study drug delays (table 5)<br>• Precision on HCV serology (section 8.1)<br>• Correction of laboratory tests (section 8.2.2)<br>• Procedure timelines clarifictions (section 8.4)<br>• Reduction of the number of time points for the PBMCs blood	Belgium<br><br>France	☒ Yes<br>☐ No<br><br>☐ Yes<br>☒ No

		sample (section 11.1.2 and table 11.3)<br>• Wording clarifications		
2.1	21/11/2019	Modifications in response to the request for additional information in Belgium (AMD-0075):<br>• Modification of the breast appearance assessment (synopsis and section 2.2.3)<br>• Modification of immune related toxicity of special interest (section 3.2.1.4)<br>• Editorial corrections in the inclusion criteria (section 4.1)<br>• Correction of the delay between subject's enrolment/randomisation and first study drug administration (section 4.3)	Belgium<br>France	☐ Yes<br>☐ No<br>☒ Not applicable
3.0	02/12/2019	• Modification of the SBRT administration timelines (sections 1.2.3.2.2, 3.2.3.1, 6.8.1.1, 8.2.1, 8.5: schedule of assessment legend)<br>• Addition of new toxicity information following durvalumab IB v14 (section 1.3.1.2)<br>• Addition of a breast cosmetic evaluation at screening (sections 8.1 and 8.5: schedule of assessment)<br>• Clarification of the amylase/lipase testing in the schedule of assessment legend (section 8.5)<br>• Correction of inconsistencies in the numbering between the schedule of assessment and its legend (section 8.5)<br>• Clarification of tissue sample collection: addition of new sections 11.1.1.1.1 to 11.1.1.1.7 and modification of sections 11.1.1.2.1 and 11.3<br>• Addition of the definition of an evaluable subject (section 12.1.1)<br>• Clarification of IDMC involvment in phase II (section 20.1)	Belgium<br><br>France	☒ Yes<br>☐ No<br><br>☐ Yes<br>☒ No (Initial submission)

		• Replacement of “patient” by “subject” throughout the text<br>• Wording clarification		
3.1	21/01/2020	Modification in response to the request for additional information in France (INI-0023)<br>• Addition of safety data on the combination of oleclumab/radiotherapy (section 1.3.1.2)<br>• Addition of safety data from the phase I of the SYNERGY trial (section 1.3.1.2)<br>• Modification of the inclusion criterion 5 (section 4.1 and synopsis)<br>• Modification of the exclusion criterion 5 and addition of the exclusion criterion 23 (section 4.2 and synopsis)<br>• Addition of a contraception note (section 6.9.1)<br>• Modification of durvalumab/oleclumab toxicity management and dose adjustements (section 7.2.1)<br>• Addition of paclitaxel toxicity management and dose adjustements for cardiac conduction abnormalities, hypotension, hypertension and bradychardia (section 7.3.1.1)<br>• Addition of cardiac assessment at screening, during study treatment period, at surgery, at the end of study treatment visit and during follow-up period (sections 8.1, 8.2.1, 8.2.2, 8.3 and 8.4)<br>• Addition of the cardiac assessment in the schedule of assessment	Belgium<br>France	☐ Yes<br>☐ No<br>☒Not applicable
4.0	01/03/2021	• Correction of the study design (synopsis, sections 3.1 and 3.2.1.2 and, figures 2, 4 and 6)<br>• Clarification of inclusion criteria 7 and 20 (synopsis, section 4.1)<br>• Subjects with cN1, cN2 or cN3 are eligible for the study	Belgium<br>France	☒ Yes<br>☐ No

		(synopsis, sections 4.1 and 8.1)<br>• Correction of exclusion criteria 24 (synopsis)<br>• Clarification of oleclumab administration timepoints (synopsos, sections 3.2.1.2, 3.2.2, 6.2.1.3 and figures 4 and 6)<br>• Removal of Harris scale (synopsis, sections 2.2.3, 8.1, 8.2.1 and 8.4)<br>• Correction of RCB as primary endpoint (synopsis, sections 1.2.4, 2.2.2, 12.1.1, 12.3, table 19)<br>• Added data favoring the combination of immunotherapy to chemotherapy in the neoadjuvant setting (section 1.2.2)<br>• Update of the rationale concerning the combination of radiation therapy with oleclumab (section 1.2.2.4)<br>• Clarification of postoperative radiation therapy and impact of SBRT on breast cosmesis (section 1.2.3.3)<br>• Added IDMC conclusion for Safety run-in (sections 1.3.1.2.2, 3.2.1, 3.2.1.6)<br>• Specified that the radiological work-up at week 12 is a breast MRI, instead of the previously mentioned 'breast radiological evaluation'(sections 3.2.3.2 and section 8.2.1)<br>• Clarification of the formulation for durvalumab and oleclumab (sections 6.1.1.1, 6.2.1.1)<br>• Removal of the preparation and administration of durvalumab and oleclumab (sections 6.1.1.1, 6.2.1.2)<br>• Update of the drug accountability logistic (sections 6.1.1.6, 6.2.1.6, 6.3.1.6, 6.4.1.6, 6.5.1.6)<br>• Clarification of surgical samples collected (section 6.7.1)		

		• Modification of the process of quality assurance for SBRT (section 6.8.1.1)<br>• Clarification about adjuvant radiotherapy (section 6.8.1.2)<br>• Addition of radiotherapy toxicites (section 6.8.2)<br>• Modification of the methods of contraception (section 6.9.1)<br>• Update of the guidance for specific immune-related adverse events (table 7)<br>• Clarification of the suggested treatment decisions when paclitaxel must be held or discontinued (section 7.3.1.1, table 10)<br>• Clarification of treatment management for ddAC (section 7.3.2.3 Table 14)<br>• Modification of the screening duration (section 8.1)<br>• Clarification of the process in case of unevaluable MammaPrint or PD-L1 result (sections 8.1 and 11.1)<br>• Addition of breast photography (sections 8.1, 8.2.1, 8.4 and schedule of assessment).<br>• Update of breast cosmetic evaluation timepoints (sections 8.1, 8.2.1, 8.4 and schedule of assessment)<br>• Removed mandatory blood urea measurements, creatinine measurements are sufficient (sections 8.1, 8.2.1, 8.2.2, 8.3, 8.4, schedule of assessment and appendix 1).<br>• Addition of CRP measurement (section 8.1, 8.2.1, 8.2.2, 8.3 and legend of schedule of assessment)<br>• Deletion of amylase testing (sections 8.1, 8.2.1, 8.2.2, 8.3, 8.4, legend of schedule of assessment and appendix 1)<br>• Addition of cortisol measurement for arm 2 and 3 (sections 8.1, 8.2.2 and schedule of assessment)		

		• Addition of optional lymph node biopsy (sections 8.1, 8.2.1 and 8.2.2)<br>• Mentioned that cardiac examinations at week 12 are only applicable for arm 2 and 3 (section 8.2.2 and legend of schedule of assessment)<br>• Clarification of the evaluation of local control of breast and lymph nodes (section 8.4)<br>• Clarification of the definition of AESI for durvalumab (section 10.2.4.1)<br>• Clarification of AESIs reporting (section 10.2.4.2 and section 10.2.5.2)<br>• Clarification of the tissue samples collection (sections 11.1.1 to 11.1.2 and section 11.3)<br>• Modification of the translational research objectives (section 11.2)<br>• Modification of the central biobank (section 11.5)<br>• Added interim analysis section 12.2<br>• Updated procedure for obtaining subject's consent (section 18.2.2)<br>• Updated of IDMC (section 20.1)<br>• Update of name and function of the representatives of the sponsor (section 21)<br>• Update of the dosing modification and toxicity management guidelines for durvalumab (Appendix 1)<br>• Implementation of an algorithm to select index lesion in case of multifocal or bilateral breast cancer (Appendix 2)<br>• Wording clarification		
5.0		• Addition of two new stratification factors in the randomization process (Primary tumour size: cT1/cT2 versus cT3 and Nodal status:	Belgium<br>France	☒ Yes<br>☐ No

		cN0 versus cN+) (synopsis and section 4.3)<br>• Clarification of Inclusion criterion N°5 (synopsis and section 4.1)<br>• Modification of Inclusion criterion N°7: Addition of the possibility to randomize the patient before knowing the MammaPrint result for a subset of patients with tumors that are grade III or Ki67>20% and depending of age and nodal status (synopsis, section 4.1, and section 8.1)<br>• Modification of Inclusion criterion N°8: Addition of the possibility to perform ultrasound instead of MRI in specific cases (synopsis and section 4.1)<br>• Modification of Inclusion criterion N°9: In particular conditions, biopsy of all foci are not mandatory (synopsis section 3.2.3.2 ,4.1 and 11.1.1.3)<br>• Clarification of Inclusion criterion N°15 (synopsis and section 4.1)<br>• Clarification of Inclusion criterion N°16: 28 days of screening (synopsis and section 4.1)<br>• Update of study rationale (section 1.2)<br>• Update of potential risks and benefits (section 1.3)<br>• Addition of the software program that will be used for breast cosmetic evaluation (section 2.2.3)<br>• Clarification on SBRT schedule in case of chemotherapy delay (section 3.2.3.1)<br>• Clarifications to the radiotherapy protocol (section 6.8.1.1)<br>• Update of the toxicity management and dose adjustement according to the		

		new version of the toxicity mangement guideline (section 7.1)<br>• Modification of toxicity management for neutrophil count decrease (section 7.3.1.1)<br>• Clarification of placement of MRI-compatible markers (section 8.1)<br>• Addition of the possibility to perform ultrasound instead of MRI (section 8.1; 8.2; 8.5)<br>• Addition of the possibility to perform screening MRI 2 months before enrolment (section 8.1)<br>• Addition of the time window allowed for coagulation tests (section 8.2, 8.5)<br>• Clarification of the time window allowed for the biospy post SBRT (section 8.2, 8.5)<br>• Clarification of the time window allowed for CT scan before SBRT (section 8.2)<br>• Addition of one breast photography assessment at 3 years (section 8.4, 8.5)<br>• Clarification for AESI reporting and addition of the new AESI: diabetes insipidus, pemphigoid, immune-mediated neutropenia, cholangitis sclerosing, immune-mediated cystitis (section 10.2)<br>• Update of the Appendix 1		
6.0		• Clarification of concomitant medication, therapies & procedures reporting (section 8.6)<br>• Clarification of AESI reporting (section 10.2)<br>• Change in volume of PBMC collection tube (section 11.1 and 11.3)<br>• Update of the evaluable subject definition (section 12.1, 12.2)	Belgium<br>France	☒ Yes<br>☐ No

		• Clarification of the studied population for final analysis (section 12.3)<br>• Added IDMC conclusion for safety analysis (section 12.2.2)<br>• Update of the Appendix 1		
7.0		• Update of the page layout to be ready for the transition under CTR<br>• Update of the study rationale after ESMO 2023 (section 12.1)<br>• FU period extended from 3 to 5 years (section 3 and 8)<br>• Update of objectives (section 2.1 and 11)<br>• Update of endpoints (section 2.2)<br>• Clarification of radiotherapy protocol (section 6.8)<br>• Clarification of AESI reporting during FU phase (section 10.2)<br>• Addition of the third safety analysis conclusions (section 12)<br>• Clarification of the statistical analysis to be performed for the final analysis (section 12)<br>• Addition of new safety information regarding immunotherapies (section 1.3.1 and update of Appendix 1)	Belgium<br>France	☒ Yes<br>☐ No
8.0		• Update according to the new CTR regulation<br>• Removal of the assesment of aspartate aminotransferase (AST) levels from the follow-up procedures<br>• Addition of the report of biological characteristics of tumors after surgery for patients who have not obtained a PCR.	Belgium<br>France	☒ Yes<br>☐ No

TABLE OF CONTENTS

LIST OF ABBREVIATIONS 34
1. BACKGROUND AND SCIENTIFIC RATIONALE 37
1.1. Background and General Rationale 37
1.1.1. Why luminal B breast cancer? 37
1.1.2. The immune response in luminal B breast cancer and the importance of priming the microenvironment 37
1.1.2.1. Single agent immunotherapy is probably ineffective in luminal B breast cancer 37
1.1.2.2. Priming of the immunologic response to increase efficacy of immunotherapy in luminal B breast cancer 38
1.1.3. Why the neo-adjuvant setting 38
1.2. In-depth rationale 39
1.2.1. Individual treatment components of the trial 39
1.2.1.1. Targeting PD-L1 with durvalumab (MEDI4736) 39
1.2.1.2. Targeting CD73 with oleclumab (MEDI9447) 40
1.2.1.3. Pre-operative radiation therapy to the primary breast cancer 41
1.2.2. Rationale of combining treatments and priming of the cancer microenvironment 42
1.2.2.1. Synergism between durvalumab and oleclumab 43
1.2.2.2. Rationale for combining durvalumab/oleclumab with chemotherapy 44
1.2.2.3. Synergism between SBRT and durvalumab 44
1.2.2.4. Synergism between SBRT and oleclumab 45
1.2.3. Rationale for SBRT dose and SBRT timing 45
1.2.3.1. Rationale for the choice of SBRT dose fractionation in combination with immunotherapy 45
1.2.3.2. Rationale for the timing of the combination of SBRT, immunotherapy and chemotherapy 46
1.2.3.3. Impact of pre-operative SBRT on standard adjuvant radiotherapy treatment 47
1.2.4. Rationale for giving radiation therapy in the three treatment arms 48
1.2.5. Are there similar ongoing trials? 48
1.3. Potential Risks and Benefits 49
1.3.1. Risks 49
1.3.1.1. Individual treatment components 49
1.3.1.2. Risks associated with the combination of treatment components 49
1.3.2. Benefits 50
2. OBJECTIVES AND ENDPOINTS OF THE STUDY 50
2.1. Objectives 50

2.1.1. Safety Run-in objectives 50
2.1.2. Phase II Primary objective 51
2.1.3. To demonstrate improved tumour response of the primary tumour and nodal metastases in arms 2 or 3 versus arm 1Phase II Secondary objectives 51
2.1.4. Phase II Exploratory objectives 51
2.2. Endpoints 52
2.2.1. Safety run-in endpoints 52
2.2.2. Phase II Primary endpoint 52
2.2.3. Phase II Secondary endpoints 52
2.2.4. Phase II Exploratory endpoints 53
3. STUDY DESIGN 54
3.1. General design of the trial 54
3.2. Pre-operative phase 55
3.2.1. Safety run-in [completed] 55
3.2.1.1. Rationale 55
3.2.1.2. Design 56
3.2.1.3. Subject enrolment rules during safety run-in 57
3.2.1.4. Requirements to meet before starting the phase II randomised trial 57
3.2.1.5. Procedure if the safety run-in conditions do not allow proceeding with Phase II randomised trial 59
3.2.1.6. Results of the safety run-in and IDMC recommendations 60
3.2.2. Phase II randomised trial 61
3.2.3. Treatment and work-up details 61
3.2.3.1. Pre-operative SBRT 61
3.2.3.2. Clinical and radiologic work up during pre-operative phase 62
3.3. Surgery and end of treatment 62
3.4. Follow-up 62
3.4.1. Adjuvant radiotherapy 63
3.4.2. Follow-up and hormonal treatment after study treatments 63
4. SELECTION OF SUBJECTS 63
4.1. Inclusion criteria 63
4.2. Exclusion criteria 65
4.3. Subject registration/randomisation procedure 67
5. CRITERIA FOR SUBJECT WITHDRAWAL 68
6. STUDY TREATMENTS 68
6.1. Investigational Medicinal Product: **Durvalumab** 68
6.1.1. Drug information 68
6.1.1.1. Formulation 69
6.1.1.2. Preparation and administration 69

6.1.1.3. Therapeutic regimen and dose 69
6.1.1.4. Stability and storage 69
6.1.1.5. Labelling 69
6.1.1.6. Drug logistics and accountability 69
6.1.2. Toxicity 70
6.2. Investigational Medicinal Product: **Oleclumab** 70
6.2.1. Drug information 70
6.2.1.1. Formulation 70
6.2.1.2. Preparation and administration 70
6.2.1.3. Therapeutic regimen and dose 70
6.2.1.4. Stability and storage 70
6.2.1.5. Labelling 70
6.2.1.6. Drug logistics and accountability 71
6.2.2. Toxicity 71
6.3. Investigational Medicinal Product: **Paclitaxel** 71
6.3.1. Drug information 71
6.3.1.1. Formulation 71
6.3.1.2. Preparation 71
6.3.1.3. Therapeutic regimen and dose 71
6.3.1.4. Stability and storage 71
6.3.1.5. Labelling 71
6.3.1.6. Drug logistics and accountability 72
6.3.2. Toxicity 72
6.4. Investigational Medicinal Product: **Doxorubicin** 72
6.4.1. Drug information 72
6.4.1.1. Formulation 72
6.4.1.2. Preparation 72
6.4.1.3. Therapeutic regimen and dose 72
6.4.1.4. Stability and storage 72
6.4.1.5. Labelling 73
6.4.1.6. Drug logistics and accountability 73
6.4.2. Toxicity 73
6.5. Investigational Medicinal Product: **Cyclophosphamide** 73
6.5.1. Drug information 73
6.5.1.1. Formulation 73
6.5.1.2. Preparation 73
6.5.1.3. Therapeutic regimen and dose 73
6.5.1.4. Stability and storage 74

6.5.1.5. Labelling ... 74
6.5.1.6. Drug logistics and accountability ... 74
6.5.2. Toxicity ... 74
6.6. Non-Investigational Medicinal Products (NIMPs) ... 74
6.6.1. G-CSF ... 74
6.6.2. Chemotherapy pre- and post-medication ... 74
6.6.3. Gadolinium-based contrast agents ... 74
6.6.4. Iodinated contrast agent ... 75
6.7. Protocol surgery ... 75
6.7.1. Surgical treatment ... 75
6.7.2. Surgery toxicity ... 75
6.8. Protocol radiotherapy ... 76
6.8.1. Radiotherapy treatment ... 76
6.8.1.1. **Stereotactic Body Radiotherapy (SBRT)** of the primary tumour in the breast. 76
6.8.1.2. Adjuvant radiotherapy ... 83
6.8.2. Radiotherapy toxicity ... 83
6.9. Contraception ... 84
6.9.1. Contraception ... 84
6.10. Menopausal status ... 84
6.11. Concomitant treatments ... 84
6.11.1. Allowed Concomitant treatments ... 85
6.11.2. Prohibited Concomitant treatments ... 85
6.11.3. Incompatibilities/Drug Interactions ... 85
6.11.3.1. Durvalumab ... 85
6.11.3.2. Oleclumab ... 86
6.11.3.3. Paclitaxel ... 86
6.11.3.4. Doxorubicin ... 86
6.11.3.5. Cyclophosphamide ... 87
6.11.3.6. Radiation Therapy ... 90
6.12. Study treatment duration ... 90
7. TOXICITY MANAGEMENT AND DOSE ADJUSTMENTS ... 90
7.1. General management of study drug delays ... 90
7.2. Durvalumab and oleclumab ... 91
7.2.1. Immune-related Adverse Events ... 91
7.2.2. Infusion-related reactions ... 92
7.3. Chemotherapy ... 93
7.3.1. Paclitaxel ... 94

7.3.1.1. Suggested treatment decisions when paclitaxel must be held or discontinued (**Table 9**)....... 94
7.3.1.2. Treatment management for paclitaxel-related neuropathy (**Table 10)** 96
7.3.1.3. Treatment management for paclitaxel-related musculoskeletal pain (**Table 11)** 97
7.3.2. ddAC 97
7.3.2.1. Cardiac AEs during ddAC (**Table 12**)....... 97
7.3.2.2. Non-cardiac toxicity....... 98
7.3.2.3. Treatment management for ddAC (**Table 13**) 98
7.3.3. Infusion-related Reactions (IRRs) 100
7.4. Radiation therapy 101
8. SCHEDULE OF ASSESSMENTS....... 102
8.1. Screening period 102
8.2. Study treatment period 105
8.2.1. Chemotherapy in combination or not with immunotherapy and SBRT (= 19 weeks). 105
8.2.2. Surgery....... 107
8.3. End of study treatment visit 108
8.4. Follow-up period....... 109
8.5. End of study visit 110
8.6. Schedule of assessments: Phase II....... 110
8.7. Schedule of assessments: Phase II – Follow-up Period 113
9. ASSESSMENT OF EFFICACY 115
10. ASSESSMENT OF SAFETY 115
10.1. Safety parameters and Methods for assessing safety parameters....... 115
10.2. Pharmacovigilance parameters definitions and reporting guidance....... 116
10.2.1. Adverse Events....... 116
10.2.1.1. Definitions....... 116
10.2.1.2. AE intensity....... 116
10.2.1.3. Relationship to study treatments....... 117
10.2.1.4. Reporting of adverse events 117
10.2.2. Serious Adverse Events....... 118
10.2.2.1. Definitions....... 118
10.2.2.2. Reporting of serious adverse events by the investigators....... 119
10.2.2.3. Reporting of serious adverse events by the sponsor....... 120
10.2.3. Exceptions to AEs/SAEs reporting 120
10.2.4. Adverse Events of Special Interest for durvalumab: immune-mediated adverse events 121
10.2.4.1. Definition....... 121

10.2.4.2. Reporting of AESIs for durvalumab ... 122
10.2.5. Adverse events of special interest for oleclumab: cardiovascular adverse events 122
10.2.5.1. Definition ... 122
10.2.5.2. Reporting of AESIs for oleclumab ... 123
10.2.6. Potential Hy's law case ... 123
10.2.6.1. Definition ... 123
10.2.6.2. Reporting ... 123
10.2.7. Pregnancy ... 123
10.2.8. Overdose, misuse and abuse ... 124
10.2.8.1. Definitions ... 124
10.2.8.2. Reporting of overdose, misuse and abuse ... 124
10.2.9. Medication Error ... 124
10.2.9.1. Definition ... 124
10.2.9.2. Reporting of medication error ... 124
11. TRANSLATIONAL RESEARCH(ES) ... 125
11.1. Overview of tissue and blood sample collection ... 125
11.1.1. Tissue sample collection ... 125
11.1.1.1. Overview ... 125
11.1.1.2. MammaPrint testing and PD-L1 testing ... 126
11.1.1.3. Bilateral, multifocal or multicentric disease ... 126
11.1.1.4. Unacceptable tissue samples ... 126
11.1.2. Blood samples collection ... 126
11.2. Objectives ... 127
11.3. Timelines ... 129
11.4. Biological material handling ... 130
11.5. Central biobanking of biological material collected during the trial and Destruction 130
11.6. Future undefined research ... 131
12. STATISTICAL CONSIDERATIONS ... 131
12.1. Statistical design ... 131
12.1.1. Sample size ... 131
12.1.2. Study duration ... 132
12.1.2.1. Expected Accrual Time ... 132
12.1.2.2. Expected Duration of the Trial ... 132
12.2. Interim analyses ... 133
12.2.1. Safety run-in ... 133
12.2.2. Second safety analysis ... 133
12.2.3. Analysis for futility of arm 2 and 3 and third safety analysis. ... 133

12.3. Final analysis 134
12.3.1. Populations 134
12.3.2. Analyses 134
12.3.3. Timeline of analysis of the different endpoints 136
13. FIRST ACT OF RECRUITMENT 136
14. SUSPENSION OR TRIAL TERMINATION 136
15. END OF STUDY 137
16. DIRECT ACCESS TO SOURCE DATA/DOCUMENTS 137
17. QUALITY CONTROL AND QUALITY ASSURANCE 137
17.1. Quality control 137
17.2. Quality assurance 138
18. REGULATORY AND ETHICAL CONSIDERATIONS 138
18.1. Obligations 138
18.1.1. Trial Master File 138
18.1.2. Regulations, laws and guidelines 138
18.1.3. Authorisation 138
18.1.4. Modifications 138
18.1.5. Notifications 139
18.1.6. Summary of results 139
18.2. Informed consent form and procedure for obtaining subject's consent 139
18.2.1. The informed consent 139
18.2.2. The procedure for obtaining subject's consent 140
18.3. Subject identification 142
18.4. Privacy Guarantee 142
19. DATA HANDLING AND RECORD KEEPING 144
19.1. Investigator's files and subject's clinical source documents 144
19.2. Case Report Forms (CRF) 145
19.3. Retention of documents 146
19.4. Adequate arrangements to secure data compliant with GDPR 147
19.5. Measures implemented in case of data security breach 148
19.5.1. The procedure if a Data Processor causes a data breach 148
19.5.2. Notification of the data breach 148
19.5.3. Risk assessment 149
19.5.4. Measures to mitigate the adverse effect 149
19.5.5. Record keeping 149
20. STUDY OVERSIGHT 149
20.1. Independent Data Monitoring Committee 149
20.2. Scientific Committee 150

21. STUDY SPONSORSHIP AND FINANCING 152
22. STUDY INSURANCE 152
23. PUBLICATION POLICY 152
24. REFERENCES.. 152
Appendix 1: Toxicity Management Guidelines.. 16262
Appendix 2 – Algorithm to select index lesion in case of multifocal or bilateral breast cancer 16363

SYNOPSIS

**STUDY TITLE**	Neo-adjuvant chemotherapy combined with SBRT to the primary tumour +/- durvalumab (MEDI4736), +/- oleclumab (MEDI9447) in luminal B breast cancer: a phase II randomized trial
**SHORT TITLE**	Neo-CheckRay
**SPONSOR**	Institut Jules Bordet (IJB) - Brussels, Belgium
**INDICATION(S)**	Luminal B Breast Cancer
**TARGET STUDY POPULATION**	Pre or post-menopausal female subjects with luminal B primary breast cancer who are candidates for neo-adjuvant chemotherapy
**PHASE**	Phase II with a safety run-in for the first 6 subjects
**STUDY DESIGN**	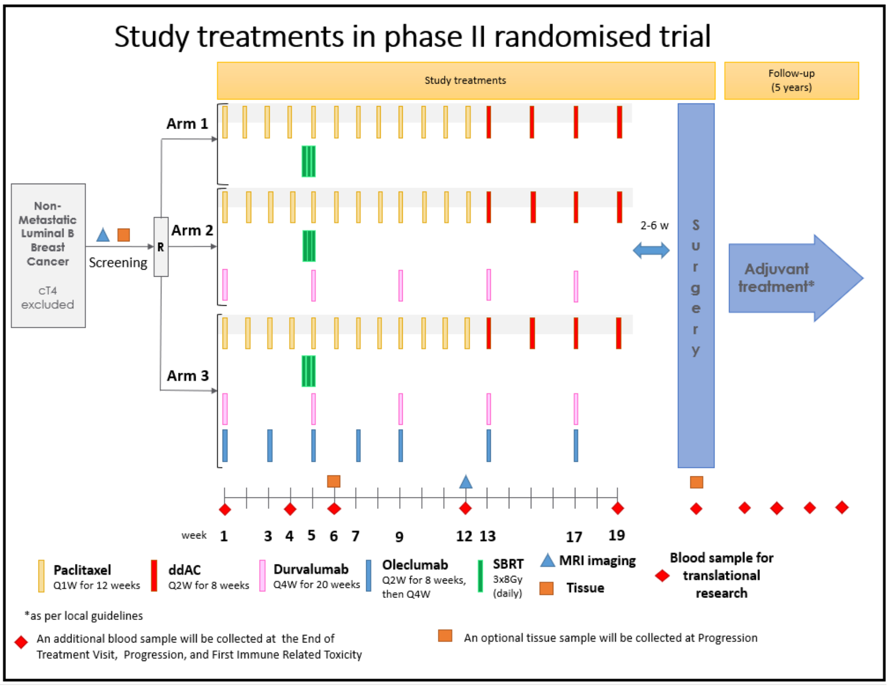<br><br>NEO-CHECKRAY is a multicenter, open-label phase II study that randomises luminal B breast cancer subjects candidate for neo-adjuvant chemotherapy in a 1:1:1 ratio in 3 arms:<br>1. Arm1: the combination of weekly paclitaxel followed by dose-dense doxorubicin-cyclophosphamide (ddAC) and pre-operative radiation therapy (boost dose) on the primary tumour<br>2. Arm 2: arm 1 with the addition of the anti-PD-L1 antibody durvalumab<br>3. Arm 3: arm 2 with the addition of the anti-CD73 antibody oleclumab<br>The primary tumour will be excised 2-6 weeks after completion of ddAC. A safety run-in is planned for the 6 first subjects before starting

	the randomised phase II trial. Those 6 subjects will receive the treatment given in Arm 3.<br><br>The randomization process is based on 4 stratification factors:<br>• PD-L1 status determined centrally: low versus high.<br>• Primary tumour size: cT1/cT2 versus cT3<br>• Nodal status: cN0 versus cN+<br>• Participating site
**STUDY RATIONALE**	Luminal breast cancer is characterised by a positive estrogen receptor (ER) status and categorised into two subclasses, A and B (1). Breast cancer-specific mortality rate is twice as high in luminal B subjects relative to luminal A subjects. The rates of pathological complete response (pCR) with chemotherapy, a surrogate marker for long-term benefit of neo-adjuvant treatment, compares poorly between luminal B and other types: 15% in luminal B versus 45.8% in HER2 positive and 44.5% in triple negative breast cancer (TNBC) (2–4). Luminal B breast cancer subjects who cannot achieve a pCR after neo-adjuvant treatment have a significant diminished event-free survival, revealing a need to focus innovative research on effective neo-adjuvant treatments for luminal B breast cancer (5).<br>More recently, important developments in immuno-oncology permit classification of tumours according to their immunological susceptibility: inflamed cancer types are characterised by the presence of tumour infiltrating lymphocytes (TILs), programmed cell death receptor ligand 1 (PD-L1) positivity of tumours or immune cells and high CD8+ T-cell density. They attain overall better long term outcomes with systemic therapy, greatly due to this immune susceptibility (6). Non-inflamed cancers, such as luminal B breast cancer, are also known as ‘immune cold tumours’. An area of active research is how to turn ‘immune cold tumours’ into inflamed cancers, leveraging the effects of systemic treatment and increasing pCR rates after treatment by immunotherapy. Although immunotherapy has a major role for the treatment of inflamed cancers, such as melanomas and lung cancer, with a considerable fraction of subjects attaining long lasting benefits of it, it has been shown that isolated PD1 and PD-L1 blockade are disappointing in the setting of an immune cold tumour, hence priming immunologic response to increase its effects are important (7–9). Priming of immunotherapy responses could be attained with a myriad of strategies: by combining it with chemotherapy, with blockade of the adenosine pathway and with radiation.<br>Positive clinical data for the *first strategy* is already available: it was demonstrated in the I-SPY 2 trial that the addition of pembrolizumab, an anti-PD1, to standard neo-adjuvant chemotherapy (paclitaxel followed by doxorubicin and cyclophosphamide) for luminal B breast cancer subjects, increased pCR rates (30% vs 13%) compared to standard chemotherapy alone(10).<br><br>Regarding the *second strategy*, there are three phase I trials ongoing with immunotherapy and adenosine pathway blockade in subjects

	with solid tumours. Extracellular adenosine promotes tumour cell metastasis, angiogenesis and has multiple immunosuppressive functions (11).<br>Within the *third strategy*, radiosensitising immunotherapy is considered a potential curative therapeutic modality, akin to radiosensitising chemotherapy (12). Radiation induces changes to the tumour cell immunophenotype by a variety of mechanisms and enhances cross-presentation of tumour antigens that can induce abscopal effects outside of the radiation field, radiation thereby acting as an in situ anti-tumour vaccine (13). Currently, a phase I trial is ongoing to demonstrate safety and tolerability of pembrolizumab combined with radiation therapy on the primary tumour for TNBC and HR +/HER2 - breast cancer subjects. Furthermore, pre-clinical research demonstrated that adenosine regulates the ability of radiation therapy to induce anti-tumour immunity, by affecting dendritic cells maturation and T-cell activation (14). These findings suggest that CD73 blockade is a promising strategy to improve the synergy between radiation therapy and immunotherapy.<br>Considering all the synergistic effect and available safety data of chemotherapy with radiation, immunotherapy and blockade of the adenosine pathway, and the lack of efficacious treatment for luminal B breast cancer, we are thus proposing this trial of neo-adjuvant treatment with weekly paclitaxel followed by dose-dense doxorubicin-cyclophosphamide (ddAC-T) with pre-operative radiation to the primary tumour, durvalumab (anti-PD-L1) and oleclumab (anti-CD73) to enable capturing of a possible benefit with an easy-to-assess end-point (pCR) in the earliest and most susceptible stage available of the disease, allowing a rapid and thorough assessment of efficacy and safety, together with performance of translational research on the surgical specimen.
**OBJECTIVES**	**Safety Run-in**<br>• To evaluate safety and toxicity of adding SBRT directed to the primary tumour to the combination durvalumab-oleclumab-paclitaxel.<br>• To evaluate feasibility of performing surgery (breast conserving surgery or mastectomy) within 6 weeks after the end of neo-adjuvant treatment.<br>**Phase II Primary objective**<br>• To demonstrate improved tumour response of the primary tumour and nodal metastases in arms 2 or 3 versus arm 1.

	**Phase II Secondary objectives**<br><br>***At surgery:***<br><br>• To evaluate the complete pathological response rate defined as ypT0/Tis ypN0: absence of residual invasive disease, residual in situ carcinoma is accepted.<br>• To evaluate the complete pathological response rate defined as ypT0 ypN0: absence of residual invasive disease *and* in situ carcinoma.<br>• To evaluate the response to the primary tumour irrespective of the response to the pathological lymph nodes.<br>• To evaluate the response to the pathological lymph nodes irrespective of the response to the primary tumour.<br>• To evaluate the feasibility to perform breast-sparing surgery of the arms 2 and 3 versus arm 1.<br>• Demonstrate an increase in TIL levels of the primary breast cancer between baseline and the week 6 biopsy.<br><br>***Follow-up phase:***<br><br>• To evaluate the ability to control invasive disease and survival in arms 2 and 3 versus arm 1 at year 3 and 5 years after surgery.<br>• To evaluate the severity and duration of AEs of the arms 2 and 3 versus arm 1.<br>• To evaluate the cosmetic changes to the breast of the arms 2 and 3 versus arm 1.<br><br>**Phase II Exploratory objectives**<br><br>• Evaluation of ovarian function 1, 2, 3 and 5 years after surgery in premenopausal subjects and perform comparison between treatment arms and in comparison to baseline (week 1 blood for translational research).<br>• Collect data about pregnancy and breastfeeding in the follow-up phase.<br>• Evaluate if biopsy at week 6 can predict the occurrence of pathological complete response (pCR) at surgery.<br>• Evaluate the use of plasma ctDNA in the pre-operative phase to predict treatment response.<br>• Evaluate the use of plasma ctDNA in the follow-up phase to predict cancer relapse.

	• Evaluate MRI images at baseline and at week 12 to predict response to surgery and investigate the correlation with TIL dynamics.
**ENDPOINTS**	**Safety run-in endpoints**<br>• Occurrence of immune related or radiation therapy related toxicity of special interest<br>• Feasibility of delivering a sufficient dose of paclitaxel and ddAC<br>• Feasibility of performing surgery within a specified timeframe after the last neo-adjuvant treatment<br>**Phase II Primary endpoint**<br>• Residual cancer burden (**RCB 0-1 vs. RCB 2-3**) at time of surgery. RCB 0 is defined as pathological complete response (pCR) and RCB 1 is defined as minimal residual disease. RCB is calculated as a continuous index combining pathologic measurements of the primary tumour (size and cellularity) and nodal metastases (number and size) as defined by Symmans et al. (15).<br>**Phase II Secondary endpoints**<br>***At surgery***<br>• Rate of pCR - ypT0/Tis ypN0, defined as the absence of residual invasive cancer at the time of definitive surgery.<br>• Rate of pCR - no DCIS (yp DCIS (ypT0 ypN0), defined as the absence of residual invasive and in situ cancer at time of definitive surgery.<br>• Complete pathologic response rate (pCR) of the primary tumour (ypT0/ Tis), irrespective of the response rate of the resected nodal metastases.<br>• Complete pathologic response rate (pCR) of the resected nodal metastases (ypN0), irrespective of the response rate of the primary tumour.<br>• % of breast conservation surgery in arms 2 and 3 versus arm 1<br>• Change in TIL levels between baseline and the week 6 biopsy.<br>***Follow-up Phase***

	• Efficacy endpoints at 3 years and 5 years after surgery will be measured, as defined by the Standardized Definitions for Efficacy End Points in Neoadjuvant Breast Cancer Clinical Trials (NeoSTEEP) (88). The following endpoints will be assessed: event-free survival (**EFS**), breast cancer event-free survival (**BC-EFS**), overall survival (**OS**) and distant recurrence-free survival (**DRFS**). Furthermore, the occurrence of **ipsilateral locoregional recurrence** (breast, chestwall or locoregional nodal recurrence), **ipsilateral local recurrence** (breast or chest wall) (laterality of the index lesion), and **ipsilateral locoregional nodal recurrence** (laterality of the index lesion) will be assessed. The endpoints will be measured using regular follow-up investigations: lab work, clinical examination and annual breast ultrasound and mammography. Radiologic imaging will not be routinely performed, unless directed by abnormal blood results or clinical examination.<br>• Duration and severity of AEs based on CTCAE 5.0.<br>• Changes in breast appearance: breast fibrosis in whole breast, breast fibrosis in boost area, breast size, breast shape, nipple position, shape of the areola and nipple, skin color, appearance of surgical scar, evaluation of teleangiectasia and global cosmetic result. Information on cosmetic and plastic surgical procedures will be collected (for example: oncoloplastic surgery, breast implants, and other procedures).<br><br>**Phase II Exploratory endpoints**<br><br>• Measurement of ovarian function in premenopausal women, with comparison between treatment arms (92). Ovarian function will be measured in blood by evaluation of anti-Mullerian hormone (AMH), follicle stimulating hormone (FSH), estradiol (E2) and progesterone at baseline and after surgery at 1 year, 2 years, 3 years and 5 years in premenopausal subjects.<br>• Information about pregnancies, pregnancies attempts and breasfeeding. The following data will be collected: spontaneous menstruation recovery, number of pregnancy attempts, number of pregnancies (determined by pregnancy test), pregnancy outcome (full term pregnancy, caesarean section, abortion, miscarriage, ectopic, stillbirth), offspring outcomes (preterm birth, low birth weight, birth defects), breastfeeding (pattern of breastfeeding, duration, use of ipsilateral breast if

	previous breast conservation, side exclusivity), use of assisted reproductive technology (ART).<br>• The correlation between cellularity and biomarkers on the week 6 biopsy and pathological complete response (pCR) at surgery.<br>• The correlation between plasma ctDNA during the pre-operative phase and tumor response at surgery.<br>• The correlation between plasma ctDNA and clinical tumor relapse in the follow-up phase.<br>• The correlation between MRI images at baseline and at week 12 with the response to surgery and changes in TILs.
**NUMBER OF SUBJECTS**	**Safety Run-in:**<br>- Number of subjects to enrol: 6<br>**Phase II**<br>- Estimated number of subjects to screen: 184<br>- Estimated number of subjects 18-64 y.o. : 110<br>- Estimated number of subjects >64 y.o. : 74<br>- Estimated number of subjects to randomise: 147<br>- Number of evaluable subjects: 132
**INCLUSION CRITERIA**	Subjects must meet all of the following criteria in order to be eligible for this study:<br>1. Age ≥ 18 years old<br>2. Female<br>3. ECOG performance status ≤ 1<br>4. Weight ≥ 35 kg<br>5. Histological diagnosis of invasive breast adenocarcinoma that is estrogen receptor-positive (ER-positive) and HER2- negative as per the updated American Society of Clinical Oncology (ASCO) - College of American Pathologists (CAP) guidelines and performed according to local testing. In addition, only tumours with Proliferation Index Ki67 ≥ 15% **or** histology grade III are accepted.<br>6. Agreement to perform new study related biopsies to provide tissue samples.<br>7. MammaPrint genomic high risk score according to centralised testing, except for specific conditions as mentioned below. MammaPrint will only be tested for luminal B breast tumours with either Proliferation Index Ki67 ≥ 15% or histology grade III tumours. (Testing to be done during screening period).<br>**MammaPrint result status at time of termination of all other screening procedures:**<br>• *MammaPrint is high risk*: subject may be randomized.

- *MammaPrint is low risk*: subject can not be randomized.
- *MammaPrint result is not yet known*:

  If the MammaPrint result is not known at time of termination of all other screening procedures, the investigator is allowed to randomize the subject and start study treatment without waiting for the result of the MammaPrint in the following situations:

<table>
<tr><th></th><th></th><th>Age < 50 years</th><th>Age ≥ 50 years</th></tr>
<tr><td rowspan="2">Ki67 ≤ 20%

and grade II</td><td>cN0</td><td colspan="2" rowspan="2">Wait for MammaPrint result</td></tr>
<tr><td>cN+</td></tr>
<tr><td rowspan="2">Ki67 > 20 %

or grade III</td><td>cN0</td><td>Allowed not to wait for MammaPrint result</td><td>Wait for MammaPrint result</td></tr>
<tr><td>cN+</td><td colspan="2">Allowed not to wait for MammaPrint result</td></tr>
</table>

  If the subject is randomized in a situation without known MammaPrint result and the MammaPrint result proves to be low risk after randomisation, the patient will stay in the study.

- *MammaPrint result is unevaluable or is technically impossible*:

  The sponsor should be contacted as soon as possible to discuss the inclusion of the concerned subject. Under specific medical conditions and breast cancer disease characteristics, the medical team of the sponsor can accept that the site continues the screening process and randomization of the subject. There will be maximum 5% of non-evaluable MammaPrint results among enrolled patients..

8. Tumour size:
   - If subject is cN0: tumour size ≥ 2 cm, as determined by MRI imaging.
   - If subject is cN1,cN2 or cN3: tumour size: ≥ 1.5 cm, as determined by MRI imaging.

   The requirement for an MRI is not applicable in the case of medical contraindications to perform MRI (e.g., obesity or claustrophobia). In this situation, tumour evaluations should be performed by ultrasound.
9. Multifocal, multicentric unilateral or bilateral breast adenocarcinoma tumours are allowed provided that all biopsiable foci are ER+/HER2- according to local testing and all foci are able to receive SBRT treatment within the defined dosimetric

	constraints. In some cases a separate biopsy of every focus is not mandatory, but only if every of the following conditions are present:<br>• small focal lesion<br>• lesion in close proximity to the main primary cancer from which a biopsy was taken<br>• the investigator and the radiologist consider the lesion to be clearly related to the main primary breast cancer from which a biopsy was taken<br>• the lesion will be removed during the same lumpectomy than the main primary breast cancer<br>For bilateral, multifocal or multicentric disease, the site selected for pre-treatment biopsy should correspond to the site of largest measurable disease meeting eligibility criteria. The location of tumour biopsy site (laterality, quadrant, position from the nipple and type of imaging modality to guide biopsy) should be collected.<br>10. Serum pregnancy test (for subjects of childbearing potential) negative within 2 weeks prior to first dose of study administration.<br>11. Women of childbearing potential must agree to use 1 highly effective method of contraception during the screening period, during the course of the study and at least 12 months after the last administration of study treatment. It is strongly recommended for the male partner of a female subject to also use male condom plus spermicide throughout this period.<br>12. Adequate bone marrow function as defined below:<br>• Absolute neutrophil count ≥1500/µL, i.e. 1.5x10^9/L<br>• Hemoglobin ≥ 9.0 g/dL<br>• Platelets ≥100000/µL, i.e. 100x10^9/L<br>13. Adequate liver function as defined below:<br>• Serum total bilirubin ≤ 1.5 x ULN. In case of known Gilbert's syndrome ≤ 3 x UNL is allowed<br>• AST (SGOT) ≤ 3.0 x ULN<br>• ALT (SGPT) ≤ 3.0 x ULN<br>14. Adequate renal function as defined below:<br>• Creatinine ≤ 1.5 x UNL or eGFR≥40ml/min/1.73m^2<br>15. Adequate coagulant function as defined below:<br>• International Normalized Ratio (INR) ≤ 1.5 x ULN<br>16. Completion of all necessary screening procedures within 28 days prior to randomisation (except if written differently).<br>17. Willingness to provide tissue and blood samples for immuno-monitoring and translational research activities<br>18. Left ventricular ejection fraction (LVEF) ≥ 50%. LVEF performed in routine is accepted if done within 6 months prior to beginning of screening.<br>19. Signed Informed Consent form (ICF) obtained prior to any study related procedure.

	**Inclusion criterion for phase II only (all phase II subjects):**<br>20. Tumour sample provided for central PD-L1 IHC assessment (Testing done during screening period).<br>**Inclusion criterion applicable to FRANCE only (Safety run-in and Phase II subjects)**<br>21. Affiliated to the French Social Security System (applicable only to subjects treated in France)
**EXCLUSION CRITERIA**	Subjects meeting one of the following criteria are not eligible for this study:<br>1. Pregnant and/or lactating women.<br>2. Subject with a significant medical, neuro-psychiatric, substance abuse or surgical condition, currently uncontrolled by treatment, which, in the principal investigator's opinion, may interfere with completion of the study.<br>3. TNM stage cT4 breast cancer including inflammatory breast cancer<br>4. Presence of any distant metastasis<br>5. Contra-indication for treatment by paclitaxel, doxorubicin or cyclophosphamide, or known allergy to any tested substance or excipients (e.g; chemotherapy or immunotherapy formulations). Contra-indication for subjects with known sensitivity to acetaminophen/paracetamol, diphenhydramine or equivalent antihistamine (this is a contra-indication for treatment with oleclumab).<br>6. Previously known contra-indication for treatment by radiation therapy such as rare genetic disorders associated with DNA repair disorders such as ataxia-telangiectasia (A-T), Nijmegen Breakage Syndrome (NBS) and Fanconi anemia.<br>7. Active or prior documented autoimmune disease (including inflammatory bowel disease, celiac disease, Wegener's granulomatosis) within the past 3 years. NOTE: Subjects with childhood atopy or asthma, vitiligo, alopecia, Grave's disease, Hashimoto's thyroiditis, or psoriasis not requiring systemic treatment (within the past 2 years) are not excluded<br>8. Prior malignancy active within the previous 5 years, except for localised cancers that are considered to have been cured and in the opinion of the investigator present a low risk for recurrence. Examples include basal or squamous cell skin cancer, superficial bladder cancer, or carcinoma in situ of the cervix or breast<br>9. Known history of, or any evidence of active, non-infectious pneumonitis.<br>10. Active infection including:<br>• Tuberculosis (TB) (clinical evaluation that includes clinical history, physical examination and radiographic findings, and TB testing in line with local practice)

	• Hepatitis B (known positive HBV surface antigen (HBsAg) result). Subjects with a past or resolved HBV infection (defined as the presence of hepatitis B core antibody [anti-HBc] and absence of HBsAg) are eligible.<br>• Hepatitis C. Subjects positive for hepatitis C (HCV) antibody are eligible only if polymerase chain reaction is negative for HCV RNA.<br>11. Significant cardiovascular disease, such as New York Heart Association cardiac disease (Class II or greater), myocardial infarction transient ischemic attack, or stroke within the previous 3 months, unstable arrhythmias, and/or unstable angina<br>12. Medical condition requiring current systemic anticoagulation, or a history of congenital hypercoagulable condition. Subjects taking aspirin at doses < 325 mg per day are eligible provided that prothrombin time is within the institutional range of normal. Use of local anticoagulation for port maintenance is permitted.<br>13. Subjects with history of venous thrombosis in the past 12 months prior to the scheduled first dose of study treatment (oleclumab)<br>14. Diabetes mellitus Type 1 or poorly controlled Type 2 diabetes mellitus defined as a screening hemoglobin A1C ≥ 8 % or a fasting plasma glucose ≥ 160 mg/dL (or 8.8 mmol/L)<br>15. Any live (attenuated) vaccine within 30 days of planned start of study therapy<br>16. Prior systemic immunosuppressive medication (excluding corticosteroids) within 30 days of planned start of study therapy<br>17. Prior radiation therapy to the ipsilateral breast.<br>18. Prior immunotherapy, including tumour vaccine, cytokine, anti-CTLA4, PD-1/PD-L1, including durvalumab, blockade or similar agents<br>19. Concomitant use of other investigational drugs<br>20. Any unresolved toxicity NCI CTCAE Grade ≥2 from previous anticancer therapy with the exception of alopecia, vitiligo, and the laboratory values defined in the inclusion criteria. Subjects with Grade ≥2 neuropathy will be evaluated on a case-by-case basis after consultation with the Study Physician. Subjects with irreversible toxicity not reasonably expected to be exacerbated by treatment with durvalumab or oleclumab may be included only after consultation with the Study Physician.<br>21. Uncontrolled intercurrent illness, including but not limited to, ongoing or active infection, symptomatic congestive heart failure, uncontrolled hypertension, unstable angina pectoris, cardiac arrhythmia, interstitial lung disease, serious chronic gastrointestinal conditions associated with diarrhea, or psychiatric illness/social situations that would limit compliance with study requirement, substantially increase risk of incurring

	AEs or compromise the ability of the subject to give written informed consent.<br>22. History or current evidence of any condition, therapy, or laboratory abnormality that might confound the results of the trial, interfere with the subject's participation for the full duration of the trial, or is not in the best interest of the subject to participate, in the opinion of the treating investigator.<br>23. Prior organ transplantation.<br>24. Subjects with urinary outflow obstruction.<br>**Exclusion criterion applicable to FRANCE only (Safety run-in and Phase II subjects)**<br>25. Vulnerable persons according to the article L.1121-6 of the CSP, adults who are the subject of a measure of legal protection or unable to express their consent according to article L.1121-8 of the CSP
**INVESTIGATIONAL MEDICINAL PRODUCT(S) DOSE/ROUTE/REGIMEN**	• **Durvalumab** (MEDI4736) 1500 mg every 4 weeks (q4w)<br>• **Oleclumab** (MEDI9447) 3000 mg every 2 weeks (q2w) for the first 4 administrations, then every 4 weeks (q4w) for the last 3 administrations.<br>• **Paclitaxel** (80 mg/m^2) administered via IV infusion weekly for 12 weeks<br>• Dose-dense **doxorubicin** (60 mg/m^2) IV + cyclophosphamide (600 mg/m^2) IV day 1, every 2 weeks for 4 doses.
**NON-INVESTIGATIONAL MEDICINAL PRODUCT(S) DOSE/ROUTE/REGIMEN**	• **G-CSF** (i.e., filgrastim or pegfilgrastim) G-CSF can be given to any subject at any time in fitting with the local site guidelines<br>• Gadolinium-based contrast agents<br>• Iodinated contrast agent<br>• Chemotherapy pre- and post-medication including anti-emetics, antihistamines and steroids are administered according to local standard of care.
**NON-MEDICINAL STUDY TREATMENT(S)**	• **Stereotactic Body Radiotherapy (SBRT)** of the primary tumour in the breast.<br>• Surgery<br>• mastectomy or tumourectomy<br>• sentinel node procedure or axillary lymph node clearance (according to local centre treatment protocol)
**CONCOMITANT MEDICATIONS**	**Not allowed:**<br>Therapies for cancers including chemotherapy, immunotherapy and hormonal anticancer treatment are not allowed while the subjects are on study treatment.<br>Inducers and strong inhibitors of CYP3A4 and CYP2B6 should be avoided if possible.

**ASSESSMENT OF EFFICACY**	• Assessment of efficacy is discussed in the section endpoints above and in the section statistical analyses hereunder.
**ASSESSMENT OF SAFETY**	Clinical and laboratory adverse events (AEs) and serious adverse events (SAEs) will be reported and graded according to the National Cancer Institute Common Terminology Criteria for Adverse Events (NCI-CTCAE) version 5.0
**STATISTICAL ANALYSES**	**Primary comparison:**<br>This analysis will be carried out on the eligible and randomised subjects who have been operated and in whom the residual cancer burden was measured. The observed proportions will be compared by a chi square test without continuity correction at an alpha two-sided level of 2.5%. Confidence intervals for the difference between proportions will be provided at the usual 95% level. The primary analyses will compare arm 1 and arm 2 as well as arm 1 and arm 3.<br>As exploratory analysis, a confidence interval for the difference between proportions in arm 2 and arm 3 will be provided.
**TRANSLATIONAL RESEARCH(ES)**	• **Blood** samples will be drawn at baseline and during treatment in weeks 1, 4, 6, 12 and end of chemo (week 19 if no treatment interruptions), as well at surgery, end of treatment visit, during the follow-up phase at 1 year, 2 years, 3 years and 5 years; first immune related toxicity and first progressive disease<br>• Tissue **biopsies** will be obtained at baseline and a second time in week 6. An optional tissue sample could also be collected at disease progression, if that occurs.<br>• Tissue from **surgical specimens** will be obtained in all eligible subjects.
**LENGTH OF THE STUDY**	**Safety Run-In**<br>• Planned recruitment period : 2 months<br>• Planned treatment period for a subject : 6 months<br>• Planned follow-up period for a subject : up to 36 months<br>**Phase II**<br>• Planned recruitment period : 24 months<br>• Planned treatment period for a subject : 6 months<br>• Planned follow-up period for a subject : up to 60 months
**END OF STUDY**	• After last follow up visit of the last subject<br>• The trial is mature for the analysis of the endpoints as defined in the protocol, if the trial reaches its endpoints<br>• The database has fully cleaned and frozen for all analyses

LIST OF ABBREVIATIONS

Abbreviation	Description
ADCC	Antibody-dependent cell-mediated cytotoxicity
ADL	Activities of Daily Living
AE	Adverse Event
AESI	Adverse Event of Special Interest
ALT	Alanine Amninotransferase
AST	Aspartate Amninotransferase
AMP	Adenosine monophosphate
APBI	Accelerated partial breast irradiation
AR	Adverse Reaction
ASCO	American Society of Clinical Oncology
A-T	Ataxia-Telangiectasia
BIG	Breast International Group
CAP	College of American Pathologists
CD	Cluster of Differentiation
cGAS	Cyclic GMP-AMP synthase
ctDNA	circulating tumour DNA
CTLA-4	Cytotoxic T-Lymphocyte Associated Protein 4
ddAC	dose-dense doxorubicin-cyclophosphamide chemotherapy regimen
DFS	Disease Free Survival
DLT	Dose limiting toxicity
DNA	Deoxyribonucleic acid
(e)CRF	(Electronic) Case Report Form
DNA	Deoxyribonucleic Acid
EC	Epirubicin-Cyclophosphamide
ECOG	Eastern Cooperative Oncology Group
EFS	Event Free Survival
EOS	End Of Study
EOT	End Of Treatment
ER	Estogen receptor
EVCTM	EudraVigilance Clinical Trial Module
FEC	Fluorouracil-Epirubicin-Cyclophosphamide
FFPE	Formalin-Fixed paraffin-embedded
FISH	Fluorescence In Situ Hybridization

Abbreviation	Description
GFR	Glomerular Filtration Rate
GMP	Good Manufacturing Practice
GP	General Practitioner
HBsAg	Hepatitis B Surface Antigen
HCV	Hepatitis C Virus
HER2	Human epidermal growth factor receptor 2
HIV	Human Immunodeficiency Virus
IB	Investigators Brochure
ICH-GCP	International Conference on Harmonisation – Good Clinical Practice
ICF	Informed Consent Form
iDFS	Invasive Disease Free Survival
IDMC	Independent Data Monitoring Committee
IEC	Independent Ethical Committee
IgG1κ	Immunoglobulin G1 kappa
IgG1λ	Immunoglobulin G1 lambda
IHC	Immunohistochemistry
IJB	Institut Jules Bordet
ILC	Invasive Lobular Carcinoma
IMP	Investigational Medicinal Product
ISF	Investigator’s study file
ISG	Interferon-stimulated genes
ISIORT	International Society of Intraoperative Radiotherapy
IRB	Institutional Review Board
mAb	monoclonal Antibody
MedDRA	Medical Dictionary for Regulatory Activities
MDT	Multi-Disciplinary Team
MDSC	Myeloid-Derived Suppressor Cells
MHC class1	Major Histocompatibility class I
NBS	Nijmegen Breakage Syndrome
NCI-CTCAE	National Cancer Institute - Common Terminology Criteria for Adverse Events
NIMP	Non-Investigational Medicinal Product
NST	No Special Type
ORR	Overall Response Rate

Abbreviation	Description
OS	Overall Survival
PBI	Partial Breast Irradiation
pCR	pathological complete response
PD-1	Programmed cell death receptor 1
PD-L1	Programmed cell death receptor ligand 1
PDX	patient derived xenograft
PFS	progression free survival
PS	Performance Status
RCB	Residual Cancer Burden
RECIST	Response Evaluation Criteria In Solid Tumours
RNA	Ribonucleic Acid
SAE	Serious Adverse Event
SAR	Serious Adverse Reaction
SBRT	Stereotactic Body Radiotherapy
SGOT	Serum Glutamic-OxaloaceticTtransaminase
SGPT	Serum Glutamic Pyruvic Transaminase
SmPC	Summary of Product Characteristics
SOC	System Organ Class
SPM	Second Primary Malignancy
STING	Stimulator of interferon genes
SUSAR	Suspected Unexpected Serious Adverse Reaction
TIL	Tumour infiltrating lymphocytes
TNBC	Triple Negative Breast Cancer
TNM	Tumour, Node, Metastases
Treg	Regulatory T cells
UAR	Unexpected Adverse Reaction
WBI	Whole Breast Irradiation

1. BACKGROUND AND SCIENTIFIC RATIONALE

1.1. Background and General Rationale

1.1.1. Why luminal B breast cancer?

Luminal breast cancer is characterised by a positive estrogen receptor (ER) status and categorised into two subclasses, A and B. Luminal B breast cancer has a higher tumour cell proliferation rate than luminal A breast cancer (1). Breast cancer-specific mortality rate is twice as high in luminal B subjects relative to luminal A subjects (16). In the last decades, major advances in systemic treatment were made for the HER2+ subgroup, however there have been less advances for the other subtypes. The rate of pathological complete response (pCR) is also markedly different according to the molecular subtype: 7,5% in luminal A, 15% in luminal B, 22% in luminal B/HER2 positive and 31% in triple negative breast cancer (TNBC) (2). With the exception of luminal A tumours, a correlation has been established between achieving a pCR and overall survival (17). Luminal B breast cancers who cannot achieve a pCR after neo-adjuvant treatment present a significant diminished event-free survival (HR=0.27). Ten year relapse-free survival in early breast cancer is worse for grade 3 luminal cancer (luminal B) than for grade 1-2 luminal cancer (HR=2.7) (5). There is thus a real actual need to focus innovative research on effective neo-adjuvant treatments for luminal B breast cancer. One of the suggested innovative strategies is exploring the use of neo-adjuvant immuno-radiation.

1.1.2. The immune response in luminal B breast cancer and the importance of priming the microenvironment

Recent research on immune response has drawn a correlation between genetic instability and the immune response. Mutational load and heterogeneity can drive the creation of novel breast cancer antigens. TNBC, HER2+ and luminal B breast cancers have a high mutational burden compared to luminal A breast cancer possibly immunogenic and suggesting that immunotherapeutic approaches may be effective in these breast cancer subtypes. The immune response of luminal B breast cancer, however, is different than the TNBC and HER2+ subtype because luminal B breast cancer is a less inflamed cancer type (6). Inflamed cancer types are characterised by the presence of tumour infiltrating lymphocytes (TILs), programmed cell death receptor ligand 1 (PD-L1) positivity of tumours or immune cells and high CD8+ T-cell density (18). Non-inflamed cancers, such as luminal B breast cancer, are also known as ‘immune cold tumours’.

1.1.2.1. *Single agent immunotherapy is probably ineffective in luminal B breast cancer*

Evidence in the metastatic setting suggests that a single agent immune checkpoint inhibitor targeting PD-1/PD-L1 is most effective in inflamed tumours . Non-inflamed tumours, such as the ‘cold’ luminal B breast cancer, are hence less capable of mounting an immune response with single agent immunotherapy.

Clinical data from recent trials support this hypothesis. KEYNOTE-028 is a “basket” trial that included several types of advanced cancers that express PD-L1 (7). Among 25 evaluable subjects (from a cohort of 261 breast cancer subjects) with *estrogen receptor–positive/HER2-negative* breast cancers that express PD-L1, the overall response rate (ORR) to pembrolizumab in monotherapy was only 12%. Similarly, the phase I JAVELIN trial revealed a very poor ORR of 2.8% in 72 subjects treated with avelumab (anti-PD-L1) in monotherapy for heavily pre-treated metastatic *luminal B breast* cancer subjects (9). On the contrary, Nanda et al. evaluated the activity of the anti-PD-1 antibody pembrolizumab as monotherapy for the treatment of previously treated, metastatic ‘immune hot’ *TNBC* and reported an overall

response rate of 18.5% (19). The anti-PD-L1 antibody atezolizumab demonstrated a similar overall response rate of 19% when given as monotherapy in previously treated metastatic TNBC. Hence, the response rate to treatment with single agent immunotherapy is clearly different between luminal B breast cancer and TNBC. There is thus a need to convert the non-inflamed luminal B breast cancer to an inflamed and immune-responsive tumour. Strategies to perform this conversion were termed 'priming' and are primarily directed to the tumour microenvironment.

1.1.2.2. *Priming of the immunologic response to increase efficacy of immunotherapy in luminal B breast cancer*

As discussed, luminal B breast cancer is of the non-inflamed type, contrary to TNBC and HER2+ breast cancer, they are unlikely to derive substantial benefit from single agent immunotherapy. For immunotherapy to be potent in luminal B breast cancer, a combination strategy with chemotherapy, a doublet immunotherapy or radiotherapy is necessary. The goal is to use these agents to prime the tumour microenvironment to induce an immune response.

The I-SPY 2 trial investigated the addition of pembrolizumab to standard neo-adjuvant chemotherapy (paclitaxel followed by doxorubicin and cyclophosphamide) for breast cancer (10,19). In luminal B breast cancer, a threefold increase in pCR rate was seen (30% vs 13%) with the combination compared to standard chemotherapy alone.

Strategies to further increase pCR and ORR in estrogen receptor–positive/HER2-negative breast cancers consist of using more effective treatments to further prime the microenvironment and inhibit immunosuppressive signals. These strategies include targeted therapy (anti-CD-73 for example) and radiation therapy. The priming strategy adopted in the present trial proposal will be discussed in more detail in section 1.2.

1.1.3. Why the neo-adjuvant setting

The neo-adjuvant setting is (by definition) the earliest disease stage available for treatment with systemic therapy and is hence optimally suited for treatments with immunotherapy. Pre-clinical models of breast cancer also support the superiority of neo-adjuvant immunotherapy compared to adjuvant immunotherapy (20).

An important benefit of the neo-adjuvant setting is the ability to measure pCR rate. A pCR is defined as the absence of invasive tumour cancer in the breast and in the axillary lymph nodes after completion of a neo-adjuvant treatment (21). Subjects who achieve a pCR have improved survival, and pCR rate has therefore been suggested as a potential surrogate endpoint for long term clinical benefit in aggressive breast cancer subtypes. However, pCR has not yet been validated as a surrogate endpoint for improved EFS (Event Free Survival) and Overall Survival (OS) at the trial level (22).

Neo-adjuvant treatment followed by complete surgical resection of the tumour allows a rapid and thorough assessment of response with the possibility of performing a wide range of translational research on the surgical specimen. This advantage is typically unavailable in the metastatic setting.

Another argument in favour of the neo-adjuvant setting is that our trial proposes to prime the cancer microenvironment with three fractions of Stereotactic Body Radiotherapy (SBRT). The non-metastatic setting is ideal to convert the tumour into an in-situ, individualised vaccine using a combination of radiotherapy and immunotherapy (23). Hypothetically, the vaccine-effect could treat existing or future micro metastatic sites, hence protecting the subjects against metastasis.

1.2. In-depth rationale

1.2.1. Individual treatment components of the trial

1.2.1.1. *Targeting PD-L1 with durvalumab (MEDI4736)*

Durvalumab is a human immunoglobulin G1 kappa (IgG1κ) monoclonal antibody that targets PD-L1. Durvalumab blocks the interaction of PD-L1 (but not PD-L2) with PD-1 on T cells and CD8+ immune cells and is engineered to reduce antibody-dependent cell-mediated cytotoxicity (ADCC).

Safety data are available from several trials evaluating durvalumab in monotherapy in solid tumours (24,25) and in combination with the anti-CTLA-4, tremelimumab (26) or with gefitinib, or osimertinib and also with the anti-CD73 MEDI9447 (oleclumab). For the combination with MEDI9447, no DLTs were reported (27).

Durvalumab was first approved in the US on 01 May 2017 for the treatment of patients with locally advanced or metastatic urothelial carcinoma (UC). On 19 February 2021, and after the consultation with the FDA, the indication has been voluntarily withdrawn in the US. The voluntary withdrawal was not due to any safety concerns or new safety data.

GeparNuevo is a randomised phase II trial investigating the addition of durvalumab to a taxanes-anthracycline chemotherapy in triple negative breast cancer (TNBC). The study included a ‘window’ cohort in which durvalumab was given for a window of 2 weeks before chemotherapy, priming the immune response first. Safety data from this trial show that the addition of durvalumab did not result in increased toxicity (28). After enrolling 174 subjects, 27.6% subjects presented at least one immune averse effect of special interest. For the primary endpoint, pathologic complete response rates were 53.4% in the durvalumab arm vs 44.2% for the chemotherapy alone arm. The difference was not statistically significant, however subgroup analysis of pCR suggested that subjects with any level of tumour-infiltrating lymphocyte expression benefited from durvalumab and the best response rates were found when durvalumab was given as a window of 2 weeks before chemotherapy (28,29).

The PACIFIC trial investigated the use of durvalumab in subjects with stage III, unresectable non-small cell lung cancer (NSCLC) who did not have disease progression after concurrent chemoradiotherapy (30). This phase III study showed that durvalumab significantly prolonged overall survival: the 24-month overall survival rate was 66.3% in the durvalumab group and 55.6% in the placebo group (HR 0.68; 99.73% CI, 0.47 to 0.997; P=0.0025). PFS was also significantly extended in the durvalumab group: the median time to death or distant metastasis was 28.3 months in the durvalumab group and 16.2 months in the placebo group. A total of 30.5% of the subjects in the durvalumab group and 26.1% of those in the placebo group had grade 3 or 4 adverse events of any cause; 15.4% and 9.8% of the subjects, respectively, discontinued the trial regimen because of adverse events (30).

On 16 February 2018, durvalumab was approved in the US for the treatment of patients with unresectable Stage III non-small cell lung cancer (NSCLC). On 21 September 2018, durvalumab was approved in the EU for the treatment of locally advanced, unresectable NSCLC in adults whose tumours express PD-L1 on ≥1% of tumour cells and whose disease has not progressed following platinum-based chemoradiation therapy. On 27 August 2020, durvalumab was approved for the first-line treatment of patients with extensive-stage small cell lung cancer (ES-SCLC) in combination with etoposide and either carboplatin or cisplatin in the EU. As of 12 July 2021, durvalumab is approved in more than 70 countries for UC, 59 countries for NSCLC, and 6 countries for ES-SCLC.

Update after ESMO 2023 (20 October 2023):

The benefit of the addition of anti-PD-(L)1 to neo-adjuvant chemotherapy in early-stage luminal B BC was investigated in the KEYNOTE-756 trial (using pembrolizumab) and CheckMate-7FL (using nivolumab). Pathological complete response (pCR) is the primary endpoint of both trials. First pCR results of both trials were presented at ESMO 2023 on 20 October 2023: an absolute increase in pCR of respectively 8.5% and 10.5% was demonstrated with the addition of anti-PD-(L)1 (31,32). The rate of RCB 0/1 was a secondary endpoint. In CheckMate-7FL the rate of RCB 0/1 was increased with 9.2% with the addition of nivolumab. The odds ratio of the increase in pCR was 2.05 whereas the odds ratio of the increase in RCB 0/1 rate was 1.65.

1.2.1.2. *Targeting CD73 with oleclumab (MEDI9447)*

A total of 667 subjects have been treated with oleclumab across 12 clinical studies that are either completed or ongoing: Studies D6070C00006, D6070C00001, D6070C00004, D6070C00005, D6185C00001 (HUDSON), D9108C00001 (COAST), D9108C00002 (NeoCOAST), D933IC00001 (MAGELLAN)), D8730C00001, D8731C00001, D910CC00001 (COLUMBIA 1), and D933LC00001 (BEGONIA). D6070C00006 was completed in February 2020, oleclumab was administered as a single agent in 42 adult subjects with selected advanced solid tumours. A total of 40 of 42 subjects (95.2%) experienced treatment emergent adverse events (TEAE) and 23 subjects (54.8%) experienced treatment-related TEAE. The most frequently reported treatment related TEAE (in ≥ 5% of total subjects) were fatigue (16.7%), anemia and nausea (9.5% each).

Results from COAST (D9108C00001) were published in 2022 (33). In this trial, patients with unresectable stage III NSCLC (non-small cell lung cancer) were randomised to durvalumab alone or combined with oleclumab or anti-NKG2A mAb monalizumab as consolidation therapy after concomitant chemoradiotherapy. The trial demonstrated that the addition of oleclumab or monalizumab improved ORR and PFS over durvalumab alone. Safety was similar across arms with no new safety signals identified.

Additionally, results from the NeoCOAST trial (NCT03794544), presented as conference proceeding at AACR 2022, showed that the combination of immunotherapy with durvalumab and other novel agents (including oleclumab) outperformed durvalumab alone in the neoadjuvant setting for patients with early-stage NSCLC (34). In particular, the addition of oleclumab to durvalumab showed improved major pathological response (defined as ≤10% residual viable tumor cells at tumor site and nodes, at surgery) and pathological complete response, compared to durvalumab alone.

Interim safety data on 113 subjects and efficacy data for 96 subjects (of whom 31 with TNBC) enrolled in a phase I/Ib trial of CPI-444, an A2A antagonist, +/- atezolizumab (NCT02655822) in advanced solid tumours were presented at AACR 2017. Fifty-six percent were resistant or refractory to prior treatment with anti-PD-(L)1 antibodies. Disease control in TNBC was achieved in 41% of those receiving CPI-444 as single agent (n=17) and in 21% of those receiving the combination (n=14) for an overall disease control rate of 32% in 31 subjects. CPI-444 was well tolerated with no grade 3 or 4 adverse events with single agent CPI-444 (35).

In this trial, oleclumab (MEDI9447) a human immunoglobulin G1 lambda (IgG1λ) monoclonal antibody (mAb) that selectively binds to and inhibits the ectonucleotidase activity of CD73 will be evaluated to inhibit the production of adenosine and organic phosphate from adenosine monophosphate (AMP) by CD73.

1.2.1.3. *Pre-operative radiation therapy to the primary breast cancer*

Recent technological advancements in the field of radiation therapy opened the way to the precise delivery of high radiation doses in very few treatment fractions. Such techniques are called stereotactic body radiation therapy (SBRT). The technique can be used to selectively target the breast tumour without irradiating the whole breast and hence significantly reducing toxicity.

Bondiau et al. conducted a non-randomised phase 1 trial of neo-adjuvant SBRT to the primary breast tumour combined with 3 cycles of docetaxel followed by 3 cycles of FEC (Fluorouracil-Epirubicin-Cyclophosphamide) (36). The study revealed an overall pCR rate of 36% however no data was reported on pCR rate according to breast cancer subtypes. The trial demonstrated that SBRT could be given safely with little toxicity and the authors recommend 3 fractions of 8.5 Gy given in three consecutive days as treatment schedule for a future phase 2 trial. This dose is only a boost dose, all subjects in this trial also received classic adjuvant radiotherapy to the breast and lymph nodes after completing surgery.

The 'ABLATIVE' phase 1 trial examined the delivery of 20 Gy as a single fraction using an SBRT technique without chemotherapy or other treatments on small luminal A breast cancers (37). Surgery is planned more than 6 months after SBRT. The primary endpoint is pCR and results revealed little toxicity and encouraging pCR rates (42% pCR) (38). This trial uses a partial breast irradiation (PBI) approach, without whole breast radiotherapy (also not after surgery). PBI is a validated treatment approach for small luminal A cancers, the treatment is however not validated in non-luminal-A breast cancers, for these subjects whole breast radiotherapy remains the standard of care treatment (39).

Neo-adjuvant whole-breast radiotherapy has also been investigated and is deemed a promising approach with potential gains in disease-free survival (40). There is a renewed interest in pre-operative (neo-adjuvant) breast radiotherapy in recent years and several trials have been launched in this setting (41). Clinicaltrials.gov was accessed on 20/05/2018 to retrieve a list of trials investigating the use of pre-operative radiotherapy in breast cancer (Table 1).

Table 1: *ongoing trials investigating pre-operative radiotherapy in non-metastatic breast cancer (as a boost and/or as a full course radiotherapy), clinicaltrials.gov accessed on 20/05/2018.*

Trial identification	Trial name	Type of study	Study Hypothesis
**NCT02316561**	ABLATIVE	Non-randomised	Investigate the feasibility of a preoperative, single dose, ablative partial breast radiation treatment in subjects with early-stage breast cancer.
**NCT02806258**	NEOAPBI 01	Phase II,<br>Randomised	Primary chemotherapy versus primary chemotherapy and sequential accelerated partial breast irradiation (APBI).
**NCT02913729**	PAPBI-2	Phase III randomised	Preoperative versus postoperative APBI.
**NCT02941835**	PROBI	Phase I/II, non-randomised	Preoperative whole breast radiotherapy
**NCT03366844**	/	Phase I	Assessment of the safety and tolerability of pembrolizumab combined with a tumour RT boost before subjects undergo standard treatment that can consist of one or

			more of the following: breast-conserving surgery, radiation to the entire breast/chest wall after surgery, and chemotherapy.
**NCT03109080**	RadioPARP	Phase I	Preoperative or post-operative radiation therapy with concurrent olaparib.
**NCT02771938**	PRADA	Non-randomised	Preoperative radiation therapy: mastectomy and DIEP flap reconstruction 2-6 weeks following completion.

1.2.2. Rationale of combining treatments and priming of the cancer microenvironment

As previously discussed, luminal B breast cancer is a non-inflamed, immune cold tumour that is likely unresponsive to a single agent immunotherapy. The microenvironment needs to be primed to stimulate immune response. The priming in the present trial proposal is achieved by the combination of anti-PD-L1 with chemotherapy, anti-CD-73 and radiation therapy.

An example of a trial testing priming strategies in breast cancer is the TONIC trial: a phase 2 trial in metastatic TNBC that evaluates different immune priming strategies before starting checkpoint blockade (42). Subjects were randomised to priming with radiation therapy, low dose cyclophosphamide, doxorubicin, cisplatin and no priming. As reported at ASCO 2018, the first results of the trial revealed that tumour response without priming was lower than with priming; hence providing proof of concept that immune priming can initiate an anti-tumour immune response, which in turn is boosted using immune checkpoint blockade.

Another successful example of priming of immunotherapy in TNBC with chemotherapy has been shown in the IMpassion130 trial (43). In this phase 3 trial, metastatic or inoperable locally advanced TNBC subjects with no prior therapy for advanced disease were randomised 1:1 to atezolizumab (an anti-PD-L1) plus nab-paclitaxel or placebo plus nab-paclitaxel. The aim of the trial was to prove superiority of the experimental arm regarding PFS and OS in the ITT and PD-L1 positive populations. Four-hundred fifty-one subjects were randomised to each arm, stratified by prior taxane use in the (neo)adjuvant setting (yes vs no), presence of liver metastases (yes vs no) and PD-L1 status on tumour-infiltrating immune cells (positive [≥1%] vs negative [<1%]). Baseline characteristics were well balanced between treatment arms, with 51% of subjects in each arm previously exposed to a taxane, 28% in the experimental arm vs 26% in the control arm with liver metastases and 41% of subjects in each arm with tumours with PD-L1 positivity. In this primary analysis, the PFS in the ITT population was statistically superior in the experimental arm vs the control arm, with a median PFS of 7.2 months vs 5.5 months, respectively (stratified HR = 0.8 [95% CI: 0.69 to 0.92] p = 0.0025). This benefit was more pronounced in the pre-specified analysis of the PD-L1 positive population, with a median PFS of 7.5 months vs 5 months in the experimental vs control arm, respectively (stratified HR = 0.62 [95% CI: 0.49 to 0.78] p < 0.0001), with no signs of other relevant subgroups having detrimental effect of the addition of atezolizumab. Though the significance boundary for OS analysis was not reached in the ITT population, it was numerically higher in the experimental arm, with subjects having a median OS of 21.3 months, compared to subjects with a median of 17.6 months in the control arm. Furthermore, this numerical difference increased in the PD-L1 positive population, where subjects had a median OS of 25 months vs 15.5 months in the experimental and control arm, respectively. ORR rates were better with the addition of atezolizumab for both the ITT and PD-L1 positive population, and the toxicity profile was comparable to previous reports, with no new safety concerns, as all serious AEs of any grade were 23% in the experimental arm vs 18% in the control arm. Importantly, immune-related serious AEs, such as hypothyroidism, were 17% with atezolizumab vs 4% with placebo, and

pneumonitis was infrequent, in 3% of subjects on atezolizumab vs < 1% in subjects on placebo.

In KEYNOTE-522, a phase 3, placebo-controlled, randomized trial, patients with stage II or III TNBC were assigned 2:1 to neoadjuvant pembrolizumab/chemotherapy or placebo/chemotherapy, followed by adjuvant pembrolizumab or placebo to complete 1 year of treatment (44). The co-primary endpoints were pCR at surgery and event-free survival. At the first interim analysis, amongst 602 patients that underwent randomization, pCR rates were statistically superior with pembrolizumab/chemotherapy compared to placebo/chemotherapy (64.8% vs 51.2%; p<0.001). At fourth planned interim analysis after randomization of 1174 subjects and with a median follow-up of 39.1 months, the estimated event-free survival at 36 months was 84.5% (95% CI 72.2-80.7) in the pembrolizumab-chemotherapy group, as compared with 76.8% (95% CI 0.48-0.82) p<0.001 in the placebo-chemotherapy group (45). Safety profile was consistent with that of early-phase trials.

In the phase II adaptively randomized I-SPY2 trial, adding neoadjuvant pembrolizumab to chemotherapy has also been shown beneficial amongst patients with HER2-negative, stage II or III breast cancer (10). With pCR as its primary endpoint, 250 patients randomized, and 3 different biomarkers signatures studied, the addition of pembrolizumab increased the rates of pCR, compared to chemotherapy alone, from 22% to 60% between patients with TNBC, 13% to 30% between patients with HR-positive/HER2-negative patients, and from 17% to 44% between all HER2-negative patients. Patients who achieved pCR had better event free-survival rates than non-pCR ones. In this sense, priming of immunotherapy with chemotherapy clearly holds promise also in the setting of early luminal breast cancer.

1.2.2.1. *Synergism between durvalumab and oleclumab*

In mouse models, the potential of adenosine targeting treatment was enhanced when combined with other immunomodulatory treatment as ICB [43].The combination of ICB targeting PD-1 with an anti-CD73, MEDI9447(oleclumab) already showed synergistic activity in preclinical models (46).

Subjects with refractory advanced pancreatic or colorectal cancer were enrolled in a phase I dose escalation and expansion trial for treatment with oleclumab or oleclumab plus durvalumab. For all subjects treated with oleclumab, sustained decrease in free soluble CD73 and CD73 on peripheral T cells was demonstrated across all doses, which was consistent with the proposed mechanism of action. No DLTs were observed. The highest dose of the combo was subsequently selected for expansion in 42 subjects, with partial responses observed in 2 and stable disease response observed in 5 subjects, with a high duration of treatment for those experiencing disease control (up to 322 days). Objective response was 4.8% (95% CI 0.6%-16.2%). The most common treatment related AEs in combo expansion were diarrhea (8.7%), pyrexia (8.7%), fatigue (6.5%), and increase in ALT (6.5%), AST (6.5%), and ALP (6.5%) (47).

Results from COAST (D9108C00001) were published in 2022 (33). In this trial, patients with unresectable stage III NSCLC (non-small cell lung cancer) were randomised to durvalumab alone or combined with oleclumab or anti-NKG2A mAb monalizumab as consolidation therapy after concomitant chemoradiotherapy. The trial demonstrated that the addition of oleclumab or monalizumab improved ORR and PFS over durvalumab alone. Safety was similar across arms with no new safety signals identified.

1.2.2.2. *Rationale for combining durvalumab/oleclumab with chemotherapy*

The chemotherapy backbone of the present trial proposal consists of paclitaxel and dose-dense doxorubicin-cyclophosphamide (ddAC). This chemotherapy is the current standard of care in the neo-adjuvant setting for luminal B breast cancer (48).

There is emerging evidence of synergy between immunotherapy and chemotherapy. For instance, myeloid-derived suppressor cells (MDSC) accumulate in the tumour microenvironment and are associated with immune suppression. Docetaxel has been shown to possess immunomodulating properties by selectively suppressing MDSC (49). Anthracyclines have been shown to be capable of inducing immunogenic cell death (50).

Furthermore, treatment with anthracyclines, paclitaxel and platinum induced up-regulation of CD73 in human BC cells (51,52). Targeting the CD73 pathway to relieve the adenosine-mediated immunosuppression seems a promising therapeutic strategy in breast cancer.

1.2.2.3. *Synergism between SBRT and durvalumab*

In recent years, an impressive amount of clinical trials and animal studies have described the synergistic effects on local and distant tumour control of the combination of radiation therapy with immunotherapy (12,13,53–55). Radiosensitising immunotherapy is considered a potential curative therapeutic modality, akin to radiosensitising chemotherapy. Radiation induces changes to the tumour cell immunophenotype by a variety of mechanisms (56,57) and enhances cross-presentation of tumour antigens that can induce abscopal effects outside of the radiation field, radiation thereby acting as an in situ anti-tumour vaccine (58). Radiation acts synergistically with PD-1/PD-L1 blockade by upregulating MHC class 1 (major histocompatibility class I) (59) and the programmed cell death receptor FAS on tumour cells (60), therefore increasing T-cell susceptibility to T-cell mediated cytotoxicity in presence of anti-PD-L1 such as durvalumab.

Recent pivotal work by Vanpouille-Box et al. showed that the DNA exonuclease Trex1 is induced by radiation fraction doses above 12-18 Gy. Trex1 attenuates cancer cells immunogenicity by degrading cytosolic DNA. Cytosolic DNA is highly immunogenic by stimulating the secretion of interferon-β by cancer cells following activation of the DNA sensor cGAS (cyclic GMP-AMP synthase) and its downstream effector STING (Stimulator of interferon genes). Repeated fractionated radiation at lower doses, however, does not induce Trex1 and amplifies interferon-β production, resulting in priming of CD8+ T cells that can induce a systemic tumour rejection, particularly when combined with immune checkpoint blockade (61). The highest induction of cytoplasmic double stranded DNA, interferon-β and CD8+ T cells was seen with a fractionation schedule of 3 fractions of 8 Gy given daily (not spaced over 5 days).

An online search on clinicaltrials.gov was performed to identify ongoing clinical studies that examine the combination of SBRT and immunotherapy in breast cancer. One trial (NCT03366844) examining the safety of combining pembrolizumab and radiation in the neo-adjuvant setting (study currently recruiting 2018) was identified.

Table 2: Ongoing studies combining SBRT and immunotherapy in breast cancer in 2018.

Trial	Design	Setting	SBRT dose<br>Immunotherapy	Primary endpoint
NCT03366844	Phase I	Safety and tolerability assessment of pembrolizumab combined with a tumour RT boost	3x8 Gy	Safety

		before subjects undergo standard treatment that can consist of: breast-conserving surgery, radiation to the entire breast/chest wall after surgery, and chemotherapy. TNBC and HR+/HER2- tumours.		
NCT01421017	Phase 1-2<br>Non-randomised	Breast cancer with skin metastasis or chest wall recurrence	5X6 Gy<br>Imiquimod cream + Cyclophosphamide	Systemic tumour response rate
NCT01862900	Phase 1-2<br>Non-randomised	Metastatic breast cancer	Variable doses<br>OX-40	DLT of SBRT dose
TONIC	Phase 2<br>Non-randomised	Metastatic TNBC	1x20Gy<br>Nivolumab	PFS
NCT02538471	Phase 1<br>Non-randomised	Metastatic breast cancer	3x7,5 Gy<br>TGFβ blockade	Safety
BOSTON II	Phase 1<br>Non-randomised	Oligometastatic breast cancer	1x20Gy<br>Pembrolizumab	Safety
NCT02730130	Phase 2<br>Non-randomised	Locally recurrent or metastatic TNBC	5x6Gy<br>Pembrolizumab	Response rate in unirradiated lesions

Abbreviations: DLT, dose limiting toxicity; SBRT, stereotactic body radiotherapy; PFS, progressive-free survival; TNBC, triple negative breast cancer.

1.2.2.4. *Synergism between SBRT and oleclumab*

Radiation therapy has pre-clinically been shown to act synergistically with anti-CD-73 through the prevention of adenosine-mediated immunosuppression (62). Recent research suggest that CD73 may be a radiation-induced checkpoint, and that CD73 blockade in combination with radiotherapy and immune checkpoint blockade might improve patient response to therapy (63). Pre-clinical data show that the combination of CD73 inhibition and radiotherapy has the following effect : 1) enhancement of the radiation-induced activation of the antitumour immune response, 2) restriction of the immunosuppressive action of CD39/CD73 on circulating immune cells and 3) attenuation of adverse late effects of radiotherapy through inhibition of fibrosis, which could be especially important to reduce radiation damage of the lungs (63–65).

1.2.3. Rationale for SBRT dose and SBRT timing

1.2.3.1. *Rationale for the choice of SBRT dose fractionation in combination with immunotherapy*

Selecting the correct SBRT dose and fractionation is paramount to obtain an optimal synergistic effect between SBRT and immunotherapy. Several studies revealed that limiting the number of treatment fractions to a maximum of 3 and giving a dose of 5-20 Gy per fraction induces better results than classical fractionation schemes of 1.8-2.2 Gy per fraction (57,66). Dewan et al. examined the effect of fractionation on TSA mouse breast carcinoma cells when

combined with 9H10 monoclonal antibody against CTLA-4 (66). Three fractionation schedules were tested: 1x20 Gy, 3x8 Gy, 5x6 Gy. Maximal abscopal effect was observed with the 3x8 Gy schedule. In addition, recent work by Vanpouille-Box et al., as discussed previously, demonstrated optimal immunogenic effects with a fractionation schedule of 3 fractions of 8 Gy given daily (not spaced over 5 days).

Based on the results of the Bondiau phase 1 trial, 3x8.5 Gy is recommended as SBRT dose to the primary breast tumour in the neo-adjuvant setting in combination with chemotherapy (this trial was performed without immunotherapy). Furthermore, after the pre-operative tumour treatment with 3x8.5 Gy, it remains safe to post-operatively treat the whole breast (or the chest wall after mastectomy) and the lymph node regions with classic adjuvant radiation therapy doses (25x2 Gy) as demonstrated in the Bondiau phase 1 trial (36). However, the boost dose will be omitted as the pre-operative SBRT dose will be considered as an anticipated boost.

Taking all this information on dose fractionation together, a daily fractionation study of 3 fractions of 8 Gy to the primary breast cancer will be given.

1.2.3.2. *Rationale for the timing of the combination of SBRT, immunotherapy and chemotherapy*

1.2.3.2.1 Rationale behind the overall timing of the different treatment components

Clinical studies have shown that response to SBRT and checkpoint blockade is markedly slower than response to other cancer treatments. The ABLATIVE trial that treats early luminal A breast cancers with 1 fraction of 20 Gy in the neo-adjuvant setting (without immunotherapy) recently requested a protocol amendment to increase the time between SBRT and surgery to a minimum of 6 months. This decision was based on an ad interim analysis revealing the importance of allowing sufficient time between SBRT and measurement of pathological response (67). Results of clinical trials with checkpoint blockade inhibitors in other cancers have observed a similar pattern, hence recommending to allow enough time between start of treatment and response evaluation (68). In our trial design, a minimum of 16 weeks between SBRT and surgery of the primary breast cancer was planned. The interval between the start of immunotherapy and surgery will be minimally 21 weeks. The choice to start with weekly paclitaxel instead of the 4 cycles of AC was made to allow enough time between the SBRT and surgery for a tumour response to develop, because SBRT cannot be given concomitantly with AC chemotherapy for reasons of toxicity.

1.2.3.2.2 Rationale behind the exact timing of SBRT and immunotherapy

Current pre-clinical and clinical evidence suggest that the synergy between radiation therapy and immunotherapy is optimal if radiation therapy is given *after* anti PD-L1 treatment and/or *concomitantly* (the same day) (69–71). A large part of the priming effect of radiation therapy on the tumour microenvironment might be lost if radiation is given before immunotherapy (a 1-week interval is enough to lose the effect). An optimal interaction is expected just at that moment when radiotherapy temporarily induces surface ligands on the cancer cell increasing its vulnerability to T-cell attacks. Sundahl et al. conducted a phase 1 randomised trial in urothelial carcinoma in which radiation therapy was either given prior to the first cycle of pembrolizumab or prior to the third cycle of pembrolizumab (72). Response rate in the arm where radiation therapy was given prior to the third cycle of pembrolizumab was significantly better than when radiation therapy was given before the first cycle of pembrolizumab. In the ongoing NCT03366844 clinical trial, radiation therapy is given concomitantly with the second cycle of pembrolizumab in the neo-adjuvant setting for TNBC or HR+/HER2- tumours.

Taking this data into consideration, our choice is to give SBRT in week 5, concomitantly with the second cycle of the anti-PD-L1 durvalumab. More specifically, 3 fractions of SBRT will be given at the minimum in 3 days and at the maximum in 6 days. The third fraction of SBRT will

be given the day before or on the same day just before the 5th administration of paclitaxel, the 2nd administration of durvalumab and the 3rd administration of oleclumab.

1.2.3.3. *Impact of pre-operative SBRT on standard adjuvant radiotherapy treatment*

Time delay between pre-operative SBRT as an anticipated boost and whole breast irradiation

Benefit and indication of the boost in standard breast radiation therapy
In standard postoperative breast radiation therapy, the boost is a local dose escalation to the primary tumour bed as the region with the highest risk of recurrence. The European Organisation for Research and Treatment of Cancer (EORTC 10882-22881) phase 3 boost trial demonstrated in 5569 subjects that a boost improved local control, with the largest absolute benefit in young subjects, however without an effect on overall survival (73). The 20-year cumulative incidence of ipsilateral breast tumour recurrence was 16,4% in the no boost group versus 12,0% in the boost group. In this trial, a 16 Gy boost was administered sequentially immediately after whole breast irradiation (WBI).

Pre-operative boost to the primary tumour bed in combination with chemotherapy
Delivery of the boost before whole breast irradiation (WBI) is also known as an *anticipated tumour bed boost*. As discussed in the above sections, Bondiau et al. performed a phase 1 trial in which SBRT to the tumour bed was given as an anticipated boost in combination with chemotherapy (36). The conclusion of this trial was that 3x8.5 Gy is a safe dose permitting post-operative treatment with standard radiation therapy omitting the boost.

Influence of the time delay between boost and whole breast irradiation: evidence from the literature
The time between an anticipated boost and postoperative WBI depends mainly on whether or not chemotherapy is given in between both. Most of the research in the field of anticipated tumour bed boost was performed delivering the boost intra-operatively using an IOERT technique (intraoperative electron radiation therapy). In a cohort of 770 subjects treated with IOERT as a boost, Kaiser et al. demonstrated high local control rates for subjects in all known risk categories (74). In this study, 22% of subjects received chemotherapy between the boost and the WBI. The time between boost and WBI (analysed as <70 days vs. ≥70 days) could not be identified as a risk factor for an increase in local recurrence. These results are in line with a pooled analysis by the European Group of the International Society of Intraoperative Radiotherapy (ISIORT) in a cohort of 1109 subjects of whom 35.2% received adjuvant chemotherapy between the boost and WBI (75).

Adaptations made to the postoperative whole breast irradiation treatment
As discussed above, in this present trial we plan to deliver pre-operatively 24 Gy in 3 fractions to the tumour with an SBRT technique, which we consider as an anticipated boost. This dose is higher than the standard boost dose of 16 Gy in 8 fractions. Expressed in equivalent dose of 2 Gy per fraction (EQD2) with an α/β of 10 the difference amounts to 20 Gy $EQD2_{10}$ (3x8Gy ≈ 36 Gy $EQD2_{10}$ and 8x2 Gy ≈ 16 Gy $EQD2_{10}$). Two important factors should be considered when assessing the possible impact of this clear dose difference: 1) a SBRT technique will be used to deliver the dose pre-operatively allowing delivery of a very conformal dose to the tumour with a rapid dose fall-off in the surrounding tissues and 2) the surgeon will remove the primary tumour and the surrounding breast tissue, thereby reducing the total dose delivered to the remaining breast at the moment of postoperative WBI as the high-dose volume will be excised. However, to further compensate for the remaining difference in dose, a technique of inverted simultaneous integrated boost can be used for the postoperative WBI. This approach enables delivery of a planned under dosage of the primary tumour bed during WBI. A similar technique was described by Leonardi et al. when treating local relapses with WBI after a

previous partial breast irradiation with IOERT (76). The postoperative radiation therapy will be performed as standard of care, with the only difference that the boost will be omitted (given the boost was already given pre-operatively). The radiation oncologist treating the patient in the post-operative setting may choose to lower the dose in the tumour bed if he or she wishes, but this is not mandatory.

Possible impact of pre-operative SBRT on breast cosmesis
Bondiau et al. evaluated chronic toxicity every 6 months after treatment with pre-operative SBRT 3x8.5 Gy to the primary tumour in combination with chemotherapy and followed by standard post-operative radiation therapy (excluding the boost). This study did not identify treatment-specific toxicities with a median follow-up time of 30 months (36). Evaluated chronic toxicity parameters in the Bondiau study were fibrosis, chest wall pain, breast pain and undesirable cosmesis. No other details were mentioned about cosmetic outcome. In the Neo-checkray study, cosmetic outcome will be scored and breast photography will be performed to collect more cosmetic data. As mentioned in the above paragraph, in the Neo-checkray study and in the Bondiau study, the surgeon removes the primary tumour and the surrounding breast tissue, thereby reducing the total final radiation dose delivered to the breast. Hence, it is difficult to estimate the individual final radiation dose because it can vary depending on the lumpectomy size and the resection margins. It is therefore possible that some patients would receive in part of the breast the equivalent of a high boost dose whereas other patients would receive the equivalent of a normal or a low boost dose. Cosmetic effect of a high boost dose is available from the “Young boost trial” (77). In this trial, patients were randomized between a 26 Gy (13x2 Gy) boost dose and a 16 Gy (8x2 Gy) boost dose. At four years, cosmetic outcome was significantly better in the 16 Gy boost dose group than the 26 Gy boost group: 30% vs 40% of moderate or severe fibrosis. Patient cosmetic outcome was graded as ‘satisfied’ in 65% vs 55% of patients.

1.2.4. Rationale for giving radiation therapy in the three treatment arms

Radiation therapy is an effective localised treatment. Given the choice of RCB (Residual Cancer Burden) 0-1 as primary endpoint of the current trial, we suggest to deliver radiation therapy to the three treatment arms in order to avoid an imbalance in local treatment between the treatment arms. This allows singling out the effect of durvalumab and oleclumab without the results being disbalanced by radiation therapy.

1.2.5. Are there similar ongoing trials?

As mentioned above, the phase I clinical trial NCT03366844 is testing the safety of the combination of 3x8 Gy radiation therapy with pembrolizumab in the neo-adjuvant setting for TNBC and ER+HER2- breast cancer. The primary endpoint of this trial is safety and feasibility. Essential differences with our current study proposal are 1) no combination with chemotherapy; 2) no addition of an anti-CD73 and 3) a very short time interval of only 6 weeks between the trial onset and surgery. Whilst NCT03366844 has the merit to be the first to test this approach in the neo-adjuvant setting, it should be emphasised that the combination of this exact dose of radiation therapy (3x8 Gy) and PD-1/PD-L1 blockade has already largely been documented in many other trials and is deemed safe (78) (also discussed in section 1.3.1.2). It is unlikely that a different set of safety data would emerge from irradiation of breast tissue in comparison to irradiation of lung, liver, brain and pelvic areas concomitantly with PD-1/PD-L1 blockade. Secondly, the interval of 6 weeks between trial onset and surgery is likely too short to expect significant responses to radiation and immunotherapy, since both treatments are known to produce slow responses, unlike chemotherapy.

1.3. Potential Risks and Benefits

1.3.1. Risks

1.3.1.1. *Individual treatment components*

Safety of the individual treatment components are documented in section 6 and toxicity management and dose adjustments are discussed in section 7. Risks related to durvalumab and oleclumab are discussed in the latest investigator brochure (IB). Information on the management of immunotherapy related toxicities is discussed in the toxicity and management guidelines (Appendix 1).

1.3.1.2. *Risks associated with the combination of treatment components*

1.3.1.2.1. Known risks at time of launching of the Neo-Checkray safety run-in

Several clinical trials have reported that the combination of **radiation therapy with PD-1/PD-L1 blockade** is safe and well tolerated (78–80). Luke et al, for example, evaluated the toxicity of combining high dose SBRT (3x15 Gy; 5x10 Gy or 3x10 Gy) with pembrolizumab 200 mg IV q3w and found that the treatment was very well tolerated (78). In our present study proposal, we would not suggest high dose SBRT but limit the dose to 3x8 Gy, therefore further reducing the risk of toxicity. The investigator's brochure of durvalumab (version 14, February 2019) does not mention toxicity induced by the combination of radiation therapy and durvalumab. The only exception is a moderate increase in low-grade pneumonitis in the PACIFIC trial in subjects with locally advanced, unresectable non-small cell lung cancer (81). In this trial there was no increase of high-grade pneumonitis. Important to mention however is that in the PACIFIC trial the radiation therapy was non-SBRT using larger treatment fields involving nodal radiation and conventional fractionation. Durvalumab was giving sequentially after treatment with chemoradiation.

The combination of **radiation therapy and docetaxel** in the neo-adjuvant setting was safely tested in a phase 1 trial as mentioned above (36). The choice of combining SBRT with docetaxel was made to avoid the toxicity of combining high dose radiation therapy with anthracyclines such as doxorubicin. We will adopt the same strategy in our current trial by combining SBRT with weekly paclitaxel and so avoiding the combination of radiation therapy with doxorubicin. Further details about the combination of radiation therapy and docetaxel, including radiation recall dermatitis, can be found in section 6.11.3.6.

The combination of **radiation therapy and oleclumab** has been tested in different pre-clinical studies (82,83). This data show that oleclumab can attenuate adverse late effects of radiotherapy through inhibition of fibrosis, which could be especially important to reduce radiation damage to the lungs. Adenosine has been shown to amplify radiation-induced lung fibrosis and radiation-induced skin fibrosis (64,84,85). Oleclumab, an anti-CD73, is a potent blocker of the degradation of adenosine monophosphate into adenosine.

The COAST trial (D9108C00001) was presented at ESMO 2021 (86). In this trial, subjects with unresectable stage III NSCLC (non-small cell lung cancer) were randomised to durvalumab alone or combined with oleclumab or anti-NKG2A mAb monalizumab as consolidation therapy after concomitant chemoradiotherapy. The trial demonstrated safety was similar across arms with no new safety signals identified. Combined rates of pneumonitis and radiation pneumonitis of any grade were 21.2% with durvalumab, 28.8% with

durvalumab+oleclumab and 21.3% with durvalumab+monalizumab, with grade ≥3 events in 3.0%, 3.4% and 1.6%. However, it is important to point out that these subjects received radiation therapy for lung cancer, which results in significantly higher lung radiation doses than radiation therapy for breast cancer (especially in the case of SBRT on the primary breast cancer).

The combination of **oleclumab, durvalumab, carboplatin, and paclitaxel** was tested in the SYNERGY trial (EudraCT number 2017-004651-23, Sponsor's Protocol Code Number IJB-SYNERGY-012017). However, no carboplatin will be used in the Neo-CheckRay trial. The SYNERGY trial consisted of a phase I safety study followed by a phase II study. The phase I part of the SYNERGY protocol was completed and the recruitment for the phase II part was stopped on 22 June 2021, following the IDMC (Independent Data Monitoring Committee) recommendation after evaluating the results of futility interim analysis in which the pre-specified boundary for futility was crossed. No safety concerns were identified for the arm with oleclumab (87).

The **effect of the combination of radiation therapy and durvalumab/oleclumab on breast cosmesis**. Considerations on breast cosmesis of pre-operative SBRT on the tumour concomitant with chemotherapy and followed by post-operative standard radiation therapy are addressed in section 1.2.3.3. It is currently unknown what the cosmetic impact will be of the addition of durvalumab and oleclumab to this combination. As discussed in the above paragraph, pre-clinical data show that adenosine blockade might reduce radiation therapy induced fibrosis but the effect remains to be validated in humans.

1.3.1.2.2. Neo-Checkray safety run-in results and IDMC recommendation

Safety run-in was completed by 01/09/2020 and the results were presented to an IDMC on 24/09/2020. The IDMC recommended to proceed with the phase II randomized trial. Details on the safety run-in and the IDMC recommendation can be found in section 3.2.1.6.

1.3.2. Benefits

Potential benefits of the study are an increased response to the pre-operative treatment in comparison to the standard treatment. As a consequence, an improvement of the breast conservation surgery rate, invasive disease-free survival and overall survival is possible. Moreover, it is possible that the combination of SBRT and immunotherapy works as a vaccine protecting the subject against recurrence. However, there is no clinical data available yet validating this hypothesis based on pre-clinical evidence as detailed above.

2. OBJECTIVES AND ENDPOINTS OF THE STUDY

2.1. Objectives

2.1.1. Safety Run-in objectives

- To evaluate safety and toxicity of adding SBRT directed to the primary tumour to the combination durvalumab-oleclumab-paclitaxel.
- To evaluate feasibility of performing surgery (breast conserving surgery or mastectomy) within 6 weeks after the end of neo-adjuvant treatment.

2.1.2. Phase II Primary objective

2.1.3. To demonstrate improved tumour response of the primary tumour and nodal metastases in arms 2 or 3 versus arm 1Phase II Secondary objectives

At surgery:

- To evaluate the complete pathological response rate defined as ypT0/Tis ypN0: absence of residual invasive disease, residual in situ carcinoma is accepted.
- To evaluate the complete pathological response rate defined as ypT0 ypN0: absence of residual invasive disease *and* in situ carcinoma.
- To evaluate the response to the primary tumour irrespective of the response to the pathological lymph nodes.
- To evaluate the response to the pathological lymph nodes irrespective of the response to the primary tumour.
- To evaluate the feasibility to perform breast-sparing surgery of the arms 2 and 3 versus arm 1.
- To demonstrate an increase in TIL levels of the primary breast cancer between baseline and the week 6 biopsy.

Follow-up phase:

- To evaluate the ability to control invasive disease and survival in arms 2 and 3 versus arm 1 at 3 and 5 years after surgery.
- To evaluate the severity and duration of AEs of the arms 2 and 3 versus arm 1.
- To evaluate the cosmetic changes to the breast of the arms 2 and 3 versus arm 1

2.1.4. Phase II Exploratory objectives

- Evaluation of ovarian function 1, 2, 3 and 5 years after surgery in premenopausal subjects and perform comparison between treatment arms and in comparison to baseline (week 1 blood for translational research).
- Collect data about pregnancy and breastfeeding in the follow-up phase.
- Evaluate if biopsy at week 6 can predict the occurrence of pathological complete response (pCR) at surgery.
- Evaluate the use of plasma ctDNA in the pre-operative phase to predict response at surgery.
- Evaluate the use of plasma ctDNA in the follow-up phase to predict cancer relapse.
- Evaluate MRI images at baseline and at week 12 to predict response to surgery and investigate the correlation with TIL dynamics.

  Note: see section 11.2 for the translational objectives.

2.2. Endpoints

2.2.1. Safety run-in endpoints

- Occurrence of immune related or radiation therapy related toxicity of special interest as detailed in section 3.2.1.4.
- Feasibility of delivering a sufficient dose of paclitaxel and ddAC (details in section 3.2.1.4).
- Feasibility of performing surgery within a specified timeframe after the last neo-adjuvant treatment (details see section 3.2.1.4).

2.2.2. Phase II Primary endpoint

- Residual cancer burden (**RCB 0-1 vs. RCB 2-3**) at time of surgery. RCB 0 is defined as pathological complete response (pCR) and RCB 1 is defined as minimal residual disease. RCB is calculated as a continuous index combining pathologic measurements of the primary tumour (size and cellularity) and nodal metastases (number and size) as defined by Symmans et al (15,88).

2.2.3. Phase II Secondary endpoints

At surgery:

- Pathological complete response rate (pCR), defined as pathological stage ypT0/Tis ypN0 at the time of definitive surgery (88). pCR (ypT0/Tis ypN0) is the absence of residual invasive cancer. Residual in situ carcinoma is accepted.

  Important note: this secondary endpoint is an answer to the **primary** objective of the trial (see section 2.1.2), expressed in a slight different way than RCB 0/1. Recent phase III trials investigating the use of neo-adjuvant immunotherapy in ER+ breast cancer used ypT0/Tis ypN0 as primary endpoint instead of RCB 0/1 (see end of section 1.2.1.1). Therefore pCR (ypT0/Tis ypN0) is the most important secondary endpoint in this study.

- pCR-no DCIS (ypT0 ypN0) rate, defined as the absence of residual invasive and in situ cancer.
- Complete pathologic response rate (pCR) of the primary tumour (ypT0/Tis), irrespective of the response rate of the resected nodal metastases.
- Complete pathologic response rate (pCR) of the resected nodal metastases (ypN0), irrespective of the response rate of the primary tumour.% of breast conservation surgery in arms 2 and 3 versus arm 1.
- Change in TIL levels between baseline and the week 6 biopsy.

Follow-up phase:

- Efficacy endpoints at 3 years and 5 years after surgery will be measured, as defined by the Standardized Definitions for Efficacy End Points in Neoadjuvant Breast Cancer Clinical Trials (NeoSTEEP) (88). The following endpoints will be assessed: event-free survival (**EFS**), breast cancer event-free survival (**BC-EFS**), overall survival (**OS**) and distant recurrence-free survival (**DRFS**). Furthermore, the occurrence of **ipsilateral locoregional recurrence** (breast, chestwall or locoregional nodal recurrence), **ipsilateral local recurrence** (breast or chest wall)

(laterality of the index lesion), and **ipsilateral locoregional nodal recurrence** (laterality of the index lesion) will be assessed. The endpoints will be measured using regular follow-up investigations: lab work, clinical examination and annual breast ultrasound and mammography. Radiologic imaging will not be routinely performed, unless directed by abnormal blood results or clinical examination.

- Duration and severity of AEs based on CTCAE 5.0.
- Changes in breast appearance: breast fibrosis in whole breast, breast fibrosis in boost area, breast size, breast shape, nipple position, shape of the areola and nipple, skin color, appearance of surgical scar, evaluation of teleangiectasia and global cosmetic result. Digitalized color photographs will be taken at multiple timepoints and the cosmetic changes on the color photographs will be scored quantitatively by the BCCT.core software program (89–91). Information on cosmetic and plastic surgical procedures will be collected (for example: oncoloplastic surgery, breast implants, and other procedures).

2.2.4. Phase II Exploratory endpoints

- Measurement of ovarian function in premenopausal women, with comparison between treatment arms (92). Ovarian function will be measured in blood by evaluation of anti-Mullerian hormone (AMH), follicle stimulating hormone (FSH), estradiol (E2) and progesterone at baseline and after surgery at 1 year, 2 years, 3 years and 5 years in premenopausal subjects, and compared to baseline.
- Information about pregnancies, pregnancies attempts and breasfeeding. The following data will be collected: spontaneous menstruation recovery, number of pregnancy attempts, number of pregnancies (determined by pregnancy test), pregnancy outcome (full term pregnancy, caesarean section, abortion, miscarriage, ectopic, stillbirth), offspring outcomes (preterm birth, low birth weight, birth defects), breastfeeding (pattern of breastfeeding, duration, use of ipsilateral breast if previous breast conservation, side exclusivity), use of assisted reproductive technology (ART).
- Correlation between cellularity and biomarkers on the week 6 biopsy and pathological complete response (pCR) at surgery.
- Correlation between plasma ctDNA during the pre-operative phase and response at surgery.
- Correlation between plasma ctDNA and clinical tumor relapse in the follow-up phase.
- The correlation between MRI images at baseline and at week 12 with the response to surgery and changes in TILs.l

3. STUDY DESIGN

3.1. General design of the trial

Figure 1: Overall design of the Neo-CheckRay trial

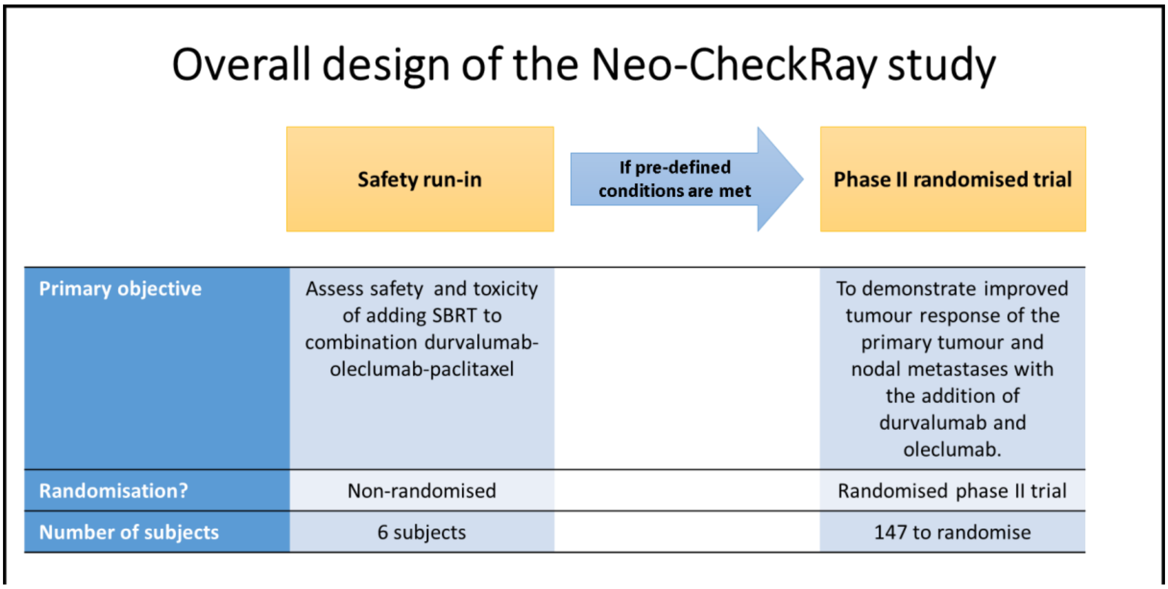

This trial consists of a safety run-in followed by a phase II randomised trial. The goal of the safety run-in is to assess the safety of adding SBRT to the neo-adjuvant systemic treatment. The doses of the IMPs will be identical in the safety run-in and the phase II randomised trial. Individual subject timelines are also identical in the safety run-in and the phase II randomised trial.

Figure 2: Individual subject timelines in the safety run-in and the phase II randomised trial

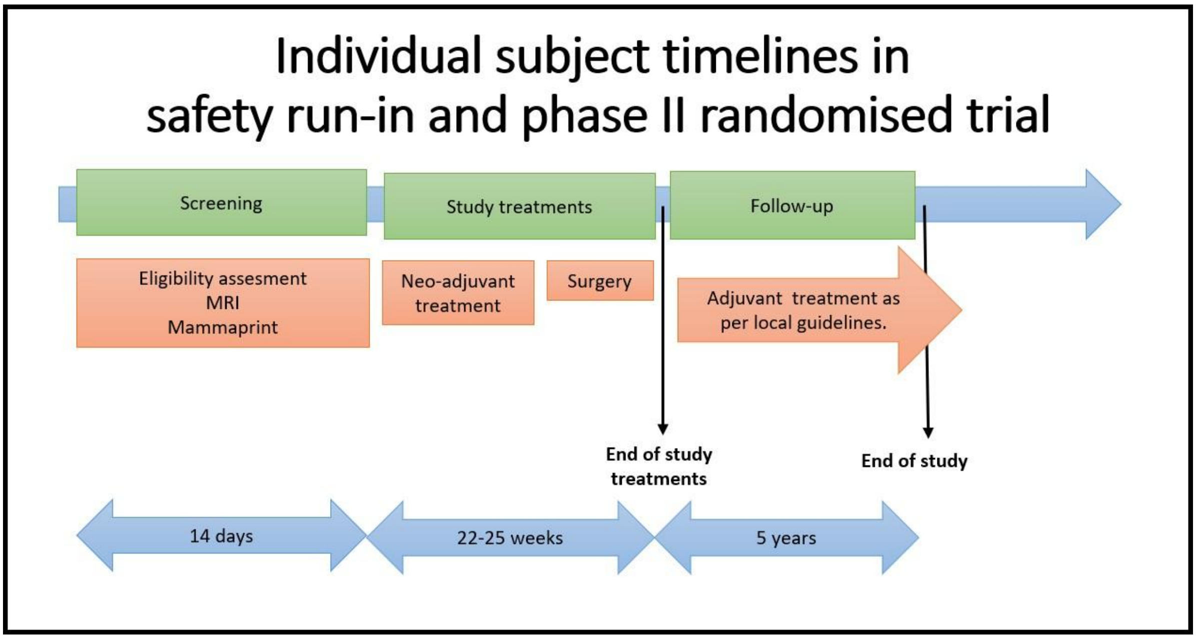

3.2. Pre-operative phase

3.2.1. Safety run-in [completed]

Note: the safety run-in was completed and its results discussed with an IDMC on 24/09/2020. The IDMC recommended to proceed with phase II of the Neo-Checkray study. Recommendations of the IDMC were implemented in protocol version 4.0. Details on the safety run-in remain in the present protocol for general study comprehensiveness, but have been fully executed. Details about the safety run-in results and IDMC recommendation are discussed in section 3.2.1.6. The results of the safety run-in were published in the Journal of Immunotherapy for Cancer in December 2023 (93).

3.2.1.1. *Rationale*

- All doses of IMPs and radiation therapy have been independently tested on humans in previous trials, making a phase 1 dose-identifying trial unnecessary (*vide infra*).
- The safety run-in was solely performed to check the safety of the specific combination of IMPs and radiation therapy proposed in this trial. However, it is important to accentuate that several combinations have already been tested and are considered safe (as explained in section 1.3):
  - The combination of oleclumab, durvalumab and paclitaxel
  - The combination of SBRT to the primary breast tumour and chemotherapy
  - The combination of SBRT and anti-PD-L1
- Therefore, the safety run-in was limited to testing the combination of weekly paclitaxel followed by dose-dense doxorubicin-cyclophosphamide (ddAC), along with the addition of durvalumab and oleclumab and a pre-operative radiation therapy (boost dose) on the primary tumour during week 4-5. This treatment combination was chosen as it contains all study treatments that were given in the phase II and corresponds to arm 3 of the phase II. **The goal of the safety run-in was not to evaluate toxicity of the individual IMPs, which are already known, but to rule out the small risk of excess toxicity resulting from the specific combination of the different IMPs.**

Figure 3: the safety run-in assesses the feasibility and toxicity of adding SBRT to the combination paclitaxel-durvalumab-oleclumab

The safety run-in assesses the feasability and toxicity of adding SBRT to paclitaxel-durvalumab-oleclumab

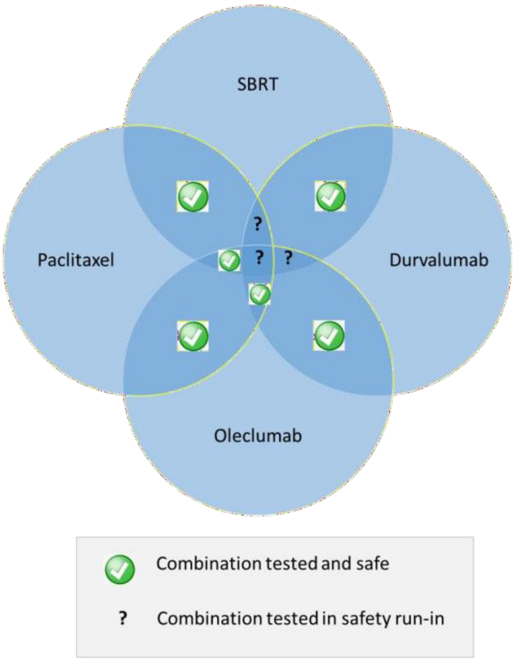

3.2.1.2. *Design*

The safety run-in was done as a precursor to the phase II randomised part of the Neo-CheckRay trial. Six subjects were included in the safety run-in. These subjects are not part of the phase II total recruitment.

Subjects in the safety run-in received the following treatments corresponding to arm 3 of the phase II randomised trial. This consists of:

- q1w paclitaxel 80 mg/m² IV for 12 adminstrations (12 weeks) followed by q2w dose-dense doxorubicin -cyclophosphamide IV (60 mg/m² and 600 mg/m² respectively) for 4 administrations (8 weeks)
- Anti-PD-L1 antibody durvalumab 1500 mg IV q4w for 5 adminstrations (20 weeks)
- Anti-CD73 antibody oleclumab 3000 mg IV q2w for 4 administrations (8 weeks), followed by q4w for 3 administrations (12weeks)
- Pre-operative radiation therapy (boost dose) 3x8 Gy on the primary tumour at week 5

Surgery, adjuvant radiotherapy and follow-up are identical for the phase II randomised trial and are detailed in section 3.3.

Figure 4: Study treatment during safety run-in

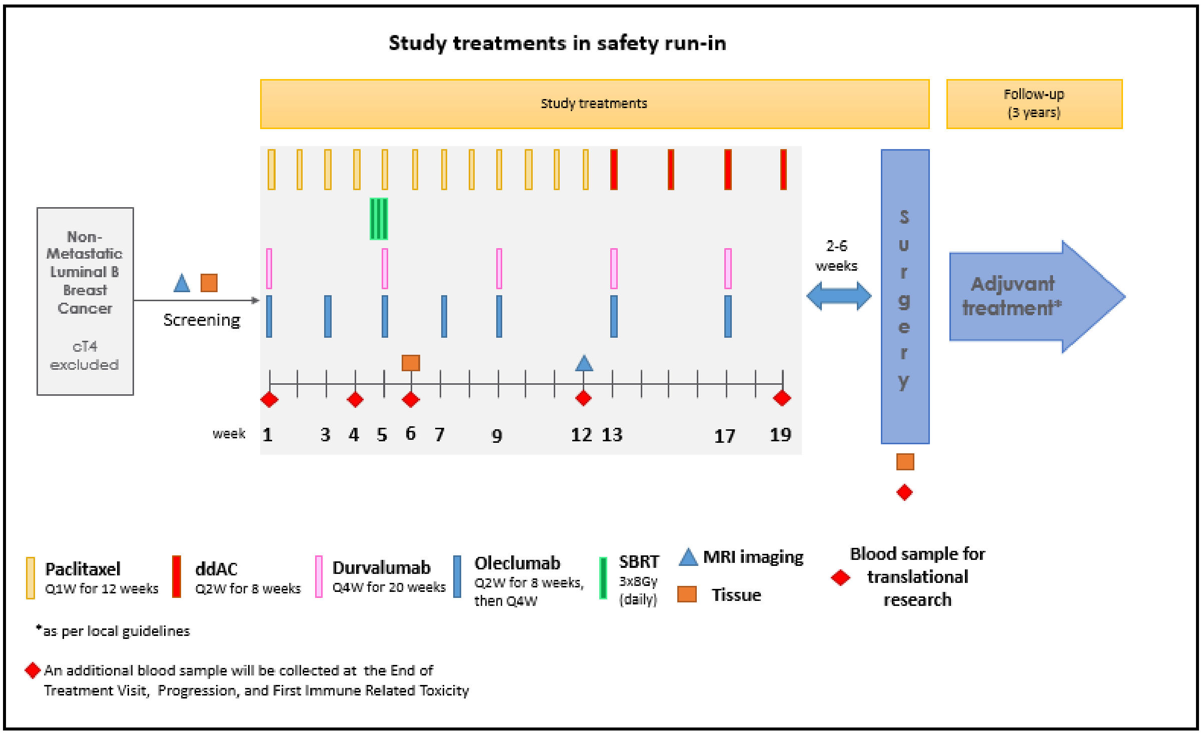

3.2.1.3. *Subject enrolment rules during safety run-in*

- A timeframe of 7 calendar days had to be present between the enrolment of each subject in order to assess for acute adverse events.
- The first fraction of SBRT of each subject could only be given at the earliest 48 hours after the last fraction of SBRT given to the previous subject. This time interval was feasible given the inclusion rate with a time interval of 7 calendar days. A report on the subject's acute tolerance to SBRT had to be distributed to all participating centres before the time of SBRT treatment of the next subject. The report had to consist of a grading of radiation dermatitis according to NCI-CTCAE v5 during a clinical examination 20-28 hours after the last SBRT fraction.
- In case of a grade III radiation dermatitis occurring for any subject at any time before surgery, the dose per fraction for all fractions of the next subject treated with SBRT were reduced by 1 Gy. The centre had to report within 24 hours of awareness, in writing, to the sponsor any grade III-IV radiation dermatitis as described in the section 10.2.4. All other centres were notified in writing within 24 hours in case of a grade III radiation dermatitis.

3.2.1.4. *Requirements to meet before starting the phase II randomised trial*

Six subjects were included in the safety run-in.

The phase II part of the trial can start once all the following conditions are met:

1. Conditions for all 6 subjects included in the safety run-in:

- All 6 subjects have undergone SBRT and were assessed at least once at minimum 2 weeks after SBRT.

- Not more than 1 out of 6 subjects included in the safety run-in experienced an immune related or radiation therapy related toxicity of special interest defined as follows:
  - Immune related toxicity of special interest:
    - Any Grade 4 immune-related AE
    - Any ≥ Grade 3 colitis
    - Any ≥ Grade 3 renal failure/nephritis
    - Any ≥ Grade 3 non-infectious pneumonitis irrespective of duration
    - Any Grade 3 immune-related AE, excluding colitis, renal failure/nephritis and pneumonitis, that does not downgrade to ≤ Grade 2 within 3 days after onset of the event despite maximal medical supportive care including systemic corticosteroids or does not downgrade to ≤ Grade 1 or baseline within 14 days
    - Liver transaminase elevation ≥ 5 ULN or total bilirubin > 3 × ULN regardless of duration or reversibility
    - Any increase in AST or ALT > 3 × ULN and concurrent increase in total bilirubin > 2 × ULN
  - The following will **not** be considered as an immune related toxicity of special interest:
    - Grade 4 asymptomatic lipase/amylase elevation with no radiographic evidence of pancreatitis.
    - Grade 3 fatigue that improves by at least 1 grade within 14 days
    - Grade 3 endocrine disorder (thyroid, pituitary, adrenal insufficiency, and/or diabetes) that can be managed with or without systemic corticosteroid therapy and/or hormone replacement therapy
    - Grade 3 inflammatory reaction attributed to a local antitumour response (e.g., inflammatory reaction at sites of metastatic disease, lymph nodes, etc.) that resolved to ≤ Grade 1 within 30 days
    - Concurrent vitiligo or alopecia of any AE grade
    - Grade 3 IRR (first occurrence and in the absence of steroid prophylaxis) that resolves within 6 hours with appropriate clinical management.
    - Grade 3 or Grade 4 neutropenia ≤ 7 days
    - Grade 3 or Grade 4 lymphopenia
    - Grade 3 or Grade 4 anemia
    - Grade 3 or Grade 4 thrombocytopenia ≤ 7 days that is not associated with clinically significant bleeding that requires medical intervention
    - Isolated Grade 3 electrolyte abnormalities that are not associated with clinical signs or symptoms and are reversed with appropriate maximal medical intervention within 3 days
    - Grade 3 fever lasting ≤ 24 hours with or without medical therapy
  - Radiation therapy related toxicity of special interest:
    - Grade 3 or grade 4 radiation dermatitis that did not downgrade to grade 2 radiation dermatitis within 4 weeks of radiation treatment
    - Any ≥ Grade 3 non-infectious pneumonitis irrespective of duration

2. *Conditions applying only to the 3 first subjects included in the safety run-in*:

- Three out of three subjects have undergone surgery within maximum 7 weeks of the last neo-adjuvant treatment.

- Three out of three subjects that have undergone surgery received at least 75% of the planned dose of paclitaxel and dose-dense AC.

3. The final decision to proceed with the phase II randomised trial will be performed in consultation with the IDMC.

- If points 1 or 2 were not met due to circumstances unrelated to the study treatment, then these points will be discussed during this meeting. In this case, more subjects could be added in order to meet the safety criteria.

3.2.1.5. *Procedure if the safety run-in conditions do not allow proceeding with Phase II randomised trial*

In case the conditions to proceed to the phase II randomised trial are not met, a new safety run-in will be planned in consultation with the IDMC. The new safety run-in will be performed with a dose reduction of the investigational part of the treatment regimen attributed to the excess toxicity. Subjects will be recruited until the adjusted treatment regimen meets the safety criteria.

Figure 5: Conditions to access Phase II

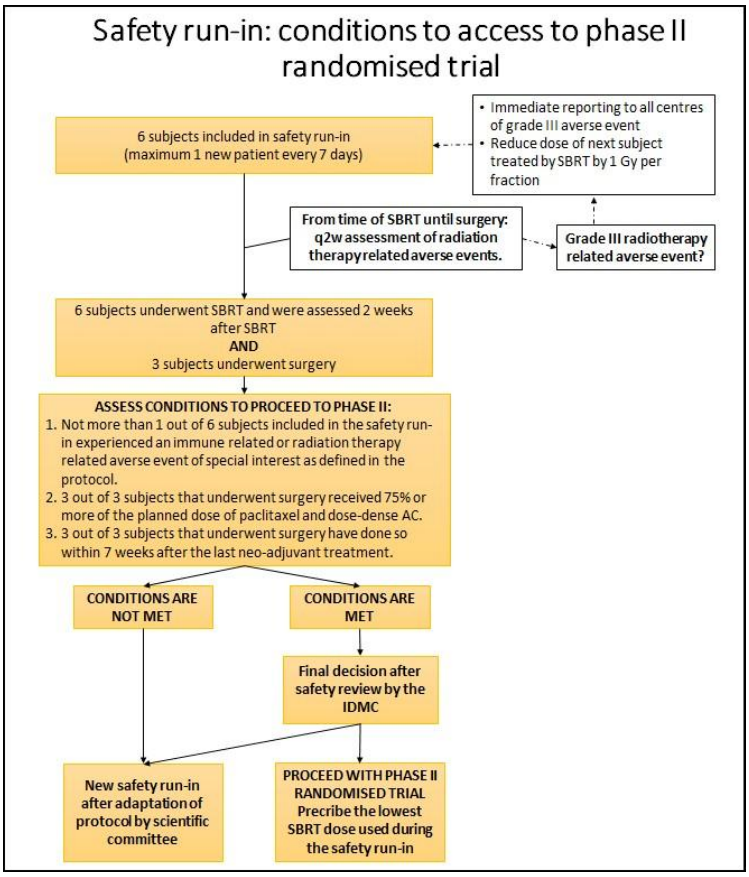

3.2.1.6. *Results of the safety run-in and IDMC recommendations*

As planned and detailed in section 3.2.1, a safety interim analysis was performed at the end of the safety run-in and presented to an IDMC before starting the phase II randomized trial.

Neo-Checkray started enrolment on 06/11/2019 . Safety run-in analysis was done on the database version of 11/08/2020. At that moment, 6 patients underwent SBRT and at least 3 patients underwent surgery. No radiation therapy related toxicity of special interest was reported. The first 3 included patients underwent surgery within 7 weeks of the last neo-adjuvant treatment and the first 3 included patients received >75% of the planned dose of paclitaxel and dose-dense AC. All patients experienced grade 1 and 2 adverse events. One patient experienced a grade 3 adverse event : pericarditis, which was also recorded as an adverse event of special interest. Hence, the conditions for starting phase II part of the Neo-checkray trial were met (see section 3.2.1.4 for the requirements to meet before starting phase II) and the results were presented to an IDMC.
The recommendations of the IDMC were implemented as from protocol version 4.0. This version was issued before starting the phase II part of the Neo-Checkray trial. The results of the safety run-in were published in the Journal of Immunotherapy for Cancer in December 2023 (93).

3.2.2. Phase II randomised trial

Luminal B breast cancer subjects candidate for neo-adjuvant chemotherapy will be randomised in a 1:1:1 ratio between 3 arms:

1. Arm 1: the combination of weekly paclitaxel 80 mg/m² IV followed by q2w dose-dense doxorubicin-cyclophosphamide (ddAC) (60 mg/m² doxorubicin IV and 600 mg/m² cyclophosphamide IV) and pre-operative radiation therapy on the primary tumour (3x8 Gy).
2. Arm 2: drugs regimen of Arm 1 with the addition of the anti-PD-L1 antibody durvalumab IV 1500 mg q4w.
3. Arm 3: drugs regimen of Arm 2 with the addition of the anti-CD73 antibody oleclumab IV 3000 mg q2w for 4 administrations, followed by q4w for 3 administrations.

Surgery, adjuvant radiotherapy and follow-up are detailed in section 3.3.

Figure 6: Study treatments of the phase II randomised trial

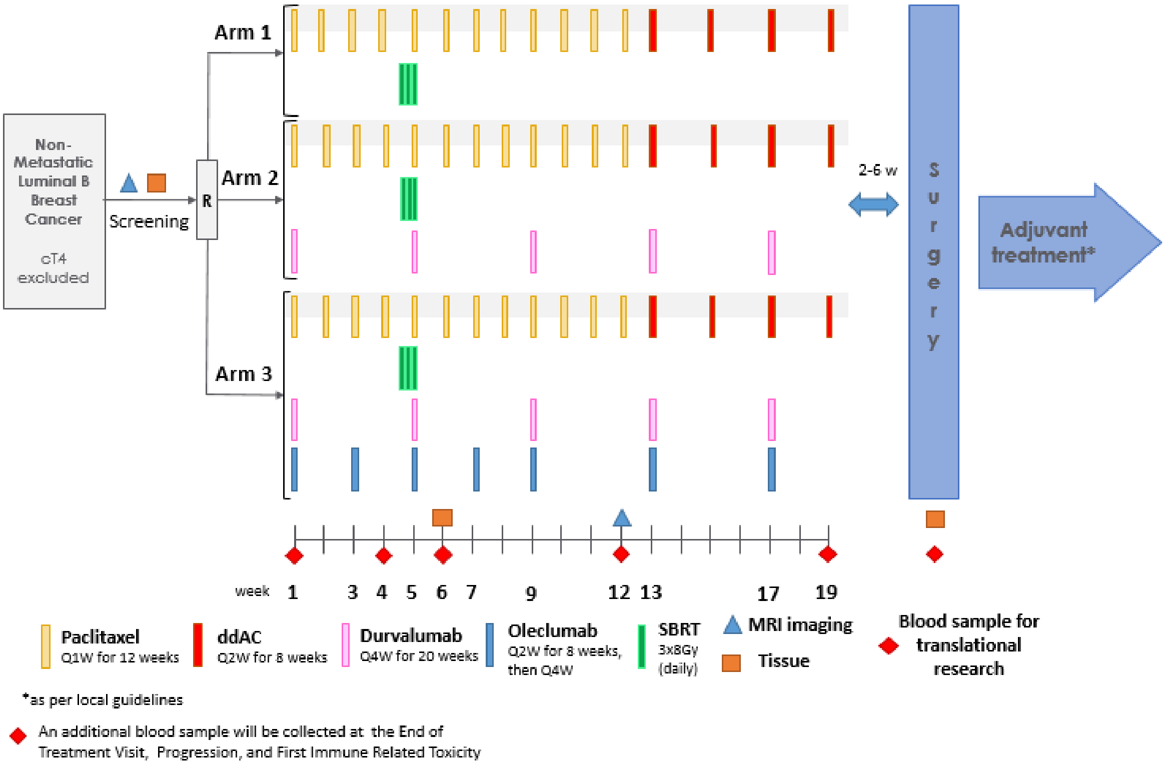

3.2.3. Treatment and work-up details

3.2.3.1. *Pre-operative SBRT*

The radiation therapy will consist of an SBRT technique (stereotactic body radiotherapy) given in 3 fractions.

Rules for SBRT scheduling:

- Administration of maximum one fraction per day.
- The 3 fractions will be spread at the minimum over 3 days and at the maximum over 6 days.

    - This rule was instaured to be able to spread the SBRT treatments over a weekend, in case of holidays, or to be able to schedule a subject in case the linear accelerator is unavailable due to maintenance or other reasons.
- Third fraction of SBRT will take place the day before or on the same day as the 5th administration of paclitaxel (arms 1, 2 and 3), the 2nd administration of durvalumab (arms 2 and 3) and the 3rd administration of oleclumab (arm 3). In case the 2nd administration of durvalumab and the 3rd administration of oleclumab is delayed, the whole SBRT treatment should also be delayed in such a way that the 3rd SBRT fraction is given on the same day than the 2nd durvalumab and 3rd oleclumab. A delay of only paclitaxel will not delay the SBRT treatment.
- If SBRT is given on the same day than the study medication, the SBRT should be given PRIOR to any study medications.

Example: if the 5th administration of Paclitaxel is planned on a Wednesday:

- First fraction of SBRT: at the earliest on Thursday preceding the Paclitaxel and at the latest the Monday preceding Paclitaxel
- Last fraction of SBRT: should be given on Tuesday or Wednesday. If given on Wednesday, it should be given BEFORE any study medications.

SBRT can thus be given on Thursday and Friday if the systemic treatment, and third fraction, is given on a Monday.

3.2.3.2. *Clinical and radiologic work up during pre-operative phase*

A clinical and radiologic work up of the breast (MRI is on week 12, before switching the chemotherapy to ddAC. In case the breast MRI reveals a potential tumour progression, a new breast MRI is to be planned within 4-6 weeks to exclude pseudo-progression. If progression is confirmed, the subjects can be withdrawn from weekly study treatment at local investigator discretion. In case of progression, the local investigator can decide to keep the subjects in the trial and continue study treatment with ddAC, as planned by the study design. All cases of progression at the work-up will be discussed with the principal investigators during the studies scientific call. The requirement for an MRI is not applicable in the case of medical contraindications to perform MRI (e.g., obesity or claustrophobia). In this situation, tumour evaluations should be performed by ultrasound.

Any other (unplanned) work up will follow the same rule to exclude pseudo-progression, unless the work up was performed less than 6 weeks to surgery, in that case the subject will undergo surgery, making it unnecessary to confirm pseudo-progression.

3.3. Surgery and end of treatment

The primary tumour will be excised 2-6 weeks after completion of ddAC. The study treatments end at surgery.

3.4. Follow-up

All treatments after surgery, such as post-operative radiotherapy and hormonal therapy, will be performed according to standard of care and local site guidelines.

3.4.1. Adjuvant radiotherapy

After surgery, radiation therapy will be given to the breast or chest wall according to local study site treatment guidelines (standard of care). The radiation oncologist treating the subject can take into account the pre-operative radiation dose that was already given to the tumour, however he/she is free to choose the treatment dose and field selection according to his/her expertise and local study sites guidelines.

3.4.2. Follow-up and hormonal treatment after study treatments

Treatment with adjuvant hormonal therapy can start per national guidelines or local site guidelines. Subjects will be continued to be followed up to 60 months after surgery to evaluate the secondary endpoints of the study. Further details on follow-up schedule of assessments are discussed in section 8.4.

4. SELECTION OF SUBJECTS

4.1. Inclusion criteria

Subjects must meet all of the following criteria in order to be eligible for this study:

1. Age ≥ 18 years old
2. Female
3. ECOG performance status ≤ 1
4. Weight ≥ 35 kg.
5. Histological diagnosis of invasive breast adenocarcinoma that is estrogen receptor-positive (ER-positive) and HER2- negative, as per the updated American Society of Clinical Oncology (ASCO) - College of American Pathologists (CAP) guidelines and performed according to local testing. In addition, only tumours with Proliferation Index Ki67 ≥ 15% **or** histology grade III are accepted.
6. Agreement to perform new study related biopsies to provide tissue samples
7. MammaPrint genomic high risk score according to centralised testing, except for specific conditions as mentioned below. Mammaprint will only be tested for luminal B breast tumours with either Proliferation Index Ki67 ≥ 15% or histology grade III tumours. (Testing to be done during screening period).

MammaPrint result status at time of termination of all other screening procedures:

- *MammaPrint is high risk*: subject may be randomized.
- *MammaPrint is low risk*: subjet can not be randomized.
- *MammaPrint result is not yet known*:

  If the MammaPrint result is not known at time of termination of all other screening procedures, the investigator is allowed to randomize the subject and start study treatment without waiting for the result of the MammaPrint in the following situations:

		Age < 50 years	Age ≥ 50 years
Ki67 ≤ 20%	cN0	Wait for MammaPrint result	

and grade II	cN+		
Ki67 > 20 % or grade III	cN0	Allowed not to wait for MammaPrint result	Wait for MammaPrint result
	cN+	Allowed not to wait for MammaPrint result	

If the subject is randomized in a situation without known MammaPrint result and the MammaPrint result proves to be low risk after randomisation, the subject will stay in the study.

- <u>*MammaPrint result is unevaluable or is technically impossible*</u>:

  The sponsor should be contacted as soon as possible to discuss the inclusion of the concerned subject. Under specific medical conditions and breast cancer disease characteristics, the medical team of the sponsor can accept that the site continues the screening process and randomization of the subject. There will be maximum 5% of non-evaluable Mammaprint results among enrolled patients.

8. Tumour size:
   - If subject is cN0: tumour size ≥ 2 cm, as determined by MRI imaging.
   - If subject is cN1, cN2 or cN3: tumour size ≥ 1.5 cm, as determined by MRI imaging.

   The requirement for an MRI is not applicable in the case of medical contraindications to perform MRI (e.g., obesity or claustrophobia). In this situation, tumour evaluations should be performed by ultrasound.

9. Multifocal, multicentric unilateral or bilateral breast adenocarcinoma tumours are allowed provided that all biopsiable foci are ER+/HER2- according to local testing and all foci are able to receive SBRT treatment within the defined dosimetric constraints. In some cases a separate biopsy of every focus is not mandatory, but only if every of the following conditions are present:
   - small focal lesion
   - lesion in close proximity to the main primary cancer from which a biopsy was taken
   - the investigator and the radiologist consider the lesion to be clearly related to the main primary breast cancer from which a biopsy was taken
   - the lesion will be removed during the same lumpectomy than the main primary breast cancer

   For bilateral, multifocal or multicentric disease, the site selected for pre-treatment biopsy should correspond to the site of largest measurable disease meeting eligibility criteria. The location of tumour biopsy site (laterality, quadrant, position from the nipple and type of imaging modality to guide biopsy) should be collected (see Appendix 2).

10. Serum pregnancy test (for subjects of childbearing potential) negative within 2 weeks prior to first dose of study administration.

11. Women of childbearing potential must agree to use 1 highly effective method of contraception (see protocol section 6.9.1) during the screening period, during the course of the study and at least 12 months after the last administration of study treatment. It is

strongly recommended for the male partner of a female subject to also use male condom plus spermicide throughout this period.

12. Adequate bone marrow function as defined below:
    - Absolute neutrophil count ≥1500/μL, i.e. $1.5x10^9$/L
    - Hemoglobin ≥ 9.0 g/dL
    - Platelets ≥100000/μL, i.e. $100x10^9$/L
13. Adequate liver function as defined below:
    - Serum total bilirubin ≤ 1.5 x ULN. In case of known Gilbert's syndrome ≤ 3 x UNL is allowed
    - AST (SGOT) ≤ 3.0 x ULN
    - ALT (SGPT) ≤ 3.0 x ULN
14. Adequate renal function as defined below:
    - Creatinine ≤ 1.5 x UNL or eGFR≥40ml/min/1.73m²
15. Adequate coagulant function as defined below:
    - International Normalized Ratio (INR) ≤ 1.5 x ULN
16. Completion of all necessary screening procedures within 28 days prior to randomisation (except if written differently).
17. Willingness to provide tissue and blood samples for immuno-monitoring and translational research activities
18. Left ventricular ejection fraction (LVEF) ≥ 50%. LVEF performed in routine is accepted if done within 6 months prior to beginning of screening.
19. Signed Informed Consent form (ICF) obtained prior to any study related procedure.

Inclusion criterion for phase II only (all phase II subjects):

20. Tumour sample provided for central PD-L1 IHC assessment. (Testing done during screening period).

Inclusion criterion applicable to FRANCE only (all safety run-in and phase II subjects):

21. Affiliated to the French Social Security System (applicable only to subjects treated in France)

4.2. Exclusion criteria

Subjects meeting one of the following criteria are not eligible for this study:

1) Pregnant and/or lactating women.
2) Subject with a significant medical, neuro-psychiatric, substance abuse or surgical condition, currently uncontrolled by treatment, which, in the principal investigator's opinion, may interfere with completion of the study.
3) TNM stage cT4 breast cancer including inflammatory breast cancer.
4) Presence of any distant metastasis.
5) Contra-indication for treatment by paclitaxel, doxorubicin or cyclophosphamide, or known allergy to any tested substances or any excipients (e.g; chemotherapy or

immunotherapy formulations). Contra-indication for subjects with known sensitivity to acetaminophen/paracetamol, diphenhydramine or equivalent antihistamine (this is a contra-indication for treatment with oleclumab).

6) Previously known contra-indication for treatment by radiation therapy such as rare genetic disorders associated with DNA repair disorders such as ataxia-telangiectasia (A-T), Nijmegen Breakage Syndrome (NBS) and Fanconi anemia.

7) Active or prior documented autoimmune disease (including inflammatory bowel disease, celiac disease, Wegener's granulomatosis) within the past 3 years. NOTE: Subjects with childhood atopy or asthma, vitiligo, alopecia, Grave's disease, Hashimoto's thyroiditis, or psoriasis not requiring systemic treatment (within the past 2 years) are not excluded

8) Prior malignancy active within the previous 5 years, except for localised cancers that are considered to have been cured and in the opinion of the investigator present a low risk for recurrence. Examples include basal or squamous cell skin cancer, superficial bladder cancer, or carcinoma in situ of the cervix or breast.

9) Known history of, or any evidence of active, non-infectious pneumonitis.

10) Active infection including:

- Tuberculosis (TB) (clinical evaluation that includes clinical history, physical examination and radiographic findings, and TB testing in line with local practice)
- Hepatitis B (known positive HBV surface antigen (HBsAg) result). Subjects with a past or resolved HBV infection (defined as the presence of hepatitis B core antibody [anti-HBc] and absence of HBsAg) are eligible.
- Hepatitis C. Subjects positive for hepatitis C (HCV) antibody are eligible only if polymerase chain reaction is negative for HCV RNA.

11) Significant cardiovascular disease, such as New York Heart Association cardiac disease (Class II or greater), myocardial infarction, transient ischemic attack, or stroke within the previous 3 months, unstable arrhythmias, and/or unstable angina.

12) Medical condition requiring current systemic anticoagulation, or a history of congenital hypercoagulable condition. Subjects taking aspirin at doses < 325 mg per day are eligible provided that prothrombin time is within the institutional range of normal. Use of local anticoagulation for port maintenance is permitted.

13) Subjects with history of venous thrombosis in the past 12 months prior to the scheduled first dose of study treatment (oleclumab).

14) Diabetes mellitus Type 1 or poorly controlled Type 2 diabetes mellitus defined as a screening hemoglobin A1C ≥ 8 % or a fasting plasma glucose ≥ 160 mg/dL (or 8.8 mmol/L)

15) Any live (attenuated) vaccine within 30 days of planned start of study therapy.

16) Prior systemic immunosuppressive medication (excluding corticosteroids) within 30 days of planned start of study therapy.

17) Prior radiation therapy to the ipsilateral breast.

18) Prior immunotherapy, including tumour vaccine, cytokine, anti-CTLA4, PD-1/PD-L1, including durvalumab, blockade or similar agents.

19) Concomitant use of other investigational drugs.

20) Any unresolved toxicity NCI CTCAE Grade ≥2 from previous anticancer therapy with the exception of alopecia, vitiligo, and the laboratory values defined in the inclusion criteria. Subjets with Grade ≥2 neuropathy will be evaluated on a case-by-case basis after

consultation with the Study Physician. Subjects with irreversible toxicity not reasonably expected to be exacerbated by treatment with durvalumab or oleclumab may be included only after consultation with the Study Physician.

21) Uncontrolled intercurrent illness, including but not limited to, ongoing or active infection, symptomatic congestive heart failure, uncontrolled hypertension, unstable angina pectoris, cardiac arrhythmia, interstitial lung disease, serious chronic gastrointestinal conditions associated with diarrhea, or psychiatric illness/social situations that would limit compliance with study requirement, substantially increase risk of incurring AEs or compromise the ability of the subject to give written informed consent.

22) History or current evidence of any condition, therapy, or laboratory abnormality that might confound the results of the trial, interfere with the subject's participation for the full duration of the trial, or is not in the best interest of the subject to participate, in the opinion of the treating investigator.

23) Prior organ transplantation.

24) Subjects with urinary outlflow obstruction.

Exclusion criterion applicable to FRANCE only (all safety run-in and phase II subjects):

25) Vulnerable persons according to the article L.1121-6 of the CSP, adults who are the subject of a measure of legal protection or unable to express their consent according to article L.1121-8 of the CSP.

4.3. Subject registration/randomisation procedure

The procedure for a subject participation in this study is a two steps procedure: a registration step and an enrolment/randomisation step.

As soon as the subject has signed the informed consent form, she should be registered in the web based "Subject Registration Tool" (= step 1 registration). The system assigns the study subject number.

For the safety run-in, if the screened subject meets all eligibility criteria, she is enrolled via the web based "Subject Registration Tool" (= step 2 enrolment). For the phase II, this second step will be a randomisation process. A maximum of 1 working day is allowed between subject's enrolment/randomisation and first study drug administration.

The treatment allocation algorithm will make use of the following stratification factors :

- PD-L1 status
- Primary tumour size: cT1/cT2 versus cT3
- Nodal status: cN0 versus cN+
- Participating site

If the subject does not meet all eligibility criteria, she is considered as a screen failure. Screen failures have also to be reported via the web based "Subject Registration Tool" as not enrolled or not randomised.

Screen failure is defined as any subject that signed the ICF and finally is not enrolled or randomised in the study.

Screening and enrolment/randomisation guidelines are provided to the participating sites.

5. CRITERIA FOR SUBJECT WITHDRAWAL

The investigator has the right to discontinue a subject from the study treatment or withdraw a subject from the study at any time. The subject has also the right to discontinue the study treatment or withdraw from the study at any time without giving their reasons. Although a subject is not obliged to give his/her reason for withdrawing prematurely from the study treatment or the complete study, the investigator should make reasonable effort to ascertain the reason(s), while fully respecting the subject's rights.

Reasons of premature discontinuation from the study treatment or from the entire study may include, but are not limited to, the following:

- Subject's request/ consent withdrawal
- Any medical condition that the investigator or the sponsor considers may jeopardise the subjects' safety if she/he continues the study treatment or the study
- Investigator or sponsor determines it is in the best interest of the subject
- Subject non-compliance
- Pregnancy

The reason should be documented in the medical notes of the subject and in (e)CRF.

In case, the subject decides to stop prematurely the study treatment, the investigator will ask her/him if they agree to continue attending the study visits as per schedule assessments and/or to provide survival data.

The outcome of the discussion between the investigator and the subject should be documented in the medical notes of the subject and in the (e)CRF.

Any subject who discontinues the study will be encouraged to return to the site for a study completion visit and to allow appropriate safety evaluations of ongoing AEs.

In the case of subject who fails to attend scheduled visits, several attempts should be made by the investigator to contact the subject or one responsible relative or the subject's general practitioner for follow up information. It is only after sufficient unsuccessful attempts to contact the subject, that a subject may be declared "lost to follow-up". An excessive rate of withdrawals and/or subjects lost to follow-up can make the study uninterpretable; therefore, unnecessary subject attrition should be avoided. The attempts and their outcome should be documented in the medical notes of the subject.

In case the sponsor or investigator recommends discontinuation of treatment, the subject should continue attending the study visits as per the schedule of assessments.

6. STUDY TREATMENTS

6.1. Investigational Medicinal Product: **Durvalumab**

6.1.1. Drug information

INN: Durvalumab (MEDI4736)

Description: Durvalumab is a human immunoglobulin G1 kappa (IgG1κ) monoclonal antibody that targets PD-L1.

Mechanism of action: Durvalumab blocks the interaction of PD-L1 (but not PD-L2) with PD-1 on T cells and CD80+ immune cells and is engineered to reduce ADCC.

6.1.1.1. *Formulation*

Durvalumab will be supplied by AstraZeneca as a 500-mg vial solution for infusion after dilution. The solution contains 50 mg/mL durvalumab, 26 mM histidine/histidine-HCl, 275 mM trehalose dihydrate, 0.02% weight/volume (w/v) polysorbate 80; it has a pH of 6.0 and density of 1.054 g/mL. The label-claim volume is 10 mL. Durvalumab is a sterile, clear to opalescent, colorless to slightly yellow solution, free from visible particles.

6.1.1.2. *Preparation and administration*

The instructions for the preparation of durvalumab solution are found in the IMP management manual.

The dose of durvalumab for administration must be prepared by the investigator's or site's designated investigational product manager using aseptic technique.

Total time from needle puncture of the durvalumab vial to the start of administration must not exceed:

- 24 hours at 2°C to 8°C
- 4 hours at room temperature

If the final product is stored at both refrigerated and ambient temperatures, the total time must not exceed 24 hours.

6.1.1.3. *Therapeutic regimen and dose*

Durvalumab will be given at the fixed dose of 1500 mg IV every 4 weeks (Q4W).

Durvalumab IV infusion will start no less than 15 minutes after the end of oleclumab infusion (for subjects enrolled in the safety run-in and in arm 3 in the phase II) or will be the first study drug administration (for subjects enrolled in arm 2 in the phase II).

6.1.1.4. *Stability and storage*

Investigational product vials are stored at 2°C to 8°C and must not be frozen. Investigational product must be kept in original packaging until use to prevent prolonged light exposure

Durvalumab must be stored at the participating sites in a secure area in accordance with Good Clinical Practice (GCP) and Good Manufacturing Practice (GMP) requirements and must be accessible to authorised personnel only.

Storage conditions are found in the IMP management manual.

6.1.1.5. *Labelling*

Durvalumab is labelled according to the regulatory requirements of each country, as well as with the Rules Governing Medicinal Products in the European Union, Volume 4, EU Guidelines to Good Manufacturing Practice, Medicinal Products for Human and Veterinary Use, Annex 13, Investigational Medicinal Products.

6.1.1.6. *Drug logistics and accountability*

Durvalumab is provided to all participating sites labelled for investigational use only. The site acknowledges receipt of the IMP according to defined study procedure set up at study start. Any damaged shipment is replaced (if applicable). Accurate records of the IMP reception, dispensing, and destruction or return to the sponsor (if applicable) must be recorded on the Drug Accountability Form.

6.1.2. Toxicity

The adverse reactions observed for durvalumab can be found in the Investigator's brochure.

Please see section 7. Toxicity management and doses adjustments.

6.2. Investigational Medicinal Product: **Oleclumab**

6.2.1. Drug information

INN: Oleclumab (MEDI9447)

Description: Oleclumab is a human immunoglobulin G1 lambda (IgG1λ) monoclonal antibody (mAb) with a triple mutation in the heavy chain constant region for reduced effector function that selectively binds to and inhibits the ectonucleotidase activity of CD73.

Mechanism of action: Oleclumab inhibits the production of adenosine from AMP by CD73. Oleclumab relieves adenosine-mediated immunosuppression in the tumour microenvironment.

6.2.1.1. *Formulation*

Oleclumab will be supplied by AstraZeneca as a 500-mg vial solution for infusion after dilution. The solution contains 50 mg/mL oleclumab, 25 mM histidine/histidine hydrochloride, 240 mM sucrose, 0.03% (w/v) polysorbate 80; it has a pH of 6.0 and density of 1.05 g/mL. The label-claimvolume is 10 mL. Oleclumab is a sterile, clear to opalescent, colorless to yellow solution, that may contain a few white to off-white translucent particles.

6.2.1.2. *Preparation and administration*

The instructions for the preparation of oleclumab solution are found in the IMP management manual.

The dose of oleclumab for administration must be prepared by the Investigator's or site's designated IP manager using aseptic technique. Total time from needle puncture of the oleclumab vial to the start of administration must not exceed:

- 24 hours at 2°C to 8°C
- 4 hours at room temperature

If the final product is stored at both refrigerated and ambient temperatures, the total time must not exceed 24 hours.

6.2.1.3. *Therapeutic regimen and dose*

Oleclumab 3000 mg will be given IV every 2 weeks (Q2W) for the first 5 administrations then every 4 weeks (Q4W) for the last 2 administrations.

6.2.1.4. *Stability and storage*

Investigational product vials are stored at 2°C to 8°C and must not be frozen. Investigational product mustbe kept in original packaging until use to prevent prolonged light exposure.

Oleclumab must be stored at the participating sites in a secure area in accordance with GCP and GMP requirements and must be accessible to authorised personnel only.

Storage conditions are found in the IMP manual.

6.2.1.5. *Labelling*

Oleclumab is labelled according to the regulatory requirements of each country, as well as to the Rules Governing Medicinal Products in the European Union, Volume 4, EU Guidelines to

Good Manufacturing Practice, Medicinal Products for Human and Veterinary Use, Annex 13, Investigational Medicinal Products.

6.2.1.6. *Drug logistics and accountability*

Oleclumab is provided to all participating sites labelled for investigational use only. The site acknowledges receipt of the IMP defined study procedure set up at study start. Any damaged shipment is replaced (if applicable). Accurate records of the IMP reception, dispensing, and destruction or return to the sponsor (if applicable) must be recorded on the Drug Accountability Form.

6.2.2. Toxicity

The adverse reactions observed for oleclumab can be found in the Investigator’s brochure. *Please see section 7. Toxicity management and doses adjustments*.

6.3. Investigational Medicinal Product: **Paclitaxel**

6.3.1. Drug information

INN: Paclitaxel

Description: Paclitaxel is an antimicrotubule agent.

Mechanism of action: Paclitaxel promotes the assembly of microtubules from tubulin dimers and stabilises microtubules by preventing depolymerisation. This stability inhibits the normal dynamic reorganisation of the microtubule network, which is essential for vital interphase and mitotic cellular functions. In addition, paclitaxel induces abnormal arrays or bundles of microtubules throughout the cell cycle and multiple asters of microtubules during mitosis.

6.3.1.1. *Formulation*

The formulation of paclitaxel drug product to use is the one commonly available at the site pharmacy.

6.3.1.2. *Preparation*

The instructions for the preparation of paclitaxel solution are found in the package insert or in the Summary of Product Characteristics.

6.3.1.3. *Therapeutic regimen and dose*

Paclitaxel is given at a dose of 80 mg/m² IV once a week for 12 weeks.

6.3.1.4. *Stability and storage*

It must be stored at the participating sites in a secure area in accordance to GCP and GMP requirements and must be accessible to authorised personnel only.

Storage conditions are found in the package insert or in the Summary of Product Characteristics.

6.3.1.5. *Labelling*

Paclitaxel is labelled according to the regulatory requirements of each country, as well as to the Rules Governing Medicinal Products in the European Union, Volume 4, EU Guidelines to Good Manufacturing Practice, Medicinal Products for Human and Veterinary Use, Annex 13, Investigational Medicinal Products.

6.3.1.6. *Drug logistics and accountability*

Paclitaxel is taken from the commercial stocks of the hospital pharmacies and will be re-labelled by the pharmacists prior to providing them to the subjects. Accurate records of the IMP dispensing and destruction or return to the sponsor (if applicable) must be recorded on the Drug Accountability Form.

The completion of this log will not be mandatory if the site is having its own drug accountability system and is able to provide the lot numbers and expiry dates of the drug administered to a specific subject upon request (e.g. in case of audit, inspection or safety issue).

6.3.2. Toxicity

The adverse reactions observed for paclitaxel can be found in the Summary of Product Characteristics. *Please see section 7. Toxicity management and doses adjustments*.

6.4. Investigational Medicinal Product: **Doxorubicin**

6.4.1. Drug information

INN: Doxorubicin

Description: Doxorubicin is an anthracycline antibiotic.

Mechanism of action: The mechanism of action is not completely elucidated. It is postulated that doxorubicin hydrochloride exerts its antineoplastic effect via cytotoxic mechanisms of action especially intercalation into DNA, inhibition of the enzyme topoisomerase II, and formation of reactive oxygen species (ROS). All of these have a deleterious effect on DNA synthesis: Intercalation of the doxorubicin molecule leads to all inhibition of RNA and DNA polymerases by way of disturbances in base recognition and sequence specificity. The inhibition of topoisomerase II produces single and double strand breaks of the DNA helix. Scission of DNA also originates from the chemical reaction with highly reactive oxygen species like the hydroxyl radical $OH^{\bullet}$. Mutagenesis and chromosomal aberrations are the consequences.

6.4.1.1. *Formulation*

The formulation of doxorubicin drug product to use is the one commonly available at the site pharmacy.

6.4.1.2. *Preparation*

The instructions for the preparation of doxorubicin solution are found in the package insert or in the Summary of Product Characteristics.

6.4.1.3. *Therapeutic regimen and dose*

Dose-dense **doxorubicin is given at a dose of** 60 mg/m^2 IV every 2 weeks for 4 doses starting one week after the end of paclitaxel.

6.4.1.4. *Stability and storage*

It must be stored at the participating sites in a secure area in accordance with Good Clinical Practice (GCP) and Good Manufacturing Practice (GMP) requirements and must be accessible to authorised personnel only.

Storage conditions are found in the package insert or in the Summary of Product Characteristics.

6.4.1.5. *Labelling*

Doxorubicin is labelled according to the regulatory requirements of each country, as well as with the Rules Governing Medicinal Products in the European Union, Volume 4, EU Guidelines to Good Manufacturing Practice, Medicinal Products for Human and Veterinary Use, Annex 13, Investigational Medicinal Products.

6.4.1.6. *Drug logistics and accountability*

Doxorubicin is taken from the commercial stocks of the hospital pharmacies and will be re-labelled by the pharmacists prior to providing them to the subjects. Accurate records of the IMP dispensing and destruction or return to the sponsor (if applicable) must be recorded on the Drug Accountability Form.

The completion of this log is not mandatory if the site is having its own drug accountability system and is able to provide the lot numbers and expiry dates of the drug administered to a specific subject upon request (e.g. in case of audit, inspection or safety issue).

6.4.2. Toxicity

The adverse reactions observed for doxorubicin can be found in the Summary of Product Characteristics. *Please see section 7. Toxicity management and doses adjustments*.

6.5. Investigational Medicinal Product: **Cyclophosphamide**

6.5.1. Drug information

INN: Cyclophosphamide

Description: Cyclophosphamide is a cytostatic agent from alkylating agents group.

Mechanism of action: The active metabolites of cyclophosphamide are alkylating agents which transfer alkyl groups to DNA during the process of cell division, thus preventing normal synthesis of DNA.

6.5.1.1. *Formulation*

The formulation of cyclophosphamide drug product to use is the one commonly available at the site pharmacy.

6.5.1.2. *Preparation*

The instructions for the preparation of cyclophosphamide solution are found in the package insert or in the Summary of Product Characteristics.

6.5.1.3. *Therapeutic regimen and dose*

Cyclophosphamide is given at a dose of 600 mg/m^2 IV every 2 weeks for 4 doses starting one week after the end of paclitaxel.

6.5.1.4. *Stability and storage*

It must be stored at the participating sites in a secure area in accordance with Good Clinical Practice (GCP) and Good Manufacturing Practice (GMP) requirements and must be accessible to authorised personnel only.

Storage conditions are found in the package insert or in the Summary of Product Characteristics.

6.5.1.5. *Labelling*

Cyclophosphamide is labelled according to the regulatory requirements of each country, as well as with the Rules Governing Medicinal Products in the European Union, Volume 4, EU Guidelines to Good Manufacturing Practice, Medicinal Products for Human and Veterinary Use, Annex 13, Investigational Medicinal Products.

6.5.1.6. *Drug logistics and accountability*

Cyclophosphamide is taken from the commercial stocks of the hospital pharmacies and will be re-labelled by the pharmacists prior to providing them to the subjects. Accurate records of the IMP dispensing and destruction or return to the sponsor (if applicable) must be recorded on the Drug Accountability Form.

The completion of this log is not mandatory if the site is having its own drug accountability system and is able to provide the lot numbers and expiry dates of the drug administered to a specific subject upon request (e.g. in case of audit, inspection or safety issue).

6.5.2. Toxicity

The adverse reactions observed for cyclophosphamide can be found in the Summary of Product Characteristics. *Please see section 7. Toxicity management and doses adjustments*.

6.6. Non-Investigational Medicinal Products (NIMPs):

Information on formulation, preparation, stability, storage conditions and toxicity of NIMPs can be found in the package insert or in the Summary of Product Characteristics.

Treatment management and compliance are left to the discretion of the participating site.

6.6.1. G-CSF

G-CSF can be given to any subject at any time in fitting with the local site guidelines.

6.6.2. Chemotherapy pre- and post-medication

Chemotherapy pre- and post-medication including anti-emetics, antihistamines and steroids are administered according to local standard of care.

6.6.3. Gadolinium-based contrast agents

Gadolinium is a rare-earth based contrast that is injected intravenously before a subject undergoes MRI in order to improve the discernibility of lesions. Examples of gadolinium based contrast agents: gadoterate meglumine, gadobutrol, gadobenate dimeglumine.
About specific Gadolinium-based contrast agents it is important to note that the participating site should follow its own usual practices of agent dose and choice, as long as they are in accordance with the regulatory authorities' rulings and guidelines. In accordance with the final

opinion of the European Medicines Agency (EMA/625317/2017 date 23 Nov 2017) on use of linear gadolinium agents in body scan, we recommend, however that:

- Macrocyclic Gadolinium-based contrast agents should be favoured over linear agents;
- The use of Magnevist (gadopentetic acid), Omniscan (gadodiamide) and Optimark (gadoversetamide) should be avoided;
- The same Gadolinium-based contrast agent is used for each subject for all MRI scans
- Gadolinium-based contrast agents should be used in the lowest doses that enhance images sufficiently.

6.6.4. Iodinated contrast agent

Iodine is an iodinated-containing contrast agent used during the performance of CT scans. It is injected intravenously into subjects before exam performance and serves the purpose of increasing the capacity to discern lesions. Example of iodinated contrast agent: iomeprol.

6.7. Protocol surgery

6.7.1. Surgical treatment

Curative Breast surgery will be performed 2-6 weeks after the last cycle of ddAC. In case of delays due to toxicity, the surgery should be performed no later than 25 weeks from beginning of study treatment. Surgical technique is left at the discretion of the responsible surgeon. Tissue must be collected for analysis of endpoints, and therefore it is very important that the surgeon be made aware of subject status as a participant in the study and of necessary study procedures, namely that FFPE and frozen tissues will be collected (inselected sites also fresh material will be collected) from the surgical material left over, after sufficient and relevant parts has been retained to establish, improve or complement the diagnosis or treatment of the subject.

6.7.2. Surgery toxicity

Table 3: Surgical Adverse Reactions

Frequency	Surgical Adverse Reactions
Very frequent	Seroma
Frequent	Pain (burning, aching), phantom breast syndrome, arm morbidity (arm numbness, arm stiffness, shoulder pain, shoulder stiffness, nerve injury)
Infrequent	Wound infection, lymphedema

6.8. Protocol radiotherapy

6.8.1. Radiotherapy treatment

6.8.1.1. ***Stereotactic Body Radiotherapy (SBRT)*** *of the primary tumour in the breast.*

6.8.1.1.1 Treatment preparation:

- At least 3 marker clips, MRI-compatible, are placed in the tumour margin preceding systemic treatment.This should be performed at the same time of the tissue collection (study biopsy). The placement of marker clips is optional if the subject will be treated with an MRI-LINAC (linear accelerator with an onboard MRI), because the radiation oncologist can choose to use the MRI for tumour matching. The markers are to be placed close to the tumour margins and preferably cranial, caudal or laterally (left or right) of the tumour. Marker locations to avoid: anterior of the tumour (this is between the tumour and the skin) or posterior of the tumour (behind the tumour).
  The markers should be spread out in different directions of the tumours, it is to be avoided to insert two or more markers in the same location (for example: 1 marker cranial of the tumour, 1 marker caudal of the tumour and 1 lateral of the tumour is an optimal distribution; whereas 2 caudal markers and 1 cranial marker is to be avoided).
- A CT scan in treatment position with IV contrast enhancement will be performed in treatment position. CT slices with a maximum thickness of 3 mm, from the bottom of the mandible to several centimetres below the infra-mammary line, including both lungs entirely. IV contrast is mandatory, the only exceptions are medical contraindications for IV contrast (for example: contrast allergy or renal insufficiency).
- The treatment planning CT scan should be planned as close as possible and maximally 14 days preceding the start of SBRT treatment. Note that 14 days before SBRT treatment corresponds to week 2-3 of systemic treatment (figure 6).
- Supine subject positioning is preferred but may be done according to the local policy.
- Respiratory control techniques may be used according to the local policy but are not mandatory.
- A diagnostic MRI should be used to improve reliability of target volume contouring; the use of an MRI in treatment position is encouraged though not obligatory.

6.8.1.1.2 Contouring:

Please use the names as mentioned between brackets [Example] in the treatment planning system, to facilitate later planning analysis and comparison between subjects. Whenever possible, the structure names were defined according to international standardized naming conventions (94). Some structures names were not available in these publications and in these cases customised names were used.

Target volumes

- Gross Tumour Volume [**GTV**]: this should be determined taking into account all available information: the contrast-enhanced treatment planning CT; the localization on the mammogram, ultrasound, MRI (MRI spectroscopy and/or MRI elastography are optional) and PET-scan (optional), and the clips placed in the tumour. If a marker is clearly not inside the tumour, it can be opted to leave the marker out of the GTV.
- Clinical Target Volume [**CTV**] = GTV without supplementary margin.
- Planning Target Volume [**PTV**]: the participating centre should ideally individualise these margins based on own measures and techniques used such as IGRT (image guided radiation therapy), SGRT (surface guided radiation therapy) and MRI-LINAC. Given the use of at least 3 fiducials for CBCT matching is mandatory, we recommend a PTV margin

between 3 mm and 5 mm.

- Clips inside the PTV [**Clips_target**]: only contour the clips (markers) that are inside the PTV. Clips that fall outside the PTV will not be taken into account for CBCT matching and should therefore not be part of this structure.
- Clips outside the PTV [**Clips_non_target**]: clips outside the PTV.

Organs at risk

- Ipsilateral breast [**Breast_ipsi**]
- Contralateral breast [**Breast_contra**]
- Ipsilateral lung [**Lung_ipsi**]
- Contralateral lung [**Lung_contra**]
- Heart [**Heart**]: to be contoured according to Feng et al. (95).
- Oesophagus [**Oesophagus**]
- Trachea [**Trachea**]
- Lymph node regions: all lymph node regions should be contoured according to the ESTRO guidelines (96,97):
    - Internal mammary lymph nodes [**IMN**]
    - Axillary levels 1-4: **[L1]; [L2]; [L3]; [L4]**
    - Interpectoral nodes (Rotter): [**IP**]
- Involved lymph nodes [**LN_INV**]: lymph nodes suspected to be involved (based on PET/CT or MRI) are to be contoured as a GTV-LN, but since these nodes are not a target, the structure is named LN_INV.
- Body [**Body**], with special attention to the precision of the body contour in the region of the ipsilateral breast.

Additional structures to create

Structures for target plan evaluation

- **[PTV+30]** = [PTV] expanded with 30 mm.
- **[PTV_eval]** = [PTV] cropped to the skin with a 3 mm margin.
- **[GTV_eval]** = [GTV] cropped to the skin with a 3 mm margin.

OAR

- **[Skin-3]**: create by extracting a 3 mm inner wall from the [Body] contour, and crop to [PTV+30].
- **[Skin-5]**: create by extracting a 5 mm inner wall from the [Body] contour, and crop to [PTV+30].
- **[Skin+5]**: create by extracting a 5 mm outer wall from the [Body] contour and crop this structure to the [PTV+30].
- **[Skin-5]**: create by extracting a 5 mm inner wall from the [Body] contour and crop this structure to the [PTV+30].
- Ipsilateral breast minus PTV **[Breast_ipsi-PTV]** = [Breast_ipsi] – [PTV]
- Chest wall **[Chest_wall]**: defined as a 1 cm external contour outside the lungs. Crop structure to the [PTV+30]
- **[LN]** = Boolean combination of all lymph node areas (IMN, L1, L2, L3, L4, IP)
- **[LN+5]** = structure [LN] expanded with 5 mm
- **[Lungs]** = [Lung_ipsi] + [Lung_contra]
- **[Body-PTV]** = [Body] – [PTV]

Structures for CBCT matching

- **[Ring_skin]** = Boolean combination of [Skin+5] and [Skin-5]. This structure will be used during CBCT matching: see figure 8 CBCT matching algorithm. After matching on the clips, the skin should fall within the [Ring_skin] structure.
- **[Ring_lung]** = Boolean combination of 1) 5 mm outer wall of [Lung_ipsi] and 2) 5 mm inner wall of [Lung_ipsi]. Crop this structure to [PTV+30]. This structure will be used during CBCT matching: see figure 8 CBCT matching algorithm. After matching on the clips, the border of the lung should fall within the [Ring_lung] structure.
- **[Clips_target+5]** = [Clips_target] expanded with 5 mm in all directions.

Structures for plan evaluation of simulated swelling/shrinking of the breast (see further)

Simulated breast swelling:
- **[Swell_Body] =** [Body] + [Skin+5] (note that [Skin+5] should have been cropped to the [PTV+30])
- **[Swell_Skin-3]** = 3 mm inner wall from [Swell_Body]
- **[Swell_Skin-5]** = 5 mm inner wall from [Swell_Body]
- **[Swell_PTV_eval]** = [PTV] cropped to the [Swell_Body] with a 3 mm margin

Simulated breast shrinking:
- **[Shrink_Body] =** [Body] - [Skin-5] (note that [Skin-5] should have been cropped to the [PTV+30])

- **[Shrink_Skin-3]**= 3 mm inner wall from [Shrink_Body]
- **[Shrink_Skin-5]** = 5 mm inner wall from [Shrink_Body]
- **[Shrink_PTV_eval]** = [PTV] cropped to the [Shrink_Body] with a 3 mm margin
- **[Shrink_GTV_eval]** = [GTV] cropped to the [Shrink_Body]

Overview of names of corresponding structures in normal, swell and shrink simulations:

	NORMAL	SWELL 5mm	SHRINK 5mm
**Body**	Body	Swell_Body	Shrink_Body
**Skin 3 mm**	Skin-3<br>= skin 3 mm thick	**Swell**_Skin-3	**Shrink**_Skin-3
**Skin 5 mm**	Skin-5<br>= skin 5 mm thick	**Swell**_Skin-5	**Shrink**_Skin-5
**GTV_eval**	GTV cropped 3 mm to skin	*Idem normal*	**Shrink**_GTV_eval
**PTV_eval**	PTV cropped 3 mm to skin	**Swell**_PTV_eval	**Shrink**_PTV_eval

6.8.1.1.3 Planning

Dose prescription:

- The total dose is 24 Gy in 3 equal doses of 8 Gy, prescribed according to ICRU report 50, its supplement 62 and taking into account the specifications of report 91 when an SBRT technique is used (98).
- Scheduling of SBRT fractions: see section 3.2.3.1 for details and scheduling rules. The 3 fractions will preferably be given on 3 consecutive days but this is not mandatory to allow flexibily in scheduling over weekends and other scheduling constraints as detailed in section 3.2.3.1.

Biological context for physicists and dosimetrists concerning dose prescription and dose objectives:

The prescribed dose seems low in comparison to other SBRT treatments routinely performed in the clinic. However, there are important differences with other SBRT treatments to be kept in mind:

- The treatment will be given concomitantly with chemotherapy and immunotherapy, thereby changing classic NTCP (normal tissue complication probability) relationships. This combination is not given in routine with other SBRT treatments.
- A post-operative breast or chest wall radiation therapy (without boost) will be given +/- 20 weeks after the SBRT, further elevating the total dose.

Details on the full study treatment scheme are found in the study protocol.

A second element to keep in mind is that pre-clinical data revealed that the immune response is reduced when giving very high doses per fraction (> 10 Gy) (61), therefore we will impose stricter dose heterogeneity constraints than classic SBRT treatments.

Dose objectives and dose constraints

- At least 100% of [GTV_eval] should receive at least 95% of the prescribed dose.
- At least 95% of [PTV_eval] should receive at least 80% of the prescribed dose.
- The dose planning should aim at a steep dose fall-of outside of [PTV_eval] (with a resulting as low as possible dose in the non-target part of the breast). For this:
    - Dmax in all circumstances should be < 150% of the prescribed dose (<36 Gy).
    - All dose > 130% of the prescribed dose (31.2 Gy) should be located inside the PTV.
    - The maximal dose to a volume of 2 cc should not exceed 120% of the prescribed dose (28.8 Gy) – and this volume should be located within the CTV.
    - The maximal dose to a volume of 2 cc outside [PTV_eval] should not exceed 100% of the prescribed dose (24 Gy) [Body-PTV].
    - No dose > 62.5% of the prescribed dose (15 Gy) should be present at more than 1 cm from the GTV [GTV+1].
    - No dose > 50% of the prescribed dose (12 Gy) can be present at more than 3 cm from the PTV [PTV+3].

Dose constraints for organs at risk:

- General principle: the dose to normal tissues should be kept as low as possible.
- None of the treatment beams/arcs should enter or exit through the lymph nodes expanded by a margin of 5 mm [LN+5]. Exception: if unavoidable; the IM lymph node region could be excluded from this constraint.
- Skin [Skin]:
    - As low as possible

  - [Skin-3]: D0.1cc < 19.2 Gy
  - [Skin-5]: D10cc < 15 Gy
- Chest wall [Chest_wall]:
  - D15cc < 10 Gy
  - D1cc < 15 Gy
- Ipsilateral non-target breast [Breast_ipsi-PTV]
  - V24Gy < 30%
  - V15Gy < 60%
- Ipsilateral lung: [Lung_ipsi]
  - V20Gy < 2%
  - V10Gy < 10%
  - V5Gy < 20%
- Contralateral lung: [Lung_contra]
  - V20Gy < 1%
  - V10Gy < 2%
  - V5Gy < 3%
- Both lungs [Lungs]: mean lung dose max 5 Gy
- Heart [Heart]
  - V20Gy ≤ 1%
  - V10Gy ≤ 2%
  - V5Gy ≤ 5%
  - Mean heart dose ≤ 2 Gy
- Spinal cord [Spinal_cord]: D0.1cc < 14.6 Gy
- Contralateral breast [Breast_contra]: as low as possible, especially in women below 50 years of age; preferably below 1 Gy maximum dose in 1 cc. Mean dose, V5Gy and V1Gy should be recorded.
- Trachea and oesophagus: no constraint, dose as low as possible.

Priority principles between target and OAR

This is the order of priority for the constraints and target coverage goals:

1. Skin constraints
2. GTV coverage constraint
3. Chest wall constraint
4. PTV coverage constraint
5. Heart constraint
6. Lung constraint
7. Contralateral breast constraint
8. Ipsilateral non-target breast constraint

Simulation of possible swelling or size-reduction of the breast around the PTV

As mentioned above, the most important OAR for this treatment is the skin, because of potential concerns of toxicity resulting from the combination with chemotherapy and immunotherapy. Given the possible variability in the position of the skin resulting from clip-based matching (see next section) or due to volume changes of the breast, special attention is brought to simulate these changes in advance.

The treatment planning should therefore take into account these possible changes. At time of CBCT matching, we accept a deviation of 5 mm of the skin position, evaluated by checking if the skin is inside the control structures [Skin+5] and [Skin-5]. These two structures are limited to the volume PTV+30mm (see higher) and therefore do not apply to parts of the breast further away from the PTV.

The treatment plan should be pre-tested for these two possibilities by re-calculating the plan with two adapted dosimetry scans:

1. Simulation CT scan with water density applied to the structure [Skin+5].
2. Simulation CT scan with air density applied to the structure [Skin-5].

Conditions to pass both tests:

Important: use the corresponding [Shrink_] and [Swell_] structures to evaluate the PTV, GTV and skin. Instructions how to create these structures were given above.

- 95% of the GTV should be covered by at least 95% of the prescription dose.
- Underdosage of the PTV is accepted as long as the GTV is well covered.
- Overdosage of the PTV according to the dose conformality constraints as defined above is not accepted.
- All OAR contraints should be met. Especially skin constraints are important to be checked.

Treatment techniques:

Any technique that fulfils the above-defined dose objectives and dose constraints is accepted.

6.8.1.1.4 Imaging during treatment:

Daily CBCT matching is mandatory for every treatment fraction. CBCT matching will be done on the three or more clips implanted in the tumour according to image guided radiotherapy guidelines developed by Harris et al. for partial breast irradiation (99). **Figure 8:** *CBCT matching procedure*:

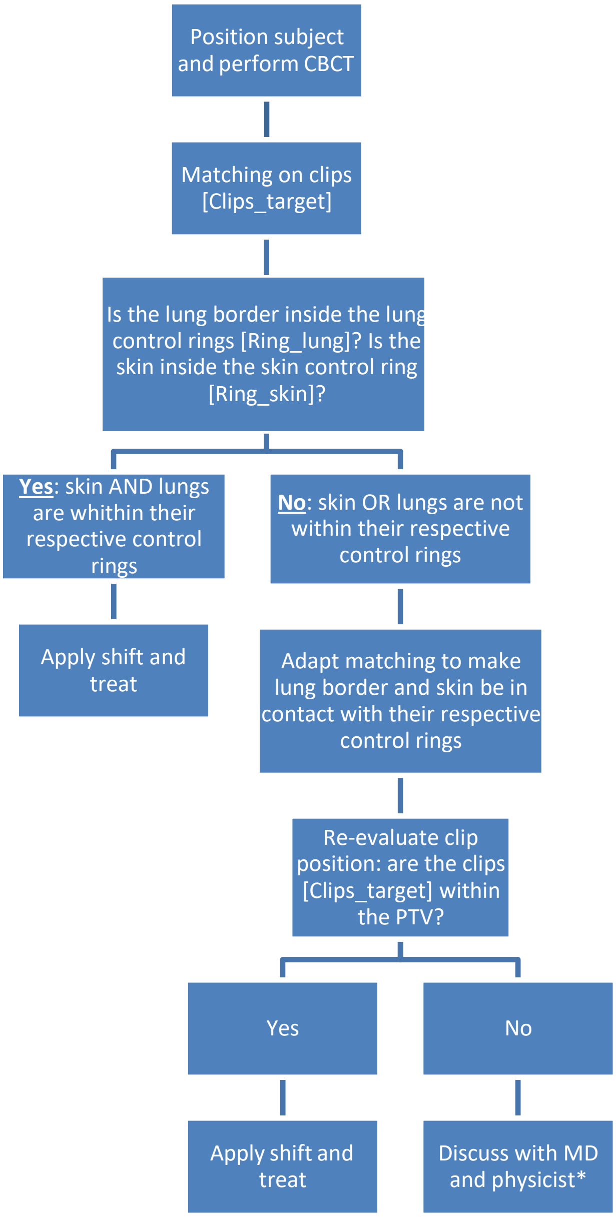

* MD and physicist to discuss and check:

- Evaluate why breast contour and/or lungs were outside control rings:
  - Oedema?
  - Inadequate positioning of the subject?
  - Has the subject moved between positioning and CBCT?
- Is there confusion due to a clip that should NOT be taken into account because far from tumour and therefore not part of the target? These clips were contoured as [Clip_non_target]. Identify and check the position these non-target clips.
- Are the clips inside the structure [Clips_target+5]? If so clip position can be accepted after reaching an optimal consensus between clips and control rings.

6.8.1.1.5 Quality assurance:

The pseudo-coded treatment plans of the first 3 subjects of each participating centre will be jointly reviewed by the radiation oncologist of the sponsor and the radiation oncologist of the study site responsible for the radiation oncology part.

6.8.1.1.6 Radiation therapy plan CRF

The radiation plan will be anonymised and sent to the study server for further analysis. Limited dosimetrical data will be asked on the study CRF because the plan itself will be analysed.

6.8.1.2. *Adjuvant radiotherapy*

Adjuvant radiotherapy should be given as per local guidelines and is not part of the study treatment. However, a boost to the tumour bed should not be given because the pre-operative SBRT is considered as an anticipated boost.

6.8.2. Radiotherapy toxicity

Radiotherapy toxicity	***Frequency***
**Dermatitis radiation grade 1**: faint erythema or dry desquamation	Very frequent
**Dermatitis radiation grade 2**: moderate to brisk erythema; patchy moist desquamation, mostly confined to skin folds and creases; moderate edema.	Frequent
**Pneumonitis grade 1:** asymptomatical; clinical or diagnostic observations only; intervention not indicated.	Frequent
**Breast fibrosis (tumour bed region) grade 1**	Frequent
**Dermatitis radiation grade 3**: moist desquamation in areas other than skin folds and creases; bleeding induced by minor trauma or abrasion.	Infrequent
**Pneumonitis grade 2:** symptomatic; medical intervention indicated; limiting instrumental ADL (activities of daily life)	Infrequent
**Breast fibrosis (tumour bed region) grade 2 or grade 3**	Infrequent
**Radiation recall reaction (dermatologic) grade 1:** faint erythema or dry desquamation.	Rare

Table 4: Radiotherapy Toxicity

Note: Grading of side-effects according to NCI-CTCAE v 5.

6.9. Contraception

6.9.1. Contraception

Female subjects of childbearing potential must agree to always use 1 highly effective method of contraception during the screening period, the course of this study and for at least 12 months after last study drugs administration.

The highly effective methods of contraception accepted are the following:

1) Nonhormonal intrauterine device (IUD)
2) Bilateral tubal occlusion
3) Vasectomised partner
4) Sexual abstinence *(relative to heterosexual activity) can be used as the sole method of contraception if it is consistently employed as the subject's preferred. Periodic abstinence (e.g., calendar, ovulation, sympto-thermal, post-ovulation methods) and withdrawal are not acceptable methods of contraception.*

Notes:

1. Female subjects of childbearing potential should seek advice regarding cryoconservation of oocytes *prior to treatment* because of the effect of the study drugs on fertility is unknown and shall refrain from donating oocytes for at least 12 months after the last administration.
2. Women should refrain from donating eggs *during the study* and for 12 months following the last dose of cyclophosphamide. Women should wait at least 12 months after stopping cyclophosphamide before attempting to conceive a child.
3. It is strongly recommended for the male partner of a female subject to also use male condom plus spermicide throughout this period.

6.10. Menopausal status

Post-menopausal status of subjects will be defined as follows:

- Age ≥60 years or,
- Age < 60 years and amenorrhea for 12 or more consecutive months in the absence of alternative pathological or physiological cause (including ovarian suppression) and FSH and serum estradiol levels within the laboratory's reference ranges for postmenopausal women.
- Prior bilateral oophorectomy (≥28 days prior to Day 1 of treatment)

Any subjects that are not post-menopausal, according to these criteria are considered to be pre-menopausal and should be treated as such.

6.11. Concomitant treatments

Concomitant therapy includes any medication (e.g., prescription drugs, over-the-counter drugs, herbal or homeopathic remedies, nutritional supplements) used by a subject from 28 days prior to initiation of study treatment to discontinuation visit (end of treatment visit). All such medications should be reported to the investigator (including start/stop dates, dose, indication and any other details as per CRF completion guideline) and recorded on the Concomitant Medications Case Report Form (CRF).

6.11.1. Allowed Concomitant treatments

The following therapies are permitted during study treatment:

- Prophylactic or therapeutic anticoagulation therapy (such as low−molecular weight heparin or warfarin at a stable dose level)
- Inhaled corticosteroids for chronic obstructive pulmonary disease
- Mineralocorticoids (e.g., fludrocortisone)
- Hydrocortisone of maximal 40 mg/day (or equivalent; e.g. prednisone 10 mg/day) used for adrenal insufficiency
- If clinically indicated, pre-medication with corticosteroids during first two weeks of paclitaxel
- Symptomatic treatment including acetaminophen, ibuprofen, promethazine, steroids and/or H2 receptor antagonists indicated for infusion-associated symptoms.
- Inactive influenza vaccinations during influenza season ONLY (approximately October to March)

All concomitant treatments medications and therapies (including start/stop dates, dose, indication and any other details as per CRF completion guideline) should be recorded in the CRF up to 30 days following the last administration of study treatment(s).

6.11.2. Prohibited Concomitant treatments

The following treatments and procedures are not allowed during the treatment period of the study and for at least 28 days prior to initiation of study treatment, unless otherwise specified below:

- Traditional herbal medicines; these therapies are not fully studied and their use may result in unanticipated drug-drug interactions that may cause or confound the assessment of toxicity
- Treatment with systemic steroids of more than the normal maximal dose of hydrocortisone (or equivalent) used for adrenal insufficiency (hydrocortisone 30 mg/day; prednisone 10 mg/day). Standard steroid premedication for paclitaxel treatment is allowed. Any other form of immunosuppressive agents (including but not limited to azathioprine, methotrexate, and thalidomide). These treatments need to have been stopped at least 14 days prior to the first dose of study medication. All immunosuppressive agents given as part of the study protocol (such as cyclophosphamide) are allowed.
- Chronic daily treatment with non-steroidal anti-inflammatory drug (NSAID) (occasional use for the symptomatic relief of medical conditions, for example, headache, fever is allowed)
- Treatment with systemic immunostimulatory agents, including but not limited to, interferon (IFN)-alpha, IFN-beta, interleukin (IL)-2, conjugated IL-2 cytokines within 42 days or five half-lives of the drug, whichever is longer, prior to screening
- Any live, attenuated vaccine during treatment, or within 28 days following the last dose of durvalumab/ oleclumab
- Treatment with anti−TNF-α agents.
- Subjects should not donate blood while participating in this study or for at least 90 days following the last infusion of durvalumab.

6.11.3. Incompatibilities/Drug Interactions

6.11.3.1. *Durvalumab*

No formal pharmacokinetics (PK) drug-drug interaction studies have been conducted with durvalumab because durvalumab is an Ig.

6.11.3.2. *Oleclumab*

No formal PK drug-drug interactions studies have been conducted with oleclumab because oleclumab is an Ig.

6.11.3.3. *Paclitaxel*

The metabolism of paclitaxel is catalysed, in part, by cytochrome P450 isoenzymes CYP2C8 and CYP3A4. Therefore, in the absence of a PK drug-drug interaction study, caution should be exercised when administering paclitaxel concomitantly with medicines known to inhibit either CYP2C8 or CYP3A4 (e.g. ketoconazole and other imidazole antifungals, erythromycin, fluoxetine, gemfibrozil, clopidogrel, cimetidine, ritonavir, saquinavir, indinavir, and nelfinavir) because toxicity of paclitaxel may be increased due to higher paclitaxel exposure. Administration of paclitaxel concomitantly with medicines known to induce either CYP2C8 or CYP3A4 (e.g. rifampicin, carbamazepine, phenytoin, efavirenz, nevirapine) is not recommended because it could result in lower paclitaxel exposures and efficacy may be compromised.

6.11.3.4. *Doxorubicin*

Doxorubicin cardiotoxicity is enhanced by previous or concurrent use of other anthracyclines, or other potentially cardiotoxic drugs (e.g. 5-fluorouracil, cyclophosphamide or paclitaxel) or with products affecting cardiac function (like calcium antagonists). When doxorubicin is used together with the above mentioned agents, cardiac function must be followed carefully.

Doxorubicin hepatotoxicity may be enhanced by other hepatotoxic treatment modalities (e.g. 6-mercaptopurine).

Doxorubicin undergoes metabolism via Cytochrome P450 (CYP450) and is a substrate for the Pgp transporter, Concomitant administration of inhibitors of CYP450 and/or Pgp might lead to increased plasma concentrations of doxorubicin and thereby increased toxicity. Conversely, concomitant administration of inducers of CYP450, such as rifampicin and barbiturates, might decrease plasma concentrations of doxorubicin and reduce efficacy.

Ciclosporin, an inhibitor of CYP3A4 and Pgp, increased the AUC of doxorubicin and doxorubicinol by 55% and 350%, respectively. The combination might require dose adjustment. Cimetidine has also been shown to reduce the plasma clearance and increase the AUC of doxorubicin.

Paclitaxel administered shortly before doxorubicin may decrease clearance and increase plasma concentrations of doxorubicin. Some data indicate that this interaction is less pronounced when doxorubicin is administered before paclitaxel.

Barbiturates may lead to an accelerated plasma clearance of doxorubicin, while the concomitant administration of phenytoin may result in lower plasma phenytoin levels.

Elevated serum doxorubicin concentrations were reported after the concomitant administration of doxorubicin and ritonavir.

The toxic effects of a doxorubicin therapy may be increased in a combination with other cytostatics (e.g. cytarabine, cisplatin, and cyclophosphamide). Necroses of the large intestine with massive haemorrhage and severe infections may occur in connection with combination therapies with cytarabine.

Clozapine may increase the risk and severity of the hematologic toxicity of doxorubicin.

Marked nephrotoxicity of Amphotericin B can occur during doxorubicin therapy.

As doxorubicin is rapidly metabolised and predominantly eliminated by the biliary system, the concomitant administration of known hepatotoxic chemotherapeutic agents (e.g.

mercaptopurine, methotrexate, streptozocin) could potentially increase the toxicity of doxorubicin as a result of reduced hepatic clearance of the drug.

Dosing of doxorubicin must be modified if concomitant therapy with hepatotoxic drugs is mandatory.

Doxorubicin is a potent, radio sensitizing agent ("radio sensitizer"), and recall phenomena induced by it may be life-threatening. Any preceding, concomitant or subsequent radiation therapy may increase the cardiotoxicity or hepatotoxicity of doxorubicin. This applies also to concomitant therapies with cardiotoxic or hepatotoxic drugs.

Doxorubicin may cause exacerbations of hemorrhagic cystitis caused by previous cyclophosphamide therapy.

Doxorubicin therapy may lead to increased serum uric acid, therefore dose adjustment of uric acid lowering agents may be necessary.

Doxorubicin may reduce oral bioavailability of digoxin.

During treatment with doxorubicin subjects should not be actively vaccinated and also avoid contact with recently polio vaccinated persons.

In a clinical study, an increase in doxorubicin AUC of 21% was observed when given with sorafenib 400 mg twice daily. The clinical significance of this finding is unknown.

6.11.3.5. *Cyclophosphamide*

<u>Interactions affecting the pharmacokinetics of cyclophosphamide and its metabolites</u>

- Reduced activation of cyclophosphamide may alter the effectiveness of cyclophosphamide treatment. Substances that delay activation of cyclophosphamide include:
    - Aprepitant
    - Bupropion
    - Busulfan: cyclophosphamide clearance has been reported to be reduced and half-life prolonged in subjects who receive high-dose cyclophosphamide less than 24 hours after high-dose busulfan.
    - Ciprofloxacin: When given prior to the treatment with cyclophosphamide (used for conditioning prior to bone marrow transplantation), ciprofloxacin has been reported to result in a relapse of the underlying disease.
    - Chloramphenicol
    - Fluconazole
    - Itraconazole
    - Prasugrel
    - Sulfonamides
    - Thiotepa: A strong inhibition of cyclophosphamide bioactivation by thiotepa in high-dose chemotherapy regimens has been reported when thiotepa was administered 1 hour prior to cyclophosphamide.
- An increase of the concentration of cytotoxic metabolites may occur with:
    - Allopurinol
    - Chloral hydrate
    - Cimetidine
    - Disulfiram
    - Glyceraldehyde
    - Inducers of human hepatic and extrahepatic microsomal enzymes (e.g., cytochrome P450 enzymes): The potential for hepatic and extrahepatic microsomal enzyme induction must be considered in case of prior or concomitant treatment with substances known to induce an increased activity

of such enzymes such as rifampin, phenobarbital, carbamazepine, phenytoin, St. John's wort, and corticosteroids.

- Protease inhibitors: concomitant use of protease inhibitors may increase the concentration of cytotoxic metabolites. Use of protease inhibitor-based regimens was found to be associated with a higher incidence of infections and neutropenia in subjects receiving cyclophosphamide, doxorubicin, and etoposide (CDE) than use of an NNRTI-based regimen.

• Ondansetron

There have been reports of a pharmacokinetic interaction between ondansetron and high-dose cyclophosphamide resulting in decreased cyclophosphamide AUC.

<u>Pharmacodynamic interactions and interactions of unknown mechanism affecting the use of cyclophosphamide</u>

Combined or sequential use of cyclophosphamide and other agents with similar toxicities can cause combined (increased) toxic effects.

• Increased hematotoxicity and/or immunosuppression may result from a combined effect of cyclophosphamide and, for example
- ACE inhibitors: ACE inhibitors can cause leukopenia.
- Natalizumab
- Paclitaxel: Increased hematotoxicity has been reported when cyclophosphamide was administered after paclitaxel infusion.
- Thiazide diuretics
- Zidovudine
- Clozapine

• Increased cardiotoxicity may result from a combined effect of cyclophosphamide and, for example
- Anthracyclines
- Cytarabine
- Pentostatin
- Radiation therapy of the cardiac region
- Trastuzumab

• Increased pulmonary toxicity may result from a combined effect of cyclophosphamide and, for example
- Amiodarone
- G-CSF, GM-CSF (granulocyte colony-stimulating factor, granulocyte macrophage colony-stimulating factor): Reports suggest an increased risk of pulmonary toxicity in subjects treated with cytotoxic chemotherapy that includes cyclophosphamide and G-CSF or GMCSF.

• Increased nephrotoxicity may result from a combined effect of cyclophosphamide and, for example
- Amphotericin B
- Indomethacin: Acute water intoxication has been reported with concomitant use of indomethacin.

• Increase in other toxicities
- Azathioprine: Increased risk of hepatotoxicity (liver necrosis)
- Busulfan: Increased incidence of hepatic veno-occlusive disease and mucositis has been reported.
- Protease inhibitors: Increased incidence of mucositis.

Other interactions

- Alcohol

  A reduced antitumour activity was observed in tumour-bearing animals during ethanol (alcohol) consumption and concomitant oral low-dose cyclophosphamide medication.

  In some subjects, alcohol may increase cyclophosphamide-induced vomiting and nausea.

- Etanercept

  In subjects with Wegener's granulomatosis, the addition of etanercept to standard treatment, including cyclophosphamide, was associated with a higher incidence of non-cutaneous solid malignancies.

- Metronidazole

  Acute encephalopathy has been reported in a subject receiving cyclophosphamide and metronidazole. Causal association is unclear.

  In an animal study, the combination of cyclophosphamide with metronidazole was associated with increased cyclophosphamide toxicity.

- Tamoxifen

  Concomitant use of tamoxifen and chemotherapy may increase the risk of thromboembolic complications.

<u>Interactions affecting the pharmacokinetics and/or actions of other drugs</u>

- Bupropion

  Cyclophosphamide metabolism by CYP2B6 may inhibit bupropion metabolism.

- Coumarins

  Both increased and decreased warfarin effect have been reported in subjects receiving warfarin and cyclophosphamide.

- Cyclosporine

  Lower serum concentrations of cyclosporine have been observed in subjects receiving a combination of cyclophosphamide and cyclosporine than in subjects receiving only cyclosporine. This interaction may result in an increased incidence of graft-versus-host disease.

- Depolarising muscle relaxants

  Cyclophosphamide treatment causes a marked and persistent inhibition of cholinesterase activity. Prolonged apnea may occur with concurrent depolarising muscle relaxants (e.g., succinylcholine). If a subject has been treated with cyclophosphamide within 10 days of general anesthesia, the anesthesiologist should be alerted.

- Digoxin, β-acetyldigoxin

  Cytotoxic treatment has been reported to impair intestinal absorption of digoxin and β-acetyldigoxin tablets.

- Vaccines

The immunosuppressive effects of cyclophosphamide can be expected to reduce the response to vaccination. Use of live vaccines may lead to vaccine-induced infection.

- Verapamil

Cytotoxic treatment has been reported to impair intestinal absorption of orally administered verapamil.

6.11.3.6. *Radiation Therapy*

- Paclitaxel

Radiation dermatitis

Paclitaxel or docetaxel administration concomitant with radiation therapy has been reported and the combination is considered safe (36,100–102). In esophageal cancer, neo-adjuvant treatment consisting of a combination of carboplatinum and paclitaxel weekly (50 mg per square meter of body-surface area) for 5 weeks concomitant with radiation therapy (41.4 Gy in 23 fractions, 5 days per week) is standard practice (100). This combination did not yield significant radiation therapy induced skin toxicity. In breast cancer, the concomitant combination of docetaxel or paclitaxel with radiation therapy has also been tested. Bellon et al. demonstrated that acute skin toxicity was less pronounced using concomitant paclitaxel instead of docetaxel: 10% grade 3 toxicity with paclitaxel and 42% grade 3 toxicity with docetaxel (102). Subjects in this study received a median dose of 50 Gy in 25 fractions. As mentioned previously, Bondiau et al, delivered 3x8.5 Gy to the primary tumour concomitantly with *docetaxel*, with no subjects developing a grade 3 radiation dermatitis at that dose level. In the present study, 3x8 Gy will be delivered concomitantly with *paclitaxel*, taking into account the Bellon study and the Bondiau study, we do not expect a significant number of grade 3 radiation dermatitis.

Radiation recall phenomenom

Radiation recall reaction (or radiation recall phenomenon) is an uncommon and unpredictable phenomenon that is characterised by an acute inflammatory reaction confined to skin areas previously treated with radiation therapy and that is triggered by the administration of precipitating systemic agents (usually chemotherapy) after the radiation treatment (103). Recall reactions with paclitaxel and doxorobucin have been described in the literature but are very rare (104,105). In the Bondiau study no recall reaction was described when docetaxel was given concomitantly with radiation therapy. Only one subject developed a recall reaction, but this subject received concomitant doxorubicin instead of concomitant docetaxel. In the present study, SBRT will be given concomitantly with paclitaxel.

- Durvalumab

6.12. Study treatment duration

Study treatment duration is from the first pre-operative systemic treatment until surgery. Surgery should be performed 2-6 weeks after the last pre-operative systemic treatment

7. TOXICITY MANAGEMENT AND DOSE ADJUSTMENTS

7.1. General management of study drug delays

Important rules:

- No immunotherapy is given after full completion of the planned chemotherapy.
- In the event of immune related adverse events leading to holding or discontinuation of immunotherapy, both durvalumab and oleclumab should be held or discontinued.

	**Chemotherapy**	**Immunotherapy**
HOLDING		
When to hold?	Discussed in section 7.3	Discussed in section 7.2
What to do when deciding to hold?	Continue immunotherapy if no indications to hold immunotherapy.	Continue chemotherapy if no indications to hold immunotherapy.
Interval between administrations after holding.	Keep interval as planned.	Keep interval as planned.
What to do with a missed treatment (treatment not given because of holding)?	Planned chemotherapy may be given later after holding to keep the number of initially planned treatments unchanged. This is not mandatory and the site is allowed to opt to skip the planned treatment.	Planned immunotherapy may be given later after holding to keep the number of initially planned treatments unchanged. This is not mandatory and the site is allowed to opt to skip the planned treatment.<br>However, immunotherapy should be discontinued once all planned chemotherapy has been given or chemotherapy has been discontinued.
DISCONTINUE		
When to discontinue?	• If chemotherapy has been held for 4 weeks or more.<br>• In case of toxicity mandating discontinuation as discussed section in 7.3.	• If complete schedule of chemotherapy has been given.<br>• If chemotherapy has been discontinued.
What to do when deciding to discontinue?	• If Paclitaxel is discontinued: switch to ddAC<br>• If ddAC is discontinued: plan surgery	Stop immunotherapy. Continue chemotherapy as planned.

Table 5: Chemotherapy delays

7.2. Durvalumab and oleclumab

7.2.1. Immune-related Adverse Events

Immune-mediated or related AEs (im or irAEs) may occur after the first or several months after the last dose of durvalumab/oleclumab. Table 6 describes the general guidance for corticosteroid management of irAEs based on the recommendations from the Society for Immunotherapy of Cancer (SITC) (106). The management of specific immune-related adverse

events and when to withhold, discontinue and re-initiate durvalumab is described in the current version of the **durvalumab toxicity management guidelines for irAEs in the Appendix 1**.

The management of specific immune-related adverse events for oleclumab will follow the same current durvalumab Toxicity Management Guidelines.

Please note that these guidelines are not specific to the Neocheckray protocol and include information related to the management of Tremelimumab toxicity that is not applicable to the Neocheckray study.

After 12 weeks of treatment interruption for irAE, rechallenge with durvalumab +/- oleclumab should be discussed with the sponsor.

Note: Consider calcium and vitamin D supplementation with prolonged steroid use. All subjects receiving steroids should also be on proton pump inhibitor therapy for gastrointestinal prophylaxis.

General guidance for corticosteroid management of immune-related adverse events

Grade of immune-related AE (CTCAE/equivalent)	Corticosteroid management	Additional notes
1	• Corticosteroids not usually indicated	• Continue immunotherapy
2	• If indicated, start oral prednisone 0.5-1 mg/kg/day if patient can take oral medication.<br>• If IV required, start methylprednisolone 0.5-1 mg/kg/day IV<br>• If no improvement in 2–3 days, increase corticosteroid dose to 2 mg/kg/day<br>• Once improved to ≤grade 1 AE, start 4–6 week steroid taper	• Hold immunotherapy during corticosteroid use<br>• Continue immunotherapy once resolved to ≤grade 1 and off corticosteroids<br>• Start proton pump inhibitor for GI prophylaxis
3	• Start prednisone 1-2 mg/kg/day (or equivalent dose of methylprednisolone)<br>• If no improvement in 2–3 days, add additional/alternative immune suppressant<br>• Once improved to ≤ grade 1, start 4–6-week steroid taper<br>• Provide supportive treatment as needed	• Hold immunotherapy; if symptoms do not improve in 4–6 weeks, discontinue immunotherapy<br>• Consider intravenous corticosteroids<br>• Start proton pump inhibitor for GI prophylaxis<br>• Add PCP prophylaxis if more than 3 weeks of immunosuppression expected (>30 mg prednisone or equivalent/day)
4	• Start prednisone 1-2 mg/kg/day (or equivalent dose of methylprednisolone)<br>• If no improvement in 2–3 days, add additional/alternative immune suppressant, e.g., infliximab<br>• Provide supportive care as needed	• Discontinue immunotherapy<br>• Continue intravenous corticosteroids<br>• Start proton pump inhibitor for GI prophylaxis<br>• Add PCP prophylaxis if more than 3 weeks of immunosuppression expected (>30 mg prednisone or equivalent/day)

Note: For steroid-refractory cases and/or when steroid sparing is desirable, management should be coordinated with disease specialists. AE, adverse event

Table 6 General guidance for Immune-related Adverse Events

7.2.2. Infusion-related reactions

Infusion-related reactions (IRRs) can be observed with both durvalumab and oleclumab.

In case of a Grade 2 IRR that resolves within one hour of stopping the drug infusion and adequate treatment according to local standard of care, the infusion may be restarted at 50% of the original infusion rate. In case of Grade 3-4 IRR, the study drug has to be discontinued permanently.

NCI CTCAE Grade	Management	Premedication at subsequent dosing
Grade 1	The infusion rate may be decreased by 50% or temporarily interrupted.<br>Increase monitoring of vital signs until the subject is deemed medically stable in the opinion of the investigator.	None
Grade 2	Stop infusion and monitor symptoms<br>Additional medical therapy may include but is not limited to<br>• IV fluids<br>• Antihistamines	Premedication may include<br>• Antihistamines<br>• Promethazine/ diphenhydramine<br>• Paracetamol

NCI CTCAE Grade	Management	Premedication at subsequent dosing
	• Hydrocortisone/ other corticosteroids<br>• NSAIDS<br>• Paracetamol<br>• Narcotics<br>Increase monitoring of vital signs until the subject is deemed medically stable in the opinion of the investigator.<br>If symptoms resolve within 1 hour of stopping drug infusion, the infusion may be restarted at 50% of the original infusion rate. Otherwise, dosing will be held until symptoms resolve and the subject should be premedicated for the next scheduled visit.	• NSAIDS<br>Steroids should not be used for routine premedication of Grade ≤ 2 infusion reactions<br><br>Subjects who develop Grade 2 toxicity despite adequate premedication should be permanently discontinued from further study treatment administration.
Grade 3-4	Stop infusion<br>Additional appropriate medical therapy may include but is not limited to:<br>• IV fluids<br>• Antihistamines<br>• Hydrocortisone/ other corticosteroids<br>• NSAIDS<br>• Paracetamol<br>• Narcotics<br>• Oxygen<br>• Pressors<br>• Epinephrine<br>Increase monitoring of vital signs until the subject is deemed medically stable in the opinion of the investigator.<br>Hospitalisation may be indicated.	Permanently discontinue durvalumab/oleclumab

Table 7 Management of IRRs to durvalumab/oleclumab

7.3. Chemotherapy

Chemotherapy administration (paclitaxel and ddAC) may be interrupted due to AEs for a maximum of 4 weeks. If the subject does not meet the re-treatment criteria within this time period, chemotherapy has to be discontinued permanently. In case of treatment interruption ≤ 4 weeks, treatment with chemotherapy can continue.

Suggested dose modifications for chemotherapy are detailed in the table hereunder.

Table 8 Suggested Dose Modifications for Chemotherapy

	Start Dose	Dose Reduction 1	Dose Reduction 2	Dose Reduction 3
**Paclitaxel**	80 mg/m^2	65 mg/m^2	50 mg/m^2	Discontinue
**Doxorubicine**	60 mg/m^2	50 mg/m^2	40 mg/m^2	Discontinue
**Cyclophosph amide**	600 mg/m^2	500 mg/m^2	400 mg/m^2	Discontinue

7.3.1. Paclitaxel

7.3.1.1. *Suggested treatment decisions when paclitaxel must be held or discontinued (**Table 9**)*

Notes : • The following treatment decisions are *suggestions* and not mandatory. Because chemotherapy is standard of care in this study, investigators can follow local guidelines. • Dose modifications must be based on AEs that occurred between treatments (column 2) and AEs present on the scheduled treatment day (column 3). • Dose modifications must be based on the AE requiring the greatest modification.		
**CTCAE v5.0 Adverse Event/Grade**	**Modifications for AEs that occurred during a cycle but RESOLVE PRIOR TO THE NEXT TREATMENT CYCLE** (See footnote a)	**Modifications for AEs that REQUIRE A DELAY IN ADMINISTRATION OF THE TREATMENT CYCLE** (See footnote b)
**<u>Neutrophil count decreased:</u>** Grades 2, 3, 4	Maintain dose	**ANC:** *Hold until ≥ 1000/mm^3. If recovery takes:* 1-3 wks – maintain dose and add G-CSF[c] *If receiving G-CSF and recovery takes:* 1 wk – maintain dose; 2-3 wks – ↓ one dose level
**<u>Platelet count decreased:</u>** Grade 2, 3	Maintain dose	*Hold until ≥ 75,000/mm^3. If recovery takes:* 1 wk – maintain dose; 2 to 3 wks – ↓ one dose level
Grade 4	↓ one dose level	*Hold until ≥ 75,000/mm^3.* ↓ one dose level
**<u>GI (if related to chemotherapy):</u>** **Diarrhea** Grade 2	Maintain dose	Maintain dose or ↓ one dose level
Grade 3	↓ one dose level	↓ one dose level
Grade 4	↓ one dose level or discontinue treatment permanently	Discontinue treatment permanently
**Mucositis oral** Grade 2	Maintain dose	Maintain dose or ↓ one dose level
Grade 3	↓ one dose level	↓ one dose level
Grade 4	↓ one dose level or Discontinue treatment permanently	Discontinue treatment permanently

**Vomiting** *(despite antiemetics)* Grade 2	Maintain dose or ↓ one dose level	Maintain dose or ↓ one dose level
Grades 3, 4	↓ one dose level or Discontinue treatment permanently	Discontinue treatment permanently
**<u>Hepatic:</u> Bilirubin, AST/ALT, alk phos** Grade 2	↓ one dose level	*Hold until bilirubin returns to the **baseline grade** and AST and alk phos have returned to ≤ grade 1;* ↓ one dose level
Grade 3	↓ one dose level or Discontinue treatment permanently	*Hold until bilirubin returns to the **baseline grade** and AST and alk phos have returned to ≤ grade 1;* ↓ one dose level or discontinue treatment permanently
Grade 4	Discontinue treatment permanently	Discontinue treatment permanently
**<u>Cardiac</u>** **Cardiac conduction abnormalities (e.g. atrioventricular block) or cardiac arrhythmias (e.g. atrial fibrillation, atria flutter or bradycardia)** Grade 2	Maintain dose *Continuous cardiac monitoring during subsequent therapy with paclitaxel.*	Maintain dose *Continuous cardiac monitoring during subsequent therapy with paclitaxel.*
Grades 3 and 4	Discontinue treatment permanently	Discontinue treatment permanently
Grade 4	Discontinue treatment permanently	Discontinue treatment permanently
**<u>Vascular disorders</u>** <u>(hypotension or hypertension)</u> Grade 2	Vital signs monitoring, particularly during the first hour of paclitaxel infusion.	Vital signs monitoring, particularly during the first hour of paclitaxel infusion
Grade 3	Discontinue treatment permanently	Discontinue treatment permanently
Grade 4	Discontinue treatment permanently	Discontinue treatment permanently

**Infection or febrile neutropenia:**<br>Grade 2 (N/A for febrile neutropenia)	Maintain dose and add G-CSF[c] prophylaxis for subsequent chemotherapy administrations if neutropenia was present.[d]	
Grade 3	Maintain dose and add G-CSF[c] prophylaxis with subsequent chemotherapy administrations. If receiving prophylactic G-CSF, ↓ one dose level.	
Grade 4	Maintain dose and add G-CSF[c] prophylaxis with subsequent chemotherapy administrations. If receiving prophylactic G-CSF, ↓ one dose level.	
**Other clinically significant AEs:**<br>Grade 2	Maintain dose or ↓ one dose level	
Grade 3	↓ one dose level	↓ one dose level or discontinue treatment permanently
Grade 4	↓ one dose level or discontinue treatment permanently	Discontinue treatment permanently
**a** Treatment may not proceed until clinically significant AEs are ≤ grade 1 (except neutrophils, which must be ≥ 1000/mm^3, and bilirubin, which must be ≤ the baseline grade).<br>**b** Hold and check weekly. With exception of neutrophils and bilirubin, resume treatment when toxicity is ≤ grade 1. If toxicity has not resolved to ≤ grade 1 after 3 weeks of delay, discontinue paclitaxel.<br>**c** If grade 2 criteria for infection include topical and/or systemic antibiotics or other local treatment, use of G-CSF is at the investigator's discretion.<br>**d** Determination of "clinically significant" AEs is at the discretion of the investigator		

7.3.1.2. *Treatment management for paclitaxel-related neuropathy* (***Table 10***)

**Nervous System Disorders**<br>• Paraesthesia<br>• Peripheral sensory neuropathy	**1–7 Days Duration**	**Persistent for> 7 Days *or* Caused the Next Treatment to be Delayed**
**Grade 1**	Maintain paclitaxel dose	
**Grade 2**	Maintain paclitaxel dose[a]	Decrease paclitaxel one dose level[b]
**Grade 3**	**First episode:** Decrease paclitaxel one dose level[a]<br>**Second episode:** Discontinue paclitaxel	Discontinue paclitaxel
**Grade 4**	Discontinue paclitaxel	

**a** Must be resolved to ≤ grade 1 on the next treatment day.<br>**b** Hold paclitaxel for ***persistent*** grade 2 neuropathy. When ≤ grade 1, resume treatment with dose modification for paclitaxel. If grade 2 toxicity persists after 3 weeks of delay, discontinue paclitaxel.

7.3.1.3. *Treatment management for paclitaxel-related musculoskeletal pain (**Table 11)***

Note: The treatment management instructions below apply to subjects with **musculoskeletal pain not controlled by analgesics**. Use of narcotics and NSAIDs is encouraged to maintain the paclitaxel dose if possible.

**Musculoskeletal and Connective Tissue Disorders**<br>• Arthralgia<br>• Myalgia	**1–7 Days Duration**	**Persistent for> 7 Days or Caused the Next Treatment to be Delayed**
**Grade 1** (despite analgesics)	Maintain paclitaxel dose	
**Grade 2** (despite analgesics)	Maintain paclitaxel dose	Maintain paclitaxel dose or Decrease paclitaxel one dose level*
**Grade 3** (despite analgesics)	**First episode:** Decrease paclitaxel one dose level<br>**Second episode:** Discontinue paclitaxel	**First episode:** Decrease paclitaxel one dose level* or Discontinue paclitaxel<br>**Second episode:** Discontinue paclitaxel
* Hold paclitaxel for ***persistent*** grade 2 or 3 musculoskeletal pain. When ≤ grade 1, resume treatment with dose modification. If grade 2 or grade 3 toxicity persists after 3 weeks of delay, discontinue paclitaxel.		

7.3.2. ddAC

7.3.2.1. *Cardiac AEs during ddAC (**Table 12**)*

If the subject develops any of the following cardiac AEs during AC, study therapy should be discontinued:

• Any ≥ grade 3 AE listed in the Cardiac Disorders section of the CTCAE v5.0.

• Any ≥ grade 2 cardiac AE defined as follows:

CARDIAC DISORDERS – CTCAE v5.0	
Adverse Event Grade 2 Criteria	Adverse Event Grade 2 Criteria
Acute coronary syndrome	Symptomatic, progressive angina; cardiac enzymes normal; hemodynamically stable
Chest pain - cardiac	Moderate pain; limiting instrumental ADL

Heart failure	Symptoms with mild to moderate activity or exertion
Myocardial infarction	Asymptomatic and cardiac enzymes minimally abnormal and no evidence of ischemic ECG changes
Myocarditis	Symptoms with mild to moderate activity or exertion
Pericarditis	Symptomatic pericarditis (e.g., chest pain)
Right ventricular dysfunction	Symptoms with mild to moderate activity or exertion
Ventricular tachycardia	Non-urgent medical intervention indicated

7.3.2.2. *Non-cardiac toxicity*

• If ddAC is discontinued due to non-cardiac toxicity, surgery should be scheduled regardless of immunotherapy completion.

7.3.2.3. *Treatment management for ddAC (**Table 13**)*

**<u>Important table instructions:</u>**<br>•The following treatment decisions are *suggestions* and not mandatory. Because chemotherapy is standard of care in this study, investigators are allowed to follow local guidelines.<br>• Dose modifications should be based on AEs that occurred during the cycle (column 2) and AEs present on the scheduled Day 1 of Administrations 2-4 (column 3).<br>• All modifications in dose levels apply to both doxorubicin and cyclophosphamide (AC).<br>• Dose modifications should be based on the AE requiring the greatest modification.<br>• **Primary prophylaxis with G-CSF is strongly recommended.**		
CTCAE v4.0 Adverse Event/Grade	Modifications for AEs that occurred during a cycle but RESOLVE PRIOR TO THE NEXT TREATMENT CYCLE (See footnote **a**)	Modifications for AEs that REQUIRE A DELAY IN ADMINISTRATION OF THE TREATMENT CYCLE (See footnote **b**)
**Neutrophil count decreased:**<br>Grades 2 (1000-1500/mm^3), 3, 4	Maintain dose	***ANC:*** Hold until ≥ 1200/mm^3. If recovery takes: 1-3 wks – maintain dose and add G-CSFc If receiving G-CSF and recovery takes: 1 wk – maintain dose; 2-3 wks – ↓ one dose level
**Platelet count decreased:** Grades 2, 3	Maintain dose	Hold until ≥ 75,000/mm^3. If recovery takes: 1 wk – maintain dose; 2 to 3 wks – ↓ one dose level

Grade 4	↓ one dose level	Hold until ≥ 75,000/mm^3. ↓ one dose level
**GI (if related to chemotherapy): Diarrhea** Grade 2	Maintain dose	Maintain dose or ↓ one dose level
Grade 3	↓ one dose level	↓ one dose level
Grade 4	↓ one dose level or discontinue treatment permanently	Discontinue treatment permanently
**Mucositis oral** Grade 2	Maintain dose	Maintain dose or ↓ one dose level
Grade 3	↓ one dose level	↓ one dose level
Grade 4	↓ one dose level or discontinue treatment permanently	Discontinue treatment permanently
**Vomiting (despite antiemetics)** Grade 2	Maintain dose or ↓ one dose level	Maintain dose or ↓ one dose level
**Hepatic: Bilirubin, AST/ALT, alk phos** Grade 2	↓ one dose level	Hold until bilirubin returns to the ***baseline grade*** and AST and alk phos have returned to ≤ grade 1; ↓ one dose level
Grade 3	↓ one dose level or discontinue treatment permanently	Hold until bilirubin returns to the ***baseline grade*** and AST and alk phos have returned to ≤ grade 1; ↓ one dose level or discontinue treatment permanently
Grade 4	Discontinue treatment permanently	Discontinue treatment permanently
**Infection or febrile neutropenia:** Grade 2 (N/A for febrile neutropenia)	Maintain dose and add G-CSF[c] prophylaxis for subsequent chemotherapy administrations if neutropenia was present.[d]	
Grade 3	Maintain dose and add G-CSF[c] prophylaxis with subsequent chemotherapy administrations. If receiving prophylactic G-CSF, ↓ one dose level.	
Grade 4	Maintain dose and add G-CSF[c] prophylaxis with subsequent chemotherapy administrations. If receiving prophylactic G-CSF, ↓ one dose level or discontinue treatment permanently .	
**Other clinically significant AEs:**[e]	Maintain dose or ↓ one dose level	

Grade 2		
Grade 3	↓ one dose level	↓ one dose level
Grade 4	↓ one dose level or discontinue treatment permanently	Discontinue treatment permanently

a Resolved means that all clinically significant AEs are ≤ grade 1 (except neutrophils, which must be ≥ 1200/mm^3, and bilirubin, which must be ≤ the baseline grade) on Day 1 of the next scheduled cycle (i.e., treatment can be given without delay).

b Hold and check weekly. ***With exception of neutrophils and bilirubin, resume treatment when toxicity is ≤ grade 1.*** If toxicity has not resolved after 3 weeks of delay, discontinue AC and proceed to paclitaxel.

c Pegfilgrastim, at a fixed dose of 6 mg SQ on Day 2, is preferred. Filgrastim, if used, should be administered according to the drug package insert.

d If grade 2 criteria for infection include topical and/or systemic antibiotics or other local treatment, use of G-CSF is at the investigator's discretion.

e Determination of "clinically significant" AEs is at the discretion of the investigator.

7.3.3. Infusion-related Reactions (IRRs)

Hypersensitivity to paclitaxel is common. While symptoms usually develop within the first 10-15 minutes of paclitaxel infusion and occur in nearly 90% of the cases during the first or second drug infusion. To avoid allergic reactions, premedication with antihistamines and corticosteroids is mandatory and should be followed as per local standard of care. The table hereunder outlines the management of allergic reactions with paclitaxel and/or ddAC.

Table 14 Management of IRRs to Paclitaxel or ddAC

NCI CTCAE Grade	Treatment	Premedication at subsequent dosing
Grade 1	Infusion interruption not indicated<br>Increase monitoring of vital signs until the subject is deemed medically stable in the opinion of the investigator.	According to local standard of care.
Grade 2	Stop infusion and monitor symptoms<br>Additional medical therapy may include but is not limited to<br>• IV fluids<br>• Antihistamines<br>• Hydrocortisone<br>• NSAIDS<br>• Paracetamol<br>• Narcotics<br>Increase monitoring of vital signs until the subject is deemed medically stable in the opinion of the investigator.<br>If symptoms resolve within 1 hour of stopping drug infusion, the infusion may be restarted at 50% of the original infusion rate. Otherwise, dosing will be stopped.	According to local standard of care.
Grade 3-4	Stop infusion<br>Additional appropriate medical therapy may include but is not limited to<br>• IV fluids<br>• Antihistamines<br>• Hydrocortisone/ other corticosteroids<br>• NSAIDS<br>• Paracetamol<br>• Narcotics<br>• Oxygen<br>• Pressors<br>• Epinephrine<br>Increase monitoring of vital signs until the subject is deemed medically stable in the opinion of the investigator.<br>Hospitalisation may be indicated.<br>Subject is permanently discontinued from further administration of the respective drug.	No subsequent dosing of the respective drug.

7.4. Radiation therapy

Table 15

TOXICITY	GRADE	DESCRIPTION	MANAGEMENT
**Radiation dermatitis**	1	Faint erythema or dry desquamation	Application of hydroactive colloid gel (example: Flamigel or Bepanthol).
	2	Moderate to brisk erythema; patchy moist desquamation, mostly confined to skin folds and creases; moderate edema	Application of soft silicone dressing (example: Mepilex Lite) in combination with gel administered for grade 1 radiation dermatitis or with an enzyme alginogel (example: Flaminal Hydro)
	3	Moist desquamation in areas other than skin folds and	Application of absorbing soft silicone dressing (example: Mepilex or Mepilex

TOXICITY	GRADE	DESCRIPTION	MANAGEMENT
		creases; bleeding induced by minor trauma or abrasion	XT) in combination with enzyme alginogel (example: Flaminal Hydro).
	4	Life-threatening consequences; skin necrosis or ulceration of full thickness dermis; spontaneous bleeding from involved site; skin graft indicated	Continue local treatment as in grade 3 radiation dermatitis with the addition of systemic treatment by hydrocortisone or other corticosteroids and NSAIDS.
**Radiation pneumonitis**	All	See section 7.1.1.	Identical to management of corresponding grade of pneumonitis caused by durvalumab and oleclumab: see section 7.1.1.

8. SCHEDULE OF ASSESSMENTS

This is for both the safety run-in and Phase II.

8.1. Screening period

During the screening period, following signature of the Informed Consent Form, the principal investigator confirms the subject's eligibility for the study by conducting the assessments described below and complete the corresponding CRF upon enrolment/randomisation in the study.

All screening evaluations are to be performed within 28 days before enrolment/randomisation except if described differently in the text.

The evaluations and procedures to be conducted in the screening period include the following:

- Date of ICF signature (as soon as the ICF signed, the subject should be registered in the study via Subject Registration Tool = step 1)
- Demographic data: date of birth
- Eligibility check: review of inclusion and exclusion criteria (eligible subject to be enrolled/randomised in Subject Registration Tool = step 2)
- Diagnosis: Female subjects with non-metastatic, luminal B breast cancer (ER+/HER2-, grade III and/or Ki67≥15%), histologically confirmed carcinoma of NST or ILC with disease measuring at least 1.5 cm (except for cT4), as determined by MRI, who are candidate for neo-adjuvant treatment
- Biopsy in order to provide new tumour tissue.
    - cN+ subjects (node positive subjects at diagnosis) only: biopsy (or fine needle aspiration) of the positive lymph node if easily accessible (OPTIONAL)
- Placement of MRI-compatible markers (clips): 3 markers to be placed around the tumour.
    - The markers are preferably placed at the same time of the tissue collection during screening.
    - The markers are to be placed close to the tumour margins and preferably cranial, caudal or laterally (left or right) of the tumour. Marker locations to avoid: anterior of the tumour (this is between the tumour and the skin) or posterior of the tumour (behind the tumour). The markers should be spread out in different directions of the tumours, it is to be avoided to insert two or more markers in the same location (for example: 1 marker cranial of the tumour, 1 marker caudal of the tumour and 1

lateral of the tumour is an optimal distribution; whereas 2 caudal markers and 1 cranial marker is to be avoided).

Placement of markers is mandatory for all subjects. The only exception is if the subject will be treated on an MRI-LINAC (linear accelerator with an onboard MRI): in this case the placement of the markers are optional.

- MammaPrint genomic high risk score according to centralised testing. MammaPrint will only be tested for luminal B breast tumours with either Proliferation Index Ki67 ≥ 15% or histology grade III tumours. (Testing to be done during screening period).

MammaPrint result status at time of termination of all other screening procedures:

- *MammaPrint is high risk*: subject may be randomized.
- *MammaPrint is low risk*: subject can not be randomized.
- *MammaPrint result is not yet known*:

If the MammaPrint result is not known at time of termination of all other screening procedures, the investigator is allowed to randomize the subject and start study treatment without waiting for the result of the MammaPrint in the following situations:

<table>
<tr><th colspan="2"></th><th>Age < 50 years</th><th>Age ≥ 50 years</th></tr>
<tr><td rowspan="2">Ki67 ≤ 20% and grade II</td><td>cN0</td><td colspan="2" rowspan="2">Wait for MammaPrint result</td></tr>
<tr><td>cN+</td></tr>
<tr><td rowspan="2">Ki67 > 20 % or grade III</td><td>cN0</td><td>Allowed not to wait for MammaPrint result</td><td>Wait for MammaPrint result</td></tr>
<tr><td>cN+</td><td colspan="2">Allowed not to wait for MammaPrint result</td></tr>
</table>

If the subject is randomized in a situation without known MammaPrint result and the MammaPrint result proves to be low risk after randomisation, the subject will stay in the study.

- *MammaPrint result is unevaluable or is technically impossible*:

The sponsor should be contacted as soon as possible to discuss the inclusion of the concerned subject. Under specific medical conditions and breast cancer disease characteristics, the medical team of the sponsor can accept that the site continues the screening process and randomization of the subject. There will be maximum 5% of non-evaluable MammaPrint results in this group of 'MammaPrint unevaluable'.

- Confirmed tumour PD-L1 IHC assessment according to central testing for stratification purposes (only for phase II). **Note**: In case the PD-L1 test returns an unevaluable result or is technically impossible, the concerned subject can still be randomized, however analysis of a second sample is preferred if possible.
- Medical history: clinically significant prior and/or current medical conditions (including assessed inherited mutations in breast cancer related genes (such as BRCA1, BRCA2, and others) and surgical procedures and menopausal status.
- Cancer history and prior cancer therapies specifying name of therapy/procedure, total cumulative dose, date of treatment and number of cycles administered when available for each past treatment.

- Concomitant medications, therapies and procedures from 28 days prior to the first administration of study treatment.
- Medical consultation including:
  - Physical examination (body weight, height, body surface area (DuBois method).
  - Vital signs (body temperature, blood pressure, pulse (heart rate) and respiratory rate)
  - ECOG performance status
- Breast appearance evaluation
  - Evaluation of breast cosmetic: breast fibrosis in the whole breast, breast fibrosis around the tumour (corresponding to the future boost area), breast size, breast shape, nipple position, shape of the areola and nipple, skin color, evaluation of teleangiectasia and global cosmetic result.
  - Breast photography (according to the breast photography guidelines (89–91,107)
- Baseline cardiac assessment
  - Cardiac-focused history
  - Physical cardiac examination
  - Electrocardiogram (ECG)
  - LVEF assessment by echocardiogram or multigated acquisition (MUGA) scan. The same method should be used through the study.
- Laboratory tests:
  - Complete blood count (CBC) and analysis should be performed: red blood cell count (RBC), haemoglobin (Hb), platelet count and white blood cell count (WBC). WBC must include differential count
    - Chemistry panel: glucose, creatinine, eGFR (according to institutional standard of care), sodium, potassium, magnesium, chloride, bicarbonate, calcium, phosphorus, total serum bilirubin, ALT, AST, ALP, LDH, total protein, albumin CRP,thyroid function test (thyroid-stimulating hormone [TSH], T4) and lipase.
  - Serum pregnancy test for all female subjects of childbearing potential (within 2 weeks prior to first dose of study administration).
  - Coagulation: aPTT and INR
  - Serology:
    - HBV serology (HBsAg, antibody to HBsAg [anti-HBs], anti-HBc). HBV DNA testing is only required if the subject has negative serology for HBsAg and positive serology for anti-HBc
    - HCV serology. HCV-RNA testing is only required if the subject has positive serology.
- Radiological examinations:
  - Breast MRI
    - The requirement for an MRI is not applicable in the case of medical contraindications to perform MRI (e.g., obesity or claustrophobia). In this situation, tumour evaluations should be performed by ultrasound.

- Breast MRI is accepted if performed within 2 months before enrolment.

- SAEs related to protocol mandated intervention.

  Results of all screening evaluations and procedures must be reviewed by the principal investigator to ensure that all eligibility criteria have been satisfied prior to subject enrolment.

  Screen failure is defined as any subject that signed the ICF and finally is not enrolled /randomised in the study.

8.2. Study treatment period

8.2.1. Chemotherapy in combination or not with immunotherapy and SBRT (= 19 weeks).

A maximum of 1 working day is allowed between subject's enrolment/randomisation and first study drug administration.

The following evaluations and procedures have to be performed **every 2 weeks during safety run in** and at **weeks 1, 3, 5, 7, 9, 13, and 17 during the Phase II study treatment period, except if described differently in the text below:**

- Treatment administration
- Concomitant medications, therapies and procedures.
- Medical consultation including: (within 3 days prior to study drug administration)
  - Physical examination (body weight, body surface area (DuBois method)).
  - Physical cardiac examination
  - Vital signs (body temperature, blood pressure, pulse (heart rate) and respiratory rate)
  - ECOG performance status
- Laboratory tests (within 3 days prior to study drug administration)
  - CBC including RBC, Hb, platelet count, WBC (including differential count)
    Chemistry panel: glucose, creatinine, sodium, potassium, magnesium, chloride, bicarbonate, calcium, phosphorus, total serum bilirubin, ALT, AST, ALP, LDH, total protein, albumin thyroid function testing (thyroid-stimulating hormone [TSH], T4),CRP (not requested at week 1) and lipase.

At week 1: For subjects of arms 2 and 3 only: Cortisol and ACTH blood sampling preferably at 08:00 in the morning.

- Coagulation tests: aPTT and INR every 8 weeks ± 1 week.
- All AEs including SAEs documented according to NCI-CTCAE v 5.0., and AESIs, pregnancies, adverse reactions due to overdose, abuse, misuse and medication error, as described in the section 10.2 Pharmacovigilance parameters definitions and reporting guidance.

  The following evaluations and procedures have to be performed at specific time points during the study treatment duration.

- Monthly urine pregnancy test for all female subjects of childbearing potential (if positive to be confirmed by serum) within 3 days prior to study drug administration

- Samples collection:
  - **Blood** samples:
    - Week 1 (before treatment start)
    - Weeks 4, 6, 12, and end of chemo (week 19 if no treatment delay), 1st immune related AE and 1st Disease Progression.
  - **Tissue** samples:
    - Week 6:
      - All subjects: biopsy of the primary breast cancer
      - cN+ subjects (node positive patients at diagnosis) only: biopsy (or fine needle aspiration) of the positive lymph node if easily accessible (OPTIONAL)
      - The week 6 biopsy should be taken 5-12 days after the SBRT, this corresponds to week 6. However, if SBRT was delayed, the week 6 biopsy should be delayed as well in order that the biopsy is taken 5-12 days after the SBRT.
    - 1st Disease Progression (optional)

      Important notes:

      The on-treatment tumour biopsy obtained at week 6 should be performed at the same site as the pre-treatment biopsy. Marking and identifying the pre-treatment biopsy site will follow local practice methods.
- Test procedures:
  - During week 11 or 12 obligatory prior to start of ddAC:
    - Breast MRI
      - In case the breast MRI reveals a potential tumour progression, a new breast MRI is to be planned within 4-6 weeks to exclude pseudo-progression.

        The requirement for an MRI is not applicable in the case of medical contraindications to perform MRI (e.g., obesity or claustrophobia). In this situation, tumour evaluations should be performed by ultrasound.
    - **For subjects of arms 2 and 3 only**:
      - Electrocardiogram (ECG)
      - LVEF assessment by echocardiogram or MUGA scan

  Note: breast MRI and cardiac evaluations of week 11-12 should be performed before ddAC treatment, hence these evaluations should be scheduled earlier if the ddAC is given early (for example after early discontinuation of paclitaxel).

- SBRT: Radiotherapy anticipated boost that is given in three fractions over minimum 3 days and maximum 6 days at Week 5. Detailed rules about SBRT scheduling are given in section 3.2.3.1.

  Important note : the treatment planning CT scan should be planned as close as possible and maximally 14 days preceding start of SBRT treatment.

14 days before SBRT treatment corresponds to week 2-3 of systemic treatment (figure 6).

- Breast cosmetic evaluation at week 9 +/- 1 week (= 1-3 weeks following SBRT) and between week 19-23 (= 2 weeks before surgery).
    - Evaluation of breast cosmetic: breast fibrosis in the whole breast, breast fibrosis around the tumour (corresponding to the future boost area), breast size, breast shape, nipple position, shape of the areola and nipple, skin color, evaluation of teleangiectasia and global cosmetic result.
    - Breast photography (according to the breast photography guidelines (89–91,107)

8.2.2. Surgery

Surgery should be performed within 2-6 weeks after the last dose of chemotherapy. A visit should be performed within 3 days before surgery except if described differently in the following text. The surgery should be performed at the earliest week 21 and at the latest week 25.

- Concomitant medications, therapies and procedures.
- Medical consultation including:
    - Physical examination (body weight)
    - Physical cardiac examination
    - Vital signs (body temperature, blood pressure, pulse (heart rate) and respiratory rate)
    - ECOG performance status
- Urine pregnancy test for all female subjects of childbearing potential (if positive to be confirmed by serum).
- Laboratory tests
    - CBC including RBC, Hb, platelet count, WBC (including differential count)
    - Chemistry panel: glucose, creatinine, sodium, potassium, magnesium, chloride, bicarbonate, calcium, phosphorus, total serum bilirubin, ALT, AST, ALP, LDH, total protein, albumin, CRP, Thyroid function testing (thyroid-stimulating hormone [TSH], T4) and lipase.
    - Coagulation: aPTT and INR
    - For subjects of arms 2 and 3 only: Cortisol and ACTH blood sampling, preferably at the same time than the cortisol and ACTH sampling baseline (section 8.1), recommended at 08:00 AM.

- Cardiac evaluations**:**
    Electrocardiogram (ECG) within 1 week before surgery or the day of surgery, but should be done before surgery.
    - LVEF assessment by echocardiogram or MUGA scan (echocardiogram is preferred) within 1 week before surgery (can be done the day of the surgery but not after surgery)
- All AEs including SAEs documented according to NCI-CTCAE v 5.0., and AESIs, pregnancies, adverse reactions due to overdose, abuse, misuse and medication error, as

described in the section 10.2 Pharmacovigilance parameters definitions and reporting guidance.

- Surgery
  - Important note: Information on cosmetic and plastic surgical procedures need to be documented (for example: oncoloplastic surgery, breast implants, and other procedures whenever occurs).
  - Report :
    - RCB and variables correlated to RCB
    - Standard of care anatomo-pathological results, including Ki67, tumor grade, ER, PR, HER2+ status, and other information available on the pathological report of the surgical sample.
- Samples collection:
  - Blood samples: day of surgery or at the same time as the laboratory tests required at this visit
  - Tissue samples: day of surgery
  - cN+ subjects (node positive subjects at diagnosis) only: biopsy (or fine needle aspiration) of the positive lymph node if easily accessible (OPTIONAL)

  Important note: the post-treatment tumour biopsy obtained at the time of definitive surgery should be performed at the same site as the pre-treatment biopsy. Marking and identifying the pre-treatment biopsy site will follow local practice methods.

8.3. End of study treatment visit

An end of treatment (EOT) visit should be carried out **30 days (± 7 days) after the surgery** to perform the following evaluations and procedures.

The following should be performed:

- Concomitant medications, therapies and procedures.
- Medical consultation including:
  - Physical examination (body weight).
  - Physical cardiac examination
  - Vital signs (body temperature, blood pressure, pulse (heart rate) and respiratory rate)
  - ECOG performance status
- Urine pregnancy test for all female subjects of childbearing potential (if positive to be confirmed by serum).
- Laboratory tests:
  - CBC including RBC, Hb, platelet count, WBC (including differential count)
  - Chemistry panel: glucose, creatinine, sodium, potassium, magnesium, chloride, bicarbonate, calcium, phosphorus, total serum bilirubin, ALT, AST, ALP, LDH, total protein, albumin CRP, thyroid function testing (thyroid-stimulating hormone [TSH], T4),and lipase.
  - Coagulation: aPTT and INR
- Samples collection:

- Blood samples: End of Study Treatment Visit. As well, if subject has 1st immune related AE and/ or 1st Disease Progression,
- Tissue samples: 1st Disease Progression (optional)

- All AEs including SAEs documented according to NCI-CTCAE v 5.0., AESIs, and pregnancies as described in the section 10.2 Pharmacovigilance parameters definitions and reporting guidance

8.4. Follow-up period

A follow-up visit has to be performed: 1) 0-24 months after surgery: every 3 months +/- 2 weeks; 2) 25-36 months after surgery: every 6 months +/- 4 weeks 3) 37-60 months after surgery:yearly +/- 4 weeks. During this time the following evaluations and procedures are conducted:

- Update changes in medical history: clinically medical conditions (including assessed inherited mutations in breast cancer related genes (such as BRCA1, BRCA2, and others), and surgical procedures.
- Medical consultation including:
  - Physical examination (body weight).
  - Physical cardiac examination
  - Vital signs (body temperature, blood pressure, pulse (heart rate) and respiratory rate)
  - ECOG performance status
- Pregnancy:
  - Urine pregnancy test for all female subjects of childbearing potential (if positive to be confirmed by serum) only the first 4 follow-up visits.
  - If a pregnancy occurs during follow-up period, under the consent of the subject previously obtained, the following information will be collected: spontaneous menstruation recovery, pregnancy outcome, offspring outcomes, pregnancy attempts, breastfeeding, use of conception method(assisted reproductive technology (ART) or spontaneous).

- Laboratory tests:
  - CBC including RBC, Hb, platelet count, WBC (including differential count)
  - Chemistry panel: glucose, creatinine, sodium, potassium, magnesium, chloride, bicarbonate, calcium, phosphorus, total serum bilirubin, ALT, ALP, LDH, total protein, albumin thyroid function testing (thyroid-stimulating hormone [TSH], T4) and lipase.
  - Coagulation: aPTT and INR

- SAEs, AESIs and pregnancies as described in section 10.2. Pharmacovigilance parameters definitions and reporting guidance
- Breast cosmetic evaluation:
  - Evaluation of breast cosmetic and changes: at 3 months after surgery, then at 1 year after surgery and then yearly. The following will be evaluated: changes in breast appearance: breast fibrosis in whole breast, breast fibrosis in boost area, breast size, breast shape, nipple position, shape of the areola and nipple, skin color, appearance of surgical scar, evaluation of teleangiectasia and global cosmetic result .

    - Breast photography (according to the breast photography guidelines (89–91,107) at 3 months after surgery, 1 year after surgery and then yearly.

- Evaluation of local control of breast and lymph nodes: timing and choice of technical modality (bilateral mammography and/or ultrasound and/or breasts MRI) can be done according to local practice and based on subject characteristics. The first evaluation scheduled according to the local practices should be repeated every year.
- Samples collection:
    - Blood samples:
        - At 1 year, 2 years, 3 years and 5 years after surgery.
        - At the 1st immune related AE
        - At the 1st Disease Progression
    - Tissue samples: If subject has 1st Disease Progression, an optional sample can be collected.

8.5. End of study visit

The last Follow up visit, performed at month 60 (+/- 4 weeks) or earlier if the subject leaves the study, will serve as the End of Study visit.

8.6. Schedule of assessments: Phase II

Docusign Envelope ID: 8E3E1A28-D1DF-4A9A-A65F-9548F273A2D6

Evaluations	Screening 28 days before enrolment	Study Treatment Period Chemotherapy + SBRT +/- Immunotherapy																				
		W 1	W 2	W 3	W 4	W5	W6	W 7	W 8	W 9	W 10	W 11	W12	W 13	W 14	W 15	W 16	W 17	W 18	W 19	Surgery W21-25	EOT Visit [1]
ICF signature	X																					
Eligibility	X																					
Selection of the index lesion in case of multifocal or bilateral breast cancer if applicable (see Appendix 2)	X																					
Tumour tissue central review for eligibility MammaPrint (10 slides)	X																					
Tumour tissue central review for stratification PD-L1 (4 to 6 slides)	X																					
**Clinical assessments**																						
Past medical and cancer history [2]	X																					
Concomitant medication, therapies & procedures	X	X[20]																				
Physical examination, Vital signs, ECOG PS	X	X		X		X		X		X				X				X			X	X
Breast cosmetic evaluation	X									X [3]										X [4]		
Breast photography	X									X [3]										X [4]		
Adverse Events (AEs) reporting		X continuously (from 1st administration of study treatment until 60 days after surgery)																				
Serious Adverse Events (SAEs) reporting [5]	X	X continuously (from 1st administration of study treatment until 60 days after surgery)																				
**Laboratory tests**																						
Pregnancy test: serum/urine	X (serum) [6]	X [7]				X [7]				X [7]				X [7]				X [7]			X [7]	X [7]
Laboratory tests [8]	X	X		X		X		X		X				X				X			X	X
Serology (Hepatitis B and C)	X																					
Cortisol and ACTH; preferably at 08:00 am **only for Arms 2 & 3**		X																			X [9]	
Blood sample for translational research		X			X		X[10]						X							X	X [11]	X [12]
**Tissue samples**																						
Biopsy of the primary tumour (1 FFPE + 2 frozen tissues and in selected sites only: 1 fresh tissue also asked)	X						X[10]														X	X [13]
Biopsy of a at diagnosis positive lymph node (optional)	X						X[10]														X	
**Cardiac evaluation**																						
Electrocardiogram (ECG)	X												X[14]								X [9]	
LVEF assessment (Echocardiogram or MUGA scan)	X												X[14]								X [9]	
**Imaging and markers placement**																						
Placement of MRI-compatible markers [15]	X																					
Breast MRI	X[16]												X[17]									
**Treatment administration**																						
Paclitaxel		X	X	X	X	X	X	X	X	X	X	X	X									
ddAC														X		X		X		X		
SBRT (3 fractions)						X[18]																
Durvalumab		X				X				X				X				X				

Docusign Envelope ID: 8E3E1A28-D1DF-4A9A-A65F-9548F273A2D6

**only for Arms 2 & 3**																						
Oleclumab<br>**only for Arm 3**		X		X		X		X		X				X				X				
Surgery																					X [19]	

1.) To take place 30 days (± 7 days) after surgery.

2.) Includes contraception methods.

3.) To be performed at week 9 (± 1 week; =1-3 weeks following SBRT).

4.) To be performed between week 19-23 (=2 weeks before surgery).

5.) From IC signature up to 1st study drugs administration, only SAEs related to protocol-mandated intervention have to be reported. From 1st study drugs administration up to 60 days after surgery, all AEs & SAE have to be reported. From 61 days after surgery, only SAEs related to study drugs must be reported.

6.) Serum at baseline for woman of childbearing potential. Max 14 days prior first administration

7.) Monthly for woman of childbearing potential. Urine, if positive to be confirmed with serum test.

8.) Haematology, biochemistry, coagulation function test (every 8 weeks ± 1 week: aPTT and INR), thyroid function test, CRP (not requested at week 1 nor FU visits), liver function test, lipase.

9.) Within 1 week before surgery (can be done the day of surgery but before surgery).

10.) To be done at week 6 (day 5-12 after SBRT). If SBRT was delayed, the biopsy should be taken between day 5 and 12 after SBRT.

11.) Collected Day of Surgery.

12.) A blood sample for translational research should be taken at the 1st immune related toxicity and 1st disease progression, if those events occur.

13.) A tissue sample for translational research could be taken (optional sample) at the 1st disease progression, if this occurs.

14.) Only applicable for arms 2 and 3.

15.) 3 markers (clips) to be placed in the tumour. This should be performed at the same time of the tissue collection. Mandatory (optional if subject is treated on an MRI-LINAC).

16.) The requirement for an MRI is not applicable in the case of medical contraindications to perform MRI (e.g., obesity or claustrophobia), in this case an ultrasound evaluation of the breast is required instead of the MRI. Breast MRI is accepted if performed within 2 months before enrolment.

17.) To be performed during week 11 or 12: obligatory prior to start of ddAC. The requirement for an MRI is not applicable in the case of medical contraindications to perform MRI (e.g., obesity or claustrophobia), In this situation, tumour evaluations should be performed by ultrasound. .

18.) SBRT in 3 fractions should be spread at the minimum over 3 days and at the maximum over 6 days. The 3rd fraction of SBRT will take place the day before or on the same day as the 5th administration of paclitaxel (arms 1, 2 and 3), the 2nd administration of durvalumab (arms 2 and 3) and the 3rd administration of oleclumab (arm 3). If SBRT is given on the same day than the study medication, the SBRT should be given prior to any study medications.

19.) Surgery performed 2- 6 weeks after completion of ddAC. See section 8.2.2. for details on reporting of anatomo-pathological analysis of the surgical specimen.

Important note: Information on cosmetic and plastic surgical procedures need to be documented (for example: oncoloplastic surgery, breast implants, and other procedures whenever occurs).

20.) And from EOT until 60 days after surgery if related to AE/SAE

8.7. Schedule of assessments: Phase II – Follow-up Period

Evaluations	Follow-up Period [1]											
	1st year				2nd year				3rd year		4th year	5th year
	M3	M6	M9	M12	M15	M18	M21	M24	M30	M36	M48	M60
**Clinical assessments**												
Update medical history (including BRCA1/BRCA2 status if known)	X	X	X	X	X	X	X	X	X	X	X	X
Physical examination, Vital signs, ECOG PS[1]	X	X	X	X	X	X	X	X	X	X	X	X
Breast cosmetic evaluation [2]	X			X				X		X	X	X
Breast photography [3]	X			X				X		X	X	X
Adverse Events (AEs) reporting [4]	X											
Adverse Events of Special Interest (AESI) reporting[10]	X	X	X	X	X	X	X	X	X	X	X	X
Serious Adverse Events (SAEs) reporting	X	X	X	X	X	X	X	X	X	X	X	X
Concomitant medication, therapies & procedures[11]	X											
Recurrence / new cancer assessment				X				X		X	X	X
Survival reporting	X											
Pregnancy outcomes reporting								X		X		X
**Laboratory tests**												
Pregnancy test: serum/urine [5]	X	X	X	X								
Laboratory tests [6]	X	X	X	X	X	X	X	X	X	X	X	X
Blood sample for translational research				X				X		X		X
Blood sample for translational research in case of event [7]	X											
**Tissue samples**												
Biopsy in case of progression disease (1FFPE + 2 frozen tissues and in selected sites only: 1 fresh tissue also asked) [8]	X (optional)											
**Imaging**												
Radiological evaluation of breast and lymph nodes [9]	X (every year)											
**Survival**												
Survival reporting	X											

1) 0-24 months after surgery: every 3 months +/- 2 weeks; 25-36 months after surgery: every 6 months +/- 4 weeks; 37-60 months after surgery: yearly +/- 4 weeks2) At 3 months after surgery, then at 1 year (+/- 2 weeks) and yearly (± 4weeks); analysis of cosmetic result of treated breast.

3) Breast photographs to be taken at 3 months, 1,2, 3, 45 years (+/- 4 weeks) after surgery.

4) Continuously until 60 days after sugery, all AEs should be reported. After this period, all AEs that are immune-related and/ or endocrine-related and/or cardiovascular-related and/or radiation therapy-related should be reported on the AE page of the CRF. See section 10.2.1 for details on AE reporting.5) For all subjects of childbearing potential (if positive to be confirmed by serum) only the first 4 follow-up visits.

6) Haematology, biochemistry, coagulation function test, thyroid function test, liver function test, lipase.

7) A blood sample for translational research should be taken at the 1st immune related toxicity and 1st progressive disease, if those occur.

8) A tissue sample for translational research could be taken (optional sample).Performed at first local or distant progression prior to the next anti-cancer therapy, if this occurs.

9) Timing and choice of technical modality (bilateral mammography and/or ultrasound and/or breast MRI) can be done according to local practice and based on subject characteristics. The first evaluation schedule according to local practices should be repeated every year.

10) AESI grade ≥ 2 for durvalumab and all AESIs for oleclumab should be reported in the CRF and on SAE form. AESI of grade 1 for durvalumab should be reported in the CRF only. See section 10.2.4 and 10.2.5 for details on AESI reporting.

11) Only necessary if related to reported AE, AESI or SAE.

9. ASSESSMENT OF EFFICACY

Efficacy is assessed using different objectives and endpoints as defined in section 2, the schedule of assessments (section 8) and the statistical plan (section 12).

10. ASSESSMENT OF SAFETY

10.1. Safety parameters and Methods for assessing safety parameters

The following safety parameters are assessed:

- Demographic data (for details please refer to the paragraph “Schedule of Assessments”)
- Medical history (for details please refer to the paragraph “Schedule of Assessments”)
- Cancer history (for details please refer to the paragraph “Schedule of Assessments”)
- Prior cancer treatment (for details please refer to the paragraph “Schedule of Assessments”)
- Concomitant medications (for details please refer to the paragraph “Schedule of Assessments”)
- Physical examination and vital signs (for details please refer to the paragraph “Schedule of Assessments”)
- ECOG performance status score (for details please refer to the paragraph “Schedule of Assessments”)
- Laboratory tests (for details please refer to the paragraph “Schedule of Assessments”)
- Test procedures (for details please refer to the paragraph “Schedule of Assessments”)
- Adverse Events (AEs) (for details please refer to the paragraph “Pharmacovigilance parameters definitions and reporting guidance”)
- Adverse Event of Special Interests (AESIs) (for details please refer to the paragraph “Pharmacovigilance parameters definitions and reporting guidance”)
- Serious Adverse Events (SAEs) (for details please refer to the paragraph “Pharmacovigilance parameters definitions and reporting guidance”)
- Pregnancy (for details please refer to the paragraph “Pharmacovigilance parameters definitions and reporting guidance”)
- Adverse reactions due to overdose, abuse, misuse and medication error (for details please refer to the paragraph “Pharmacovigilance parameters definitions and reporting guidance”)

10.2. Pharmacovigilance parameters definitions and reporting guidance

10.2.1. Adverse Events

10.2.1.1. *Definitions*

An ***adverse event (AE)*** is any untoward medical occurrence in a subject or clinical investigation subject receiving/undergoing the study treatments (paclitaxel, ddAC, durvalumab, oleclumab, SBRT and surgery) and which does not necessarily have a causal relationship with these study treatments.

An AE can therefore be any unfavourable and unintended sign (including an abnormal laboratory finding, for example), symptom or disease temporally associated with the study treatments, whether or not considered related to them.

An ***adverse reaction (AR)*** is any untoward and unintended response to the study treatments (paclitaxel, ddAC, durvalumab, oleclumab, SBRT and surgery).

This definition covers also medication errors and uses outside what is foreseen in the protocol, including misuse and abuse of the study drugs.

This definition implies a reasonable possibility of a causal relationship between the event and the study treatments (paclitaxel, ddAC, durvalumab, oleclumab, SBRT and surgery).

An ***unexpected adverse reaction (UAR)*** is an adverse reaction, the nature or severity (intensity) of which is not consistent with the applicable product information (investigator's brochure for an unauthorised investigational product or summary of product characteristics for an authorised product, protocol for radiotherapy/surgery).

Remarks:

- Pre-existing conditions which worsen during a study are to be reported as AEs.
- Abnormal laboratory findings (e.g. hematology, hemostatic function, blood chemistry and serology) or other abnormal assessments (e.g. vital signs) that are judged by the principal investigator as clinically significant have to be recorded as AEs.

10.2.1.2. *AE intensity*

Intensity of all AEs will be graded according to the NCI-CTCAE version 5.0 on a five-point scale (Grade 1 to 5) and reported in detail on the CRF. Adverse events not listed in CTCAE should be graded as listed in table 16.

Table 16 Grading for Adverse Events not listed in NCI-CTCAE

CTC Grade	Equivalent To:	Definition
Grade 1	Mild	Asymptomatic or mild symptoms; clinical or diagnostic observations only; intervention not indicated
Grade 2	Moderate	Minimal, local or non-invasive intervention indicated; limiting age-appropriate instrumental activities of daily living (ADL)*
Grade 3	Severe	Severe or medically significant but not immediately life threatening; hospitalisation or prolongation of hospitalisation indicated; disabling; limiting self-care ADL**

Grade 4	Life-threatening/disabling	Life-threatening consequences; urgent intervention indicated
Grade 5	Death	Death related to AE

*Instrumental ADL refer to preparing meals, shopping for groceries or clothes, using the telephone, managing money etc.

**Self-care ADL refer to bathing, dressing and undressing, feeding self, using the toilet, taking medications, and not bedridden.

10.2.1.3. *Relationship to study treatments*

The causality relationship of the AE to the study treatments is assessed by the principal investigator as either: related, unrelated.

If there is a reasonable suspected causal relationship to the study treatments, i.e., there are facts (evidence) or arguments to suggest a causal relationship, event relationship should be assessed as **Related**.

The following criteria should be considered in order to assess the relationship as **Related (Reasonable possibility):**

- Reasonable temporal association with study treatment.
- It may or may not have been produced by the subject's clinical state, environmental or toxic factors, or other modes of therapy administered to the subject.
- Known response pattern to suspected study treatment.
- Disappears or decreases on cessation or reduction in dose.
- Reappears on re-challenge.

The following criteria should be considered in order to assess the relationship as **Unrelated (No reasonable possibility)**:

- It does not follow a reasonable temporal sequence from administration of study treatment.
- It may readily have been produced by the subject's clinical state, environmental or toxic factors, or other modes of therapy administered to the subject.
- It does not follow a known pattern of response to the suspected study treatment.
- It does not reappear or worsen when the study treatment is re-administered.

10.2.1.4. *Reporting of adverse events*

From the first administration of study treatment until 60 days after the surgery, all AEs should be reported on the AE page of the CRF. After this period, the following AE should be reported on the AE page of the CRF:

- immune-related AE
- endocrine-related AE
- cardiovascular-related AE
- radiation therapy-related AE

All AEs will be followed until one of the following occurs:

- Complete resolution

- Return to baseline condition
- Event is considered by the principal investigator to be unlikely to resolve
- Death
- Subject end of study

The final outcome of each AE must be recorded in the CRF.

The principal investigator has to follow-up any AEs that are not resolved after the subject end of study as long as medically indicated but without further recording in the CRF.

Note: Any abnormal laboratory test result and any abnormal assessment (e.g. vital signs) result reported as AE should be followed until it has returned to the normal range, baseline value (laboratory results obtained during the screening) and/or an adequate explanation of the abnormality is found.

10.2.2. Serious Adverse Events

10.2.2.1. *Definitions*

A ***serious adverse event (SAE)*** or ***reaction (SAR)*** is any untoward medical occurrence that results in any of the following outcomes:

- Death;
- Life-threatening;
- In-patient hospitalisation or prolongation of existing hospitalisation;
- Persistent or significant disability/incapacity;
- Congenital anomaly/birth defect;

Medical and scientific judgement should be exercised in deciding whether expedited reporting is appropriate in other situations, such as important medical events that may not be immediately life-threatening or result in death or hospitalisation but may jeopardise the subject or may require intervention to prevent one of the other outcomes listed in the definition above. These should also usually be considered serious (medically significant condition).

The term “serious” is not synonymous with the term “severe”. The term "severe" is often used to describe the intensity (severity) of a specific event (as in mild, moderate, or severe myocardial infarction); the event itself, however, may be of relatively minor medical significance (such as severe headache). While the term "serious” is based on subject or event outcome or action criteria usually associated with events that pose a threat to a subject's life or functioning. Seriousness (not severity) serves as a guide for defining regulatory reporting obligations.

A ***suspected unexpected serious adverse reaction (SUSAR)*** is a suspected adverse reaction that is both unexpected and serious and is subject to expedited reporting. The expectedness assessment of a serious adverse reaction is done by the sponsor by using the reference safety information within the investigator’s brochure (IB) or the summary of product characteristics (SmPC) for the study drugs and the protocol and the ICF for the surgery and radiotherapy.

In this study, the expectedness assessment will be performed by using the Reference Safety Information within:

- Investigator’s brochure for durvalumab.

- Investigator's brochure for oleclumab.
- Summary of product characteristics for paclitaxel.
- Summary of product characteristics for doxorubicin.
- Summary of product characteristics for cyclophosphamide.

Remarks:

- Death is an outcome and not an event therefore the CTCAE event(s) that caused the death should be reported as SAE(s) except in case of sudden death (an unexpected cessation of life that cannot be attributed to a CTCAE term associated with Grade 5) or death of unknown origin.
- The term "Life-threatening" refers to an event in which the subject was at risk of death at the time of the event; it does not refer to an event which hypothetically might have caused death if it were more severe.
- An in-patient hospitalisation is a hospital stay equal or greater than 24 hours.
- Related second primary malignancies (SPM) [i.e. a cancer judged by the investigator to be related to study treatments. It is not considered a metastasis of the initial malignancy] are to be reported as SAEs at any time regardless of the time elapsed since the last dose of adjuvant radiotherapy.

10.2.2.2. *Reporting of serious adverse events by the investigators*

From Informed Consent Form signature until initiation of the first study treatment, all SAEs related to a protocol-mandated intervention (e.g., SAEs related to invasive procedures such as biopsies) have to be reported on SAE form.

All SAEs from the first administration of study treatment and up to 60 days after the surgery must be collected and reported on SAE form and on AE page of the CRF.

After this period, only SAEs which have a reasonable possibility to be related to the study treatments (even if the study has been closed) must be reported.

All SAEs will be followed until one of the following occurs:

- Complete resolution
- Return to baseline condition
- Event is considered by the principal investigator to be unlikely to resolve
- Death
- End of study

Note: Any abnormal laboratory test result and any abnormal assessment (e.g. vital signs) result reported as SAE should be followed until it has returned to the normal range, baseline value (laboratory results obtained during the screening) and/or an adequate explanation of the abnormality is found.

Table 17: SAE reporting

From IC signature up to 1st study treatment administration	SAEs related to protocol-mandated intervention
From 1st study treatment administration up to 60 days after surgery	All SAEs

From day 61 after surgery	Only SAEs related to study treatments (paclitaxel, ddAC, durvalumab, oleclumab, SBRT and surgery)

All SAEs must be reported to the sponsor immediately and in any case no later than 24 hours from the time the principal investigator or study staff member became aware of the event. The SAE must be submitted to the sponsor via the study-specific SAE form completed in English.

The SAE report has to be sent to the fax number or email address mentioned on the study-specific SAE form.

Any additional documents in local language have to be provided with an English translation of the relevant information.

To be compliant with regulatory reporting requirements, all initial SAE forms must contain the following mandatory information: one identifiable subject, one identifiable reporter, one adverse event, one seriousness criteria, the relationship to study medication, outcome at last observation. Within 7 calendars days, the principal investigator must provide complete information requested on the SAE form of any reported serious adverse event. If the completed form is not received within this deadline, the sponsor will issue queries to the principal investigator.

Any queries on the SAEs sent out by the sponsor need to be answered within 7 days.

All SAE forms need to be reviewed, dated and signed by the principal investigator or medical physician as specified in the study task delegation log.

The principal investigator should report SAEs that occur at their institution to their respective ethics committee according to the local committee's policies.

10.2.2.3. *Reporting of serious adverse events by the sponsor*

The SAE reporting and the SUSAR reporting will be in accordance with EU Directive 2001/20/EC and the CT-3 guidance on the collection, verification and presentation of adverse event/reaction reports arising from clinical trials on medicinal products for human use (2011/C 172/01). The sponsor will be responsible of the reporting of the SUSARs to the EudraVigilance Clinical Trial Module (EVCTM), the Competent Authorities, the Ethics Committees and the participating investigators.

10.2.3. Exceptions to AEs/SAEs reporting

Some hospitalisation scenarios do not require reporting as an SAE such as:

- Elective hospitalisation for pre-existing conditions that have not been exacerbated by study medication.
- Hospitalisation planned before the subject consented for study participation and where admission did not take longer than anticipated.
- Hospitalisations for reasons described in the protocol (e.g. hospitalisation for study medication administration, hospitalisation for study related procedures). However, event requiring hospitalisation or prolongation of hospitalisation as a result of a complication of study treatment administration or study related procedures is reported as SAE.
- Hospitalisation or prolonged hospitalisation in absence of an AE (social, technical, practical reason and/or convenience admission to a hospital, palliative care, rehabilitation).

Progression of underlying malignancy should not be reported as an AE or SAE if it is clearly consistent with the progression of the underlying cancer but will be reported on the CRF.

Unrelated SPM (i.e. a cancer that is unrelated to the study treatment(s) and is not a metastasis from the initial malignancy) should not be reported as an AE or SAE but should however be reported on the CRF on the appropriate form (SPM form).

Hospitalisation due solely to the progression of underlying malignancy should NOT be reported as an SAE.

Clinical symptoms of confirmed progression of underlying malignancy should not be reported as AEs or SAEs unless the symptoms cannot be determined as exclusively due to the confirmed progression of the underlying malignancy or does not fit the expected pattern of confirmed progression for the disease under study.

Deaths related to progression of the underlying disease during the course of the study will not be reported as a SAE but should be reported on the Death CRF page section (unless the subject has withdrawn consent).

10.2.4. Adverse Events of Special Interest for durvalumab: immune-mediated adverse events

10.2.4.1. *Definition*

The adverse events of special interest (AESIs) for durvalumab are defined as AEs that include, but are not limited to, events with a potential inflammatory or immune-mediated mechanism that may require more frequent monitoring and/or interventions such as corticosteroids, immunosuppressants, and/or endocrine therapy.

Early recognition of signs and symptoms potentially related to an inflammatory or immune-mediated mechanism is important for proper management of toxicities. For guidance on identifying, evaluating, and treating AESI/imAEs, see the Appendix 1 to the protocol.

AESI/imAEs observed include:

- Diarrhoea/colitis.
- Intestinal perforation.
- Pneumonitis events (e.g. pneumonitis, interstitial lung disease (ILD), acute interstitial pneumonitis and pulmonary fibrosis).
- Hepatitis events (e.g. autoimmune hepatitis, hepatitis toxic, hepatocellular injury, hepatotoxicity and hepatitis). Other AESIs such as hepatic failure, jaundice, hyperbilirubinaemia and laboratory abnormality AESIs have been observed.
- Endocrinopathies (i.e. events of hypophysitis, hypopituitarism adrenal insufficiency, diabetes insipidus, hyper- and hypothyroidism, type I diabetes mellitus and diabetes insipidus).
- Rash/dermatitis (including maculo-papular rash, erythematous rash, pruritus, alopecia aerata and vitiligo, eczema and erythema, Steven-Johnsons syndrome, toxic epidermal necrolysis, dermatitis bullous, dermatitis exfoliative or dermatitis psoriasiform, pemphigoid).
- Nephritis events (blood creatinine increases, nephrotic syndrome, rapid progressive glomerulonephritis and acute renal failure).
- Pancreatitis (or laboratory findings suggestive of pancreatitis - increased serum lipase, increased serum amylase)
- Myocarditis

- Myositis/polymyositis
- Events with an inflammatory or immune mediated mechanism could occur in nearly all organs.

Other inflammatory responses that are rare/less frequent with a potential immune-mediated aetiology are also considered as AESIs and include, but are not limited to, pericarditis, sarcoidosis, uveitis and other events involving the eye (e.g. keratitis and optic neuritis), skin (e.g. scleroderma and vitiligo), haematological (e.g. haemolytic anaemia, immune thrombocytopenic purpura and immune-mediated neutropenia), rheumatological events (polymyalgia rheumatic and autoimmune arthtitis), vasculitis, non-infectious meningitis and non-infectious encephalitis, cholangitis sclerosing, immune-mediated cystitis. Neuropathy/neuromuscular toxicities such as Guillain-Barré syndrome, and myasthenia gravis are also considered as AESIs.

In addition, infusion-related reactions and hypersensitivity/anaphylactic reactions with a different underlying pharmacological aetiology are also considered AESIs.

More details can be found in the IB.

10.2.4.2. *Reporting of AESIs for durvalumab*

Any AESIs for durvalumab ≥ grade 2 (whatever their relationship to durvalumab) occurring during the study treatment period and up to 90 days after the last administration of study treatment (oleclumab/durvalumab) must be reported to the sponsor on SAE form.

After this period, only AESIs ≥ grade 2 which have a reasonable possibility to be related to study drug (even if the study has been closed) must be reported on the SAE form.

All AESIs ≥ grade 2 for durvalumab must be reported within 1 week by the principal investigator or an authorised staff member (i.e. as specified in the study task delegation log) to the sponsor on the study-specific SAE form in English. The report has to be sent to the fax number or email address mentioned on the Serious Adverse Event form and on CRF.

All AESIs for durvalumab (< grade 2 and ≥ grade 2) (whatever their relationship to durvalumab) must be reported in the CRF.

Important notes:

1. If the above-mentioned AEs ≥ grade 2 are assessed as definitely related to chemotherapy by the investigator, they should not be not be reported on the study specific SAE form.

2. If the above AESIs are also observed for oleclumab, they should be reported to the sponsor as specified above.

10.2.5. Adverse events of special interest for oleclumab: cardiovascular adverse events

10.2.5.1. *Definition*

The adverse events: cardiac chest pain, transient ischemic attack and thromboembolic events are of special interest due to oleclumab potential risk of arterial calcifications, arterial ischemic disorder, and thrombosis.

Edema (e.g, pulmonary or peripheral) is regarded as AESI due to oleclumab potential risks of increased microvascular permeability.

10.2.5.2. *Reporting of AESIs for oleclumab*

Any AESIs for oleclumab (irrespective of grade and relationship to oleclumab) occurring during the study treatment period and up to 90 days after the last administration of study treatment must be reported to the sponsor.

After this period, only AESIs which have a reasonable possibility to be related to study drugs (even if the study has been closed) must be reported on the SAE form.

All AESIs for oleclumab must be reported within 1 week by the principal investigator or an authorised staff member (i.e. as specified in the study task delegation log) to the sponsor on the study-specific SAE form in English. The report has to be sent to the fax number or email address mentioned on the Serious Adverse Event form and on CRF.

10.2.6. Potential Hy's law case

10.2.6.1. *Definition*

Hepatic function abnormality that fulfils the biochemical criteria of a potential Hy's Law case in a study subject, with or without associated clinical manifestations, is required to be reported as AESI, unless a definitive underlying diagnosis for the abnormality (e.g., cholelithiasis or bile duct obstruction) that is unrelated to investigational product has been confirmed.

The criteria for a potential Hy's Law case is AST or ALT ≥3x ULN together with TBL ≥2xULN at any point during the study following the start of study medication irrespective of an increase in ALP.

- If the definitive underlying diagnosis for the abnormality has been established and is unrelated to investigational product, the decision to continue dosing of the study subject will be based on the clinical judgment of the investigator.
- If no definitive underlying diagnosis for the abnormality is established, dosing of the study subject must be interrupted immediately. Follow-up investigations and inquiries must be initiated by the investigational site without delay.

10.2.6.2. *Reporting*

Any potential Hy's law case occurring during the study treatment period and up to 90 days after the last administration of study treatment (oleclumab/durvalumab) must be reported to the sponsor.

All potential Hy's law case must be reported immediately and no later than 24 hours from the time the principal investigator or an authorised staff member (i.e. as specified in the study task delegation log) became aware of the event to the sponsor on the study-specific SAE form in English. The report has to be sent to the fax number or email address mentioned on the Serious Adverse Event form and on CRF.

10.2.7. Pregnancy

A female subject who becomes pregnant during the study must be instructed to stop taking study treatment and to immediately inform the principal investigator.

The principal investigator or an authorised staff member (i.e. as specified in the study task delegation log) should report any pregnancy diagnosed in a female subject during the study treatment period and within 12 months after last study drugs administration. This reporting is done immediately and no later than 24 hours from the time of awareness to the sponsor by

using the pregnancy notification form. The pregnancy report has to be sent to the fax number or email address mentioned on the Pregnancy form.

The principal investigator should counsel the female subject and discuss the risks of continuing with the pregnancy and the possible effects on the foetus. The principal investigator should follow-up with the female subject until conclusion of the pregnancy.

If the involved parties sign the specific Informed Consent Form for data collection in case of pregnancy, the principal investigator should report the outcome of any pregnancy and report any abnormality in the newborn within 1 month after the delivery as a follow-up to the initial pregnancy notification to the sponsor. While the pregnancy itself is not considered to be an AE or SAE, any pregnancy complication, any spontaneous abortion, any abnormality in newborn and any child death (intrauterine death, stillbirth, post-delivery death) will be recorded as an AE or SAE (if any of the criteria for seriousness is met).

10.2.8. Overdose, misuse and abuse

10.2.8.1. *Definitions*

Overdose: this refers to the administration of a quantity of a medicinal product given per administration or cumulatively which is above the maximum recommended dose according to the protocol. Clinical judgement should always be applied.

Misuse: this refers to situations where the medicinal product is intentionally and inappropriately used not in accordance with the protocol.

Abuse: This corresponds to the persistent or sporadic, intentional excessive use of a medicinal product, which is accompanied by harmful physical or psychological effects.

10.2.8.2. *Reporting of overdose, misuse and abuse*

The principal investigator or an authorised staff member (i.e. as specified in the study task delegation log) should report any adverse reaction due to overdose, misuse and abuse, occurring in a subject during the study drugs administration period immediately and no later than 24 hours from the time of awareness to the sponsor by using the appropriate notification form. The report has to be sent to the fax number or email address mentioned on the notification form.

10.2.9. Medication Error

10.2.9.1. *Definition*

Medication error: this refers to any unintentional error in the prescribing, dispensing, or administration of a medicinal product while in the control of the healthcare professional or subject.

10.2.9.2. *Reporting of medication error*

The principal investigator or an authorised staff member (i.e. as specified in the study task delegation log) should report any adverse reaction due to medication error, occurring in a subject during the study drugs administration period immediately and no later than 24 hours from the time of awareness to the sponsor by using the appropriate notification form. The report has to be sent to the fax number or email address mentioned on the notification form.

11. TRANSLATIONAL RESEARCH(ES)

11.1. Overview of tissue and blood sample collection

The translational research proposal within this clinical trial aims to understand the impact of the different immunotherapeutic approaches including the combination pre-operative radiation therapy with chemotherapy and durvalumab with oleclumab on the activation of the immune response in the less immunogenic luminal B breast cancer subtype. The project will also characterize the host/tumour interaction before and after treatment to identify potential predictive biomarkers of response to treatment as well as mechanisms underlying resistance to the therapeutic strategy.

For this purpose, several tissue samples and blood samples collection will be performed during the study.

11.1.1. Tissue sample collection

11.1.1.1. *Overview*

Definition	Timepoint	Tissues	Notes
**Diagnostic biopsy**	Performed at the diagnosis of the tumour, before the subject was informed of the trial	FFPE	
**Screening biopsy**	Performed during screening for the trial after signing of the ICF	1 FFPE & 2 frozen cores of the primary tumour + invaded lymph node if feasible<br>+ fresh material*	If Mammaprint and PD-L1 are performed on the screening biopsy ➔ 2 FFPE
**On-treatment**	Performed at week 6 (5-12 days after SBRT).	1 FFPE & 2 frozen cores of the primary tumour + invaded lymph node if feasible<br>+ fresh material*	If feasible same area as screening biopsy
**Surgery**	Performed on the surgical specimen	1 FFPE & 2 frozen blocks of the residual tumour + invaded lymph nodes if feasible<br>+ fresh material*	Only if residual macroscopic disease
At progression	Performed at first local or distant progression prior to the next anti-cancer therapy	1 FFPE & 2 frozen<br>+ fresh material*	Optional biopsy

* Fresh tissue material : in selected sites, additional fresh material will be collected when feasible.

11.1.1.2. *MammaPrint testing and PD-L1 testing*

Mammaprint testing is used as an inclusion criteria and will be assessed on the diagnostic or screening FFPE tumour tissue biopsy by Agendia prior to enrolment/ randomisation. For this purpose, the study sites should provide 10 FFPE slides (5µm thick).

PD-L1 expression will be assessed using immunohistochemistry (IHC) on the screening FFPE tumour tissue lesion biopsy by a central laboratory (Institut Jules Bordet, Brussels, Belgium) prior to randomization for stratification purposes (for phase II only). For this purpose, the study sites should provide between 4 to 6 unstained and freshly cut FFPE slides from the diagnostic or screening biopsy. All sites and study team staffs will be blinded to PD-L1 status.

> Note: if the requested material is most readily available from the screening biopsy, a second FFPE must be secured from the screening biopsy for MammaPrint and PD-L1 purpose. In this case, the screening biopsy will consist of 2 FFPE instead of 1 FFPE.

11.1.1.3. *Bilateral, multifocal or multicentric disease*

For bilateral, multifocal or multicentric disease: a single index lesion should be selected using the algorithm provided hereunder (Appendix 2). The "*pre-treatment biopsy"* should be taken from the index lesion. A pre-treatment biopsy is not necessary from a non-index lesion. The same index lesion should be used for the "*on-treatment*" biopsy. If necessary, marking and identification of the index lesion can be performed to avoid geographical misses. Marking should follow local practice methods.

> Please note: all biopsiable foci should be proven ER+/HER2- with a *diagnostic biopsy* (see inclusion criterium #9 for more details), however a *pre-treatment biopsy* should only be taken from the largest measurable disease.

MammaPrint and PD-L1 testing will be performed on the index lesion.

SBRT will be delivered to all lesions, index and non-index. Hence, in case of bilateral breast cancer, MRI-compatible markers (fiducials) should be placed in both breasts to allow bilateral SBRT.

11.1.1.4. *Unacceptable tissue samples*

Fine-needle aspiration (defined as samples that do not preserve tissue architecture and yield cell suspension and/or smears), brushing, and cell pellets from cytology samples are not acceptable.

11.1.2. Blood samples collection

Several blood samples collection (whole blood, PBMCs, plasma and serum) will be performed, for the purpose of the translational research part, at several time points:

- Baseline blood collection at week 1 before treatment start (meaning within 3 days before study drug administration);
- "On treatment" blood samples collection at week 4, at week 6, at week 12, at the end of chemotherapy (at week 19 in case no chemotherapy interruptions occurred) and at surgery. PBMCs will not be collected or evaluated at week 12 and week 19.
- During the follow-up phase at 1 year, 2 years, 3 years and 5 years after surgery.
- End of treatment visit blood sample collection (30 days (± 7 days) after surgery).
- "At first disease progression", blood samples collection within 40 days after PD has been reported and prior to the next anti-cancer therapy;

- At first occurrence of possible immune related toxicity (development of an irAE determined by the investigator to be unacceptable given the individual subject's potential response to therapy and severity of the event).

The blood samples must be collected as follow:

- Whole blood for isolation of normal DNA to exclude germline variants during our next-generation sequencing analyses (1x5 mL, at baseline only);
- Blood for plasma (3x9 mL per time point);
- Blood for PBMCs isolation (1x6mL per time point)
- Blood for serum isolation (1x9 mL per time point);

11.2. Objectives

The main translational objectives of the translational research project are:

- To identify potential predictive immune biomarkers by performing a widespread histological and molecular profiling of the pre- during and post- study treatment peripheral blood and tumour tissues using IHC and other histological methods as immunofluorescence as well as flow cytometry, chromatography and next generation sequencing
- To evaluate the predictive value of TILs levels in arms 2 and 3 versus arm 1.
- To evaluate the impact of the immunotherapeutic approaches on the activation of the immune response

The following translational objectives will be performed on a subset of subjects as a pilot study and might be expanded to more subjects in case of interesting results:

- Evaluation of changes in tumour immune infiltrates and immune-related gene expression in tumour tissue prior to during and after study treatment;
- Monitoring of plasma ctDNA according to response/resistance to the study treatment. Investigating the correlation between plasma ctDNA during the follow-up phase and cancer relapse.
- T-cell receptor repertoire assessment of tumour-infiltrating T-cells;
- Evaluation of blood-based gene signatures and changes in prevalence of various peripheral immune cell subpopulations (e.g., effector/memory T cells, natural killer [NK] cells, regulatory T cells [Tregs], and myeloid cells) prior to, during and post study treatment.
- Evaluation of ovarian function through measurement of anti-Mullerian hormone (AMH), follicle stimulating hormone (FSH), estradiol (E2) and progesterone.

The techniques used for the above-mentioned objectives possibly include but are not limited to:

- Molecular profiling using whole-exome and RNA sequencing, spatial transcriptomics and single-cell RNA sequening;
- Immunological profiling using flow cytometry (peripheral blood);
- Histological profiling using multiplex IHC and multispectral imaging

Other biomarker research will be defined by the study scientific committee at the end of the trial to ensure the optimal use of update technologies and hypotheses.

Due to the fast evolution within these fields, the actual analytical methodology will only be specified and decided at the time these analyses will start.

11.3. Timelines

	Screening	Week 1[a]	Week 4	Week 6	Week 12	End of Chemo (Week 19, if no	Surgery	End of Treatment	1 year after surgery	2 years after surgery	3 years after surgery	5 years after surgery	First Immune related	First PD
Tumour tissue<br>(1 FFPE + 2 frozen tissues)<br>In selected sites only: 1 fresh tissue also asked	X			X			X							X<br>(optional)
Biopsy of a at diagnosis positive lymph node (optional)	X			X			X							
Whole Blood (1X5 mL)		X												
Blood for plasma (3X9 mL)		X	X	X	X	X	X	X	X	X	X	X	X	X
Blood for serum (1X9 mL)		X	X	X	X	X	X	X	X	X	X	X	X	X
Blood for PBMCs (1x6 mL)		X	X	X			X	X					X	X

a) before treatment start (meaning within 3 days prior study drug administration)

11.4. Biological material handling

Standardised protocols are applied consistently in preparing and storing biological samples to ensure quality and to avoid introducing confounders into research studies. See also the National Cancer Institute (NCI) best practice for biospecimens resource (http://biospecimens.cancer.gov/bestpractices/), the Recommendations for Collection and Handling of Specimens from Breast International Group (BIG) (reference) and the BIG movie on improving translational research samples collection in international trials (available on http://www.youtube.com/user/BreastIntGroup).

Kits are provided to all participating sites in order to perform biological samples collection. The kits are provided by a central service provider located in Belgium.

Further details on collection, handling, local storage and shipping are provided in a separate laboratory instruction manual.

11.5. Central biobanking of biological material collected during the trial and Destruction

Biological samples, material left over and/or derivatives (after molecular analyses embedded in the current trial have been performed) are stored securely, for a minimum of 20 years or until exhaustion or for the maximum time allowed by local regulations or until a request is made for the biological samples to be returned or destroyed, under the responsibility of the Institut Jules Bordet located in Brussels, Belgium. Some of this biological material may be shipped for further analyses if necessary to other academic institutions or for-profit organisations.

To protect the subject's identity and privacy, the biological samples are double-coded. First they are labelled with a unique study number or 'code' and this code does not carry any personal identifiers. Then the biological samples are relabelled with a second code, which is linked to the first code via a second coding key. The use of the second code provides additional confidentiality and privacy protection over the use of a single code. Access to both coding keys is needed to link any data or biological samples back to a subject identifier. In general, the principal investigator is responsible for maintaining the first coding key and does not have access to the second coding key. As the biological samples and associated data can be traced back indirectly via the use of both coding keys, it may be possible to undertake actions such as sample withdrawal. The principal investigator, his/her staff or those working with them (for example, other researchers) only use the biological samples for the purpose stated in the current trial in accordance with the terms outlined in the Informed Consent Form. The principal investigator and his/her staff require anyone who works with the biological samples to agree to hold the research information and any individual results in confidence.

Subjects may request that their biological samples as well as their material left over and/or derivatives be removed from storage and destroyed or returned to their institution in accordance with the terms outlined in the Informed Consent Form. Requests for destruction or return of the biological samples should be made in writing to the principal investigator. In turn, the principal investigator must promptly inform the sponsor so that appropriate follow-up can be initiated. The principal investigator will be informed when the biological material is destroyed or returned and should notify the subject that biological material destruction is complete or that biological material is stored back in the institution.

Please note any molecular data that is already generated at time of biological material destruction or return request will be used for translational research objectives listed in the protocol. Please also note that specific molecular and clinical data (generated within the study or future research (see below)) could be put on secured database accessible via internet. This can be done for the purpose of data sharing or for publication needs. Data will be double coded and no reference to subject name will be made. Accession to this data will be made available

to any individuals making a specific request and following data access policy rules (specific per database) previously agreed by the sponsor.

Further details on the legal entity acting as data controller, the legal entity acting as data processor and the medical doctor responsible for the sponsor biobank will be provided in a separate laboratory instruction manual.

11.6. Future undefined research

Data and biological samples as well as material left over and/or derivatives (after molecular analyses embedded in the current trial have been performed), may be used for undefined future research. Proposals for access to such samples and data will have to be approved by the study team as well as the appropriate Ethics Committee(s). Access to any biological samples as well as material left over and/or derivatives thereof for future research is only possible if subject consented to the use of the biological material for future undefined research. Electronic documents to keep track of tumour and blood samples will be kept in participating sites and at study sponsor. These documents will contain the subject ID code with no reference to subject name, initials and address. Some of this biological material may be shipped for further analyses if necessary to other academic institutions or for-profit organizations.

12. STATISTICAL CONSIDERATIONS

12.1. Statistical design

12.1.1. Sample size

Safety run-in: 6 subjects (see earlier sections of this protocol).

After the safety run-in, if accrual continues, subjects will be randomised in a 1:1:1 ratio between 3 arms and the primary endpoint that will be measured on those subjects is the achievement of RCB 0-1 versus RCB 2-3. The trial is designed to compare arm 1 and arm 2 as well as arm 1 and arm 3. The trial is not powered to compare arms 2 and 3 and no specific hypothesis is formulated for that comparison.

It is expected in arm 1 to get a 15% RCB 0-1 rate and the experimental treatment arms will be considered of interest for further investigation if this rate can be increased to 45%.

To estimate the expectations in arms 2 and 3, the results of the phase 2 trial I-SPY 2 were taken into account: the addition of immunotherapy to standard neo-adjuvant chemotherapy increased pCR from 13.6% to 34.2%. Taking into account the addition of radiation therapy and the use of RCB 0-1 as endpoint, we hypothesise an increase in RCB 0-1 from 15% to 45% in either arm 2 or 3.

There will be then two hypotheses testing, both aiming to reject the null hypothesis of equality of the RCB 0-1 rates in the control arm and in the experimental arm. A two-sided alpha level of 2.5% will be used and power of 80% will be targeted. The alpha level of 2.5% was chosen to adjust for multiplicity. No continuity correction was used and a pooled estimate of variance was chosen to estimate requested sample size.

An evaluable subject is defined as follows (all criteria must be met):

1) Subject received at least the first dose assigned treatment (dose reduction is permitted).
2) Subject underwent surgery and a RCB score was obtained.
3) Subject must be MammaPrint high risk or MammaPrint unevaluable.

With those assumptions, 44 evaluable subjects per arm are needed, i.e. a total of 132 subjects. In order to take into account a 5% inevaluability rate, this sample size will be increased to 147

subjects to be randomised. Assuming 20% screening failures, the number of screened subjects will be increased to 184 subjects.

Table 18

**Safety run-in**	
Numbers of subjects needed: Enrolled	6 subjects
**Phase II trial**	
Primary endpoint achievement RCB 0/1 vs RCB 2/3	Standard arm: 15%<br>Experimental arms: 45%<br>Hypothesis: increase in RCB 0/1 from 15% to 45% in either arm 2 or 3.
Number of subjects needed:<br>• **1:1:1 randomisation**	44 subjects per arm, total of **132** subject
Increase for:<br>• 5% ineligible after randomisation<br>• 5% unevaluable subjects	Add 15 subjects<br>147 randomised subjects
Increase for 20% ineligible after screening.	Add 37 subjects.
**Total for phase 2 trial**	**184 subjects screened**
**Total for whole trial**	**190 subjects for safety run-in and phase II**

12.1.2. Study duration

12.1.2.1. *Expected Accrual Time*

SAFETY RUN-IN: It is estimated that 3 subjects per month will be enrolled leading to an accrual time between 2 months.

PHASE II: It is estimated that 6 subjects per month will enter the treatment phase leading to an accrual time of 24 months.

In total, the accrual time will be between 24 and 26 months.

12.1.2.2. *Expected Duration of the Trial*

Approximate treatment duration for a subject: 6 months

Expected duration of the all study: approximately 96 months (taken into account safety run-in and phase II accrual, treatment period and follow-up), assuming that the phase II starts approximately 1 month after the safety interim analysis of the safety run-in.

As defined in the End of Study definition (*section 15. End of study*), the study will end approximately 66 months after the date the last subject has been randomised.

12.2. Interim analyses

12.2.1. Safety run-in

As planned and detailed in section 3.2.1, a safety interim analysis was performed at the end of the safety run-in and presented to an IDMC before starting the phase II randomized trial. Details on the results of the safety run-in and IDMC recommendations can be found in section 3.2.1.6.

12.2.2. Second safety analysis

A second safety analysis will be performed after ***inclusion*** of 40 subjects in the phase II randomized trial. The safety analysis will consist in a description of all reported adverse events. The results will be presented to the IDMC.

Note: the second safety analysis was completed and its results discussed with an IDMC on 16/09/2022. The IDMC did not identify a safety signal that does not allow to continue the trial.

12.2.3. Analysis for futility of arm 2 and 3 and third safety analysis.

Interim analysis for futility of arm 2 and arm 3

This analysis will be performed at 50% information rate of the phase II randomized trial, corresponding to the timepoint in which 22 subjects underwent surgery in each arm and will compare the primary endpoint (arm 1 versus arm 2 and arm 1 versus arm 3). The analysis will consist of two parts: a futility analysis both for arms 2 and 3 and a third safety analysis All these analyses will be presented to the IDMC.

Comparison for futility for both comparisons (arm 1 versus arm 2 and arm 1 versus arm 3) will be carried out at an information fraction of **1/2**.

We keep the goal to be able to detect an increase from 15% to 45% with a power of 80% and a 2-sided alpha level of 2.5% (due to multiplicity with the 2 planned comparisons).

The futility boundaries would be calculated using Lan-Demets bêta spending functions and non-binding boundaries once 1/2 of evaluable subjects will have been evaluated (availability of RCB).

Interim analysis would be carried out after 22 subjects evaluated in each arm (22 subjects that underwent surgery in each arm).

A subject that was included in the trial with a violation of an inclusion or exclusion criterium **and** discontinued the trial within 6 weeks after inclusion will be considered unevaluable for the interim analysis and this subject will be replaced.

Stopping boundaries for early closure for both of the experimental arms (arm 2 and/or arm 3) would be +/- 0.533 on the z scale.

Study enrollment will continue until the results of the futility analysis have been discussed by the IDMC.

At the time of interim analysis, descriptive analyses without formal comparisons would be done for dose intensity and for toxicity as well as the analysis of RCB 0-1 rate and calculation of test statistics for the comparison of the RCB rates.

The whole analyses would be submitted to an IDMC for advice about the continuation of the study with 3 arms or 2 arms or terminate the trial entirely.

Note: the interim analysis for efficacy was completed and its results discussed with an IDMC on 16/09/2023. According to the prespecified criteria for futility, the IDMC recommended that all arms can continue in the trial. The trial will hence continue until full accrual.

Third safety analysis

The safety analysis will consist of a description of all reported adverse events and will be presented to an IDMC.

Note: the third safety analysis was completed and its results discussed with an IDMC on 16/09/2023. The IDMC did not identify a safety signal that does not allow to continue the trial.

12.3. Final analysis

12.3.1. Populations

The final analysis will be carried out in two populations:

1) Intention-to-treat population
2) Per-protocol population.

The populations are defined according to the following table:

	**Intention-to-treat population**	**Per-protocol population**
MammaPrint unevaluable	Include	Exclude
Subject that was included in the trial with a violation of an inclusion or exclusion criterium **and** discontinued the trial within 6 weeks after inclusion.	Include	Exclude

A CONSORT diagram will be provided to show the enrolment of subjects, their allocation to treatment, their disposition status and how they are analysed in the trial.

12.3.2. Analyses

Primary analysis: This analysis will be carried out on the eligible and randomised subjects who have been operated and in whom the residual cancer burden was measured. The observed proportions will be compared by a chi square test without continuity correction at an alpha two-sided level of 2.5%. Confidence intervals for the difference between proportions will be provided at the usual 95% level. The primary analyses will compare arm 1 and arm 2 as well as arm 1 and arm 3.

As exploratory analysis, a confidence interval for the difference between proportions in arm 2 and arm 3 will be provided.

Secondary comparisons:

At surgery:

- Rates of subjects with pathological complete response:
  - ypT0/Tis ypN0 at the time of definitive surgery
  - ypT0ypN0 at the time of definitive surgery

- ypT0/Tis at the time of definitive surgery (disregarding the nodal response)
- ypN0 at the time of definitive surgery (disregarding the response of the primary cancer)

The observed proportions will be compared by a chi square test without continuity correction at an alpha two-sided level of 2.5%. Confidence intervals for the difference between proportions will be provided at the usual 95% level. The primary analyses will compare arm 1 and arm 2 as well as arm 1 and arm 3.

For other secondary and exploratory endpoints, two-sided alpha level are set at 5%.- Rates of subjects who have a two fold increase in TIL levels in subjects with a baseline biopsy and the week 6 biopsy and with TILs available using chi-square tests.

- Rates of subjects with breast conservation surgery using chi square tests.

Follow-up phase:

- Efficacy endpoints at 3 years and 5 years after surgery will be estimated as defined by the Standardized Definitions for Efficacy End Points in Neoadjuvant Breast Cancer Clinical Trials (NeoSTEEP) (88). The following endpoints will be assessed: event-free survival (**EFS**), breast cancer event-free survival (**BC-EFS**), overall survival (**OS**) and distant recurrence-free survival (**DRFS**). In accordance to NeoSTEEP, the point origin for these endpoints is the date of randomization. Estimation will be performed using the Kaplan-Meier method at 3 years of follow-up and 5 years of follow-up. Comparison will be performed using log rank tests between arm 1 and arm 2 as well as arm 1 and arm 3. A comparison between arm 1 vs a pool of arm 2+3 will also be performed to assess the effect of durvalumab to survival. This analysis will be conducted on the intention to treat population.

- **Ipsilateral locoregional recurrence** (breast, chestwall or locoregional nodal recurrence), **ipsilateral local recurrence** (breast or chest wall), and **ipsilateral locoregional nodal recurrence; each of these events** will be assessed using cumulative incidence risks. Log-rank analyses will be used to yield first-event rate ratios, confidence intervals and perform comparisons between arms.

- Safety, toxicity and cosmesis will be descriptively analysed and compared between arms in all subjects eligible, randomised and having started treatment; at baseline (week 1 of the trial) and during follow-up.

***Exploratory analyses**:*

- Exploratory analyses will be done to try to identify predictive factors of achievement of RCB0-1 and pCR at surgery. Besides the treatment arm, the covariates that will be tested will include PDL1 status, ER status (low versus high), TILs, TNM status, MammaPrint score, BluePrint result and baseline transcriptomic data. In addition, covariates generated from the week 6 biopsy will be evaluated, such as the impact of tumor cellularity at week 6 and the occurrence of RCB 0-1 and pCR. Modelling will be done using logistic regression models.

- Exploratory analyses will be done to try to identify predictive factors correlated to EFS. Besides the treatment arm, the covariates that will be tested will include PDL1 status, TILs, TNM status, MammaPrint score, BluePrint result and baseline transcriptomic data and ERlow status at baseline. In addition, covariates generated from the week 6 biopsy will be evaluated, such as the impact of complete tumoral response at week 6 .Modelling will be done using logistic regression models.

- The ovarian function will be evaluated in pre-menopausal subjects in blood samples at baseline and during follow-up at 1 year, 2 years, 3 years and 5 years after surgery and will be descriptively compared.

- Data about pregnancies and breastfeeding will be descriptively analysed.

- Plasma ctDNA samples before surgery and in the follow-up phase will be evaluated and compared to 1) the response and surgery and 2) the relapses in order to assess its predictive and prognostic value.

- Multiparametric MRI images at baseline and at week 12 will be descriptively correlated with tumor response at surgery and TILs changes. Exploratory analyses with radiomics will be performed to investigate correlations.

12.3.3. Timeline of analysis of the different endpoints

- **1st milestone**: when all randomised subjects have all the results of the evaluations after surgery (surgery W21-25 evaluations described in the schedule of assessments during study treatment period), except subjects who have terminated the study before surgery. In this milestone, the primary endpoints and other secondary endpoints regarding the clinical results after surgery should be analysed and reported.
- **2nd milestone**: when all randomised subjects have been followed up for at least 3 years and have all the results of year 3 scheduled evaluations (M36 evaluations described in the schedule of assessments during the follow-up period), except subjects who have who have terminated the study before year 3 of follow-up period. In this milestone, the endpoints regarding efficacy 3 years after surgery should be analysed.
- **3rd milestone**: when all randomised subjects have been followed up for at least 5 years and have all the results of year 5 scheduled evaluations (M60 evaluations described in the schedule of assessments during the follow-up period), except subjects who have who have terminated the study before year 5 of follow-up period. In this milestone the endpoints regarding efficacy 5 years after surgery should be analysed.
- **4th milestone**: the final clinical study report after the end of the study.

Important notes:

- Other unscheduled analyses for publications, including translational endpoints, can be done after the first milestone using the most updated data.
- The main analyses in each milestone or presented in each publication should use cleaned and validated data.
- For each milestone, a safety analysis should also be done using the most updated data.

13. FIRST ACT OF RECRUITMENT

The first act of recruitment is the first informed consent form signed off for the study. No advertising is used for this study.

14. SUSPENSION OR TRIAL TERMINATION

The sponsor must suspend or terminate the trial prematurely in certain circumstances, including:

- if the positive opinion by the Ethics Committee in charge of the trial is not granted or irrevocably revoked;

- if the authorisation by the Competent Authority of the Member State concerned in charge of the trial is not granted or irrevocably revoked;
- if subject recruitment is insufficient;
- the safety or health of participants is at risk, particularly as a result of inadequate product safety or manufacturing defects;
- alterations in accepted clinical practice that make the continuation of the trial unwise or harmful to participants;
- the Sponsor and/or the Institution and/or the Principal Investigator become or are declared insolvent or a petition in bankruptcy has been filed against it or if one of them is dissolved;

All the investigators will be immediately informed of the suspension or termination of the trial using expedited means (e.g. e-mail, investigator letters, Ethics Committee information).

The subjects should be contacted by the investigators to tell them of the suspension or the termination of the trial and to inform them of the actions they need to take. A follow-up appointment should be made for subjects so that their condition can be reviewed and any concerns addressed.

15. END OF STUDY

The end of study is declared when all the following criteria have been met:

- After last follow up visit of the last subject
- The trial is mature for the analysis of every endpoint as defined in the protocol, if the trial reaches its objectives.
- The database has been fully cleaned and frozen for all analyses

16. DIRECT ACCESS TO SOURCE DATA/DOCUMENTS

The participating site and the principal investigator shall allow the monitors, the employees of the sponsor (provided that subject confidentiality is protected) or persons subcontracted by the sponsor responsible for auditing, the representatives of the Ethics Committees, and of the Competent Authorities/ Regulatory agencies to have direct access to source data / documents/ complete study records.

17. QUALITY CONTROL AND QUALITY ASSURANCE

17.1. Quality control

Throughout the study the dedicated sponsor study team members verify the data to ensure that:

- the rights and well-being of subjects are protected
- the reported trial data are accurate, complete and verifiable from source documents
- the conduct of the trial is compliant with the IHC-GCP, the applicable regulatory requirements, the study protocol and the study guidelines.

Quality control activities combine central monitoring and clinical site monitoring. Monitoring activities may be performed remotely and/or on site according to the study needs.

17.2. Quality assurance

To ensure compliance with the protocol, CRF completion guidelines, study documentation, SOPs, GCP and all applicable regulatory requirements, the sponsor may conduct a quality assurance audit on participating site. Regulatory agencies may also conduct a regulatory inspection of the study. The principal investigator must inform the sponsor in case an inspection from Regulatory agencies has been scheduled at his/her site.

Such audits/inspections can occur at any time during or after completion of the study. If an audit or inspection occurs, the investigator and institution must agree to allow the auditor/inspector direct access to all relevant documents and to allocate his/her time and the time of his/her staff to the auditor/inspector to discuss findings and any relevant issues in the presence of the sponsor, as needed or required.

18. REGULATORY AND ETHICAL CONSIDERATIONS

18.1. Obligations

18.1.1. Trial Master File

The sponsor shall set-up and maintain an electronic Trial Master File (e-TMF) containing documents such as the essential documents and written communications relating to that Study which allow verification of the conduct of a Study and the quality of the data generated. The e-TMF must be readily available, and directly accessible upon request, to the Member States Concerned. The e-TMF must be kept in a secure location for the duration of the Study and archived after completion or premature termination of the Study in a secure fireproof facility for at least 25 years.

The sponsor shall provide all the participating sites with a copy of the final protocol, subject information sheets, consent forms and all other relevant study documentation; and any approved modification thereof when applicable.

18.1.2. Regulations, laws and guidelines

The sponsor (or its legal representative) and the principal investigators shall ensure compliance with the Regulation EU No 536/2014, the Guideline for Good Clinical Practice" ICH-E6 Tripartite Guideline, the principles of the "Declaration of Helsinki" 1964, as revised from time to time, and the EU General Data Protection Regulation (GDPR).

18.1.3. Authorisation

The sponsor (or its legal representative) shall submit an application dossier to the intended Member States Concerned in accordance with the Regulation EU No 536/2014, taking into account any local regulations/requirements. Each Member State concerned will consider the Reporting Member State's conclusion on Part I, and the ethics committee's/institutional review board's opinion on Part II, and issue their single national decision via the Clinical Trials Information System (CTIS). A Study cannot be commenced in a Member States Concerned until the single national decision has been issued. In case of a staggered application, starting with only a part I application, the part II application may be submitted after the conclusion part I, but not later than two years after the part I conclusion.

18.1.4. Modifications

The sponsor shall be entitled to modify the Study. It is up to the sponsor to decide whether a modification is to be regarded as substantial or not, based on the definitions given in the

Regulation. Any substantial modification of the Study, including the addition of a clinical trial site or the change of a principal investigator in the clinical trial site, can only be implemented in a Member State Concerned if it has been approved in accordance with the procedure set out in this Regulation.

Information on any changes to the clinical trials which are not substantial modifications but are relevant for the supervision of the clinical trials by the Member States Concerned shall be permanently updated in CTIS by the sponsor. A non-substantial modification is a modification without substantial impact on the safety or rights of the subjects and/or the reliability and robustness of the data, and the information is not necessary for oversight. This modification should not be reported as such.

18.1.5. Notifications

The sponsor (or its legal representative) shall notify through the CTIS the start of the Study, the end of recruitment and eventually the restart of recruitment, temporary halt and eventually restart, early termination, all unexpected events, urgent safety measures, end of the study, serious breaches.

18.1.6. Summary of results

When intermediate data analysis is provided for in the protocol (related to an adaptive or integrated design), a summary of these results has to be submitted in CTIS within one year after the completion of the intermediate data analysis. It is restricted to the endpoints of the intermediate analysis as defined in the Study protocol. The summary might be expanded if justified, e.g. dose selection in integrated protocols based on safety or/and pharmacological data.

Irrespective of the outcome of the Study, within one year from the end of the Study in all Member States concerned, the sponsor shall submit to CTIS both a summary of the results of the clinical trial and a summary for laypersons.

18.2. Informed consent form and procedure for obtaining subject's consent

18.2.1. The informed consent

The informed consent form (ICF) is based on the Belgian national template which is available on the web site of competent authority (AFMPS-FAGG).

The ICF describes in details the following items: the investigator and the sponsor, objectives and description of the study protocol (procedures, methods, IMP/interventions), course of the study, drugs received as treatment, risks and discomforts (possible adverse events and potential hazards of the study including risks related to a pregnancy), benefits, costs, notification of new information, interest to participate in the study, alternative treatment, withdrawal of the study, treatment after stopping or at the end of the study, publication of study results, contact details at the hospital, translational research, data protection clause insurance and liability, signatures.

The ICF is translated into the national language(s) of the participating country(ies) under the responsibility of the sponsor or its authorized delegate. Then the translations must be approved by an appropriate independent ethics committee/independent review board (IEC/IRB). Once approved, the ICF and the IRB/IEC's written approval/favourable opinion of the ICF are provided by the sponsor to the principal investigators in the national language(s) of their countries. It is

identified by a version number and a date, and it makes reference to the associated protocol (title of the clinical trial, EudraCT number, protocol number).

The principal investigators are not allowed to make any changes in the approved version of the ICF unless these changes are non-substantial for the sponsor, such as but not limited to adding the hospital logo or crest, completing a contact person's phone number.

The ICF to be provided to subjects shall be revised whenever important new information becomes available that may be relevant to the subject's consent. Any revised ICF shall receive the IRB/IEC's approval/favourable opinion in advance of use.

18.2.2. The procedure for obtaining subject's consent

NEO-CHECKRAY is an international, multicenter clinical trial. The terms and conditions described below are subject to certain variations depending on the country and its national laws, but also on the hospitals where the participants will be recruited.

There will be no direct advertising for research subjects, i.e., advertising that is intended to be seen or heard by prospective subjects to solicit their participation in a study. Direct advertising includes, but is not necessarily limited to: newspaper, radio, TV, bulletin boards, posters, and flyers.

However, for the sake of transparency, a description of this clinical trial will be available at http://www.ClinicalTrials.gov (USA). The information will be also available in a similar European website, http://euclinicaltrials.eu/. Such an information may be available on official national websites such as but not limited to the competent authorities websites (for example BELGIUM: https://banquededonneesessaiscliniques.be/fr), the website of the National Cancer Institutes (for example, FRANCE: http://www.e-cancer.fr), and some participating sites may also publish some information about the trial on their own websites.

The informed consent process can only start once the study has been given a written positive approval/favourable opinion by the IRB/IEC's and has been approved by the National Competent Authorities if required by national laws of the participating country(-ies). Also the Investigator's site initiation process must have been completed by the Sponsor or its authorized subcontractor(s) (including a signed contract between the site and the Sponsor and/or CRO). Subjects should be identified as potentially eligible by a healthcare professional that has clinical responsibility for the subject. This is to ensure medical confidentiality is adhered to. Subjects will be also assessed for their potential eligibility by a healthcare professional that is authorised to recruit to the study.

Once a subject has been assessed and identified as potentially eligible for the study, the subject should be approached and informed to the study.

The person informing the subject on the study must be familiar with all aspects of the study as described in the latest IEC/IRB approved version of the protocol.

The investigator, or another member of the investigating team, should discuss the study with the subject whereby the subject is given the opportunity to understand the objectives, risks and inconveniences of the trial and the conditions under which it is to be conducted. The information should be provided to subjects in both oral and written forms. The language used to inform the subject, both oral and written, should be concise, described in layman's terms and should be understandable to the subject and impartial witness/interpreter, when applicable. The person obtaining the informed consent must have the subject's medical notes and the current IEC/IRB approved versions of the ICF available during the discussion. Other study documents may also be used during the interview (e.g. subject diaries, study schedule sheets).

All subjects receive the appropriate version of the written information and are asked to read and review it.

Usually, if the subject is considering participating in a clinical trial, she may take the consent document home to discuss with family, friend or advocate.

All prospective subjects must have the cognitive ability to provide legally effective informed consent. For subjects not qualified to give or incapable of giving consent, written consent must be obtained from the legal representative. The legal representative is an individual authorized under applicable law to consent on behalf of a prospective subject, to the subject's participation in the clinical study. This person should be designated by judicial decision or by a mandate (signed and dated by the two parties). In default, the authorized person who may consent on behalf of a subject is, in decreasing order: (i) the spouse, legal cohabitant and (ii) child (adult), parent, brother or sister (adult). In the case where both the subject and her legal representative are unable to read, an impartial witness should be present during the entire informed consent discussion. An impartial witness is a person, who is independent of the study, who cannot be unfairly influenced by people involved in the study, who attends the informed consent process if the subject cannot read the ICF. After the subject and/or legal representative has/have orally consented to participation in the trial, the witness's signature on the form will attest that the information in the consent form was accurately explained and understood.

Neither the investigator nor any member of the study team shall coerce or unduly influence a subject to participate or to continue to participate in a study. The principal investigator or designee also explains to the subject that the participation to the study is voluntary and that the subject is free to refuse to enter the study or to withdraw from it at any time, for any reason without any impact on the subject's subsequent care. No claims shall be made, either explicitly or implicitly, that the drug, biologic or device is safe or effective for the purposes under investigation, or that the drug, biologic or device is known to be equivalent or superior to any other drug, biologic or device. It is the responsibility of the principal investigator or a person designated by the principal investigator (if acceptable by local regulations) to obtain a signed written informed consent from each potential subject prior any study related procedure being carried out. The informed consent is applicable throughout the subject's participation to the study. It is commonly accepted that subject should have a minimum of 24 hours between the date the ICF is provided to the subject and the actual date when the subject (and/or the subject's legally designated representative) signs the informed consent. The date and time the trial was discussed and the date the ICF was given to the subject must be documented in the medical notes.

The consent form must be signed prior to any study related pre-screening or screening procedures being performed. The person obtaining the subject's consent should also inform relevant team members of the treatment decision for the subject.

The written ICF must be dated and personally signed by the principal investigator or authorised sub-investigator and the subject giving consent (or subject legal representative or impartial witness).

The original copy of the signed ICF will be retained in the investigator's study file (ISF) and must be made available for monitoring, audit or inspection. A copy of the signed ICF is given to the subject.

In case of new information that might affect the subject's willingness to continue participating in the study or results in significant changes in the risk/benefit assessment, the ICF should be reviewed and updated if necessary. All subjects, including those already being treated, should be informed of the new information, given a copy of the revised form, and give their consent to continue in the study.

18.3. Subject identification

The principal investigator or any authorised study member staff must assure that subjects' identity is maintained confidential and that their identities are protected from unauthorised parties. Personal medical information is always treated as confidential.

On CRFs or any other study document, subjects shall not be identified by their names or initials or social security number or subject chart number, but always by the assigned subject study identification code (4-digits number) and their date of birth (either complete or partial as allowed by national laws) in order to avoid identification errors.

The principal investigator should create and maintain up-to-date in the Investigator's Study File (ISF) the Subject screening/enrolment/identification log. This ICH-GCP log is required for documenting chronically any screened subject, their name, their assigned study number in case of study enrolment or the reason in case of non-enrolment. A note is made in the hospital medical records that the subject is participating in the study.

The name of a subject is neither asked for nor recorded by the sponsor. If subjects' names or any other confidential subject information are included by error on copies of documents submitted to the sponsor, they are obliterated and the assigned subject study identification code added to the document by the sponsor. Moreover, the sponsor asks to the principal investigator or any authorised study member staff to also obliterate this information on the documents that he/she has submitted.

Documents (e.g. subjects' written Informed Consent Forms) not for submission to sponsor should be maintained by the principal investigator in strict confidence.

18.4. Privacy Guarantee

Personal data

If a subject participates in the study, his/her personal data will be processed; "processing" includes the collection, storage, structuring, transmission and any other use or provision. Personal data includes the following information:

- information that identifies the subject directly (such as his/her name, address, telephone number, or health insurance number);
- the age and sex of the subject;
- information relating to the subject's state of health, including medical history;
- the subject's medical treatments and his/her response to medical treatments;
- for women who become pregnant during the study: data about the pregnancy and childbirth;
- information about the subject's biological samples and the results of analyses performed on these;
- information about the subject's medical images and the results of the evaluation of these.

There will be different groups of data:

- The data group containing all data about the subject collected at the study hospital, including information that identifies him/her directly. This data group is called "**unencoded data**" and will be kept only in the study hospital's medical records.
- There will also be groups of "**encoded data**". The principle of data encoding entails that if a subject participates in the study, a code will be assigned to him/her. It will be unique, sequential and composed of 4 digits and it will replace all directly (surname, first name) or indirectly (telephone number or other number) identifying data on documents used for data collection or biological samples collected for the study. This encoding must be

applied before any transmission to a third party (the manager of the database collected here is the sponsor, i.e. Institut Jules Bordet, Brussels, Belgium).

Legal basis for the processing of subject data

The protection of subject data and the related rights are guaranteed by the General Data Protection Regulation (European Regulation 2016/679), by the law of 22 August 2002 concerning subject rights in Belgium, by the law of 30 July 2018 concerning the data privacy in Belgium as well as any (new) applicable legislation in the participating countries.

Data controller of subject data

The sponsor (Institut Jules Bordet, Brussels, Belgium) is the data controller of the subject's encoded data. The investigator and the hospital are, on the one hand, subcontractors of the sponsor with regard to the processing of the subject's encoded data and, on the other hand, data controllers of the subject's unencoded data collected both as part of the study and outside it.

Consultation and use of unencoded data

Unencoded data can be consulted by the investigator and other individuals from the hospital who are working on the study or providing care to the subject. Individuals accessing the data are subject to professional secrecy. In addition, a limited number of the sponsor's employees, or its contractual partners, ethics committees and regulatory authorities may consult the unencoded data, but solely for the purpose of verifying that the study is being conducted properly. These persons are bound by an obligation of confidentiality.

Consultation and use of encoded data

Encoded data will be transmitted to the sponsor. The encoded data can also be transmitted to service providers on the basis of contractual agreements. In addition, the encoded data may be shared with one or more partners collaborating with the sponsor with respect to the investigational drug and the diagnostic tests associated with this study's field of research.

The aforementioned recipients may use the encoded data for the following purposes:

- to answer scientific questions;
- to learn more about the investigational drug and how this group of drugs (i.e. drugs that may act in a similar way in the body) acts;
- to learn more about cancer and related health issues, and to develop corresponding diagnostic tests;
- to contribute to the correct planning of future studies;
- to develop scientific analysis methods;
- to publish the anonymised results of the study in scientific articles or presentations and to use them for educational purposes;
- to provide the sponsor of the study with informational materials on the study, the investigational drug, cancer and related health issues and diagnostic tests.

The subject's encoded data may be transmitted to regulatory bodies (including ethics committees) for regulatory purposes, for example for reporting relevant safety information, in order to receive marketing authorisation or for discussions on the reimbursement and marketing of the drug being tested and diagnostic tests. Regulatory bodies may also use the encoded data to learn more about cancer and related health issues.

Transfer of personal data to other countries

Some recipients of the data may be located outside your country and outside the European Economic Area (United States, for example). These may be countries whose level of data

protection has not been confirmed by the European Commission as adequate. In this case, security measures equivalent to the security measures required by Belgian and European regulations will be taken in order to protect subject rights in terms of data confidentiality, by entering into specific contractual agreements.

Storage of encoded data

Encoded data will be kept for at least 25 years after the end of the study. Subsequently, they may be kept for an additional period of time, for the above-mentioned scientific purposes or for any legal reason (change of obligations with regard to storage, for example).

Subject rights related to personal data

The subject may exercise the following rights related to his/her personal data:

- Request information about the processing of data about him/her; however, he/she may not be able to access some data before the end of the study, without the risk of being excluded from the study. This measure may be necessary to protect the scientific integrity of the study.
- Request the correction of the data about him/her if they are incorrect or incomplete. The subject has the right to restrict the processing of data about him/her.
- Request the transfer of his/her personal data to himself/herself or to someone else in a commonly usable format.
- Withdraw his/her consent at any time without giving a reason. The subject also has the right to end his/her participation in the study by no longer coming, without having to justify himself/herself. However, this is not withdrawal in this case. Withdrawal means that the subject is actively withdrawing from the study and withdraws consent for data processing. This shall not affect the legality of processing data about him/her based on his/her consent given before withdrawal. However, after his/her withdrawal, data about him/her will no longer be collected.
- At the same time as his/her withdrawal, the subject has the right to request the erasure of data about him/her if it is no longer necessary for the purposes of processing or if there is no other legal basis for their further processing.
- The subject also has the right to lodge a complaint concerning the way in which his/her data is processed with the supervisory authority of his country responsible for ensuring compliance with data protection legislation.

If the subject wishes to exercise one of his/her rights relating to data about him/her, or if the subject wishes to know more about the measures to protect his/her personal data, he/she can send a request to the investigator or to the data protection officer of the study hospital.The investigator or the data protection officer of the study hospital can contact the DPO of the Sponsor at dpo@hubruxelles.be.

19. DATA HANDLING AND RECORD KEEPING

19.1. Investigator’s files and subject’s clinical source documents

The principal investigator must maintain adequate and accurate records to enable the conduct of the study to be fully documented and the study data to be subsequently verified. These documents should be classified into two separate categories: (1) investigator’s study file (ISF); and (2) subject clinical source documents.

The ISF should be established according to the ICH-GCP E6 and should include, among other documents, the site staff delegated study task log established by the principal investigator, the subject screening/enrolment and identification log and signed ICF.

Subject clinical source documents (paper or electronic) are the documents where the data are recorded for the first time and include, but are not limited to, subject hospital/clinic records; clinical and office charts, laboratory notes, memoranda, quality of life questionnaires, evaluation checklists, pharmacy dispensing records, recorded data from automated instruments, copies of transcriptions that are certified after verification as being accurate and complete, microfiche, photographic negatives, microfilm or magnetic media, X-rays, patient files, and records kept at pharmacies, laboratories, and medico-technical departments involved in the study.

19.1.2 Data Quality Assurance

The sponsor is responsible for all data management activities of this study. This includes set- up and build of the electronic data capture system, quality checking of the database throughout study conduct, and study closeout.

The data related to the medical care of the subjects are recorded in the medical notes (source documents) by site staff. Thereafter the site staff enters in the eCRF only the data required to perform the statistical analyses.

The data related to the quality of life are collected on paper questionnaires (source documents). The data from the questionnaires are afterwards entered manually into the electronic data capture system by the site staff.

Site staff is responsible for manual data entry into the electronic data capture system. In the event of discrepant data, the sponsor issues queries to site staff, which site staff resolves electronically in the electronic data capture system in a timely manner.

The sponsor writes a Data Management Plan that describes the quality checks to be performed on the data.

The study data are transferred to the statistical team prior to any study committees data reviews or prior to analyses. The electronic transfer of data is processed by using a tool designed by the sponsor which ensures a secured transfer of the data in Statistical Analysis System (SAS) format.

19.2. Case Report Forms (CRF)

Data for this study are captured by using an electronic CRF (eCRF) via a sponsor designated electronic data capture system for each subject enrolled. The site receives training, an eCRF completion guideline for appropriate eCRF handling and completion and personal access code by the sponsor.

The eCRF should only be completed by designated and trained site staff and submitted electronically to sponsor.

The principal investigator is responsible for creating and maintaining up to date the site staff delegated study task log (named Signature Log in ICH GCP) mentioning the responsible persons completing, updating and/or signing the eCRF.

Once completed, the eCRF must be reviewed and electronically signed by the principal investigator or an authorised delegate from the study staff. This also applies to records for those subjects who fail to complete the study. If a subject withdraws from the study treatment or does not complete the follow-up, the reason must be noted in the eCRF. If a subject withdraws from the entire study, the withdrawal of consent should be noted in the eCRF.

The principal investigator should ensure the accuracy, completeness, legibility and timeliness of the data reported in the eCRF and in all required reports. Data reported in the eCRF must be derived from source documents and should be consistent with the source documents.

An audit trail will maintain a record of the initial entries and changes made, reasons for change (if necessary), time and date of entry, and user name of the person adding or changing an entry.

At the end of the study, the investigator will receive subject data entered in the eCRF for his or her site in a readable format that must be kept with the study records. Acknowledgement of receipt of these documents is required.

19.2.1 Source data documentation

The site staff should maintain adequate and accurate source documents, which should be attributable, legible, contemporaneous, original, accurate, and complete, as referenced in ICH E6 (R2) in Section 8.

Source documents (paper or electronic) are those in which patient data are recorded and documented for the first time. They include, but are not limited to, hospital records, clinical and office charts, laboratory notes, memoranda, quality of life questionnaires, evaluation checklists, pharmacy dispensing records, recorded data from automated instruments, copies of transcriptions that are certified after verification as being accurate and complete, microfiche, photographic negatives, microfilm or magnetic media, X-rays, patient files, and records kept at pharmacies, laboratories, and medico-technical departments involved in the study.

Before study initiation, the types of source documents that are to be generated is defined. This includes any protocol data to be entered directly into the eCRFs (i.e., no prior written or electronic record of the data) and considered source data

Source documents that are required to verify the validity and completeness of data entered into the eCRFs must not be obliterated or destroyed and must be retained per the policy for retention of records described in Section 19.4.

When clinical observations are entered directly into a study site's computerized medical record system (i.e., in lieu of original hardcopy records), the electronic record can serve as the source document if the system has been validated in accordance with health authority requirements pertaining to computerized systems used in clinical research. An acceptable computerized data collection system allows preservation of the original entry of data. If original data are modified, the system should maintain a viewable audit trail that shows the original data as well as the reason for the change, name of the person making the change, and date of the change.

19.3. Retention of documents

Following closure of the study, the principal investigator must maintain all site study records, except for those required by local regulations to be maintained by someone else, in a safe and secure location. The records must be maintained to allow easy and timely retrieval, when needed (e.g., audit or inspection), and, whenever feasible, to allow any subsequent review of data in conjunction with assessment of the facility, supporting systems, and staff. Where permitted by local laws/regulations or institutional policy, some or all of these records can be maintained in a format other than hard copy (e.g., microfiche, scanned, electronic); however, caution needs to be exercised before such action is taken. The principal investigator must ensure that all reproductions are legible and are a true and accurate copy of the original and meet accessibility and retrieval standards, including re-generating a hard copy, if required. Furthermore, the principal investigator must ensure there is an acceptable back-up of these

reproductions and that an acceptable quality control process exists for making these reproductions.

The sponsor informs the principal investigator of the time period for retaining these records to comply with all applicable regulatory requirements. The minimum retention time meets the strictest standard applicable to that participating site for the study, as dictated by any institutional requirements or national laws or regulations; otherwise, the retention period will default to 20 years after the completion of the study and/or after approval by relevant Health Authorities, whichever is longer.

Should the principal investigator wish to assign the study records to another party or move them to another location, the sponsor must be notified in advance. If the principal investigator cannot guarantee this archiving requirement at the participating site for any or all of the documents, special arrangements must be made between the principal investigator and the sponsor to store these in a sealed container(s) outside of the participating site so that they can be returned sealed to the principal investigator, in case of a regulatory audit. Where source documents are required for the continued care of the subject; appropriate copies should be made before storing outside of the participating site.

19.4. Adequate arrangements to secure data compliant with GDPR

All “personal data” are treated in accordance with data protection laws, including the General Data Protection Regulation (GDPR).

Personal and clinical data related to our studies are stored on secured servers. Those servers protect data against loss, destruction, access, modification or dissemination by unauthorized persons. Only a limited and controlled number of persons are authorized to access the data. The Audit Trail function integrated in the data collection system used for the study allows verifying that non authorized data modification didn’t occur.All data transmitted between servers and client for the CTSU applications are encrypted by the TLS (Transport Layer Security) protocol.To ensure the compliance with GDPR, the IT Department of the Sponsor has implemented the following set of measures:

- A rigorous policy of password management. Passwords must be at least 8 characters including numbers, letters (with at least 1 uppercase and 1 lowercase) and special characters, and must be renewed after a maximum of 180 days.
- A procedure of creation and deletion of user accounts. Access to the workstations and applications must be done using registered user accounts, and not “generic” (account1, compta2...), in order to be able to trace the actions done on a file and empower all stakeholders.
  This rule is also applying to systems and networks administrators of the sponsor.
- Secured workstations. Workstation are locked automatically after a period of inactivity (10 minutes). This measure is in place to restrict the risks of fraudulent use of an application in case of temporary absence of the user.
- A precise identification of persons who can access the files. Access to personal data processed in a file is limited to persons who can legitimately access them for the execution of their assigned tasks. A periodic check of application profiles and access rights on the servers is done to ensure the adequacy of the offered rights and the reality of the functions occupied by each user.
- A secured Local Area network. Institut Jules Bordet network is secured against external attacks. Logical safety devices such as filter routers (ACLs), firewalls, intrusion probes, etc. ensure a first level of protection. Reliable protection against viruses and spyware are constantly updated, both on the server and on the user’s workstations.

- Remote accesses to the information system are authenticated by the user and the workstation. Internet access to our tools needs to pass by strong security measures, including the use of IPsec, SSL/TLS, or HTTPS protocols and 2 factors authentication for remote access.
- Secured physical access to facilities. Access to sensitive premises, such as rooms hosting computer servers and network components, is limited to authorized personnel. These premises are subject to special security: locked doors, digicode, and access controlled by name badges. Anticipating the risk of loss or disclosure of data. All of our servers are subjects to regular backup. The backup media are stored in a fireproof safe room separated from the one that hosts the servers. An "emergency-rescue" procedure describes how to quickly reassemble these servers in the event of a major crash or disaster.Information secure policy. All the rules related to computer security are formalized in a document (SOP) accessible to all users of the sponsor.

19.5. Measures implemented in case of data security breach

19.5.1. The procedure if a Data Processor causes a data breach

All agreements with Data Processors stipulate that the Data Processor must notify every data breach to the Data Controller (the Sponsor). In the event a data breach occurs and that breach was caused by a Data Processor, the Sponsor shall assess with the Data Processor if the breach poses a risk to an individual's rights and freedoms. The risk assessment shall be performed with the assistance of the Data Processor and shall evaluate if a notification to the supervisory authority is necessary pursuant to articles 33 and 34 of the GDPR and the Guidelines 01/2021 on Examples regarding Personal Data Breach Notification (Adopted on 14 December 2021, version 2.0). The risk assessment shall be carried out using the methodology developed by the European Union Agency for Network and Information Security (ENISA) for personal data breach severity assessment.

The Sponsor shall contact the Data Processor to request a corrective and preventive actions plan. The Sponsor shall request the Data Processor to provide without undue delay the following information:

- The up-to-date name and contact details of the data protection officer (DPO) or other contact point where more information can be obtained;
- A description of the likely consequences of the personal data breach;
- A description of the measures taken or proposed to address the personal data breach, including, where appropriate, measures to mitigate its possible adverse effects.

19.5.2. Notification of the data breach

The Sponsor shall notify the supervisory authority without undue delay, and at the latest within 72 hours after having become aware of the breach, if the breach poses a risk to the individual's rights and freedoms. If the data breach only has minor consequences and no significant effect on the data subjects, the Sponsor shall not notify the supervisory authority but only document the breach by keeping record of the risk assessment, and filling the notification form for information.

If the data breach poses a high risk to those individuals affected, the Sponsor shall inform those individuals in clear, plain language without undue delay, unless there are effective technical and organizational protection measures that have been put in place, or other

measures that ensure that risk is no longer likely to materialize. The individuals shall also be informed upon request from the supervisory authority.

The information to individuals includes the DPO's details, a description of the likely consequences of the breach and the measures taken (including mitigating actions and any possible adverse effects). This information shall be given by the Data Processor where the confidentiality of the individuals should be preserved.

19.5.3. Risk assessment

The risk shall be assessed in accordance with the Guidelines 01/2021 and the methodology developed by ENISA. In particular, the risk assessment shall take into considerations:

- If the breach derives from an intentional action or from an unintentional human error caused by inattentiveness;
- The quantity of data affected by the breach and the type of personal data
- The low or high amount of individuals affected
- The immediate detection of the breach
- If the receiving party is bound by professional secrecy, reported the breach himself and deleted the file and personal data upon request
- The relationship between the Sponsor and the receiving party

19.5.4. Measures to mitigate the adverse effect

Depending on the type of data breach, the measures taken to mitigate the risks may include the following:

- Limit the access to the most sensible data
- enforcing training, education and awareness programs
- avoiding data exchange through unsecure channels
- implementing measures to force user authentication when accessing sensitive personal data checking unusual dataflow between the file server and employee workstations
- Enforcing GDPR compliance program with security standards such as ISO 27000 series, COSO, CSA, CCM, HIPAA, NIST.

19.5.5. Record keeping

The Sponsor shall document any personal data breaches, comprising the facts relating to the personal data breach, its effects and the remedial action taken. That documentation shall enable the supervisory authority to verify compliance.

20. STUDY OVERSIGHT

20.1. Independent Data Monitoring Committee

An Independent Data Monitoring Committee (IDMC) will be formed for this study to review data related to the safety run-in and phase II. Sponsor employees, as well as other individuals involved in the design, setup, or conduct of the study, will be excluded from IDMC membership. The IDMC members shall be familiar with the methodology of oncology trials.

The following IDMCs are planned:

**IDMC number**	**Timepoint in study**	**Goal of IDMC**

1	At the end of the safety run-in (as detailed in section 3.2.1)<br><br>*NOTE: this IDMC held place on 24/09/2020. The results of the IDMC are discussed in section 12.2.1.*	Safety analysis and take decision whether to proceed to phase II randomized trial as detailed in section 3.2.1.<br><br>*IDMC conclusion on 24/09/2020: agree to proceed to phase II.*
2	Randomisation of 40 subjects in the phase II randomized trial<br>*NOTE: this IDMC held place on 16/09/2022. The results of the IDMC are discussed in section 12.2.2.*	Safety data analysis of the subjects included in the phase II randomized trial.<br>*IDMC conclusion on 16/09/2022: agree to continue the trial.*
3	22 subjects in every treatment arm underwent surgery in the phase II randomized trial.<br>This is at 50% information rate and is approximately equivalent to 66 subjects that underwent surgery in the phase II randomized trial.<br><br>*NOTE: this IDMC held place on 16/09/2023. The resuls of the IDMC are discussed in section 12.2.3.*	- Interim analysis for futility (see section 12.2)<br>- Safety data analysis of the subjects included in the phase II randomized trial.<br><br>*IDMC conclusion on 16/09/2023: agree to continue the trial to full accrual. According to the prespecified criteria for futility all arms can continue in the trial.*

The outcome of the first data review after the enrolment of 6 subjects in the safety run-in will decide if the study will continue in to the phase II. This information will be communicated in a timely manner to the sponsor. When necessary, the sponsor shall communicate the relevant information to investigators for notification of their respective Ethics Committees and/or for notification of the Regulatory Authority if required by the national laws of the countries where the study is conducted.

The IDMC will follow a charter that outlines the IDMC roles, responsibilities, operating procedures for the meetings and the flow of communication.

20.2. Scientific Committee

A scientific committee including the study chair, co-chair, Institut Jules Bordet study team, principal investigators and study team of participating centres will be constituted to monitor data and overall safety to ensure that the study meets the highest standards of ethics and subject safety.

During the safety run-in, the scientific committee will evaluate the safety, tolerability and all other data concerning the treatment with paclitaxel, durvalumab and oleclumab and SBRT.

In Phase II, the same scientific committee is planned to review safety and events (relapse, deaths, premature treatment stops). For this purpose, teleconferences and or meetings as well as written reports are planned every 6 months. Additional teleconferences can be organized if necessary for safety concerns.

20.3. Monitoring of sites and subjects' compliance

20.3.1. Monitoring of sites

A monitoring plan containing all the types of monitoring visits to be conducted, the timing and the frequency of the visits, and the study documentation/materials that should be reviewed at each visit is written by the Sponsor monitoring team. This monitoring plan is written based on the considerations such as:

- the risk assessment of the study relative to standard care and the extent of knowledge about the investigational medicines tested
- the nature, the complexity, the requirement and the end-points of the study,
- the study phase
- the blinding procedure
- the critical data and processes
- the recruitment rate of the investigational site,
- the study financial resources
- the site performance (e.g. delay in data entry, recurrent missing protocol assessments, high rate of data queries and etc.).

All monitoring activities on participating investigational sites are carried out using this Monitoring Plan and must be compliant with the current approved study protocol, Sponsor procedures, ICH-GCP, any study specific guideline/procedure and local regulation. It is applicable from the study initiation until the study closure and remains valid for subsequent protocol amendments unless these amendments are implying a need for updating this plan.

At the beginning of the study, a training session will be organised for the study site staff members of each participating site. The manuals detailing the instructions to conduct the study and to use the study tools and procedures will be also explained during this training session and given to the study site staff members.

During the study, several monitoring visits will be carried-out remotely and/or on-site. In line with the activities level described in the Monitoring Plan, the study monitors will perform source data review and source data verification to confirm that critical protocol data entered into the eCRFs by authorized site personnel are accurate, complete, and verifiable from source documents and the accuracy and completeness of the source documents.

At the end of the study (or earlier if required by the Sponsor and/or CA), a close-out site visit will be carried-out to ensure the proper site closure.

20.3.2. Subjects' compliance

At study level, the subjects' protocol compliance will be checked by the Sponsor monitoring team and the data managers team based on the CRF data reported by the sites.

21. STUDY SPONSORSHIP AND FINANCING

Within the European Union, Institut Jules Bordet will be the legal sponsor of the Study, as that term is defined under the Regulation EU No 536/2014.

Institut Jules Bordet is solely responsible for any and all safety reporting associated with the conduct of the Study, as well as any and all regulatory obligations associated with the conduct of the Study, except for those obligations expressly identified in specific Agreements made with third parties.

This study is supported by a grant from AstraZeneca which will also provide oleclumab and durvalumab free of charge to all study subjects.

22. STUDY INSURANCE

The sponsor is responsible for taking out the insurance for the study according to Regulation EU No 536/2014 and also the laws of the countries where the study is conducted. An insurance certificate must be available to the participating sites at the time of study initiation. Clinical study insurance is only valid in participating sites authorised by the sponsor.

The insurance of the sponsor does not relieve the participating site and the principal investigator of any obligation to maintain their own liability insurance policy.

23. PUBLICATION POLICY

Publication and presentations of any results from this study shall be in accordance with accepted scientific practice, academic standards and customs and in accordance with the specific policy developed for this study if any. The final publication of the study results is written by the study chair or designated active principal investigators of the study on the basis of the final analysis performed at the sponsor headquarters (Institut Jules Bordet, Brussels, Belgium).

The sponsor recognises right of utilising data derived from the Study for teaching purposes, communication at congresses and scientific publications.

Nevertheless, in order to ensure the accuracy and scientific value of the information, while preserving the independence and accountability of the study chair and the confidentiality of the information, only clear, checked and validated data shall be used. To that effect, it is essential that the study chair and the sponsor exchange and discuss, prior to any publication or communication, any draft publication or communication made by the study chair.

All manuscripts shall include an appropriate acknowledgment section, mentioning all principal investigators who have contributed to the study, the staff of the Clinical Trials Support Unit involved in the study, as well as the supporting bodies. The number of acknowledgments per participating entity shall depend on the journal's rules and be based on fair and practical considerations.

24. REFERENCES

1. Sorlie T, Perou CM, Tibshirani R, Aas T, Geisler S, Johnsen H, et al. Gene expression patterns of breast carcinomas distinguish tumor subclasses with clinical implications. Proc Natl Acad Sci U S A. 2001;98(19):10869–74.
2. Bonnefoi H, Litière S, Piccart M, MacGrogan G, Fumoleau P, Brain E, et al. Pathological complete response after neoadjuvant chemotherapy is an independent predictive factor irrespective of simplified breast cancer intrinsic

subtypes: a landmark and two-step approach analyses from the EORTC 10994/BIG 1-00 phase III trial. Annals of Oncology. 2014 Jun;25(6):1128–36.

3. Gianni L, Pienkowski T, Im YH, Roman L, Tseng LM, Liu MC, et al. Efficacy and safety of neoadjuvant pertuzumab and trastuzumab in women with locally advanced, inflammatory, or early HER2-positive breast cancer (NeoSphere): A randomised multicentre, open-label, phase 2 trial. Lancet Oncol. 2012;13(1):25–32.

4. Poggio F, Bruzzone M, Ceppi M, Pondé NF, La Valle G, Del Mastro L, et al. Platinum-based neoadjuvant chemotherapy in triple-negative breast cancer: A systematic review and meta-analysis. Annals of Oncology. 2018;29(7):1497–508.

5. Cortazar P, Zhang L, Untch M, Mehta K, Costantino JP, Wolmark N, et al. Pathological complete response and long-term clinical benefit in breast cancer: The CTNeoBC pooled analysis. The Lancet [Internet]. 2014;384(9938):164–72. Available from: http://dx.doi.org/10.1016/S0140-6736(13)62422-8

6. Gajewski TF, Corrales L, Williams J, Horton B, Sivan A, Spranger S. Cancer Immunotherapy Targets Based on Understanding the T Cell-Inflamed Versus Non-T Cell-Inflamed Tumor Microenvironment BT - Tumor Immune Microenvironment in Cancer Progression and Cancer Therapy. In: Kalinski P, editor. Cham: Springer International Publishing; 2017. p. 19–31. Available from: https://doi.org/10.1007/978-3-319-67577-0_2

7. Rugo HS, Delord JP, Im SA, Ott PA, Piha-Paul SA, Bedard PL, et al. Safety and antitumor activity of pembrolizumab in patients with estrogen receptor–positive/human epidermal growth factor receptor 2–negative advanced breast cancer. Clinical Cancer Research. 2018;24(12):2804–11.

8. Chen DS, Mellman I. Elements of cancer immunity and the cancer-immune set point. Nature. 2017;541(7637):321–30.

9. Dirix LY, Takacs I, Jerusalem G, Nikolinakos P, Arkenau HT, Forero-Torres A, et al. Avelumab, an anti-PD-L1 antibody, in patients with locally advanced or metastatic breast cancer: a phase 1b JAVELIN Solid Tumor study. Breast Cancer Res Treat. 2017;1–16.

10. Nanda R, Liu MC, Yau C, Shatsky R, Pusztai L, Wallace A, et al. Effect of Pembrolizumab Plus Neoadjuvant Chemotherapy on Pathologic Complete Response in Women With Early-Stage Breast Cancer. JAMA Oncol. 2020;60637.

11. Allard B, Turcotte M, Spring K, Pommey S, Royal I, Stagg J. Anti-CD73 therapy impairs tumor angiogenesis. International journal of cancer Journal international du cancer. 2014 Mar;134(6):1466–73.

12. Sharabi AB, Lim M, DeWeese TL, Drake CG. Radiation and checkpoint blockade immunotherapy: radiosensitisation and potential mechanisms of synergy. The Lancet Oncology. 2015;16(13):e498–509.

13. Ngwa W, Irabor OC, Schoenfeld JD, Hesser J, Demaria S, Formenti SC. Using immunotherapy to boost the abscopal effect. Nat Rev Cancer [Internet]. 2018;18(5):313–22. Available from: http://www.nature.com/doifinder/10.1038/nrc.2018.6

14. Wennerberg E, Kawashima N, Demaria S. Adenosine regulates radiation therapy-induced anti-tumor immunity. J Immunother Cancer [Internet]. 2015;3(Suppl 2):P378. Available from: http://www.immunotherapyofcancer.org/content/3/S2/P378

15. Symmans WF, Peintinger F, Hatzis C, Rajan R, Kuerer H, Valero V, et al. Measurement of residual breast cancer burden to predict survival after neoadjuvant chemotherapy. Journal of Clinical Oncology. 2007;25(28):4414–22.

16. Haque R, Ahmed SA, Inzhakova G, Shi J, Avila C, Polikoff J, et al. Impact of breast cancer subtypes and treatment on survival: An analysis spanning two decades. Cancer Epidemiology Biomarkers and Prevention. 2012;21(10):1848–55.

17. Haque W, Verma V, Hatch S, Suzanne Klimberg V, Brian Butler E, Teh BS. Response rates and pathologic complete response by breast cancer molecular subtype following neoadjuvant chemotherapy. Breast Cancer Research and Treatment. 2018;170(3):559–67.

18. Woo SR, Corrales L, Gajewski TF. The STING pathway and the T cell-inflamed tumor microenvironment. Trends Immunol [Internet]. 2015;36(4):250–6. Available from: http://dx.doi.org/10.1016/j.it.2015.02.003

19. Nanda R, Chow LQM, Dees EC, Berger R, Gupta S, Geva R, et al. Pembrolizumab in Patients With Advanced Triple-Negative Breast Cancer: Phase Ib KEYNOTE-012 Study. Journal of clinical oncology : official journal of the American Society of Clinical Oncology. 2016 Jul 20;34(21):2460–7.

20. Liu J, Blake SJ, Yong MCR, Harjunpää H, Ngiow SF, Takeda K, et al. Improved efficacy of neoadjuvant compared to adjuvant immunotherapy to eradicate metastatic disease. Cancer Discovery. 2016;6(12):1382–99.

21. Von Minckwitz G, Untch M, Blohmer JU, Costa SD, Eidtmann H, Fasching PA, et al. Definition and impact of pathologic complete response on prognosis after neoadjuvant chemotherapy in various intrinsic breast cancer subtypes. Journal of Clinical Oncology. 2012;30(15):1796–804.

22. Korn EL, Sachs MC, McShane LM. Statistical controversies in clinical research: Assessing pathologic complete response as a trial-level surrogate end point for early-stage breast cancer. Annals of Oncology. 2016;27(1):10–5.

23. Jatoi I, Benson JR, Kunkler I. Hypothesis: can the abscopal effect explain the impact of adjuvant radiotherapy on breast cancer mortality? npj Breast Cancer. 2018;4(1):8.

24. Powles T, O'Donnell PH, Massard C, Arkenau HT, Friedlander TW, Hoimes CJ, et al. Efficacy and Safety of Durvalumab in Locally Advanced or Metastatic Urothelial Carcinoma. JAMA Oncology. 2017;3(9):e172411.

25. Zandberg D, Algazi A, Jimeno A, Good JS, Fayette J, Bouganim N, et al. 1042ODurvalumab for recurrent/metastatic (R/M) head and neck squamous cell carcinoma (HNSCC): Preliminary results from a single-arm, phase 2 study. Annals of Oncology. 2017 Sep;28(suppl_5).

26. Hao D, Juergens R, Laurie S, Mates M, Tehfe M, Bradbury P, et al. A Canadian Cancer Trials Group phase IB study of durvalumab with or without tremelimumab + standard platinum-doublet chemotherapy in patients with advanced, incurable solid malignancies (IND.226). European Journal of Cancer. 2016 Dec;69:S95.

27. Bendell JC, Reddy V, Tavakkoli F, Leow CC, Li X, Kumar R, et al. A phase 1 study to evaluate the safety, tolerability, pharmacokinetics, immunogenicity, and antitumor activity of MEDI9447 alone and in combination with durvalumab (MEDI4736) in patients with advanced solid tumors. Journal of Clinical Oncology. 2016 May;34(15_suppl):TPS3096–TPS3096.

28. Loibl S, Untch M, Burchardi N, Huober JB, Blohmer JU, Grischke EM, et al. A randomized phase II neoadjuvant study (GeparNuevo) to investigate the addition of durvalumab, a PD-L1 antibody, to a taxane-anthracycline containing chemotherapy in triple negative breast cancer (TNBC). Journal of Clinical Oncology. 2017 May;35(15_suppl):3062.

29. Loibl S, Untch M, Burchardi N, Huober JB, Blohmer JU, Grischke EM, et al. Randomized phase II neoadjuvant study (GeparNuevo) to investigate the addition of durvalumab to a taxane-anthracycline containing chemotherapy in triple negative breast cancer (TNBC). Journal of Clinical Oncology. 2018 May 20;36(15_suppl):104.

30. Antonia SJ, Villegas A, Daniel D, Vicente D, Murakami S, Hui R, et al. Overall Survival with Durvalumab after Chemoradiotherapy in Stage III NSCLC. New England Journal of Medicine. 2018;NEJMoa1809697.

31. Loi S, Curigliano G, Salgado RF, Romero Diaz RI, Delaloge S, Rojas C, et al. LBA20 A randomized, double-blind trial of nivolumab (NIVO) vs placebo (PBO) with neoadjuvant chemotherapy (NACT) followed by adjuvant endocrine therapy (ET) ± NIVO in patients (pts) with high-risk, ER+ HER2− primary breast cancer (BC). Annals of Oncology [Internet]. 2023 Oct 1;34:S1259–60. Available from: https://doi.org/10.1016/j.annonc.2023.10.010

32. Cardoso F, McArthur HL, Schmid P, Cortés J, Harbeck N, Telli ML, et al. LBA21 KEYNOTE-756: Phase III study of neoadjuvant pembrolizumab (pembro) or placebo (pbo) + chemotherapy (chemo), followed by adjuvant pembro or pbo + endocrine therapy (ET) for early-stage high-risk ER+/HER2– breast cancer. Annals of Oncology [Internet]. 2023 Oct 1;34:S1260–1. Available from: https://doi.org/10.1016/j.annonc.2023.10.011

33. Herbst RS, Majem M, Barlesi F, Carcereny E, Chu Q, Monnet I, et al. COAST: An Open-Label, Phase II, Multidrug Platform Study of Durvalumab Alone or in Combination With Oleclumab or Monalizumab in Patients With Unresectable, Stage III Non–Small-Cell Lung Cancer. Journal of Clinical Oncology. 2022;

34. Cascone T, García-Campelo R, Spicer J, Weder W, Daniel D, Spigel D, et al. Abstract CT011: NeoCOAST: open-label, randomized, phase 2, multidrug platform study of neoadjuvant durvalumab alone or combined with novel agents in patients (pts) with resectable, early-stage non-small-cell lung cancer (NSCLC). Cancer Research. 2022 Jun 15;82(12_Supplement):CT011–CT011.

35. Emens L, Powderly J, II, Fong L, Brody J, Forde P, et al. CPI-444, an oral adenosine A2a receptor (A2AR) antagonist, demonstrates clinical activity in patients with advanced solid tumors. Abstract CT119, AACR 2017.

36. Bondiau PY, Courdi A, Bahadoran P, Chamorey E, Queille-Roussel C, Lallement M, et al. Phase 1 clinical trial of stereotactic body radiation therapy concomitant with neoadjuvant chemotherapy for breast cancer. International journal of radiation oncology, biology, physics. 2013;85(5):1193–9.

37. Charaghvandi RK, Yoon SM, van Asselen B, den Hartogh MD, van den Bongard HJGD. Single dose external beam preoperative radiotherapy in breast cancer: experience and guidelines. Radiotherapy and Oncology. 2017;123(Supplement 1):S164.

38. Vasmel JE, Charaghvandi RK, Houweling AC, Philippens MEP, van Asselen B, Vreuls CPH, et al. Tumor Response After Neoadjuvant Magnetic Resonance

Guided Single Ablative Dose Partial Breast Irradiation. International Journal of Radiation Oncology Biology Physics. 2020;1–9.

39. Correa C, Harris EE, Leonardi MC, Smith BD, Taghian AG, Thompson AM, et al. Accelerated Partial Breast Irradiation: Executive summary for the update of an ASTRO Evidence-Based Consensus Statement. Practical Radiation Oncology. 2017;7(2):73–9.

40. Poleszczuk J, Luddy K, Chen L, Lee JK, Harrison LB, Czerniecki BJ, et al. Neoadjuvant radiotherapy of early-stage breast cancer and long-term disease-free survival. Breast Cancer Research. 2017;19(1):75.

41. Lightowlers S V., Boersma LJ, Fourquet A, Kirova YM, Offersen B V., Poortmans P, et al. Preoperative breast radiation therapy: Indications and perspectives. European Journal of Cancer. 2017;82:184–92.

42. Kok M, Voorwerk L, Horlings H, Sikorska K, van der Vijver K, Slagter M, et al. Adaptive phase II randomized trial of nivolumab after induction treatment in triple negative breast cancer (TONIC trial): Final response data stage I and first translational data. Journal of Clinical Oncology. 2018 May 20;36(15_suppl):1012.

43. Schmid P, Adams S, Rugo HS, Schneeweiss A, Barrios CH, Iwata H, et al. Atezolizumab and Nab-Paclitaxel in Advanced Triple-Negative Breast Cancer. New England Journal of Medicine. 2018;NEJMoa1809615.

44. Schmid P, Cortes J, Pusztai L, McArthur H, Kümmel S, Bergh J, et al. Pembrolizumab for early triple-negative breast cancer. New England Journal of Medicine. 2020;382(9):810–21.

45. Schmid P, Cortes J, Dent R, Pusztai L, McArthur H, Kümmel S, et al. Event-free Survival with Pembrolizumab in Early Triple-Negative Breast Cancer. New England Journal of Medicine. 2022 Feb 10;386(6):556–67.

46. Hay CM, Sult E, Huang Q, Mulgrew K, Fuhrmann SR, McGlinchey KA, et al. Targeting CD73 in the tumor microenvironment with MEDI9447. Oncoimmunology. 2016 Aug;5(8):e1208875.

47. Overman MJ, LoRusso P, Strickler JH, Patel SP, Clarke SJ, Noonan AM, et al. Safety, efficacy and pharmacodynamics (PD) of MEDI9447 (oleclumab) alone or in combination with durvalumab in advanced colorectal cancer (CRC) or pancreatic cancer (panc). Journal of Clinical Oncology. 2018 May;36(15_suppl):4123–4123.

48. Senkus E, Kyriakides S, Ohno S, Penault-Llorca F, Poortmans P, Rutgers E, et al. Primary breast cancer: ESMO Clinical Practice Guidelines for diagnosis, treatment and follow-up. Annals of Oncology. 2015;26(March):v8–30.

49. Kodumudi KN, Woan K, Gilvary DL, Sahakian E, Wei S, Djeu JY. A novel chemoimmunomodulating property of docetaxel: Suppression of myeloid-derived suppressor cells in tumor bearers. Clinical Cancer Research. 2010;16(18):4583–94.

50. Fucikova J, Kralikova P, Fialova A, Brtnicky T, Rob L, Bartunkova J, et al. Human tumor cells killed by anthracyclines induce a tumor-specific immune response. Cancer Research. 2011;71(14):4821–33.

51. Samanta D, Park Y, Ni X, Li H, Zahnow CA, Gabrielson E, et al. Chemotherapy induces enrichment of $CD47^+$/$CD73^+$/$PDL1^+$ immune evasive triple-negative breast cancer cells. Proceedings of the National Academy of Sciences. 2018 Feb;115(6):E1239–48.

Docusign Envelope ID: 8E3E1A28-D1DF-4A9A-A65F-9548F273A2D6

52. Loi S, Pommey S, Haibe-Kains B, Beavis PA, Darcy PK, Smyth MJ, et al. CD73 promotes anthracycline resistance and poor prognosis in triple negative breast cancer. Proceedings of the National Academy of Sciences of the United States of America. 2013 Jul;110(27):11091–6.

53. Pilones KA, Vanpouille-Box C, Demaria S. Combination of Radiotherapy and Immune Checkpoint Inhibitors. Semin Radiat Oncol [Internet]. 2015;25(1):28–33. Available from: http://dx.doi.org/10.1016/j.semradonc.2014.07.004

54. Derer A, Frey B, Fietkau R, Gaipl US. Immune-modulating properties of ionizing radiation: rationale for the treatment of cancer by combination radiotherapy and immune checkpoint inhibitors. Cancer Immunology, Immunotherapy. 2016;65(7):779–86.

55. Vatner RE, Cooper BT, Vanpouille-Box C, Demaria S, Formenti SC. Combinations of Immunotherapy and Radiation in Cancer Therapy. Front Oncol [Internet]. 2014;4(November). Available from: http://journal.frontiersin.org/article/10.3389/fonc.2014.00325/abstract

56. Garnett CT, Palena C, Chakarborty M, Tsang K yok, Schlom J, Hodge JW. Sublethal Irradiation of Human Tumor Cells Modulates Phenotype Resulting in Enhanced Killing by Cytotoxic T Lymphocytes Sublethal Irradiation of Human Tumor Cells Modulates Phenotype Resulting in Enhanced Killing by Cytotoxic T Lymphocytes. Cancer Res. 2004;64:7985–94.

57. Filatenkov A, Baker J, Mueller AMS, Kenkel J, Ahn GO, Dutt S, et al. Ablative Tumor Radiation Can Change the Tumor Immune Cell Microenvironment to Induce Durable Complete Remissions. Clinical Cancer Research [Internet]. 2015 Aug [cited 2015 Nov 4];21(16):3727–39. Available from: http://clincancerres.aacrjournals.org/cgi/doi/10.1158/1078-0432.CCR-14-2824

58. Sharabi AB, Nirschl CJ, Kochel CM, Nirschl TR, Francica BJ, Velarde E, et al. Stereotactic Radiation Therapy Augments Antigen-Specific PD-1-Mediated Antitumor Immune Responses via Cross-Presentation of Tumor Antigen. Cancer immunology research. 2015;3(4):345–55.

59. Reits EA, Hodge JW, Herberts CA, Groothuis TA, Chakraborty M, Wansley EK, et al. Radiation modulates the peptide repertoire, enhances MHC class I expression, and induces successful antitumor immunotherapy. J Exp Med [Internet]. 2006;203(5):1259–71. Available from: http://www.pubmedcentral.nih.gov/articlerender.fcgi?artid=3212727&tool=pmcentrez&rendertype=abstract

60. Kuwabara M, Takahashi K, Inanami O. Induction of Apoptosis through the Activation of SAPK/JNK Followed by the Expression of Death Receptor Fas in X-irradiated Cells. Journal of Radiation Research. 2003;44(3):203–9.

61. Vanpouille-Box C, Alard A, Aryankalayil MJ, Sarfraz Y, Diamond JM, Schneider RJ, et al. DNA exonuclease Trex1 regulates radiotherapy-induced tumour immunogenicity. Nat Commun [Internet]. 2017;8:15618. Available from: http://www.nature.com/doifinder/10.1038/ncomms15618

62. Vaupel P, Multhoff G, Peter Vaupel petervaupel med M, rernat Gabriele Multhoff tumde. Adenosine can thwart antitumor immune responses elicited by radiotherapy Therapeutic strategies alleviating protumor ADO activities. Strahlenther Onkol. 2016;192:279–87.

63. Wennerberg E, Spada S, Rudqvist NP, Lhuillier C, Gruber S, Chen Q, et al. CD73 blockade promotes dendritic cell infiltration of irradiated tumors and tumor

rejection. Cancer Immunol Res [Internet]. 2020; Available from: http://www.ncbi.nlm.nih.gov/pubmed/32047024

64. de Leve S, Wirsdörfer F, Cappuccini F, Schütze A, Meyer A v, Röck K, et al. Loss of CD73 prevents accumulation of alternatively activated macrophages and the formation of prefibrotic macrophage clusters in irradiated lungs. FASEB J [Internet]. 2017/03/21. 2017 Jul;31(7):2869–80. Available from: https://www.ncbi.nlm.nih.gov/pubmed/28325757

65. Wirsdorfer F, de Leve S, Cappuccini F, Eldh T, Meyer A v., Gau E, et al. Extracellular adenosine production by ecto-50-nucleotidase (CD73) enhances radiation-induced lung fibrosis. Cancer Res. 2016;76(10):3045–56.

66. Dewan MZ, Galloway AE, Kawashima N, Dewyngaert JK, Babb JS, Formenti SC, et al. Fractionated but not single-dose radiotherapy induces an immune-mediated abscopal effect when combined with anti-CTLA-4 antibody. Clinical Cancer Research. 2009;15(17):5379–88.

67. Charaghvandi RK, van Asselen B, Philippens MEP, Verkooijen HM, van Gils CH, van Diest PJ, et al. Redefining radiotherapy for early-stage breast cancer with single dose ablative treatment: a study protocol. BMC Cancer. 2017;17(1):181.

68. Hodi FS, Hwu WJ, Kefford R, Weber JS, Daud A, Hamid O, et al. Evaluation of Immune-Related Response Criteria and RECIST v1.1 in Patients With Advanced Melanoma Treated With Pembrolizumab. Journal of Clinical Oncology. 2016 May 1;34(13):1510–7.

69. Dovedi SJ, Illidge TM. The antitumor immune response generated by fractionated radiation therapy may be limited by tumor cell adaptive resistance and can be circumvented by PD-L1 blockade. OncoImmunology. 2015;4(7):1–4.

70. Dovedi SJ, Adlard AL, Lipowska-Bhalla G, McKenna C, Jones S, Cheadle EJ, et al. Acquired resistance to fractionated radiotherapy can be overcome by concurrent PD-L1 blockade. Cancer Research. 2014;74(19):5458–68.

71. Van Limbergen EJ, De Ruysscher DK, Pimentel VO, Marcus D, Berbee M, Hoeben A, et al. Combining radiotherapy with immunotherapy: The past, the present and the future. British Journal of Radiology. 2017.

72. Sundahl N, De Wolf K, Rottey S, Decaestecker K, De Maeseneer D, Meireson A, et al. A phase I/II trial of fixed-dose stereotactic body radiotherapy with sequential or concurrent pembrolizumab in metastatic urothelial carcinoma: Evaluation of safety and clinical and immunologic response. Journal of Translational Medicine. 2017;15(1):1–9.

73. Bartelink H, Maingon P, Poortmans P, Weltens C, Fourquet A, Jager J, et al. Whole-breast irradiation with or without a boost for patients treated with breast-conserving surgery for early breast cancer: 20-year follow-up of a randomised phase 3 trial. The Lancet Oncology. 2015 Jan;16(1):47–56.

74. Kaiser J, Kronberger C, Moder A, Kopp P, Wallner M, Reitsamer R, et al. Intraoperative Tumor Bed Boost With Electrons in Breast Cancer of Clinical Stages I Through III: Updated 10-Year Results. International journal of radiation oncology, biology, physics. 2018 May;

75. Fastner G, Sedlmayer F, Merz F, Deutschmann H, Reitsamer R, Menzel C, et al. IORT with electrons as boost strategy during breast conserving therapy in limited stage breast cancer: Long term results of an ISIORT pooled analysis. Radiotherapy and Oncology. 2013;108(2):279–86.

76. Leonardi MC, Dell'Acqua V, Cattani F, Morra A, Fodor C, Cambria R, et al. PO-0687: Technical feasibility of whole breast radiotherapy for local relapse after a previous partial breast irradiation. Vol. 106, Radiotherapy and Oncology. 2013. 263–264 p.

77. Brouwers PJAM, van Werkhoven E, Bartelink H, Fourquet A, Lemanski C, van Loon J, et al. Predictors for poor cosmetic outcome in patients with early stage breast cancer treated with breast conserving therapy: Results of the Young boost trial. Radiotherapy and Oncology. 2018;128(3):434–41.

78. Luke JJ, Lemons JM, Karrison TG, Pitroda SP, Melotek JM, Zha Y, et al. Safety and Clinical Activity of Pembrolizumab and Multisite Stereotactic Body Radiotherapy in Patients With Advanced Solid Tumors. Journal of Clinical Oncology [Internet]. 2018 Feb 13;JCO.2017.76.2229. Available from: https://doi.org/10.1200/JCO.2017.76.2229

79. Kroeze SGC, Fritz C, Hoyer M, Lo SS, Ricardi U, Sahgal A, et al. Toxicity of concurrent stereotactic radiotherapy and targeted therapy or immunotherapy: A systematic review. Cancer Treat Rev [Internet]. 2017;53:25–37. Available from: http://dx.doi.org/10.1016/j.ctrv.2016.11.013

80. Hwang WL, Pike LRG, Royce TJ, Mahal BA, Loeffler JS. Safety of combining radiotherapy with immune-checkpoint inhibition. Nat Rev Clin Oncol [Internet]. 2018;1. Available from: http://www.nature.com/articles/s41571-018-0046-7

81. Antonia SJ, Villegas A, Daniel D, Vicente D, Murakami S, Hui R, et al. Overall Survival with Durvalumab after Chemoradiotherapy in Stage III NSCLC. New England Journal of Medicine. 2018;NEJMoa1809697.

82. Leve S De, Wirsdörfer F, Jendrossek V. Targeting the Immunomodulatory CD73 / Adenosine System to Improve the Therapeutic Gain of Radiotherapy. 2019;10(April).

83. Wirsdörfer F, Leve S De, Jendrossek V. Combining Radiotherapy and Immunotherapy in Lung Cancer : Can We Expect Limitations Due to Altered Normal Tissue Toxicity ? 2019;1–21.

84. Perez-Aso M, Mediero A, Low YC, Levine J, Cronstein BN. Adenosine A2A receptor plays an important role in radiation-induced dermal injury. FASEB journal : official publication of the Federation of American Societies for Experimental Biology. 2016 Jan;30(1):457–65.

85. Wirsdorfer F, Jendrossek V. The Role of Lymphocytes in Radiotherapy-Induced Adverse Late Effects in the Lung. Frontiers in immunology. 2016;7:591.

86. Martinez-Marti A, Majem M, Barlesi F, Carcereny Costa E, Chu Q, Monnet I, et al. LBA42 - COAST: An open-label, randomised, phase II platform study of durvalumab alone or in combination with novel agents in patients with locally advanced, unresectable, stage III NSCLC. Annals of Oncology. 2021;32:S1320.

87. Debien V, Maurer C, Aftimos P, Clatot F, Loirat D, Punie K, et al. Abstract OT1-18-02: First-line chemo-immunotherapy with durvalumab, paclitaxel and carboplatin with or without anti-CD73 antibody oleclumab in advanced or metastatic triple-negative breast cancer: Preliminary results of the randomized phase II SYNERGY trial. Cancer Research. 2022 Feb 15;82(4_Supplement):OT1-18-02-OT1-18–02.

88. Litton JK, Regan MM, Pusztai L, Rugo HS, Tolaney SM, Garrett-Mayer E, et al. Standardized Definitions for Efficacy End Points in Neoadjuvant Breast Cancer

Clinical Trials: NeoSTEEP. Journal of Clinical Oncology. 2023 Sep 20;41(27):4433–42.

89. Cardoso MJ, Vrieling C, Cardoso JS, Oliveira HP, Williams NR, Dixon JM, et al. The value of 3D images in the aesthetic evaluation of breast cancer conservative treatment. Results from a prospective multicentric clinical trial. Breast. 2018 Oct 1;41:19–24.

90. Cardoso MJ, Cardoso J, Amaral N, Azevedo I, Barreau L, Bernardo M, et al. Turning subjective into objective: The BCCT.core software for evaluation of cosmetic results in breast cancer conservative treatment. The Breast. 2007 Oct 1;16(5):456–61.

91. Cardoso MJ, Cardoso JS, Oliveira HP, Gouveia P. The breast cancer conservative treatment. Cosmetic results - BCCT.core - Software for objective assessment of esthetic outcome in breast cancer conservative treatment: A narrative review. Comput Methods Programs Biomed. 2016 Apr 1;126:154–9.

92. Cui W, Rocconi RP, Thota R, Anderson RA, Bruinooge SS, Comstock IA, et al. Measuring ovarian toxicity in clinical trials: an American Society of Clinical Oncology research statement. Lancet Oncol [Internet]. 2023;24(10):e415–23. Available from: https://www.sciencedirect.com/science/article/pii/S147020452300390X

93. De Caluwe A, Romano E, Poortmans P, Gombos A, Agostinetto E, Marta GN, et al. First-in-human study of SBRT and adenosine pathway blockade to potentiate the benefit of immunochemotherapy in early-stage luminal B breast cancer: results of the safety run-in phase of the Neo-CheckRay trial. J Immunother Cancer [Internet]. 2023 Dec 6;11(12):e007279. Available from: https://jitc.bmj.com/lookup/doi/10.1136/jitc-2023-007279

94. Santanam L, Hurkmans C, Mutic S, Van Vliet-Vroegindeweij C, Brame S, Straube W, et al. Standardizing naming conventions in radiation oncology. International Journal of Radiation Oncology Biology Physics. 2012;83(4):1344–9.

95. Feng M, Moran JM, Koelling T, Chughtai A, Chan JL, Freedman L, et al. Development and validation of a heart atlas to study cardiac exposure to radiation following treatment for breast cancer. International Journal of Radiation Oncology Biology Physics. 2011;79(1):10–8.

96. Offersen B V., Boersma LJ, Kirkove C, Hol S, Aznar MC, Biete Sola A, et al. ESTRO consensus guideline on target volume delineation for elective radiation therapy of early stage breast cancer. Radiotherapy and Oncology. 2015;114(1):3–10.

97. Offersen B V., Boersma LJ, Kirkove C, Hol S, Aznar MC, Sola AB, et al. ESTRO consensus guideline on target volume delineation for elective radiation therapy of early stage breast cancer, version 1.1. Radiotherapy and Oncology. 2016;118(1):205–8.

98. ICRU. ICRU Report No. 91. Journal of the ICRU. 2017;14(2).

99. Harris EJ, Mukesh MB, Donovan EM, Kirby AM, Haviland JS, Jena R, et al. Amulticentre study of the evidence for customizedmargins in photon breast boost radiotherapy. British Journal of Radiology. 2016;89(1058).

100. van Hagen P, Hulshof MCCM, van Lanschot JJB, Steyerberg EW, Henegouwen MI van B, Wijnhoven BPL, et al. Preoperative Chemoradiotherapy for Esophageal or Junctional Cancer. New England Journal of Medicine. 2012 May;366(22):2074–84.

Docusign Envelope ID: 8E3E1A28-D1DF-4A9A-A65F-9548F273A2D6

101. Karasawa K, Katsui K, Seki K, Kohno M, Hanyu N, Nasu S, et al. Radiotherapy with concurrent docetaxel for advanced and recurrent breast cancer. Breast cancer (Tokyo, Japan). 2003;10(3):268–74.

102. J.R. B, K.L. L, G.K. E, J.R. G, R.B. L, M.M. AS. Concurrent radiation therapy and paclitaxel or docetaxel chemotherapy in high-risk breast cancer. International Journal of Radiation Oncology Biology Physics. 2000;48(2):393–7.

103. Ruocco E, Maio R Di, Caccavale S, Siano M, Schiavo A Lo. Radiation dermatitis , burns , and recall phenomena : Meaningful instances of immunocompromised district. Clinics in Dermatology. 2014;32(5):660–9.

104. Camidge DR, Kunkler IH. Docetaxel-induced radiation recall dermatitis and successful rechallenge without recurrence. Vol. 12, Clinical oncology (Royal College of Radiologists (Great Britain)). England; 2000. p. 272–3.

105. Kazandjieva J, Gergovska M, Darlenski R, Broshtilova V, Balabanova M, Stransky L. Pharmacology and therapeutics Recall dermatitis after systemic treatment with paclitaxel. 2010;2–5.

106. Puzanov I, Diab A, Abdallah K, Bingham CO, Brogdon C, Dadu R, et al. Managing toxicities associated with immune checkpoint inhibitors: consensus recommendations from the Society for Immunotherapy of Cancer (SITC) Toxicity Management Working Group. Journal for ImmunoTherapy of Cancer. 2017 Dec;5(1):95.

107. Institute of Medical Illustrators. IMI NATIONAL GUIDELINES : IMI National Guidelines A Guide to Good Practice Cultural Diversity. 2016;

Docusign Envelope ID: 8E3E1A28-D1DF-4A9A-A65F-9548F273A2D6

Appendix 1: Toxicity Management Guidelines

The current version of the document is provided by AstraZeneca and is available in a separate document.

Please note that these guidelines are not specific to the Neocheckray protocol and include information related to the management of Tremelimumab toxicity that is not applicable to the Neocheckray study.

Appendix 2 – Algorithm to select index lesion in case of multifocal or bilateral breast cancer

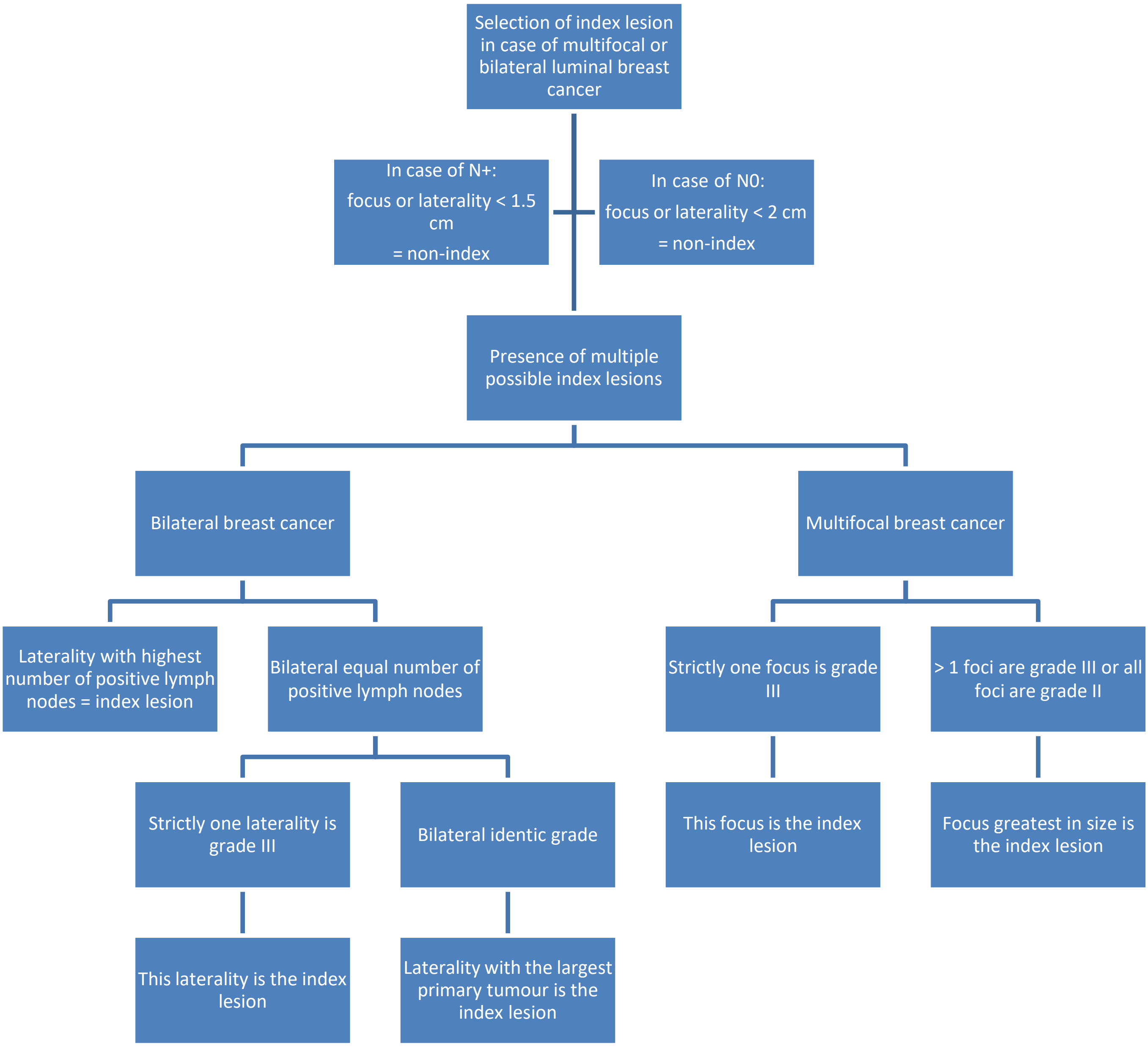

INSTITUT
JULES BORDET
INSTITUUT

Statistical Analysis Plan (SAP)

Neo-adjuvant chemotherapy combined with Stereotactic Body Radiotherapy to the primary tumour +/- durvalumab (MEDI4736), +/- oleclumab (MEDI9447) in luminal B breast cancer: a phase II randomised trial
Neo-CheckRay

Neo-CheckRay

EudraCT Number: 2018-004165-13
Sponsor Protocol Number: IJB-LBC-NEOCHECKRAY-2018
ClinicalTrials.gov Number: NCT03875573

Study Chair
Alex De Caluwé, MD – Institut Jules Bordet
Co-Study Chair
Emanuela Romano, MD PhD– Institut Curie

Sponsor: Institut Jules Bordet
Rue Meylemeersch 90
1070 Anderlecht Belgique/België

SAP version: 1.0 Issue date 03/07/2023
Created by: Paulus Kristanto

INSTITUT
JULES BORDET
INSTITUUT

Table of Contents

1 Introduction 4
2 Study objectives and endpoints 5
2.1 Study objectives 5
2.1.1 Primary objective 5
2.1.2 Secondary objective 5
2.2 Endpoints 5
2.2.1 Primary endpoints 5
2.2.2 Secondary endpoints 5
3 General analysis considerations 5
3.1 Timing of analyses 6
3.2 Analysis populations 6
4 Summary of baseline data 9
5 Paclitaxel, doxorubicin, cyclosphosphamide, durvalumab, and oleclumab administration 10
6 SBRT administration 12
7 Surgery 12
8 Efficacy analysis 13
8.1 Futility interim analysis 13
8.2 Final efficacy analysis 13
9 Safety and toxicity assessment 14
10 Cosmetics 16
11 Biomarkers 16
12 Other exploratory analyses 17
13 Notes 17

Abbreviations and definitions

AE	Adverse Event
BMI	Body Mass Index
CRF	Case Report Form
ddAC	dose-dense doxorubicin-cyclophosphamide
DFS	Disease Free Survival
ECOG	Eastern Cooperative Oncology Group
ER	Estrogen Receptor
GCP	Good Clinical Practice
G-CSF	Granulocyte colony-stimulating factor
iDFS	Invasive Disease Free Survival
IDMC	Interim Data Monitoring Committee
IMP	Investigational Medicinal Product
ITT	Intent to Treat
MedDRA	Medical Dictionary for Regulatory Activities
NCI-CTCAE	National Cancer Institute - Common Terminology of Cancer Adverse Events
pCR	pathologic complete response
PD-L1	Programmed death-ligand 1
PR	Progesterone Receptor
RCB	Residual Cancer Burden
SAP	Statistical Analysis Plan
SOC	System Organ Class

1 INTRODUCTION

This Statistical Analysis Plan is intended to clarify the Statistical Analysis Plan (SAP) written in the protocol of Neo-CheckRay and applies to the phase II of the study (the period after the safety run-in).

NEO-CHECKRAY is a multicenter, open-label phase II study that randomises luminal B breast cancer subjects candidate for neo-adjuvant chemotherapy in a 1:1:1 ratio in 3 arms:

1. Arm1: the combination of weekly paclitaxel followed by dose-dense doxorubicin-cyclophosphamide (ddAC) and pre-operative radiation therapy (boost dose) on the primary tumour
2. Arm 2: arm 1 with the addition of the anti-PD-L1 antibody durvalumab
3. Arm 3: arm 2 with the addition of the anti-CD73 antibody oleclumab

The primary tumour will be excised 2-6 weeks after completion of ddAC. A safety run-in had been done for the 6 first subjects before starting the randomised phase II trial.

The randomization process is based on 4 stratification factors:

- PD-L1 status determined centrally: low versus high.
- Primary tumour size: cT1/cT2 versus cT3
- Nodal status: cN0 versus cN+
- Participating sites

Figure 1.1 Study design

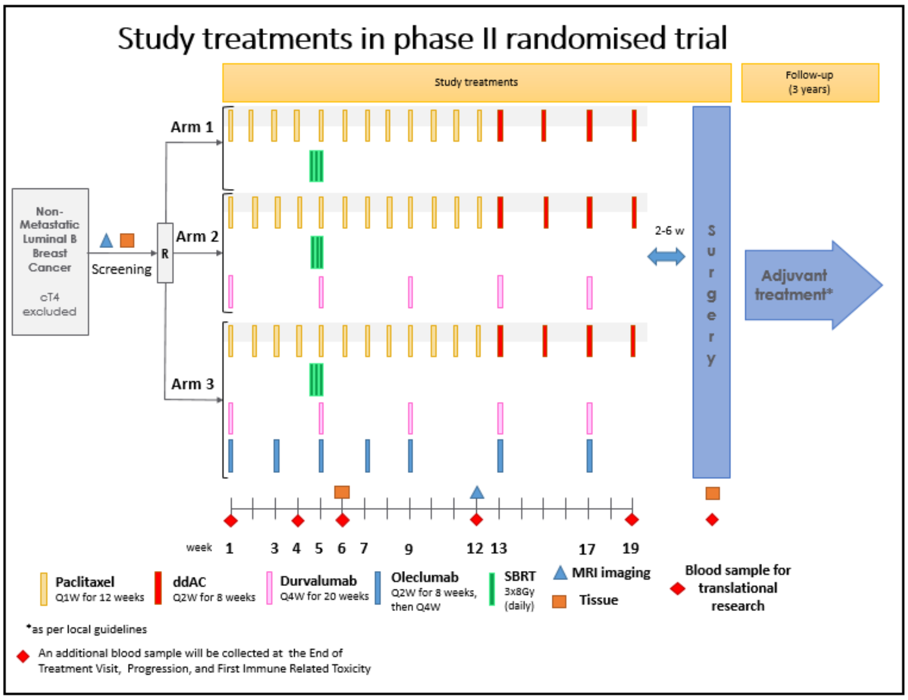

2 Study objectives and endpoints

2.1 Study objectives

2.1.1 Primary objective

To demonstrate improved tumour response of the primary tumour and nodal metastases in arms 2 or 3 versus arm 1.

2.1.2 Secondary objectives

- To evaluate the response to the primary tumour irrespective of the response to the pathological lymph nodes.
- To evaluate the response to the pathological lymph nodes irrespective of the response to the primary tumour.
- To evaluate the feasibility to perform breast-sparing surgery of the arms 2 and 3 versus arm 1.
- To evaluate the ability to control invasive disease in arms 2 and 3 versus arm 1 during three years after surgery.
- To evaluate the severity and duration of AEs of the arms 2 and 3 versus arm 1.
- To evaluate the cosmetic changes to the breast of the arms 2 and 3 versus arm 1.

2.2 Endpoints

2.2.1 Primary endpoint

- Residual cancer burden (RCB 0-1 vs. RCB 2-3) at time of surgery. RCB 0 is defined as pathological complete response (pCR) and RCB 1 is defined as minimal residual disease. RCB is calculated as a continuous index combining pathologic measurements of the primary tumour (size and cellularity) and nodal metastases (number and size) as defined by Symmans et al.

2.2.2 Secondary endpoints

- Pathological complete response rate (pCR), defined as pathological stage ypT0/Tis ypN0 at the time of definitive surgery. pCR (ypT0/Tis ypN0) is the absence of residual invasive cancer. Residual in situ carcinoma is accepted.

Important note: this secondary endpoint is an answer to the primary objective of the trial (see section 2.1.2 of the protocol), expressed in a different way than RCB 0/1. Recent phase III trials investigating the use of neo-adjuvant immunotherapy in ER+ breast cancer used ypT0/Tis ypN0 as primary endpoint instead of RCB 0/1 (see end of section 1.2.1.1 of the protocol). Therefore pCR (ypT0/Tis ypN0) is the most important secondary endpoint in this study.

• Complete pathologic response rate (pCR) of the primary tumour (ypT0/Tis), irrespective of the response rate of the resected nodal metastases.

• Complete pathologic response rate (pCR) of the resected nodal metastases (ypN0), irrespective of the response rate of the primary tumour.% of breast conservation surgery in arms 2 and 3 versus arm 1.

• Change in TIL levels between baseline and the week 6 biopsy.

3 GENERAL ANALYSIS CONSIDERATIONS

3.1 TIMING OF ANALYSES

The futility of arm 2 and 3 and third safety analysis will be performed at 50% information rate of the phase II randomized trial, corresponding to the time point in which 22 subjects underwent surgery in each arm and will compare the primary endpoint (arm 1 versus arm 2 and arm 1 versus arm 3). The analysis will consist of two parts: a futility analysis both for arms 2 and 3 and a third safety analysis. All these analyses will be presented to the IDMC.

Any results for publications will be performed once the primary endpoints for all evaluable subjects at the data cutoffs have been validated.

The final statistical analysis will be performed once the database is fully validated and locked.

3.2 ANALYSIS POPULATIONS

The main analysis populations are:

- Randomized population: all enrolled and randomized subjects.
- Eligible population: all randomized subjects, who are considered eligible. Any protocol deviations on eligibility should be reported. Despite of any protocol deviation, a randomized subject may be considered eligible by the investigators/medical review team and therefore should be included in the analysis population. In this case, any recorded eligibility justification from a protocol deviation should be reported. Hence, randomized and eligible populations will be different only when there is a subject that is considered not eligible post-randomization.
- Eligible subject will be considered to be evaluable in interim futility analysis or final primary analysis if all the criteria below are met:
    1) Subject received at least the first dose assigned treatment (dose reduction is permitted).
    2) Subject underwent surgery and a RCB score was obtained.
    3) Subject must be MammaPrint high risk or MammaPrint unevaluable.
- A subject that was included in the trial with a violation of an inclusion or exclusion criterium and discontinued the trial within 6 weeks after inclusion will be considered unevaluable for the interim analysis and this subject will be replaced.
- Interim analysis would be carried out after 22 evaluable subjects evaluated in each arm. The futility analysis will be performed to only the first 22 evaluable subjects in each arm, even if there are more evaluable subjects at the data cutoff. However, the safety and the toxicity

analysis will include all subjects who received at least the first dose assigned treatment at the data cutoff.

- The final primary analysis will be carried out in two populations:
    1) Intention-to-treat population, defined in the table below
    2) Per-protocol population, defined in the table below
- Safety population: all eligible subjects who received at least the first dose assigned treatment (dose reduction is permitted). In the interim analysis, the safety population is not limited to the number of evaluable subjects needed for the futility analysis.
- The analysis populations for the interim analysis are defined according to the following table:

	**Eligible Population (ITT)**	**Interim futility analysis**	**Safety and toxicity analysis**
Did not take the first dose assigned treatment	Include	Exclude	Exclude
Did not have any surgery or a RCB score was not obtained	Include	Exclude	Include
MammaPrint low risk	Include	Exclude	Include
MammaPrint unevaluable risk	Include	Include	Include
Subject that was included in the trial with a violation of an inclusion or exclusion criterium **and** discontinued the trial within 6 weeks after inclusion.	Include	Exclude	Include

INSTITUT
JULES BORDET
INSTITUUT

- The analysis populations for the final analysis are defined according to the following table:

	**Modified intention-to-treat (mITT) – primary analysis (RCB), ypTNM, breast conservation, biomarkers, cosmetics**	**Per-protocol – primary analysis (RCB), ypTNM, breast conservation, cosmetics, biomarkers**	**mITT – Disease Free Survival (DFS) and Invasive Disease Free Survival (IDFS) analysis**	**Per-protocol – DFS and IDFS analysis**	**Safety and toxicity analysis**
Did not take the first dose assigned treatment	Exclude	Exclude	Exclude	Exclude	Exclude
Did not have any surgery or a RCB score was not obtained	Exclude	Exclude	Include	Exclude	Include
MammaPrint low risk	Exclude	Exclude	Exclude	Exclude	Include
MammaPrint unevaluable risk	Include	Exclude	Include	Exclude	Include
Subject that was included in the trial with a violation of an inclusion or exclusion criterium **and** discontinued the trial within 6 weeks after inclusion.	Include	Exclude	Include	Exclude	Include

- In the case of MammaPrint unevaluable, MammaPrint reevaluation will be made before the analysis. In some cases, MammaPrint will remain unevaluable after reevaluation. The probability that these subjects are low risk is estimated to be low (below 15%). Therefore,

these subjects are included in the interim futility analysis to avoid any delay without any valid reason and to avoid imbalances in the interim analysis.

- Study enrollment will continue until the results of the futility analysis have been discussed by the IDMC. If early closure occurs on any cohort and accrual has gone beyond the number of subjects needed for the interim evaluation then all enrolled subjects who satisfy the evaluability criteria defined above should be included in the population for final analysis.

For each population analysis (randomized, eligible, evaluable (interim, ITT final, per-protocol final, safety), this information should be reported:

- The number (N) of subjects in each analysis population
- The date of randomization of the first subject and the last subject for each group (duration of subject enrollment)
- The number subjects by clinical site.
- Summary of time from randomization to data cutoff/database lock date (weeks)
- Summary of time from surgery to data cutoff/database lock date (weeks)
- A list of subjects with information on:
    - subject eligibility
    - site
    - date of randomization
    - indicator if at least one dose of first dose assigned treatment has been received
    - indicator if the subject has undergone surgery
    - date of surgery
    - MammaPrint status
    - RCB evaluable status
    - indicator of evaluability for the population analyzed.

4 SUMMARY OF BASELINE DATA

The summary of baseline data will be done for each arm and total for the eligible, evaluable (interim, or ITT and per-protocol for final analysis), and for safety populations.

Categorical variables should be summarized by the counts (frequency) and percent to total in each category. Continuous variables should be summarized by the number of available data (N), mean and standard deviation, minimum and maximum values (range), Median and IQR. Missing data should be reported as a category but not counted in the descriptive statistical analysis. Missing data should not be imputed.

Subjects' baseline information included in the report:

1. Demography:
    i. Age
    ii. Weight
    iii. BMI
    iv. ECOG Performance status
    v. Menopausal status
2. Cancer characteristics:
    i. Laterality: left, right, bilateral
    ii. cTNM:
        1. Primary tumour size: cT1/cT2 versus cT3
        2. Nodal status: cN0 versus cN+

   iii. Histological Grade
   iv. Invasive histological type
   v. Largest diameter of the index lesion (mm)
   vi. Multifocal yes/no
   vii. Breast tumor location
   viii. Lymph node tumor location
3. Biomarkers:
   i. PD-L1 status determined centrally: data from Biotracker
   ii. MammaPrint result: for bilateral, data from index lesion
   iii. MammaPrint numeric score – data will come Agendia (we will need a mapping from Kit-ID to ssid).
   iv. BluePrint result – data will come Agendia (we will need a mapping from Kit-ID to ssid).
   v. Ki67 %
   vi. ER
   vii. PR score
   viii. HER2 score
4. Tumour evaluation at screening:

   Tumor size measured by MRI? Yes/No, if No: tumor size measured by ultrasound? Yes/No

5. Medical History: Concomitant diseases or past surgeries Yes/No

5 PACLITAXEL, DOXORUBICIN, CYCLOSPHOSPHAMIDE, DURVALUMAB, AND OLECLUMAB ADMINISTRATION

The summary of all treatments received will be done for each arm and total for the eligible, evaluable (interim, or ITT and per-protocol for final analysis), and for safety populations.

Categorical variables should be summarized by the counts (frequency) and percent to total in each category. Missing data should be reported as a category. Continuous variables should be summarized by the number of available data (N), mean and standard deviation, minimum and maximum values (range), Median and IQR. Missing data should not be imputed.

For each treatment (paclitaxel, doxorubicin, cyclosphosphamide, durvalumab, oleclumab), the information below will be summarized:

- At least one dose: yes, no
- Treatment duration (in days)
- Number of cycles received
- Dose administered per cycle
- Dose adjustment: yes, no. Reasons of dose adjustment: Administrative reasons, Adverse event, Subject non-compliance with study drug, Technical problem, Other (specify)
- Dose delay: yes, no. Reasons of dose delay: Administrative reasons, Adverse event, Subject non-compliance with study drug, Technical problem, Other (specify)
- Dose interruption: yes, no. Reasons of treatment interruption: Administrative reasons, Adverse event, Subject non-compliance with study drug, Technical problem, Other (specify)

- N subjects with dose adjustment or treatment interruption
- Relative dose intensity

Treatment	Formula used	Prescribed dose
Paclitaxel	Total actual paclitaxel dose taken/(80*Body surface *12)*100% Body surface is calculated as the average body surface measurements during the study	Paclitaxel is given at a dose of 80 mg/m² IV once a week for 12 weeks.
Doxorubicin	Total actual doxorubicin dose taken /(60* Body surface *4)*100%	Dose-dense doxorubicin is given at a dose of 60 mg/m2 IV every 2 weeks for 4 doses starting one week after the end of paclitaxel
Cyclophosphamide	Total actual cyclophosphamide dose taken /(600* Body surface *4)*100%	Cyclophosphamide is given at a dose of 600 mg/m2 IV every 2 weeks for 4 doses starting one week after the end of paclitaxel.
Durvalumab	Total actual durvalumab dose taken /(1500*5)*100%	Durvalumab will be given at the fixed dose of 1500 mg IV every 4 weeks (Q4W)
Oleclumab	Total actual oleclumab dose taken /(3000*7)*100%	Oleclumab 3000 mg will be given IV every 2 weeks (Q2W) for the first 5 administrations then every 4 weeks (Q4W) for the last 2 administrations

- Treatment discontinuation: yes, no.
- Reasons of treatment discontinuation: Adverse event, Completed, Death, Lost to follow-up, Non-compliance with study drug, Physician decision, Pregnancy, Progressive disease, Protocol deviation, Site terminated by sponsor, Study terminated by sponsor, Subject decides to withdraw, Technical problems, Other (specify)
- When a subject does not receive all cycles, the site might not note “complete” in the treatment completion page. Instead the site might check “Other” and specify as “…, subject did not receive all cycles”. We will not treat this subject as a case of early discontinuation, but rather as a treatment interruption or a dose delay.

6 SBRT ADMINISTRATION

The summary of all treatments received will be done for each arm and total for the eligible, evaluable (interim, or ITT and per-protocol for final analysis), and for safety populations.

Categorical variables should be summarized by the counts (frequency) and percent to total in each category. Missing data should be reported as a category. Continuous variables should be summarized by the number of available data (N), mean and standard deviation, minimum and maximum values (range), Median and IQR. Missing data should not be imputed.

The information below will be summarized:

- At least one dose: yes, no
- Number of fractions delivered
- Dose delivered to 95%of the PTV volume
- Dose delay: yes, no. Reasons of dose delay: Administrative reasons, Adverse event, Subject non-compliance with study drug, Technical problem, Other (specify)
- Dose interruption: yes, no. Reasons of treatment interruption: Administrative reasons, Adverse event, Subject non-compliance with study drug, Technical problem, Other (specify)

7 SURGERY

The summary of surgery treatment will be done for each arm and total for the eligible, evaluable (interim, or ITT and per-protocol for final analysis), and for safety populations.

Categorical variables should be summarized by the counts (frequency) and percent to total in each category. Missing data should be reported as a category. Continuous variables should be summarized by the number of available data (N), mean and standard deviation, minimum and maximum values (range), Median and IQR. Missing data should not be imputed.

The information below should be reported:

- ypTNM at Surgery : The study chair and the investigators will be blinded of the ypTNM data in the Interim Analysis.
- Sentinel lymph node biopsy performed, Yes/No
- Number of positive sentinel lymph nodes. Blinded for Study Chair and investigators in the Interim Analysis.
- Axillary node dissection performed, Yes/No :
- Number of positive axillary lymph nodes. Blinded for Study Chair and investigators.
- Size of largest positive lymph node (mm). Blinded for Study Chair and investigators in the Interim Analysis.
- Breast surgery performed, Yes/No.
- Type of surgery performed

8 EFFICACY ANALYSIS

The evaluability criteria for the efficacy interim and final analysis is defined in Section 3.2.

Categorical variables should be summarized by the counts (frequency) and percent to total in each category. Missing data should be reported as a category. Continuous variables should be summarized by the number of available data (N), mean and standard deviation, minimum and maximum values (range), Median and IQR. Missing data should not be imputed.

8.1 FUTILITY INTERIM ANALYSIS

- Interim analysis is intended to evaluate futility of arm 2 and arm 3
- The primary endpoint is the residual cancer burden (**RCB 0-1 vs. RCB 2-3**) at time of surgery. RCB 0 is defined as pathological complete response (pCR) and RCB 1 is defined as minimal residual disease. Comparison for futility for both comparisons (arm 1 versus arm 2 and arm 1 versus arm 3) will be carried out at an information fraction of 1/2. We keep the goal to be able to detect an increase from 15% to 45% with a power of 80% and a 2-sided alpha level of 2.5% (due to multiplicity with the 2 planned comparisons). The futility boundaries would be calculated using Lan-Demets bêta spending functions and non-binding boundaries once 1/2 of evaluable subjects will have been evaluated (availability of RCB).
- Stopping boundaries for early closure for both of the experimental arms (arm 2 and/or arm 3) would be +/- 0.533 on the z scale.
- Residual cancer burden (**RCB 0 vs. RCB 1-2-3**) at time of surgery.
- The RCB continuous score will be summarized by arms and all subjects. The RCB continuous score will also be summarized by clinical sites.
- The yT and yN at Surgery will be reported for each RCB categorical score to verify the consistency of the scoring.
- The whole analyses would be submitted to an IDMC for advice about the continuation of the study with 3 arms or 2 arms or terminate the trial entirely. The study chairs and the investigators will be blinded of the futility interim analysis.

8.2 FINAL EFFICACY ANALYSIS

- The primary endpoint is the residual cancer burden (**RCB 0-1 vs. RCB 2-3**) at time of surgery. RCB 0 is defined as pathological complete response (pCR) and RCB 1 is defined as minimal residual disease. The primary analyses will compare arm 1 and arm 2 as well as arm 1 and arm 3. The observed proportions will be compared by a chi square test without continuity correction at an alpha two-sided level of 2.5%. Confidence intervals for the difference

between proportions will be provided at the usual 95% level. As exploratory analysis, a confidence interval for the difference between proportions in arm 2 and arm 3 will be provided.

- The RCB continuous score will be summarized by arms and all subjects. The RCB continuous score will also be summarized by clinical sites.
- Residual cancer burden (**RCB 0 vs. RCB 1-2-3**) at time of surgery.
- The yT and yN at Surgery will be reported for each RCB categorical score to verify the consistency of the scoring.
- Rates of subjects with breast conservation surgery in arms 2 and 3 versus arm 1 will be compared using chi square tests.

- Comparison between between arm 1 and arm 3 as well as arm 2 and arm 3 of complete pathologic response rate (pCR) of the primary tumour (ypT0), irrespective of the response rate of the resected nodal metastases using chi-square tests.

- Comparison between between arm 1 and arm 3 as well as arm 2 and arm 3 of complete pathologic response rate (pCR) of the resected nodal metastases (ypN0), irrespective of the response rate of the primary tumour using chi-square tests.

- Invasive Disease Free Survival (IDFS) and Disease Free Survival (DFS) will be measured using regular follow-up investigations: lab work, clinical examination and annual breast ultrasound and mammography. Radiologic imaging will not be routinely performed, unless directed by abnormal blood results or clinical examination. IDFS or DFS is defined at the time from randomization to the disease progression event. In case of no event, the time to event variable will be censored at the last visit recorded. The disease progression is considered invasive when the location of the disease progression is not 'in situ' or occurs outside the breast. The findings should be based on biopsy. The survival curves will be estimated using Kaplan-Meier method of disease-free survival and of invasive disease-free survival will be compared between arm 1 and arm 3 as well as arm 2 and arm 3 using log rank. This analysis will be conducted on ITT and per-protocol population (See Section 3.2 for the criteria).
- Other exploratory analysis will be defined post-hoc.

9 SAFETY AND TOXICITY ASSESSMENT

The analysis population for safety and toxicity assessment is described in Section 3.2. The analysis will include

The intensity of all AEs has been graded according to the NCI-CTCAE version 4.03 on a five-point scale (Grade 1 to 5). The analysis should be based on the classification of adverse events of MedDRA Preferred Term (PT). This classification is based on the reported free-text AE reported in the CRF.

Further classification will be performed for reporting:

- Neutropenia: Neutrophil Count Decrease (SOC name should be grouped under Blood and lymphatic system disorders): will be grouped together with Neutropenia
- Lymphopenia: Lymphocyte Count Decrease(SOC name should be grouped under Blood and lymphatic system disorders): will be grouped together with Lymphopenia
- Leukopenia: Leucocyte Count Decrease (SOC name should be grouped under Blood and lymphatic system disorders) will be grouped together with Leukopenia
- Thrombocytopenia: Platelet Count Decrease (SOC name should be grouped under Blood and lymphatic system disorders)will be grouped together with Thrombocytopenia
- Rash: All kind of rash indications will be grouped together (Rash, Rash erythematous, Rash pustular, Rash maculo-papula)

The count in the analysis should be based on the number of subjects (not the number of episodes). The AE episodes should be summarized for each subject. The maximum grade of the episodes of the same adverse event occurred to a subject should be used to characterize the grade of that particular adverse event occurred to that subject. If more than one episodes of the same adverse event occurred to a subject and at least one episode is classified as SAE or AESI then this subject should be considered to have SAE or AESI for that particular adverse event. If more than one episodes of the same adverse event occurred to a subject and at least one episode is attributed to certain treatment then this subject should be considered to have that particular adverse event due to this treatment.

Information needed to be reported for each AE:

- Number of subjects (N (%))
- Max grade
- Number of subjects (N (%)) for each grade (or by grade 1 and 2 combined and grade 3 and 4 combined)

Analyses will be presented:

- All AEs
- All Serious Adverse Events (SAEs): A serious adverse event (SAE) or reaction (SAR) is any untoward medical occurrence that results in any of the following outcomes: death, life-threatening, in-patient hospitalisation or prolongation of existing hospitalization, persistent or significant disability/incapacity, or congenital anomaly/birth defect.
- All Adverse Events of Special Interests (AESIs): The description of the AESIs can be found in the Protocol. Based on this definition, the investigators made an assessment whether an adverse event should be considered as AESI or not.
- All AEs of Grade 4
- All AEs of Grade 3
- All AEs related to any treatment: Treatment related adverse events are adverse events related to any treatment (paclitaxel, doxorubicin, cyclophosphamide, durvalumab, oleclumab, SBRT, or surgery) in this study. In some cases, there were more than one episodes of the same adverse events occurred to a patient. Therefore, when at least one of them was

considered by the investigator to be related to a certain treatment then the patient is considered having a treatment related adverse event of this kind.

- All AEs related to durvalumab
- All AEs related to oleclumab
- All AEs related to SBRT
- All AEs related to surgery
- All AEs related to paclitaxel
- All AEs related to doxorubicin
- All AEs related to cyclophosphamide
- All specific Adverse Events of interest (predefined). A set of specific AEs will be reported. The indication will not depend on the investigators' assessments but will be based on pre-determined AE list to be reported.
- All deaths
- Early discontinuations due to AE

The AE list should be presented by the descending order of the number of subjects and then by maximum grade observed. For the list of all AEs and all AEs related to any treatment, the AEs should also be presented by medDRA System Organ Classification (SOC) categories.

For patients who had neutropenia related to doxorubicin or cyclosphosphamide treatment, granulocyte colony-stimulating factor (G-CSF) medication will be reported. G-CSF treatment is reported as concomitant medication and the pre-post medications as a free-text. The mapping of this information into G-CSF usage will be reviewed before analysis.

10 Cosmetics

Cosmetics evaluation will be performed in the final analysis.

- Changes in breast appearance: breast fibrosis in whole breast, breast fibrosis in boost area, breast size, breast shape, nipple position, shape of the areola and nipple, skin color, appearance of surgical scar, evaluation of teleangiectasia and global cosmetic result. Digitalized color photographs will be taken at multiple time points and the cosmetic changes on the color photographs will be scored quantitatively by the BCCT.core software program. The score comparison in arms 2 and 3 versus arm 1 will be done using non-parametric Wilcoxon test.
- Other cosmetics analysis will be defined post-hoc.

11 Biomarkers

Analysis of biomarkers will be performed in the final analysis.

- Rates of subjects who have a two fold increase in TIL levels in subjects with a baseline biopsy and operated and with TILs available using chi-square tests. ;

- Other analysis based on the biomarkers will be defined post-hoc.

12 Other exploratory analyses

Further exploratory analysis will be performed in the final analysis.

- Exploratory analyses will be done to try to identify predictive factors of achievement of RCB0-1. The covariates that will be tested will include PD-L1 status, TILs, TNM status. Modelling will be done using logistic regression models.
- Changes in prevalence of immune cell subpopulations will be descriptively analysed.
- Further exploratory data analysis will be decided post-hoc.

13 Notes

This SAP can be updated based on any protocol amendments. It can also be modified to further clarify the analysis needed.
