# Supplementary material for: Neoadjuvant stereotactic body radiation therapy with durvalumab and oleclumab in ER+HER2− breast cancer: a randomized phase 2 trial
Source: Nat Med. 2026 Jun 25;32(7):2461–72. doi: 10.1038/s41591-026-04453-z (PMC13375581; doi:10.1038/s41591-026-04453-z)
Supplement: Supplementary file 2 — Reporting Summary [file 41591_2026_4453_MOESM2_ESM.pdf]

nature portfolio

Corresponding author(s): Alex De Caluwé

Last updated by author(s): Jan 31, 2026

Reporting Summary

Nature Portfolio wishes to improve the reproducibility of the work that we publish. This form provides structure for consistency and transparency in reporting. For further information on Nature Portfolio policies, see our Editorial Policies and the Editorial Policy Checklist.

Statistics

For all statistical analyses, confirm that the following items are present in the figure legend, table legend, main text, or Methods section.

n/a	Confirmed	
☐	☒	The exact sample size (*n*) for each experimental group/condition, given as a discrete number and unit of measurement
☒	☐	A statement on whether measurements were taken from distinct samples or whether the same sample was measured repeatedly
☐	☒	The statistical test(s) used AND whether they are one- or two-sided<br>*Only common tests should be described solely by name; describe more complex techniques in the Methods section.*
☐	☒	A description of all covariates tested
☐	☒	A description of any assumptions or corrections, such as tests of normality and adjustment for multiple comparisons
☐	☒	A full description of the statistical parameters including central tendency (e.g. means) or other basic estimates (e.g. regression coefficient) AND variation (e.g. standard deviation) or associated estimates of uncertainty (e.g. confidence intervals)
☐	☒	For null hypothesis testing, the test statistic (e.g. *F*, *t*, *r*) with confidence intervals, effect sizes, degrees of freedom and *P* value noted<br>*Give P values as exact values whenever suitable.*
☒	☐	For Bayesian analysis, information on the choice of priors and Markov chain Monte Carlo settings
☒	☐	For hierarchical and complex designs, identification of the appropriate level for tests and full reporting of outcomes
☒	☐	Estimates of effect sizes (e.g. Cohen's *d*, Pearson's *r*), indicating how they were calculated

Our web collection on statistics for biologists contains articles on many of the points above.

Software and code

Policy information about availability of computer code

Data collection	Clinical data were captured in the study clinical database (electronic case record form - eCRF).<br>Translational data were captured in the translational database.
Data analysis	R v4.2.2 (R Foundation for Statistical Computing). R code was made available in a community repository (FigShare).<br>R packages: tximport — v1.38.2; biomaRt — v2.66; DESeq2 — v1.50.2; GSVA — v2.4.4; ComplexHeatmap — v2.26<br>R code available from https://doi.org/10.6084/m9.figshare.29489597<br><br>Sequencing / preprocessing software used: Trimmomatic — v0.39; STAR — v2.7.11; Salmon — v1.10.0

For manuscripts utilizing custom algorithms or software that are central to the research but not yet described in published literature, software must be made available to editors and reviewers. We strongly encourage code deposition in a community repository (e.g. GitHub). See the Nature Portfolio guidelines for submitting code & software for further information.

Data

Policy information about availability of data

All manuscripts must include a data availability statement. This statement should provide the following information, where applicable:

- Accession codes, unique identifiers, or web links for publicly available datasets
- A description of any restrictions on data availability
- For clinical datasets or third party data, please ensure that the statement adheres to our policy

Source data used for the analyses are deposited with this paper in FigShare and available at https://doi.org/10.6084/m9.figshare.29489597 (27). The source data provided with this manuscript contain all data required to reproduce the analyses and figures presented. The clinical trial protocol and statistical analysis plan are provided as Supplementary Information with this manuscript. Raw sequencing data have been deposited in the European Genome-phenome Archive (EGA) under accession number EGAD50000002552. Additional de-identified individual participant-level data generated in the Neo-CheckRay study, beyond the source data files provided with this manuscript, have been deposited at the Data Centre of Institut Jules Bordet. Institut Jules Bordet will honor requests for clinical trial data from qualified researchers with a clearly defined scientific objective. Sharing is also subject to the protection of patient privacy, compliance with applicable regulatory, ethical and General Data Protection Regulation (GDPR) requirements, and respect for patients' informed consent. Data considered for sharing may include nonidentifiable patient-level and study-level clinical trial data, full clinical study reports and protocols. The expected timeframe for response to access requests is up to 6 months. Once access has been granted, the data will be available for 12 months and may be extended upon approval. No third-party data were used in this study.

Research involving human participants, their data, or biological material

Policy information about studies with human participants or human data. See also policy information about sex, gender (identity/presentation), and sexual orientation and race, ethnicity and racism.

Reporting on sex and gender	The manuscript reports on patients with breast cancer. All patients were biological females. Female sex was determined based on self-report.
Reporting on race, ethnicity, or other socially relevant groupings	We do not provide reporting on race, ethnicity, or other social groupings.
Population characteristics	This information is provided in Table 1.
Recruitment	Participants were recruited consecutively at seven participating clinical centres in Belgium and France in accordance with Good Clinical Practice (GCP) standards. Participating sites in Belgium: Institut Jules Bordet, UZ Leuven, GZA Antwerpen, ChU Namur, Hôpitaux Universitaires St Luc. France: Institut Curie, Paris; CGFL Dijon. Eligible patients were identified by the local investigators during routine clinical care and were informed about the study. Those willing to participate provided informed consent prior to inclusion.<br>As participation required consent, a degree of self-selection bias cannot be excluded. Patients who agreed to participate may have been more motivated to participate to clinical research, more engaged in their healthcare, or differed in socio-demographic characteristics from those who declined participation. In addition, recruitment from participating centres may introduce centre-related selection bias, as these sites may not be fully representative of all healthcare settings in Belgium and France.<br>To reduce selection bias, consecutive inclusion of all eligible patients was encouraged across all centres using harmonised eligibility criteria and standardised study procedures. Given the multicentre design involving seven sites across two countries, the risk of single-centre bias is reduced, although some limitations in generalisability may remain.
Ethics oversight	The trial protocol was approved by the ethics committee in Belgium and in France. For Belgium: Commissie Medische Ethiek UZ Brussels/VUB, Laarbeeklaan 101, 1090 Brussels, Reference number: 2019/P/02. For France: CHU de Grenoble, Comité de Protection des personnes, CS 10217 38043, Grenoble Cedex 9, Reference number: 20-JUBO-01.<br>Patients provided written informed consent.<br>Participants were not financially compensated for study participation. The sponsor covered all study-specific costs, including protocol-mandated consultations, examinations and procedures not part of standard care, as well as the investigational immunotherapy (durvalumab plus oleclumab), which was provided free of charge. Participants therefore incurred no additional costs compared with standard treatment. Reasonable travel expenses were reimbursed upon presentation of supporting documents, up to a maximum of €75 for the entire study.<br>An external independent data monitoring committee oversaw the study; assessed safety at 3 different timepoints; and assessed efficacy at the prespecified interim analysis.

Note that full information on the approval of the study protocol must also be provided in the manuscript.

Field-specific reporting

Please select the one below that is the best fit for your research. If you are not sure, read the appropriate sections before making your selection.

☒ Life sciences ☐ Behavioural & social sciences ☐ Ecological, evolutionary & environmental sciences

For a reference copy of the document with all sections, see nature.com/documents/nr-reporting-summary-flat.pdf

Life sciences study design

All studies must disclose on these points even when the disclosure is negative.

Sample size	Primary endpoint: with a total of 132 evaluable patients, the trial had 80% power to detect a true difference (superiority) of 30 percentage points for the comparison of the rate of RCB 0/1 between No_ICI and Single_ICI, or No_ICI and Double_ICI at a two-sided α level of 0.025 for each comparison, assuming a 15% RCB 0/1 rate in No_ICI. Allowing for nonevaluable patients, the target sample size was increased to 147 randomized patients, with 184 planned for screening to account for screening failures.<br>Analyses were performed in either the entire intention-to-treat (ITT) population or the per-protocol population, as appropriate; the specific population used is indicated for each figure.
Data exclusions	Primary and secondary endpoints were assessed in the ITT population and the predefined per protocol population. The ITT population comprised all randomized patients, regardless of their MammaPrint status, regardless of whether the treatment was started or not, and regardless of treatment discontinuation or not. In case the result at surgery was unavailable, the patient was considered as a non-responder. The per protocol population comprised all MammaPrint High Risk patients with an available RCB score. All patients who initiated neoadjuvant treatment were included in the per protocol population, irrespective of whether study treatments were discontinued or not. Safety was assessed in the ITT population.
Replication	This was a clinical trial, for which replication is not appropriate.
Randomization	The randomisation was performed by an integrated Web-response system. The trial was stratified by centrally assessed PD-L1 status (<1% vs ≥1%), nodal status (cN0 vs cN+) and tumour size (cT1/2 vs cT3). Randomisation was performed using the minimization method. The allocation ratio was 1:1:1, with parallel group comparisons and no crossover allowed.
Blinding	The trial was open-label because it was designed an exploratory phase II trial using a novel combination treatment. The open-label design allowed for closer monitoring of immune-related adverse events .

Reporting for specific materials, systems and methods

We require information from authors about some types of materials, experimental systems and methods used in many studies. Here, indicate whether each material, system or method listed is relevant to your study. If you are not sure if a list item applies to your research, read the appropriate section before selecting a response.

Materials & experimental systems

n/a	Involved in the study	
☐	☒	Antibodies
☒	☐	Eukaryotic cell lines
☒	☐	Palaeontology and archaeology
☒	☐	Animals and other organisms
☐	☒	Clinical data
☒	☐	Dual use research of concern
☒	☐	Plants

Methods

n/a	Involved in the study	
☒	☐	ChIP-seq
☒	☐	Flow cytometry
☒	☐	MRI-based neuroimaging

Antibodies

Antibodies used	Antibodies used for immuno-histochemistry (IHC): PD-L1, Ki67, CD73 and MHC-I. Details on clone, isotype, company, catalog, dilution, antigen retrieval, incubation conditions, amplificiation and detection kit are detailed in Supplemental Figure 11 and is made available with the manuscript.
Validation	All antibodies used were commercially available and validated by the manufacturers for immunohistochemistry on formalin-fixed paraffin-embedded tissue. Validation information was provided by the manufacturers and is available on the respective product webpages and technical data sheets. PD-L1 (clone SP263, Ventana/Roche, cat#790-4905) and Ki67 (clone 30-9, Ventana/Roche, cat#790-4286) were validated according to manufacturer protocols for the VENTANA BenchMark platform. CD73 (clone D7F9A, Cell Signaling Technology, cat#13160S) and MHC-I (clone EMR8-5, Abcam, cat#ab70328) were validated by the manufacturers for IHC applications.

Clinical data

Policy information about clinical studies
All manuscripts should comply with the ICMJE guidelines for publication of clinical research and a completed CONSORT checklist must be included with all submissions.

Clinical trial registration	Clinical trials.gov identifier: NCT03875573
Study protocol	The clinical trial protocol of the Neo-CheckRay trial is provided as a Supplementary Information file accompanying the manuscript.

Data collection

Data were collected in seven participating centres in Belgium and France. Participating sites in Belgium: Institut Jules Bordet, UZ Leuven, GZA Antwerpen, ChU Namur, Hôpitaux Universitaires St Luc. France: Institut Curie, Paris; CGFL Dijon. Screening took place from 15 June 2021 to 11 March 2024. During this period, 200 patients were screened and 147 were randomized. All data were recorded in case report forms (CRFs) in accordance with Good Clinical Practice (GCP), with data management coordinated by the Clinical Trials Centre of Institut Jules Bordet, sponsor of the trial. The database lock for all analyses was 04 December 2025.

Outcomes

The study's primary endpoint was residual cancer burden (RCB) 0 or 1 at time of surgery. RCB 0 is equivalent to pathological complete response (pCR). RCB is calculated as a continuous index combining pathologic measurements of the primary tumour (size and cellularity) and lymph node metastases (number and size).
Secondary outcomes were prospectively pre-defined in the study protocol prior to study initiation. They were selected to reflect clinically relevant pathological, survival, safety, and cosmetic outcomes consistent with the neoadjuvant breast cancer trial setting. Secondary endpoints included pCR (defined as ypT0/Tis, ypN0), pCR limited to the primary tumour, and pCR limited to lymph nodes in case of baseline nodal positivity, and safety. Other endpoints will be reported at a longer follow-up time, such as EFS and cosmetic outcome at 3 years and 5 years (not reported in the present manuscript).
Biomarker analyses were pre-specified exploratory analyses included in the translational research section of the clinical trial protocol (protocol page 127; section 11.2).

Plants

Seed stocks

Report on the source of all seed stocks or other plant material used. If applicable, state the seed stock centre and catalogue number. If plant specimens were collected from the field, describe the collection location, date and sampling procedures.

Novel plant genotypes

Describe the methods by which all novel plant genotypes were produced. This includes those generated by transgenic approaches, gene editing, chemical/radiation-based mutagenesis and hybridization. For transgenic lines, describe the transformation method, the number of independent lines analyzed and the generation upon which experiments were performed. For gene-edited lines, describe the editor used, the endogenous sequence targeted for editing, the targeting guide RNA sequence (if applicable) and how the editor was applied.

Authentication

Describe any authentication procedures for each seed stock used or novel genotype generated. Describe any experiments used to assess the effect of a mutation and, where applicable, how potential secondary effects (e.g. second site T-DNA insertions, mosiacism, off-target gene editing) were examined.
