# Supplementary material for: Neoadjuvant stereotactic body radiation therapy with durvalumab and oleclumab in ER+HER2− breast cancer: a randomized phase 2 trial
Source: Nat Med. 2026 Jun 25;32(7):2461–72. doi: 10.1038/s41591-026-04453-z (PMC13375581; doi:10.1038/s41591-026-04453-z)
Supplement: Supplementary file 3 — Peer Review File [file 41591_2026_4453_MOESM3_ESM.pdf]

nature portfolio

Peer Review File

Neoadjuvant stereotactic body radiation therapy with durvalumab and oleclumab in ER+/HER2- breast cancer: a randomised phase 2 trial.

Corresponding Author: Dr Alex De Caluwé

This file contains all reviewer reports in order by version, followed by all author rebuttals in order by version.

Version 0:

Reviewer comments:

Reviewer #1

[redacted]

[redacted]

[redacted]

[redacted]

[redacted]

Reviewer #2

(Remarks to the Author)
This manuscript reports the response at surgery of the patients enrolled in the Neo-CheckRay trial, a phase 2, randomized trial testing NACT plus radiation (8GyX3, SBRT) with or without durvalumab (anti-PD-L1) and with or without oleclumab (anti-CD73) in 147 high-risk early ER+/HER2- breast cancer patients.

In two prior phase III studies performed in similar patient populations (refs 11 and 12) addition of anti-PD1 (pembrolizumab or nivolimumab) to NACT increased pCR rate from 15.6% to 24.3% and 13.8% to 24.5%, respectively. Therefore, the pCR rate of 16.7% observed here in the arm treated with SBRT and NACT without ICI seems to be in line with what could be expected in the absence of SBRT; in the arms receiving SBRT+anti-PD1 and SBRT+anti-PD1+anti-CD73 the pCR rate is higher (29.4 and 33.3%, respectively) but given the small number of patients in each arm this results do not provide evidence for a true benefit of SBRT addition to immunotherapy. Although in the pre-defined MammaPrint high population the difference between pCR in SBRT only versus SBRT+double_ICI achieves significance, this reflects the benefits of ICI. The strength of the study is in the analysis of responses based on baseline PDL1 status: the latter demonstrates a remarkable pCR rate of 28.1% (Single_ICI), and 30.3% (Double_ICI) in PDL-1 negative tumors, which in the two phase III trials showed variable but much lower pCR to NACT+anti-PD1.

Thus, although the study was not randomized to compare the outcome in the presence vs absence of SBRT, results support the concept that the benefits of radiation used with ICI can be discerned in “cold tumors” but not in unselected patient

populations. This interpretation is supported by a recent report in NSCLC (PMID: 40696153). It is also supported by the correlative studies performed on tumor biopsies showing an increase in PDL1 expression in the tumor 1week post-SBRT.

Overall, the study is very well described and provides critical new information of high relevance to BC treatment. Multiple exploratory analyses are performed, adding scientific depth. There are only a few issues that need to be addressed.

1) An intriguing result is the apparent loss of ICI efficacy in patients who received >1 Gy to the axilla level I lymph nodes. While multiple pre-clinical studies have demonstrated that radiation to draining nodes compromises tumor responses to ICI, axillary radiation was not performed in this study and the effects of the relatively low dose that was delivered to the nodes (likely due to the lateral location of the tumor) is concerning. Since all patients have at least sentinel node dissection it is important to analyze the lymph nodes for evidence of an effect of radiation or any changes (e.g., an increase in regulatory T cells?) that could explain the results.

2) As acknowledged by the authors, there is no clear evidence of a benefit of anti-CD73. It is possible that the latter is effective only in a subset of the patients and the cohort analyzed here is too small to identify such signal. The only correlative study performed is tissue staining for CD73 expression. Given that "adenosine signaling" gene signatures have been published (PMID 31953314) it would be of great interest to analyze the tumors for expression of such signature at baseline and any changes after SBRT. It may also be informative to measure soluble CD73 in the serum (see PMID 33361405).

3) In figure 3 a value of 1% for sTILs is chosen. Can the authors explain the reasons for this choice? In many studies evaluating the prognostic/predictive significance of sTILs much higher values have been used.

4) The most important endpoint for ICI is recurrence-free survival- an endpoint that was improved by ICI also in patients w/o pCR in prior studies. The authors should include a sentence in discussion about the importance of evaluating if the increase in pCR achieved by SBRT+ICI will reflect in improved recurrence-free survival. The latter will be likely required for regulatory approval.

Minor issues:

1) What is the positive control used for CD73 IHC, and is it possible to show in supplementary?
2) The 1% cutoff for PDL1 positivity refers to 1% tumor area or 1% of all immune cells present in the tumor? This difference may be important, especially for tumors with <1% sTILs.
3) Page 6, last paragraph, the sentence "We then examined two immune escape mechanisms that could be reversed by iSBRT, namely, expression of major histocompatibility index I (MHC-I) and CD73" should be modified, as CD73 expression is not reversed but could be exacerbated by SBRT.
4) Figure 1: in the middle panel under "Final Mammaprint status", "unevaluable after randomization" is repeated twice, is one instance supposed to be "Low risk after randomization"?
5) Ref 36 is an abstract reporting early results of the paper cited as ref 21. I suggest to remove ref 36 and cite here ref 21 instead.

(Remarks on figshare data availability)

Reviewer #3

(Remarks to the Author)
(A) In this study, De Caluwé et al. report on primary and translational results of a randomized, multicentre trial exploring the role of immune-modulating stereotactic body radiation therapy (iSBRT) and checkpoint inhibitors in addition to neoadjuvant chemotherapy in high-risk early ER+ HER2- breast cancer. Aiming to detect differences early, response at surgery, defined as residual cancer burden (RCB 0 and 1) as well as pathologic complete remission (pCR) define main primary and secondary endpoints comparing the 1:1:1 randomized arms. 147 patients randomized, the primary endpoint is negative while a secondary endpoint comparing the addition of the PD-L1 targeting checkpoint inhibitor Durvalumab and the CD73 targeting antibody Oleclumab to iSBRT/neoadjuvant chemotherapy (Arm 3) significantly improved pCR rates over Arm 1, which is standard neoadjuvant chemotherapy and iSBRT alone. In context, trying to capture regulation and overall quality of immunogenicity and tumor-specific immune responses in luminal (HR+) eBC are of critical relevance for both, translational and clinical breast cancer research. Interestingly, the authors report provocative results comparing to both phase III trials (CheckMate 7FL and Keynote 756) demonstrating benefit of the addition of checkpoint inhibition in PD-L1 negative but not in PD-L1 positive patients, which the author conclude to be a novel concept (neoadjuvant chemotherapy + iSBRT + anti-PD-L1 in PD-L1 negative luminal eBC). Together which some very granular findings on iSBRT effects on regional lymph nodes and correlation with other well established biomarker, this is new to the literature and my best knowledge (B). Providing a deep dive into biomarkers together with primary results very early (pCR) is a major strength which is partly made possible by analyzing on-treatment biopsies 1 week after iSBRT. In their conlusion, the authors report TME reprogramming toward an inflamed phenotype with iSBRT + ICI.

However, the scientific significance suffers (unfortunately) from the design of the study in many aspects.
1. Sample size considerations and assumptions are missing. Please add this information which will be informative if and how endpoints are appropriate (C, D).
2. The definition of endpoints is not precise (D). The Phase II design is certainly intended to measure early efficacy signals and is written in SAB and protocol that the question if Arm 2 or Arm 3 is superior against Arm 1. However, the definition of

primary and secondary endpoint lacks clarity when endpoints are met. Further, in the abstract, the authors (line 12) mention …the endpoint was achieved in 35,4%.. which is a description report on the pCR rate, however, is not sufficient to answer the research question.
3. I assume the authors are interested in two main aspects when the design was built, impact of iSBRT, impact of checkpoint inhibitor Durvalumab and impact of Durvalumab plus Oleclumab, each on both the microenvironment and on the surrogate endpoint pCR. Again, this bears great impact to the field of luminal eBC. However, it is not possible to draw any valid conclusion on a causal effect of iSBRT as there is no control arm without iSBRT and there is simultaneous Paclitaxel even in Arm 1 over more than 4 weeks which is possibly mediating similar effects (PMID: 30104241, 39009452). The impact of Paclitaxel is not mentioned in the manuscript. Concerning Arm 2 and 3, I would be grateful to understand sample size calculations and statistical plan as mentioned under 1. (C,D)
The analytical approach (C,D) is built on a comparison of PD-L1 positive to negative patients. The scientifically important finding, demonstration of meaningful benefit for add on Durvalumab in PD-L1 negative but not PD-L1 positive patient relies on a well justified (evidence based) PD-L1 threshold, with more implications for sample size calculations in terms of type I+II error and power. However, there is no consensus on PD-L1 scoring in luminal eBC and results from CheckMate 7FL and Keynote 756, showing increasing benefit with more PD-L1 expression is yet to be explained. Therefore, it would be mandatory to treat PD-L1 as a continuous or categorical instead of dichotomous variable.(E)
Minor: Definition of PD-L1 % expression and categorical IC scoring should be harmonized throughout the manuscript. It would also be appreciated to explain which a cut off of 1% using SP263 was chosen. Collectively, it remains unclear to me if the results are robust only one cutoff.
The other important finding is generated by the exploratory analysis including whole-transcriptome analysis and MammaPrint definition of subgroups. Low expression of immune signatures at baseline in PD-L1-negative patients are compared to biopsies 1 week after iSBRT and the authors report TME reprogramming toward an inflamed phenotype with iSBRT + ICI (line 19, not described in the results part). This corresponds to Fig. 4a which is hard to read. Please clarify what is shown. The title suggests two coloumns for the two timepoints but if so what is meant with PD-L1? Which arm is shown? Please provide data for all treatment arms.
Citation are appropriate. The manuscript is easy to read, well written and well structured in general. (G,H)

Thank you!

(Remarks on figshare data availability)
Please see my main comment on the trial design and validity to draw desired conclusions. The data itself seems to be in line with downstream analysis and may be reproducible.

Version 1:

Reviewer comments:

Reviewer #1

[redacted]

[redacted]

[redacted]

Reviewer #2

(Remarks to the Author)
The authors have addressed all of my prior concerns and the revised manuscript is much improved. While the new data presented add mechanistic depth and more clarity to the study, there is an issue with the presentation of data concerning the gene expression analyses.

I appreciate the testing of the "adenosine signaling" gene signature, which provides some evidence that increased adenosine signaling is associated with increased inflammation, increased Teff and increased CTLA4 expression (likely reflecting more activated Tregs) elicited by the combination of RT+anti-PDL1 (Fig 4e). While the lack of an increase in adenosine signaling in the presence of anti-CD73 suggests that the antibody is active at reducing adenosine generation, it is difficult to explain why there is lower inflammation and effector T cells in the presence of anti-CD73, given that the opposite would be expected and the responses were comparable in the two groups. The issue may be the imbalance in the samples available for sequencing between the two treatment arms: only 15% of samples are derived from responders in the arm with anti-CD73 versus 29% in the arm with only anti-PDL1. Thus, data should be interpretated with caution, and some discussion about this should be included in results and/or discussion sections. The fact that only samples with tumor still present at

week 6 were analyzed suggests that when anti-CD73 is added to SBRT +anti-PDL1 tumor rejection is faster and the best responders are thus excluded from the paired analysis. It also suggests that other barriers (not addressed by blocking CD73) are present in the non-responders. In this respect, B7-H3 stands out as a highly expressed inhibitory molecule irrespective of therapy arm. Can the authors verify if there is a significant increase in B7-H3 expression between baseline and post-SBRT, which would suggest that this may be an actionable radiation-induced barrier to tumor rejection?

I could not find in Methods, Results or figure legend a description of the genes that make up the adenosine signaling signature or the reference to the paper where it is described. Both should be provided. Moreover, in Figure 4 “d” and “e” it is written “adenosine” without any explanation about what this represents: this may be wrongly interpreted as adenosine itself being measured. The latter would be impossible in such materials but to avoid misinterpretations a clear definition is required in the figure legend.

In Fig 4d statistical significance is not shown by the color but by asterisks and the difference in adenosine signaling signature between PDL1<1% and >1% is highly significant. This directly contradicts the sentence in rebuttal “At baseline, no significant differences in adenosine pathway activity were observed between PD-L1–positive and PD-L1–negative tumors (Fig. 4d)”. Again, this deserves to be mentioned when describing the results.
Finally, a black square outline in Fig 4 indicates the correlation of each gene/signature with pCR but it is unclear if it is a positive or negative correlation. I found this display of the data somewhat confusing. A clarification in the figure legend or results section is needed.

(Remarks on figshare data availability)
I do not have expertise in working with R program

Reviewer #3

(Remarks to the Author)
The authors invested significant efforts, revised the manuscript extensively and replied in detail to all points raised. Overall, this led to a clear improvement of clarity and, specifically, robustness of data from my point of view (e.g. Fig 4; PD-L1 re-assesment). The approach using SBRTs plus ICI in HR+ HER2- eBC whithin a prospective protocol, including stratification of PD-L1 and further provision of in depth TME analysis provides significant data of unmet need. Thank you very much. Expect responses on comment #3, I can well follow modifications incl. additional data generated on PD-L1. However, the data, presentation and discussion need further modification to ensure that scientific conclusions reflect the actual data and to avoid exaggerated take aways. (E, H) For the fact that there is no control arm without iSBRT and there is simultaneous Paclitaxel over more than 4 weeks, it is (unfortunately) not possible to assign iSBRT any effect size on any endpoint. This is emphasized by the authors as a limitation stating (Discussion section, line 32 and following) “precludes disentangling the individual contributions of RT and concurrent systemic therapies —in particular paclitaxel and ICI— to TME modulation and pCR (39,40). Consequently, the observed effects should be interpreted as reflecting the combined impact of multimodal treatments, including their mutual synergistic effects, rather than a causal effect of iSBRT alone.” In contrast, the presentation and interpretation of data clearly suggests (Discussion section, line 2) “substantial evidence that iSBRT can prime tumours for response to anti-PD-L1, based on high response rates at surgery and upregulation of immune-related biomarkers one week after iSBRT”. Further, the authors conclude that (Discussion section) “In conclusion, the NeoCheckRay phase II study shows that adding iSBRT to ICI in the neoadjuvant setting increases pCR rates in ER+/HER2– BC and supports iSBRT as a promising immune-priming strategy, particularly in immunologically “cold” tumours””. Though I fully agree that this is a strong and well justified hypothesis generated from the data and cross-trial comparison, this could not (by trial design) and has not been shown by data.

(Remarks on figshare data availability)

Open Access This Peer Review File is licensed under a Creative Commons Attribution 4.0 International License, which permits use, sharing, adaptation, distribution and reproduction in any medium or format, as long as you give appropriate credit to the original author(s) and the source, provide a link to the Creative Commons license, and indicate if changes were made.
In cases where reviewers are anonymous, credit should be given to 'Anonymous Referee' and the source.
The images or other third party material in this Peer Review File are included in the article’s Creative Commons license, unless indicated otherwise in a credit line to the material. If material is not included in the article’s Creative Commons license and your intended use is not permitted by statutory regulation or exceeds the permitted use, you will need to obtain permission directly from the copyright holder.
To view a copy of this license, visit https://creativecommons.org/licenses/by/4.0/

Responses to Reviewers' Comments:

We thank the reviewers for their thorough evaluation of our manuscript and for their constructive, insightful comments, which have helped us significantly improve the clarity and quality of the work. We address each comment point by point below; our responses are in italics, and all corresponding changes have been incorporated into the revised manuscript.

[redacted]

[redacted]

[redacted]

[redacted]

[redacted]

[redacted]

[redacted]

[redacted]

[redacted]

[redacted]

[redacted]

[redacted]

Reviewer #2 (Remarks to the Author):

This manuscript reports the response at surgery of the patients enrolled in the Neo-CheckRay trial, a phase 2, randomized trial testing NACT plus radiation (8GyX3, SBRT) with or without durvalumab (anti-PD-L1) and with or without oleclumab (anti-CD73) in 147 high-risk early ER+/HER2- breast cancer patients.

In two prior phase III studies performed in similar patient populations (refs 11 and 12) addition of anti-PD1 (pembrolizumab or nivolumab) to NACT increased pCR rate from 15.6% to 24.3% and 13.8% to 24.5%, respectively. Therefore, the pCR rate of 16.7% observed here in the arm treated with SBRT and NACT without ICI seems to be in line with what could be expected in the absence of SBRT; in the arms receiving SBRT+anti-PD1 and SBRT+anti-PD1+anti-CD73 the pCR rate is higher (29.4 and 33.3%, respectively) but given the small number of patients in each arm this results do not provide evidence for a true benefit of SBRT addition to immunotherapy. Although in the pre-defined MammaPrint high population the difference between pCR in SBRT only versus SBRT+double_ICI achieves significance, this reflects the benefits of ICI. The strength of the study is in the analysis of responses based on baseline PD-L1 status: the latter demonstrates a remarkable pCR rate of 28.1% (Single_ICI), and 30.3% (Double_ICI) in PDL-1 negative tumors, which in the two phase III trials showed variable but much lower pCR to NACT+anti-PD1.

> ***Reply:*** *We appreciate the reviewer's analysis and fully agree that our trial's main strength is the central assessment and stratification by PD-L1. This approach clearly showed a notably high pCR rate in the PD-L1-negative subgroup, which was much higher than that observed in the PD-L1-negative subgroups of the CheckMate 7FL and KEYNOTE-756 trials.*

Thus, although the study was not randomized to compare the outcome in the presence vs absence of SBRT, results support the concept that the benefits of radiation used with ICI can be discerned in "cold tumors" but not in unselected patient populations. This interpretation is supported by a recent report in NSCLC (PMID: 40696153).

> ***Reply:*** *We thank the reviewer for this thoughtful observation. Although PMID 40696153 (Huang et al., Nature Cancer 2025) was conducted in metastatic NSCLC, their results align with ours. In that study, the combination of RT (3 × 8 Gy to one metastasis) with anti-PD-L1 demonstrated the greatest benefit in the PD-L1–negative subgroup, consistent with the concept that immunomodulatory radiation may preferentially augment immune checkpoint inhibition in immunologically "cold" tumours.*
>
> *Despite important differences between the studies—including disease setting (metastatic NSCLC vs neoadjuvant luminal B breast cancer in our trial), tumor type, and trial design—the convergence of results supports the hypothesis that the benefit of combining radiation with immune checkpoint blockade may be most pronounced in biologically selected (i.e., PD-L1–negative) tumors rather than in unselected patient populations. While these observations remain hypothesis-generating, together they reinforce the rationale for biomarker-guided use of immunomodulatory radiation therapy across disease contexts.*
>
> *We have added a reference to Huang et al. in the Discussion (page 20, line 35).*

It is also supported by the correlative studies performed on tumour biopsies showing an increase in PDL1 expression in the tumour 1week post-SBRT.

Overall, the study is very well described and provides critical new information of high relevance to BC treatment. Multiple exploratory analyses are performed, adding scientific depth. There are only a few issues that need to be addressed.

1) An intriguing result is the apparent loss of ICI efficacy in patients who received >1 Gy to the axilla level I lymph nodes. While multiple pre-clinical studies have demonstrated that radiation to draining nodes compromises tumor responses to ICI, axillary radiation was not performed in this study and the effects of the relatively low dose that was delivered to the nodes (likely due to the lateral location of the tumor) is concerning. Since all patients have at least sentinel node dissection it is important to analyse the lymph nodes for evidence of an effect of radiation or any changes (e.g., an increase in regulatory T cells?) that could explain the results.

> ***Reply**: We thank the reviewer for this insightful suggestion. Sentinel lymph node analyses were not prospectively planned in the original trial protocol; however, limited sentinel lymph node material was made available, yielding a total of 13 sentinel lymph nodes for exploratory analysis. We performed RNA sequencing on these samples and analysed the data according to the estimated axillary radiation dose (<1 Gy vs ≥1 Gy). Seven lymph nodes received <1 Gy, and six lymph nodes received ≥1 Gy.*
>
> *In the dataset, we did not observe statistically significant transcriptional differences that could readily explain the apparent attenuation of immune checkpoint inhibitor efficacy. Please find a volcano plot and a box plot comparing Treg signatures below (Rebuttal Figure 1).*
>
> *Importantly, this analysis was exploratory and likely underpowered, and the relatively long interval between 3x8 Gy and surgery (median 20 weeks) may have attenuated or normalized early radiation-induced immunologic changes in the draining lymph nodes. Future studies incorporating protocol-mandated lymph node sampling at earlier time points (e.g., 1–2 weeks post-RT) will be critical to more definitively characterize the impact of very low-dose incidental nodal irradiation on lymph node immune composition in humans, including potential effects on regulatory T-cell populations*

2) As acknowledged by the authors, there is no clear evidence of a benefit of anti-CD73. It is possible that the latter is effective only in a subset of the patients and the cohort analyzed here is too small to identify such signal. The only correlative study performed is tissue staining for CD73 expression. Given that “adenosine signaling” gene signatures have been published (PMID 31953314) it would be of great interest to analyze the tumors for expression of such signature at baseline and any changes after SBRT. It may also be informative to measure soluble CD73 in the serum (see PMID 33361405).

> ***Reply:** This is an important point. Targeting the adenosine pathway is central to our study, as RT has been shown to induce CD73 expression and promote immunosuppressive adenosine accumulation in the TME, which we aimed to counteract with the anti-CD73 antibody oleclumab.*
>
> *In response to this comment, we evaluated adenosine signalling using the published adenosine gene signature (PMID 31953314) and by analysing the expression of CD39*

(ENTPD1), CD73 (NT5E), and the canonical adenosine receptor gene ADORA2A. The RNA sequencing was performed at two timepoints: baseline (n=132) and one week after RT—enabling paired, within-patient analyses (n=75 pairs). These analyses are now included in Fig. 4d and Fig. 4e of the main manuscript as part of the exploratory translational results.

At baseline, no significant differences in adenosine pathway activity were observed between PD-L1–positive and PD-L1–negative tumors (Fig. 4d). At week 6 (iSBRT + 1 week), we observed a significant increase in the adenosine signature in iSBRT + anti-PD-L1 (Single_ICI), whereas the signature tended to decrease in iSBRT_only (No_ICI) and iSBRT + durvalumab + oleclumab (Double_ICI). This increase at week 6 was primarily driven by PD-L1–negative tumors (Fig. 4e).

In addition, as suggested, we measured circulating soluble CD73 (sCD73) at multiple timepoints (baseline, week 4, and week 6; n=124) in serum using the Abcam ab213761 ELISA kit. Baseline sCD73 levels were comparable to those measured in healthy donors (Rebuttal Figure 2a) and were well balanced across the 3 treatment arms (Rebuttal Figure 2b). This contrasts with our previous observations in metastatic TNBC, in which baseline sCD73 levels were elevated and associated with worse clinical outcomes. In the present trial of early luminal B breast cancer, baseline sCD73 levels were not associated with pCR at surgery (Rebuttal Figure 2c–d). Longitudinal analyses revealed a significant decrease in circulating sCD73 levels in iSBRT_only, particularly between baseline and week 6, in patients who did not achieve a pCR (Rebuttal Figure 2f; $P = 0.02$). In contrast, in patients treated with anti-CD73 (Double_ICI), a significant increase in sCD73 levels was observed between week 4 and week 6 (pre- and post-iSBRT) among patients who achieved a pCR (Rebuttal Figure 2h; $P = 0.02$). Importantly, this increase in sCD73 during the RT window (ΔsCD73 W4–W6) was associated with a higher probability of achieving pCR in Double_ICI (Rebuttal Figure 2k; $P = 0.0495$). No significant association between ΔsCD73 (W4–W6) and pCR was observed in the arms without anti-CD73 treatment.

Given the exploratory nature of these findings and the limited statistical power of these subgroup analyses, we suggest not including these data in the main manuscript, which focuses on the prespecified clinical and translational endpoints. We are currently pursuing further studies to more comprehensively investigate the dynamics of circulating sCD73 and other soluble biomarkers.

3) In figure 3 a value of 1% for sTILs is chosen. Can the authors explain the reasons for this choice? In many studies evaluating the prognostic/predictive significance of sTILs much higher values have been used.

Reply: *Stromal TIL levels are generally low in ER+/HER2− breast cancer; therefore, the 1% cutoff was chosen to reflect the distribution in this subtype and align with large phase III neoadjuvant trials, such as CheckMate 7FL, which used a <1% versus ≥1% threshold. However, because the association between sTILs and pCR is continuous, evaluating multiple cutoffs can indeed be indicated. Higher thresholds have been used in other ER+/HER2− studies, including GIADA and I-SPY2, which applied a 10% cutoff.*

In our cohort, 32.0% of patients had baseline sTIL levels ≥10%. pCR rates were 18.4% in patients with <10% sTILs compared with 42.6% in those with ≥10% sTILs. The median sTIL percentage was 10% in patients achieving pCR versus 1% in those without pCR. In response to the reviewer's suggestion, we have therefore added the 10% sTIL cutoff to the Forest plot in Fig. 3 and mention the 10% threshold in the results (page 6, lines 2-4), providing additional granularity across clinically relevant thresholds and facilitating comparison with prior studies.

4) The most important endpoint for ICI is recurrence-free survival- an endpoint that was improved by ICI also in patients w/o pCR in prior studies. The authors should include a sentence in discussion about the importance of evaluating if the increase in pCR achieved by SBRT+ICI will reflect in improved recurrence-free survival. The latter will be likely required for regulatory approval.

<u>Reply:</u> *We agree with the reviewer that while pCR is a useful early endpoint, recurrence-free/event-free survival (RFS/EFS) remains the clinically decisive endpoint for neoadjuvant ICI, including in patients who do not achieve pCR, as demonstrated in prior studies with chemo-immunotherapy. As such RFS/EFS, is also the endpoint on which regulatory approval is ultimately based. We have therefore added a sentence to the Discussion explicitly stating that longer follow-up and large phase III trials are required to determine whether the observed increase in pCR with SBRT+ICI translates into improved RFS/EFS (page 21, lines 36-39).*

<u>Minor issues:</u>

1) What is the positive control used for CD73 IHC, and is it possible to show in supplementary?

<u>Reply:</u> *The positive control for CD73 IHC was human tonsil tissue, used for staining optimization and included with each IHC run. The negative control was the same tonsil processed without the primary antibody. This information was added in the Materials & Methods section, and examples of positive and negative controls were added to Supp. Fig. 13. Similarly, we added examples of positive and negative controls for MHC-I to Supp. Fig. 12. The MHC-I controls were also performed on human tonsil tissue.*

2) The 1% cutoff for PDL1 positivity refers to 1% tumor area or 1% of all immune cells present in the tumor? This difference may be important, especially for tumors with <1% sTILs.

<u>Reply:</u> *PD-L1 was prospectively and centrally assessed using the IC-score, defined as the proportion of tumour area occupied by PD-L1-expressing immune cells within the TME. Patients were subsequently stratified by IC score <1% or ≥1%.*

To better assess PD-L1, we also retrospectively calculated the CPS (combined positive score), defined as the number of PD-L1–positive cells (tumour cells, lymphocytes, macrophages) divided by the total number of viable tumour cells, then multiplied by 100. The CPS score is hence provided along PD-L1 in Figure 3.

For translational analyses, we also retrospectively evaluated PD-L1 expression separately on both the immune and tumour compartments to assess the impact of treatments on each compartment, as shown in Supplementary Fig. 5.

3) Page 6, last paragraph, the sentence "We then examined two immune escape mechanisms that could be reversed by iSBRT, namely, expression of major histocompatibility index I (MHC-I) and CD73" should be modified, as CD73 expression is not reversed but could be exacerbated by SBRT.

   ***Reply**: We agree with this comment, and the sentence has been revised to avoid implying that CD73 expression is reversed by iSBRT. The sentence was revised to: "We then examined two immune escape mechanisms potentially modulated by iSBRT, namely major histocompatibility complex class I (MHC-I) expression and CD73-mediated adenosine signalling." (page 6, lines 22-23).*

4) Figure 1: in the middle panel under "Final Mammaprint status", "unevaluable after randomization" is repeated twice, is one instance supposed to be "Low risk after randomization"?

   ***Reply:** We thank the reviewer for identifying the typographical error. One of the two entries has been corrected to "MammaPrint Low Risk," without changing the numeric values.*

5) Ref 36 is an abstract reporting early results of the paper cited as ref 21. I suggest to remove ref 36 and cite here ref 21 instead.

   ***Reply:** We thank the reviewer for identifying this error. We have removed reference 36 and now cite reference 21 (Wennerberg et al., Cancer Immunol. Res., 2020) instead.*

Reviewer #3 (Remarks to the Author):

(A) In this study, De Caluwé et al. report on primary and translational results of a randomized, multicentre trial exploring the role of immune-modulating stereotactic body radiation therapy (iSBRT) and checkpoint inhibitors in addition to neoadjuvant chemotherapy in high-risk early ER+ HER2- breast cancer. Aiming to detect differences early, response at surgery, defined as residual cancer burden (RCB 0 and 1) as well as pathologic complete remission (pCR) define main primary and secondary endpoints comparing the 1:1:1 randomized arms. 147 patients randomized, the primary endpoint is negative while a secondary endpoint comparing the addition of the PD-L1 targeting checkpoint inhibitor Durvalumab and the CD73 targeting antibody Oleclumab to iSBRT/neoadjuvant chemotherapy (Arm 3) significantly improved pCR rates over Arm 1, which is standard neoadjuvant chemotherapy and iSBRT alone. In context, trying to capture regulation and overall quality of immunogenicity and tumor-specific immune responses in luminal (HR+) eBC are of critical relevance for both, translational and clinical breast cancer research. Interestingly, the authors report provocative results comparing to both phase III trials (CheckMate 7FL and KEYNOTE-756) demonstrating benefit of the addition of checkpoint inhibition in PD-L1 negative but not in PD-L1 positive patients, which the author conclude to be a novel concept (neoadjuvant chemotherapy + iSBRT + anti-PD-L1 in PD-L1 negative luminal eBC). Together which some very granular findings on iSBRT effects on regional lymph nodes and correlation with other well established biomarker, this is new to the literature and my best knowledge (B). Providing a deep dive into biomarkers together with primary results very early (pCR) is a major strength which is partly made possible by analyzing on-treatment biopsies 1 week after iSBRT. In their conclusion, the authors report TME reprogramming toward an inflamed phenotype with iSBRT + ICI.

However, the scientific significance suffers (unfortunately) from the design of the study in many aspects.

1. Sample size considerations and assumptions are missing. Please add this information which will be informative if and how endpoints are appropriate (C, D).

> ***Reply:*** *We thank the reviewer for this important comment. We agree that explicit reporting of the sample size assumptions is essential to interpret the appropriateness of the endpoints in this phase II trial.*
>
> *Sample size justification and statistical assumptions were prespecified in the statistical analysis plan and have now been clarified and made more explicit in the Methods section. Briefly, the trial was powered for the primary endpoint (RCB 0/1) using a binomial superiority two-sample design, assuming an RCB 0/1 rate of 15% in the No_ICI arm and targeting an absolute improvement of 30 percentage points in either experimental arm. With 132 evaluable patients, the study had 80% power at a two-sided α level of 0.025 per comparison, applying a Bonferroni correction for the two primary comparisons (Single_ICI vs No_ICI and Double_ICI vs No_ICI). No formal comparison between Single_ICI and Double_ICI was planned.*
>
> *We have revised the manuscript to explicitly link these assumptions to the definitions of the primary and secondary endpoints and to clarify the hypothesis-testing framework underlying the study design (page 30, lines 16-26).*

2. The definition of endpoints is not precise (D). The Phase design is certainly intended to measure early efficacy signals and is written in SAB and protocol that the question if Arm 2 or Arm 3 is superior against Arm 1. However, the definition of primary and secondary endpoint lacks clarity when endpoints are met. Further, in the abstract, the authors (line 12) mention …the endpoint was achieved in 35,4%.. which is a description report on the pCR rate, however, is not sufficient to answer the research question.

   Reply: *We agree that the decision framework of this randomized phase II study requires clearer articulation.*

   The primary endpoint of the trial was the proportion of patients achieving RCB 0 or 1 at surgery. The study was designed as a signal-seeking phase II trial to evaluate whether either experimental arm (Single-ICI or Double-ICI) demonstrated superiority over the control arm (No_ICI). Superiority was assessed through prespecified pairwise comparisons of each experimental arm versus the control arm only; no formal comparison between the two experimental arms was planned.

   Secondary endpoints, including pCR, were predefined as efficacy measures.

   We have now clarified in the Methods section that achievement of the primary endpoint refers to demonstrating a statistically significant increase in the rate of RCB 0/1 in at least one experimental arm compared with the control arm, consistent with the phase II objective of identifying early efficacy signals (page 30 lines 16-26).

   In addition, the Abstract has been revised to avoid descriptive reporting of response rates and to reflect the comparative nature of the primary endpoint (page 2, lines 12-15).

3. I assume the authors are interested in two main aspects when the design was built, impact of iSBRT, impact of checkpoint inhibitor Durvalumab and impact of Durvalumab plus Oleclumab, each on both the microenvironment and on the surrogate endpoint pCR. Again, this bears great impact to the field of luminal eBC. However, it is not possible to draw any valid conclusion on a causal effect of iSBRT as there is no control arm without iSBRT and there is simultaneous Paclitaxel even in Arm 1 over more than 4 weeks which is possibly mediating similar effects (PMID: 30104241, 39009452). The impact of Paclitaxel is not mentioned in the manuscript.

   Reply: *We thank the reviewer for this thoughtful comment and agree that the absence of a control arm without iSBRT is a limitation, as it prevents definitive assessment of the independent additional effect of iSBRT. However, the uniform use of iSBRT across all treatment arms was intended to avoid imbalances in local treatment and to ensure that comparisons of pCR rates primarily reflected differences in systemic therapy.*

   Similarly, because paclitaxel was administered in all arms, we acknowledge that some of the observed biological effects on the TME and on pCR may be mediated, at least in part, by chemotherapy rather than by iSBRT +/- ICI. These

limitations —namely the inability to evaluate the isolated impact of iSBRT— were already acknowledged in the Discussion and have been further clarified in the revised manuscript including the two references on paclitaxel effects on TAMs (page 21 lines 32-36).

Importantly, although we included on-treatment biopsies one week after iSBRT, the absence of corresponding biopsies from patients not exposed to iSBRT precludes assessment of its isolated biological impact at this time point. Nevertheless, the availability of paired on-treatment biopsies represents a major strength of our study, as it enables a unique analysis of early dynamic changes in the TME. Such biopsies are rarely feasible in randomized trials, which limits direct comparison with the existing literature.

Concerning Arm 2 and 3, I would be grateful to understand sample size calculations and statistical plan as mentioned under 1. (C,D) .

Reply: *This point is addressed under points 1 and 3 higher in the rebuttal.*

The analytical approach (C,D) is built on a comparison of PD-L1 positive to negative patients. The scientifically important finding, demonstration of meaningful benefit for add on Durvalumab in PD-L1 negative but not PD-L1 positive patient relies on a well justified (evidence based) PD-L1 threshold, with more implications for sample size calculations in terms of type I+II error and power. However, there is no consensus on PD-L1 scoring in luminal eBC and results from CheckMate 7FL and KEYNOTE-756, showing increasing benefit with more PD-L1 expression is yet to be explained. Therefore, it would be mandatory to treat PD-L1 as a continuous or categorical instead of dichotomous variable (E).

Minor: Definition of PD-L1 % expression and categorical IC scoring should be harmonized throughout the manuscript. It would also be appreciated to explain which a cut off of 1% using SP263 was chosen. Collectively, it remains unclear to me if the results are robust only one cutoff.

Reply:

We acknowledge that there is currently no consensus on PD-L1 scoring in luminal early breast cancer, and that recent results from CheckMate 7FL and KEYNOTE-756 suggest increasing benefit with higher PD-L1 expression. We therefore agree that analyses treating PD-L1 as a continuous or categorical variable are of interest.

In the present trial, PD-L1 was prospectively and centrally assessed prior to randomization as a dichotomous variable (IC ≥1% vs <1%) for stratification purposes, based on a pre-specified, evidence-based cut-off. This approach was chosen to ensure balanced randomization and to maintain consistency with major neoadjuvant trials in this setting, particularly CheckMate 7FL.

In exploratory analyses, we also evaluated PD-L1 using the CPS score, both as a continuous variable (Supplementary Fig. 5) and with a cutoff of 1, as applied in KEYNOTE-756. To provide additional granularity, we have added analyses using a CPS cutoff of 10 (Supplementary Fig. 3), which show higher pCR rates at higher CPS levels. Furthermore, for translational analyses, PD-L1 was re-scored as a continuous

variable in both the immune and tumor compartments to assess dynamic changes (Supplementary Fig. 5).

Across all PD-L1 assessment approaches, the addition of ICI to iSBRT was associated with the greatest benefit in PD-L1–negative tumors, supporting the robustness of this finding irrespective of the scoring method used.

We considered modelling the IC score across multiple additional thresholds; however, such post hoc analyses were not prespecified and could increase the risk of type I error.

Following the reviewer's comment, we have harmonized PD-L1 terminology and clarified the scoring methodology and rationale for the selected cutoff throughout the manuscript. Unless otherwise specified, PD-L1 negativity refers to IC <1% and positivity to IC ≥1%, consistent with the prospective stratification used in our trial.

The other important finding is generated by the exploratory analysis including whole-transcriptome analysis and MammaPrint definition of subgroups. Low expression of immune signatures at baseline in PD-L1-negative patients are compared to biopsies 1 week after iSBRT and the authors report TME reprogramming toward an inflamed phenotype with iSBRT + ICI (line 19, not described in the results part). This corresponds to Fig. 4a which is hard to read. Please clarify what is shown. The title suggests two columns for the two timepoints but if so, what is meant with PD-L1? Which arm is shown? Please provide data for all treatment arms.

Reply: *We thank the reviewer for this comment and agree that the initial presentation of Figure 4a lacked clarity and may have led to misinterpretation. We have revised the figure to improve readability and have clarified the legend and Results text accordingly.*

In response, we have now completed RNA sequencing for all baseline samples (n = 132) and for on-treatment biopsies obtained one week after iSBRT in which tumour cells were present (n = 75), enabling paired analyses. Figure 4 has been redesigned to explicitly display longitudinal changes between baseline and week 6, with the 3 treatment arms presented separately to detail treatment effects. The figure now clearly distinguishes immunohistochemistry findings (top panel) from transcriptomic results (bottom panel).

The revised figure clearly indicates the time points analysed, the PD-L1 subgrouping, and the corresponding treatment arms. In addition, we have expanded the Results section to explicitly describe these findings and to clarify the observed reprogramming of the tumour microenvironment toward an inflamed phenotype in PD-L1–negative tumours following iSBRT plus ICI.

We believe that these revisions substantially improve the clarity, interpretability, and robustness of the presented data.

Citation are appropriate. The manuscript is easy to read, well written and well structured in general. (G,H)

Reply: *We thank the reviewer for this positive feedback.*

Thank you!

Reviewer #3 (Remarks on figshare data availability):

Please see my main comment on the trial design and validity to draw desired conclusions. The data itself seems to be in line with downstream analysis and may be reproducible.

> ***Reply:*** *We thank the reviewer for their assessment of the data availability. We appreciate the comment that the shared datasets are consistent with the downstream analyses and support reproducibility. Concerns related to trial design and the interpretation of conclusions are addressed in detail in our response to the main comment and have been further clarified in the revised manuscript.*

Rebuttal Table 1 | Adjusted analysis accounting for baseline factors.

Without adjustment:

	Events/Total	OddsRatio (95% CI)	P-value
	39/147		
**ARM**			0.1625[2]
1: Without Immunotherapy	8/48	Reference	
2: Durvalumab	15/51	2.08 (0.79-5.49)	0.48411
3: Durvalumab+Oleclumab	16/48	2.50 (0.95-6.58)	0.16281

[1] Covariate Wald p-value; [2] Type 3 Wald p-value

Accounting for T-stage (cT3 vs. cT1cT2):

	Events/Total	Odds Ratio (95% CI)	P-value
	39/147		
**ARM**			0.1229[2]
1: Without Immunotherapy	8/48	Reference	
2: Durvalumab	15/51	2.39 (0.89-6.44)	0.3372[1]
3: Durvalumab+Oleclumab	16/48	2.63 (0.98-7.01)	0.1868[1]
**Primary Tumour Size**			0.0247[2]
cT1-2	35/113	3.66 (1.18-11.35)	0.0247[1]
cT3	4/34	Reference	

[1] Covariate Wald p-value; [2] Type 3 Wald p-value

Accounting for the MammaPrint:

	Events/Total	Odds Ratio (95% CI)	P-value
	39/147		
**ARM**			0.0638[2]
1: Without Immunotherapy	8/48	Reference	
2: Durvalumab	15/51	3.12 (1.07-9.03)	0.1904[1]
3: Durvalumab+Oleclumab	16/48	3.13 (1.09-8.96)	0.1782[1]
**MammaPrint**			0.0071[2]
ULTRA HIGH RISK	17/35	Reference	
HIGH RISK	20/98	0.23 (0.09-0.54)	0.9730[1]
LOW RISK	0/10	0.00 (0.00-I)	0.9604[1]
UNEVALUABLE	2/4	1.20 (0.14-10.59)	0.9530[1]

[1] Covariate Wald p-value; [2] Type 3 Wald p-value

Rebuttal Fig. 1 | Gene expression in sentinel lymph nodes at surgery, stratified by pre-operative axillary radiation dose contribution from iSBRT.

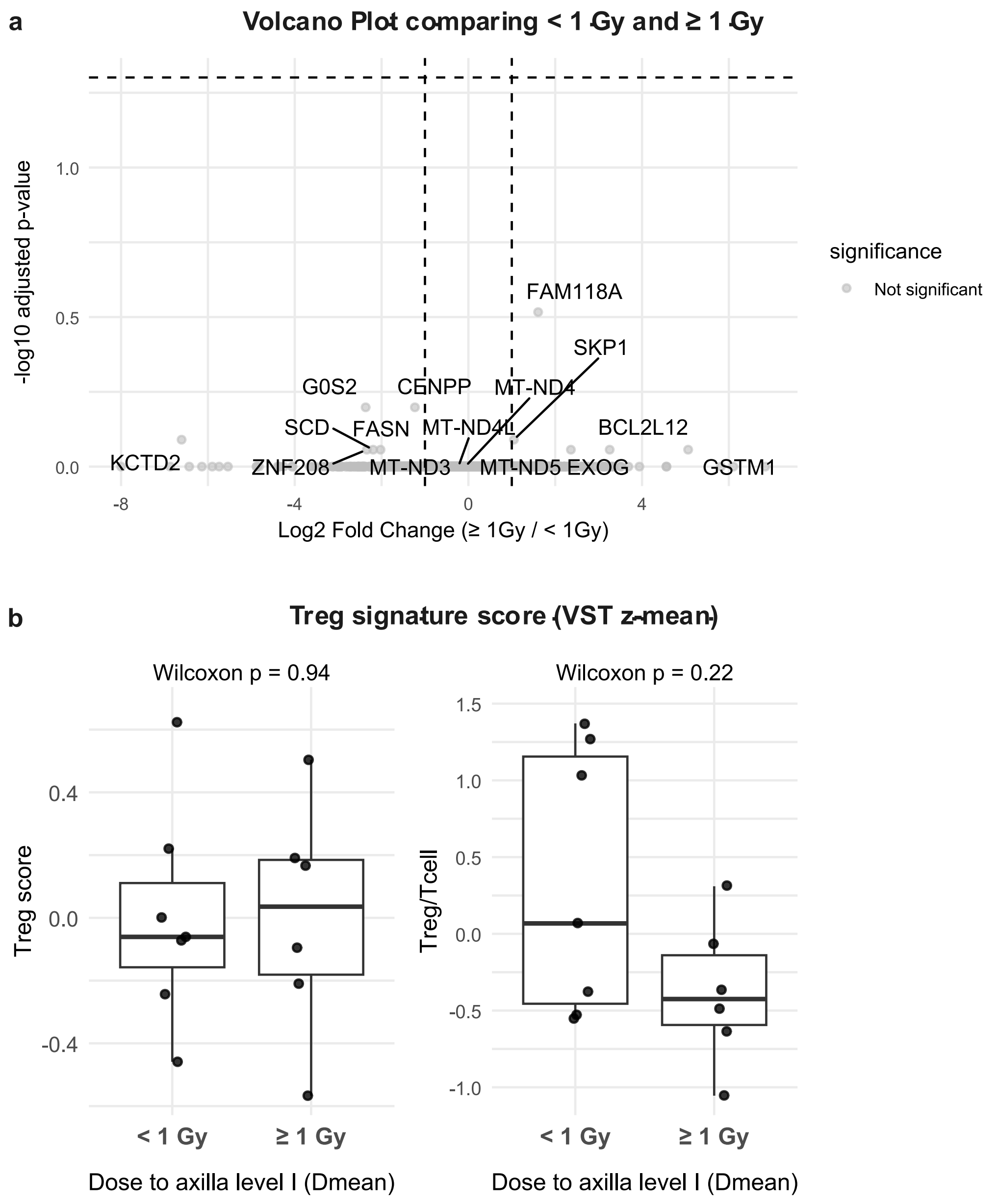

Rebuttal Fig. 1 | Gene expression in sentinel lymph nodes at surgery, stratified by pre-operative axillary radiation dose contribution from iSBRT. a, Volcano plot of differentially expressed genes showing bulk RNA-seq genes between sentinel lymph nodes from patients treated with < 1 Gy and ≥ 1 Gy axillary radiation dose. **b**, Box plots of T reg signatures expression in sentinel lymph nodes from patients treated with < 1 Gy and ≥ 1 Gy axillary radiation dose.

Rebuttal Fig. 2 | Distribution, longitudinal dynamics and predictive value of circulating soluble CD73 (sCD73).

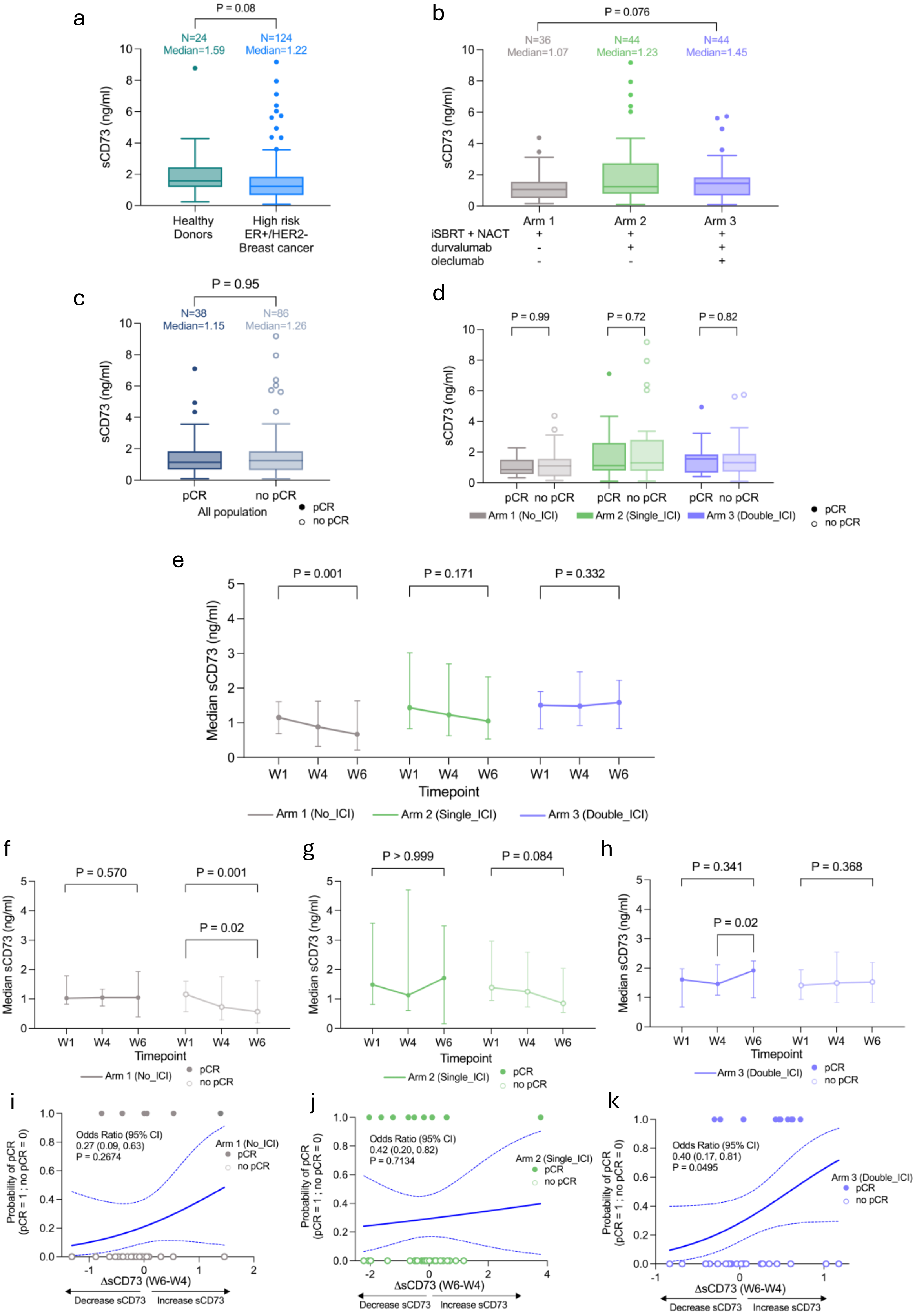

Rebuttal Fig. 2 | Distribution, longitudinal dynamics and predictive value of circulating soluble CD73 (sCD73). sCD73 levels were measured by ELISA in serum at baseline (W1), week 4 (W4) and week 6 (W6).

(a–d) Baseline distribution of sCD73 levels in healthy donors and the Neo-CheckRay cohort (a), across treatment arms (b), and according to pathological response (pCR) in the overall population and by treatment arm (c–d). Data are shown as Tukey box-and-whisker plots (median, interquartile range). Group comparisons were performed using Kruskal–Wallis tests with Mann–Whitney tests for pairwise comparisons, as indicated.

(e–h) Longitudinal evolution of circulating sCD73 levels across W1, W4 and W6 according to treatment arm (e) and pathological response at surgery (f–h). Lines represent median values with interquartile range. Overall time effects were assessed using Friedman's test, with selected pre-specified pairwise comparisons assessed using paired Wilcoxon signed-rank tests.

(i–k) Logistic regression analyses showing the association between changes in sCD73 levels between W4 and W6 (ΔsCD73 W4–W6) and probability of achieving pCR in each treatment arm. Solid lines represent predicted probabilities, and dashed lines indicate 95% confidence intervals.

Responses to Reviewers' Comments:

We thank the reviewers for their thorough evaluation of our revised manuscript and for their comments. We address each comment point by point below; our responses are in italics, and all corresponding changes have been incorporated into the revised manuscript.

Reviewer #1 (Remarks to the Author):

[redacted]

[redacted]

[redacted]

[redacted]

[redacted]

[redacted]

[redacted]

[redacted]

[redacted]

Reviewer #2 (Remarks to the Author):

The authors have addressed all of my prior concerns and the revised manuscript is much improved.

Reply: *Thank you.*

While the new data presented add mechanistic depth and more clarity to the study, there is an issue with the presentation of data concerning gene expression analyses.

I appreciate the testing of the “adenosine signaling” gene signature, which provides some evidence that increased adenosine signaling is associated with increased inflammation, increased Teff and increased CTLA4 expression (likely reflecting more activated Tregs) elicited by the combination of RT+anti-PD-L1 (Fig 4e). While the lack of an increase in adenosine signaling in the presence of anti-CD73 suggests that the antibody is active at reducing adenosine generation, it is difficult to explain why there is lower inflammation and effector T cells in the presence of anti-CD73, given that the opposite would be expected and the responses were comparable in the two groups. The issue may be the imbalance in the samples available for sequencing between the two treatment arms: only 15% of samples are derived from responders in the arm with anti-CD73 versus 29% in the arm with only anti-PD-L1. Thus, data should be interpreted with caution, and some discussion about this should be included in results and/or discussion sections. The fact that only samples with tumour still present at week 6 were analyzed suggests that when anti-CD73 is added to SBRT +anti-PD-L1 tumour rejection is faster and the best responders are thus excluded from the paired analysis.

Reply: *We thank the reviewer for this insightful comment. We agree that the observed differences in inflammatory and effector T cell–related signatures in the anti-CD73 arm appear counterintuitive and warrant cautious interpretation. As correctly noted, this analysis relies on paired biopsies (baseline and on-treatment) and therefore includes only patients with residual tumour at the on-treatment timepoint and hence introduces a potential selection bias. The lower number of paired biopsies and lower proportion of responders represented in the anti-CD73 arm (Double_ICI) may reflect more rapid tumour clearance in this group, leading to the exclusion of the best responders from the paired transcriptomic analyses. Consequently, the analyzed samples in this arm may be enriched for relatively less responsive tumours, which could in turn contribute to the lower levels of inflammation and effector T cell signatures observed, compared to the Single_ICI arm where tumour regression may have occurred more gradually. We have now explicitly acknowledged this limitation in the revised manuscript (Discussion section) and emphasize that these findings should be interpreted with appropriate caution.*

It also suggests that other barriers (not addressed by blocking CD73) are present in the non-responders. In this respect, B7-H3 stands out as a highly expressed inhibitory molecule irrespective of therapy arm. Can the authors verify if there is a significant increase in B7-H3 expression between baseline and post-SBRT, which would suggest that this may be an actionable radiation-induced barrier to tumour rejection?

Reply: *We confirm that B7-H3 expression increases significantly between baseline and week 6 across treatment arms and represents one of the most pronounced transcriptomic changes observed (Fig. 4d). In subgroup analyses, B7-H3 expression showed a numerical*

increase in baseline PD-L1–positive tumours treated with anti–PD-L1, but this change did not reach statistical significance. We agree with the reviewer that B7-H3 is a potential radiation-induced immunoregulatory barrier and a rational target for future combination strategies.

I could not find in Methods, Results or figure legend a description of the genes that make up the adenosine signaling signature or the reference to the paper where it is described. Both should be provided. Moreover, in Figure 4 "d" and "e" it is written "adenosine" without any explanation about what this represents: this may be wrongly interpreted as adenosine itself being measured. The latter would be impossible in such materials but to avoid misinterpretations a clear definition is required in the figure legend.

***Reply**: We agree that the term "adenosine" in Fig. 4c,d could be misleading. We have therefore revised the figure and its legend to clarify that gene names are shown in uppercase italics, whereas signatures are presented in bold plain text.*

For each signature discussed in Fig. 4c-d, we have also added references to their original publications in the legend and in the Methods section. In addition, we created a new Supplementary Figure (Supplementary Fig. 15) detailing the gene sets for each signature. Specifically, the adenosine signature consists of 14 genes (PPARG, CYBB, COL3A1, FOXP3, LAG3, APP, CD81, GPI, PTGS2, CASP1, FOS, MAPK1, MAPK3 and CREB1), whose concordant activity is indicative of adenosine signaling, as described by Sidders et al. (Clin Cancer Res 2020).

In Fig 4d statistical significance is not shown by the color but by asterisks and the difference in adenosine signaling signature between PD-L1<1% and >1% is highly significant. This directly contradicts the sentence in rebuttal "At baseline, no significant differences in adenosine pathway activity were observed between PD-L1–positive and PD-L1–negative tumours (Fig. 4d)". Again, this deserves to be mentioned when describing the results.

***Reply**: We acknowledge that the adenosine signalling signature is significantly higher in PD-L1–positive tumours at baseline (Fig. 4c) and have revised the Results section accordingly. This observation is consistent with previous reports, including Ben Sidders et al. Clin Cancer Res 2020, showing that adenosine signaling is associated with immune-infiltrated but immunosuppressed TME. In this context, increased adenosine signaling activity in PD-L1–positive tumours likely reflect a compensatory immunosuppressive mechanism that counterbalances ongoing immune activation.*

In addition, our dynamic analyses further support this interpretation. We observed that the adenosine signaling signature was upregulated upon treatment in the Single_ICI arm, which also displayed the higher levels of immune activation, as well as in baseline PD-L1–negative tumours treated with ICI. In contrast, a relative downregulation of this pathway was observed in the Double_ICI arm compared to Single_ICI. We have added a sentence to the Results to draw the reader's attention.

Altogether, these findings suggest that ICI-induced immune activation may trigger adaptive immunoregulatory mechanisms, including adenosine signaling, highlighting a dynamic balance between immune stimulation and suppression within the TME.

Finally, a black square outline in Fig 4 indicates the correlation of each gene/signature with

pCR but it is unclear if it is a positive or negative correlation. I found this display of the data somewhat confusing. A clarification in the figure legend or results section is needed.

Reply : *We thank the reviewer for highlighting this important point. We agree that the representation of correlations using black square outlines was not sufficiently clear. At baseline (Fig. 4c), black squares denote features associated with an increased likelihood of pCR. The legend and the caption has been revised to clarify that these markers reflect a positive association with pCR (↑ pCR).*

Regarding the on-treatment analysis (shown in Fig. 4d), we acknowledge that the interpretation of the correlation with pCR is hampered by selection bias, as a substantial proportion of patients achieving pCR—particularly in the Double_ICI arm—no longer had residual tumour at week 6 and were therefore not included in the paired analysis. As a result, the dataset is not representative of the full pCR population, making these exploratory correlations difficult to interpret reliably, as correctly pointed out by the reviewer. In light of this limitation and to avoid potential confusion, we have removed the black square annotations from Fig. 4d and removed this information from the Results.

As the baseline data represents the ITT population and is not subject to potential selection bias arising from the exclusion of cases without residual tumour in the on-treatment biopsy, the black squares indicating significant correlation with pCR increase are retained for the baseline transcriptomic features in Fig. 4c.

Reviewer #2 (Remarks on figshare data availability): I do not have expertise in working with R program.

Reply*: To improve accessibility of the data, we have now uploaded a CSV file containing the dataset used for the primary analysis. This file is included in the revised data sharing materials and allows straightforward access without the need for R-specific tools. In addition, we have provided separate Excel files containing the source data for each figure to ensure the data is as accessible and transparent as possible.*

Reviewer #3 (Remarks to the Author):

The authors invested significant efforts, revised the manuscript extensively and replied in detail to all points raised. Overall, this led to a clear improvement of clarity and, specifically, robustness of data from my point of view (e.g. Fig 4; PD-L1 re-assesment). The approach using SBRTs plus ICI in HR+ HER2- eBC within a prospective protocol, including stratification of PD-L1 and further provision of in depth TME analysis provides significant data of unmet need. Thank you very much.

Reply*: Thank you.*

Expect responses on comment #3, I can well follow modifications incl. additional data generated on PD-L1. However, the data, presentation and discussion need further modification to ensure that scientific conclusions reflect the actual data and to avoid exaggerated take aways. (E, H) For the fact that there is no control arm without iSBRT and there is simultaneous Paclitaxel over more than 4 weeks, it is (unfortunately) not possible to

assign iSBRT any effect size on any endpoint. This is emphasized by the authors as a limitation stating (Discussion section, line 32 and following) "precludes disentangling the individual contributions of RT and concurrent systemic therapies —in particular paclitaxel and ICI— to TME modulation and pCR (39,40). Consequently, the observed effects should be interpreted as reflecting the combined impact of multimodal treatments, including their mutual synergistic effects, rather than a causal effect of iSBRT alone." In contrast, the presentation and interpretation of data clearly suggest (Discussion section, line 2) "substantial evidence that iSBRT can prime tumours for response to anti-PD-L1, based on high response rates at surgery and upregulation of immune-related biomarkers one week after iSBRT". Further, the authors conclude that (Discussion section) "In conclusion, the NeoCheckRay phase II study shows that adding iSBRT to ICI in the neoadjuvant setting increases pCR rates in ER+/HER2− BC and supports iSBRT as a promising immune-priming strategy, particularly in immunologically "cold" tumours"". Though I fully agree that this is a strong and well justified hypothesis generated from the data and cross-trial comparison, this could not (by trial design) and has not been shown by data.

Reply : *We thank the reviewer for this important comment. We agree that our original wording could be interpreted as over-attributing causal effects to iSBRT. We have therefore revised the manuscript to avoid causal claims and to consistently frame our findings as reflecting the combined effects of multimodal therapy (iSBRT, chemotherapy, and ICI), in line with the study design, which does not include a control arm without iSBRT.*

We have softened mechanistic interpretations throughout the Discussion, replacing them with more cautious language (e.g., "associated with" or "consistent with") and explicitly stating that the individual contribution of iSBRT cannot be isolated and remains hypothesis-generating (Discussion section).

Finally, we have revised the Conclusion to align with these limitations and to ensure that all statements strictly reflect the data. The revised Conclusion now emphasizes association rather than causation and highlights that the observed immune activation is consistent with, but does not demonstrate, a potential immune-priming effect in the context of multimodal therapy (Discussion section).
